# Influence of an industrial discharge on long-term dynamics of abiotic and biotic resources in Lavaca Bay, Texas, USA

**DOI:** 10.1007/s10661-022-10665-w

**Published:** 2022-10-27

**Authors:** Elizabeth K. Harris, Paul A. Montagna, Audrey R. Douglas, Lisa Vitale, David Buzan

**Affiliations:** 1 Harte Research Institute for Gulf of Mexico Studies, Texas A&M University-Corpus Christi, 6300 Ocean Drive, Unit 5869, Corpus Christi, TX 78412 USA; 2 Freese and Nichols, Inc, 10431 Morado Circle, Bldg. 5, Suite 300, Austin, TX 78759 USA

**Keywords:** Plankton, Infauna, Nekton, Water quality, Sediment quality, Formosa Plastics, Estuary

## Abstract

The current study seeks to identify possible anthropogenic and/or natural environmental stressors that may account for the long-term decline of ecosystem health in Lavaca Bay, Texas, USA. The Formosa Plastics Corporation instituted monitoring of an industrial discharge into the bay with 16 fixed point stations and quarterly sampling from 1993 to 2020. Comprehensive measurements included organic and inorganic solutes in surface water, porewater and sediment, sediment content, plankton, nekton, and infaunal benthos. All parameter trends changed over time due to climate, freshwater inflow events, and/or seasonal changes. Biological community structure and sediment changed with distance from the discharge site. Dominance characterized community structure because three to four taxa comprised > 70% of individuals for nekton (trawl and gill net), phytoplankton, zooplankton, and ichthyoplankton samples. Sediment became sandier over time (48 to 75%) and away from the discharge. Surface water and porewater at reference (R) stations and stations near the discharge site had similar hydrographical and biological trends over time, indicating no long-term impact due to the discharge. However, 99.9% of 424,671 measurements of organic contaminants were non-detectable because the methods were insensitive to ambient concentrations. Thus, it is still not known if contaminants play a role in the long-term decline of ecosystem health in Lavaca Bay. Furthermore, only four R stations were sampled and were all 3810 m from the discharge site, so it is possible that trends in R stations do not represent the natural background. Future studies should include more R stations and lower detection limits for contaminants.

**Supplementary Information:**

The online version contains supplementary material available at 10.1007/s10661-022-10665-w.

## Introduction

Long-term declines in benthic community dynamics (abundance, biomass, and diversity) in Lavaca Bay, Texas, USA, have been noted since 1988 (Hardegree, 2018; Montagna et al., 2020; Pollack et al., 2011). Thus, there is a need to determine the possible mechanisms (i.e., anthropogenic and/or natural environmental stressors) that may influence the decline of ecological health in Lavaca Bay. Initially, benthic macrofauna response was linked to climatic variability because of the response of salinity patterns to Oceanic Niño Index, North Atlantic Oscillation, and North Pacific Index. However, a later study comparing the Lavaca-Colorado Estuary (LCE) and adjacent Nueces Estuary and Guadalupe Estuary found long-term declines in benthic community dynamics in the LCE only (Hardegree, 2018).

Environmental stressors are defined as biological, chemical, or physical factors that have an adverse effect on habitat quality and its biotic components (Toft et al., 2018). Environmental stressors, or disturbances, occur in many forms and can be either natural or anthropogenic in origin. Natural stressors may be extreme weather events (i.e., hurricanes, drought), diseases, parasites, fluctuating salinity regimes, and low dissolved oxygen (DO) levels (Montagna et al., 2020). Anthropogenic stressors are human-induced and may be expressed in forms by chemical pollution, upstream water diversions, excessive nutrient loading (resulting in eutrophication), and resource exploitation (Cardoso et al., 2008; Pollack et al., 2011). Multiple stressors, natural and anthropogenic in origin, are increasingly affecting biological community structure, therefore transforming ecosystems’ condition and functionality (Bruder et al., 2019). Most studies measure the direct effects of multiple stressors and their interactions on community dynamics of select biological communities within one trophic level. This approach generates useful information for management and restoration efforts of stressed environments. However, conducting a study that analyzes data by one community type (i.e., fish, benthos, and/or phytoplankton), or trophic level, minimizes the importance of community on system dynamics, or biotic interactions, within the trophic pyramid and the changes stressor effects have on trophic dynamics (Bruder et al., 2019; Zhang et al., 2016). The interaction between multiple stressors influences biotic interactions and may create new stressor interactions or minimize or strengthen stressor effects.

The goal here is to identify possible mechanisms or the driving force(s) that may be contributing to changes in the ecological condition of Lavaca Bay over time. Questions include as follows: (1) Is Lavaca Bay still exhibiting benthic declines? (2) What stressors are influencing biotic community dynamics? (3) Are biotic community trends acting in unison (synergistically), or do they act independently (antagonistically), and does that interaction type influence variation explained in community dynamics? An opportunity is provided by examining a long-term monitoring dataset that includes hydrological, biological, and chemical data that was collected over a 27-year time frame (1993–2020) for the purpose of monitoring water conditions at a discharge site erected by Formosa Plastics Corporation (FPC), Texas, Point Comfort facility. Data collection near the point of discharge is to ensure adequate dilution of the plant’s wastewater and was established to meet the objectives of the Texas Commission on Environmental Quality (TCEQ) and the US Environmental Protection Agency (USEPA) as outlined in TCEQ Wastewater Permit # 02,436, USEPA Permit # TX0085570. FPC is located near the northeastern shore of Lavaca Bay and produces polyvinyl chloride, polyethylene, and polypropylene resins and other additional plastic products. The dataset is comprehensive and includes in situ physical–chemical parameters, priority pollutants, and biological data.

## Methods

In May 1993, the Receiving Water Monitoring Program, Lavaca Bay (Monitoring Program), submitted the *Receiving Water Monitoring Program, Scope of Work for the Formosa Plastics Corporation, TX, Point Comfort, Texas Facility* to the Texas Water Commission (now the TCEQ) to satisfy permit requirements. Following additional revisions and a final approval, monitoring trips began in May of 1993 and continue today (as of April 2022).

The monitoring program was supervised by four consulting firms over 23 years. However, no staff turnover occurred because the name differences were due to buyouts. Subcontractor groups completed external analyses (i.e., chemical detection and bioassays). The list of subcontractors can be found in Table S1. The study is ongoing as of publication.

### Study area

Lavaca Bay is a secondary bay within the LCE, which is the second largest estuarine system located along the Texas Gulf Coast (Yamada & Armstrong, 1982). Lavaca Bay is a small (190 km^2^) lagoon-like ecosystem in a sub-tropical humid climate with mean annual precipitation of 107.2 cm and an average depth of 1.2 m, except in the ship channel, which has a depth of 10.5 m. Average inflow to Lavaca Bay (for the period from 1977 to 2016) was approximately 1.3 million acre-feet per year (1.6 10^9^ m^3^/y), with about 65% coming from the Lavaca River, and it hasn’t declined since construction of Lake Texana (Montagna et al., 2020). The LCE is connected to the Gulf of Mexico with tidal exchange occurring through Pass Cavallo and the Matagorda Bay Ship Channel. The bay is mostly muddy with some fringing marsh and seagrass beds. The eastern oyster (*Crassostrea virginica*) and black drum (*Pogonias cromis*) appear to be increasing over time, while blue crab (*Callinectes sapidus*), southern flounder (*Paralichthys lethostigma*), and all benthic metrics (abundance, biomass, and diversity) are decreasing over time (Montagna et al., 2020).

### Sampling

**Fig. 1:**
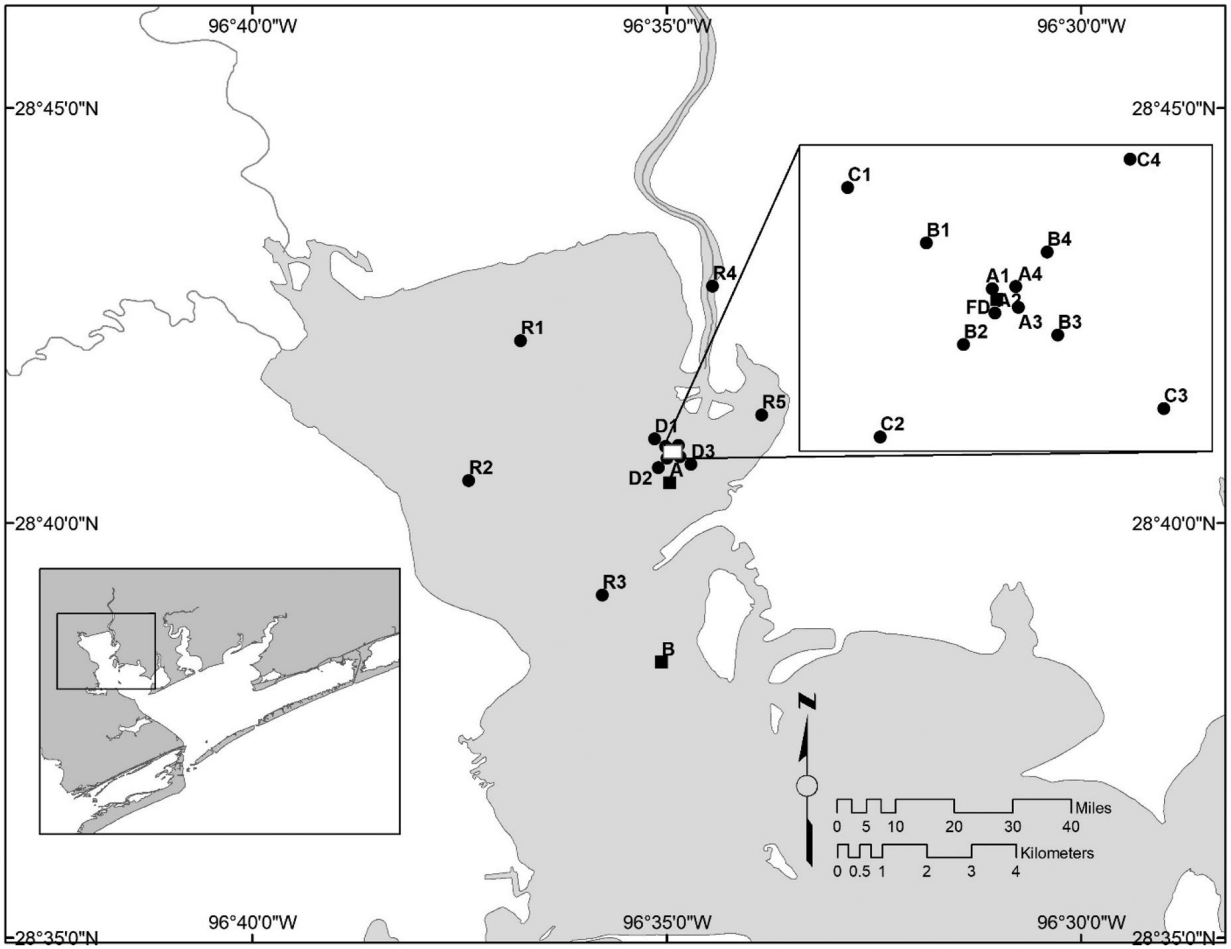
Sampling stations in Lavaca Bay, Texas.  Stations A, B, and FD are HRI stations. Measurement Variables

Fixed-point sampling was collected at 19 stations (Fig. 1). Stations were set by general circulation patterns in Lavaca Bay, zone of initial dilution, and mixing zone descriptions. Distances A, B, C, D, and R form rings around the diffuser with a total of 4 replicate stations (1, 2, 3, 4) per ring, except for D-stations which only have 3. One sample was taken at each station during each sampling trip except for plankton samples. Four replicate plankton samples were collected at each of the R stations, and one plankton sample was collected at each of the B stations. Plankton samples were not collected at the A, C, or D stations. The discharge is near long-term sampling stations A, B, and FD by the Harte Research Institute (HRI): station 85 (Kalke & Montagna, 1991) was relabeled station A (Montagna & Kalke, 1995). Exact locations are provided in Table S2.

The stations were sampled for 27 years (1993–2020) on a quarterly basis; except, Year 1 has 7 trips, 3 as baseline collections, and 4 as post-discharge collections, and Year 2 has 5 trips. A list of all sampling trips by year, trip, and date is provided in Table S3.

### Measurement variables

The dataset is large, containing 672,574 rows (each with a value) and 24 columns (2 for the value and qualifier and 22 describing the sample location and type), and is publicly available (Montagna et al., 2022). Variable types include conventional chemistry (e.g., salinity, temperature, dissolved oxygen (DO), pH, and turbidity) and contaminant chemistry including trace metals, semi-volatile organic compounds (SVOC, e.g., hydrocarbons, aldehydes, ethers, esters, phenols, organic acids, ketones, amines, amides, nitroaromatics, polychlorinated biphenyls (PCB), also known as Aroclors, polycyclic aromatic hydrocarbons (PAH), phthalate esters, nitrosamines, haloethers and trihalomethanes), and volatile organic compounds (VOC, e.g., halogenated hydrocarbons, aromatics, ketones, nitriles, acrylates, acetates, ethers, and sulfides). Chemistry matrices include water, sediment, porewater, and tissues. Biological variables (benthic macrofauna, ichthyoplankton, phytoplankton, zooplankton, trawl, and gill net) were measured to study biological responses to environmental stressor effects on the ecosystem health. Typically, 10% of chemistry samples were duplicated for quality assurance purposes.

### Field and laboratory methods

The methods reported here summarize Freese and Nichols, Inc. (FNI, 2020), collection and analytical methods for abiotic and biotic measurements. Water chemistry samples were collected with a non-contaminating submersible plastic pump lowered and raised through the water column to collect vertically mixed samples (except those for metals analysis). Dissolved metals samples, except for mercury (Hg) and selenium, were field filtered through a 0.45-μm filter cartridge. Water samples were stored in containers of ice until transported to a chemistry subcontractor. As a minimum, 10% of sample stations per trip were triplicated for chemistry collections in water.

Porewater chemistry and sediment samples were collected with a stainless-steel posthole digger. Porewater was stored in 3.5-gallon plastic buckets and sealed to exclude air. Porewater was extracted with 5.0-μm polyester filters and chilled to ≤ 4 °C and treated with the proper preservative (if needed). Sediment samples were placed into airtight glass jars and stored at 2–4 °C.

Priority pollutants include total and dissolved metals, VOCs, and pesticides and PCBs. The method detection limit (MDL) is defined as the minimum concentration of a substance that can be measured and reported with 99% confidence that the analyte concentration is greater than zero and is determined from analysis of a sample in each matrix containing the analyte. FNI based quality assurance protocols on several USEPA (2019) analytical method standards in the efforts to measure the extent of environmental contamination in Lavaca Bay. All parameters, methods, and MDLs are found in Table S4.

Nekton/epifauna were collected with a 3-m otter trawl with a 6-mm mesh cod end and 45.7-m long by 2.4-m wide gill nets with six equal-sized mesh panels of 5, 7.6, 10, 12.7, 15, and 17.8 cm. Trawls were towed for 5 min at approximately 3 km/h, and distance was determined with a flow meter. Each trawl sampled approximately 280–300 m by 3-m width. Gill nets were set overnight and collected the subsequent morning.

Ichthyoplankton were captured with a 46-cm diameter, 7:1 length-to-mouth ratio tow net with throat-mounted flow meter, and #505 mesh. Ichthyoplankton tows were made at all stations sampled by trawl and gill nets and towed obliquely over a 5-min period. Ichthyoplankton samples were preserved in 10% formalin and transferred to 70% ethanol, plus glycerin after hardening.

Phytoplankton were collected with a non-contaminating submersible pump. Two L were collected from the surface to a depth of 1 m and preserved within a 1% Lugols solution. A 2.2-mL subsample was placed in a sedimentation chamber for analysis with an inverted microscope. The sample was set to settle for approximately 4 h. Phytoplankton collected from years 1 to 15, minus year 5, were analyzed under 400 × magnification. Years 16–27 were analyzed at a magnification of 1000 × . Phytoplankton and small zooplankton were identified to the lowest possible taxonomic unit.

Zooplankton were collected with a #20-(80-μm) mesh plankton net. Forty-eight L of water from the surface were measured, and samples were preserved in 1% Lugols solution. A 1-mL subsample was put into a 1-mL Sedgwick-Rafter cell for analysis with an inverted microscope. All zooplankton and large phytoplankton were identified and counted at 100 × magnification. Zooplankton and large phytoplankton were identified to the lowest taxonomic unit possible.

Benthos were collected using a 6-by-6-inch Ekman dredge (0.023 m^2^), washed in the field with a #30-mesh screen, and then preserved in 10% formalin. Organisms were picked from the sample and then stored in 70% ethanol, plus glycerin. A dissecting microscope was used to pick out all invertebrates from a small aliquot of the sample placed in a pan with water.

### United States Geological Survey (USGS) flow data

A daily-average time series of historical river flows from 1968 to 2020 was downloaded from the USGS gauge 08164000 on the Lavaca River near Edna, TX, https://waterdata.usgs.gov/tx/nwis/dv?site_no=08164000. The time series was used to classify climatic intervals as “wet,” “average,” and “dry” periods using quartile ranges of the cumulative flow data collected 30 days prior to each sampling trip.

### Statistical analyses

All data were first split by type and then averaged by lab duplicate for replicates for chemistry and then by trip-station combinations for both biotic and abiotic data. Trip and station represent independent variables in this dataset, and abiotic physical–chemical measurements and community measurements are the dependent variables. All data was manipulated and analyzed using SAS 16.1 software (Institute, S. A. S., & Inc, 2016, 2017a, b), and the biotic community data was analyzed using PRIMER (Clarke & Gorley, 2015).

All variables were tested for linear trends over time using regression and correlation analysis. Biological data were natural logarithm transformed prior to analysis. A two-way analysis of variance (ANOVA) could not be used because there were no replicates at stations. Instead, a one-way block ANOVA was used to test for differences with distance from the discharge where sampling trips were blocks and stations within rings were used as replicates. Linear contrasts were used to test for specific differences with distance from discharge (i.e., station rings).

Principal component analysis (PCA) is a multivariate technique used to represent similarities among variables within each group (Chiang et al., 2002). Before the PCA analysis, data was standardized to a normal distribution with a mean of zero and standard deviation of one so that scales were the same for all variables. Spearman correlation coefficients were calculated to identify the relationship between the new PCA variables for the hydrographical and sediment analyses with biological univariate metrics.

Percent of sand, silt, and clay was plotted in ternary diagrams following the Shepherd sediment classification scheme (Shepherd, 1954). Ternary plots were made with Python 3.9 standard libraries and python-ternary package (Harper et al., 2015). Data was imported as netcdf after conversion of Excel to netcdf or pandas data frame.

Differences in community structure of the biotic communities was analyzed with multivariate, nonmetric multidimensional scaling (nMDS), using PRIMER (2015) software. Species abundances were square-root transformed, and similarity between stations was calculated using the Bray–Curtis similarity index. The resulting similarity matrix was ordinated using nMDS. Differences in community structure among independent variables (trips and distances from discharge) were analyzed using analysis of similarity (ANOSIM).

Structural equation modeling (SEM) is a multivariate technique to identify links between the abiotic and biotic manifest (observed) or latent (unobserved) variables in multivariate space. Path analysis was used to identify branches from SEM to test hypothesized patterns of directional and non-directional relationships among a set of manifest variables (Hoyle, 1995). The SEM and path analyses were calculated using SAS PROC CALIS. All variables were first standardized to a normal distribution.

## Results

### USGS flow data

**Fig. 2:**
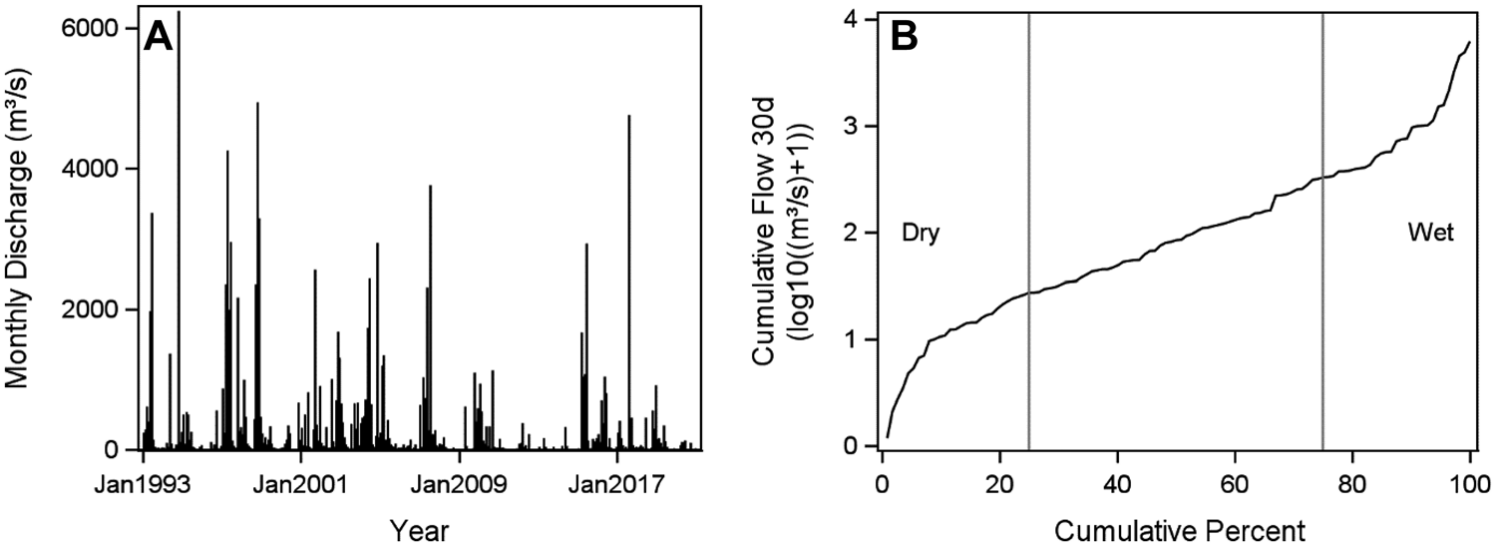
Cumulative discharge at USGS 8164000. **A** Cumulative monthly discharge (m^3^/s) over time. **B** Cumulative discharge 30 days prior to sampling (log10 m^3^/s) by cumulative percent rank.  Vertical lines represent 25th and 75th percentile

The cumulative monthly discharge pattern is cyclical with dry and wet periods (Fig. 2A). The 30-day cumulative gauged inflow prior to each sampling date was calculated. The 25th quartile range and below indicates a dry climatic period (Fig. 2B). Most inflow data collected during these periods was less than approximately 30 m^3^/s. In between the 25th and 75th quartile range indicates data collected during an average climatic period with inflow between 30 and 350 m^3^/s. Data above the 75th quartile range indicates the portion collected during a wet climatic period. Freshwater inflow rates were 351 to approximately 6300 m^3^/s during these collection periods. The log scale indicates inflow trends into Lavaca Bay is log-linear during the average climatic period. There were only 7 flood events over 1000 m^3^/s captured by the sampling events.

**Fig. 3:**
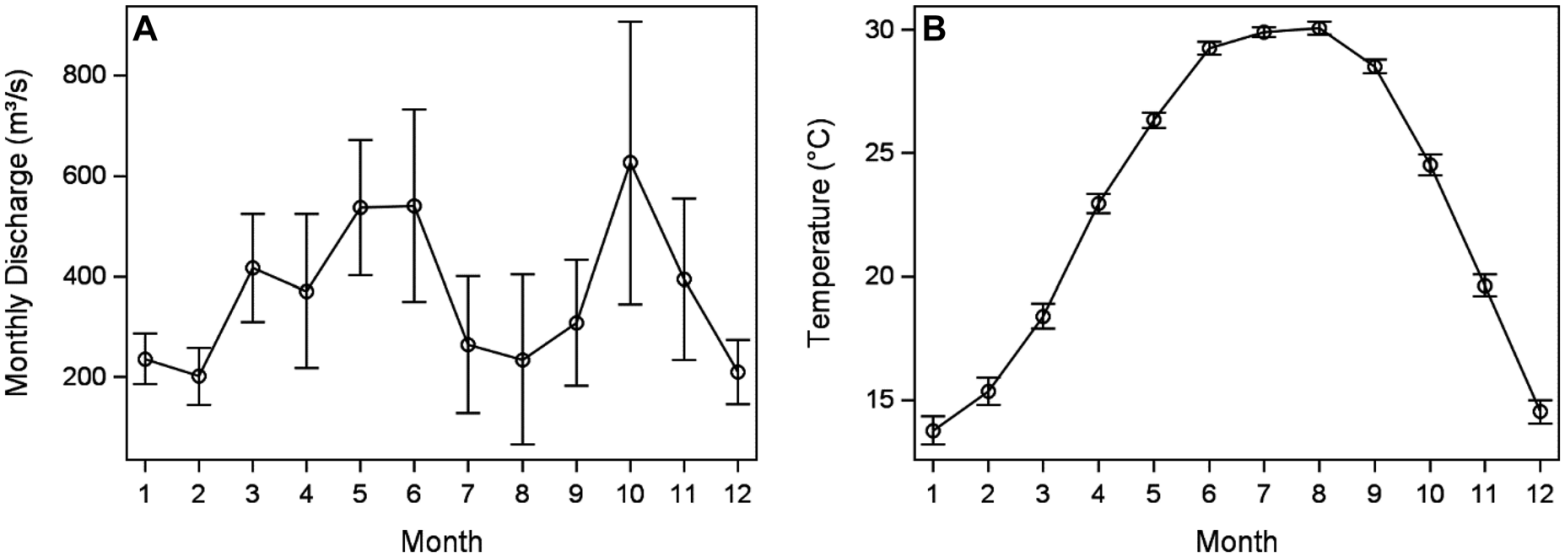
Seasonal dynamics. **A** Average cumulative monthly inflow from 1993 - 2020 from USGS 8164000. **B** Average water temperature from 1977 - 2020 from Texas Parks and Wildlife Department. Error bars are standard error

There is a seasonal inflow pattern in Lavaca Bay, and the weather is variable from year to year as indicated by the error bars in Fig. 3A. Freshwater inflow discharges in late spring-early summer (May–June) and fall (October) months are greater than freshwater inflow rates in winter and later summer months. Fall peaks are likely due to tropical storms. October has the largest error bars because storms are more variable than spring rain and runoff. While there is also a seasonal change in temperature typical of the northern hemisphere, the error bars are small (Fig. 3B).

### Non-detected vs. detected contaminants

The contaminant chemistry was nearly all below methods detection limits (Table 1). All organic contaminant groups (i.e., PAH, PCB, pesticide, volatile, and semi-volatile) were > 99% of samples non-detected. Therefore, no further analysis of the organic contaminants was attempted. There was more data for metals with ~ 62% of all metal samples non-detected. However, only sediment metals were detected in > 75% of samples and are used in further analysis. Physical–chemical groups had higher percentages of detected concentrations compared to the contaminant groups. Conventional, physical, and sediment groups had > 94% detected samples, and inorganic, organic, and oxygen demand had < 66% of samples detected. However, nitrogen nutrients were below the detection limits. Table S5 displays all sample variables with associated detected and non-detected counts.

**Table 1:** Summary table of detected vs. non-detected values for contaminant and chemical measurements in all media

**Type**	**Group**	**Number of samples**		**Percent of samples**	
		**Total**	**Non-detect**	**Non-detect**	**Detect**
Contaminant	Metal	75,371	46,909	62.24%	37.76%
	PAH	89,014	88,981	99.96%	0.04%
	PCB	4246	4239	99.84%	0.16%
	Pesticide	19,408	19,403	99.97%	0.03%
	Volatile	148,590	148,553	99.98%	0.02%
	Semi-volatile	163,413	163,061	99.78%	0.22%
Phys-chem	Conventional	33,134	1775	5.36%	94.64%
	Inorganic	38,083	13,020	34.19%	65.81%
	Organic	23,957	12,366	51.62%	48.38%
	Oxygen demand	8128	2838	34.92%	65.08%
	Physical	6624	1	0.02%	99.98%
	Sediment	6333	0	0.00%	100.00%

### Temporal trends

Plots for all the variables over time are in Fig. S1. Linear regressions and Spearman rank correlations were calculated to determine if there has been no change over time (Table 2). All sediment metals, including aluminum (Al), copper (Cu), lead (Pb), Hg, and zinc (Zn), decreased over time along with silt and clay (= mud) content, while sand content increased. Mercury concentrations began at ~ 0.06 mg/kg in 1993, increased to the largest concentration documented for mercury at ~ 0.65 mg/kg in 1994, and then gradually declined in concentration over time until a spike of 0.58 mg/kg in 2015. The Hg concentrations dropped back down in 2016 and remained low into 2020.

**Table 2:** Linear regression by date for chemical and biological variables from 1993 to 2020. Phytoplankton and zooplankton from 2009 to 2020. Abbreviations: *n* = sample size, *r* = correlation, *P* = probability level

**Matrix**	**Variable (abbreviation)**	**Regression**		**Spearman correlation**		
		**Intercept**	**Slope**	***n***	***r***	***P***
Water	Total phosphorous (TP)	151.15	−0.075	108	−0.18	0.0611
	Orthophosphate (PO_4_)	−0.47	0.0003	108	0.04	0.6652
	Silicate (SO_4_)	−52,776.86	26.93	108	0.28	0.0031
	Dissolved organic nitrogen (DON)	38.42	−0.019	108	−0.29	0.0022
	Total organic carbon (TOC)	956.31	−0.471	110	−0.46	0.0001
	Chemical oxygen demand (COD)	−8041.86	4.052	110	0.40	0.0001
	Dissolved oxygen (DO)	−55.72	0.0317	112	0.22	0.0219
	Salinity	−685.79	0.3495	112	0.31	0.0007
	Temperature	188.49	−0.0827	112	−0.11	0.2608
	Turbidity	1808.36	−0.8856	108	−0.27	0.0045
	pH	−0.59	0.0043	110	0.10	0.2902
Sediment	Aluminum (Al)	88,5867	−434.4	112	−0.43	0.0001
	Copper (Cu)	402.31	−0.1965	112	−0.66	0.0001
	Lead (Pb)	873.75	−0.4304	112	−0.73	0.0001
	Mercury (Hg)	15.57	−0.0077	112	−0.57	0.0001
	Zinc (Zn)	1470.80	−0.7192	112	−0.70	0.0001
	Clay	1423.84	−0.6970	112	−0.80	0.0001
	Sand	−1,549.68	0.8018	112	0.63	0.0001
	Silt	223.94	−0.1039	112	−0.23	0.0162
Biological	Phytoplankton richness (PP_R)	−2439.39	1.2371	36	0.63	0.0001
	Phytoplankton abundance (PP_n)	−185,159,087	92,774	36	0.57	0.0003
	Ichthyoplankton richness (IP_R)	−9.10	0.0056	108	0.02	0.8041
	Ichthyoplankton abundance (IP_n)	1795.05	−0.8785	108	−0.11	0.2694
	Zooplankton richness (ZP_R)	−719.83	0.3655	36	0.62	0.0001
	Zooplankton abundance (ZP_n)	−2,383,434,430	1,194,044	36	0.58	0.0002
	Benthic richness (BN_R)	−126.84	0.0673	112	0.13	0.1863
	Benthic abundance (BN_n)	−19,154.80	9.8939	112	0.10	0.2745
	Gill net richness (GN_R)	−84.20	0.0463	109	0.13	0.1665
	Gill net abundance (GN_n)	−2047.58	1.0645	109	0.15	0.1140
	Trawl richness (TR_R)	309.75	−0.1515	111	−0.55	0.0001
	Trawl abundance (TR_n)	54,778.34	−27.0639	111	−0.60	0.0001

There was a consistent linear trend for water quality measures over time indicating freshwater inflow has decreased. Salinity and chemical oxygen demand (COD) increased over time. Dissolved organic nitrogen (DON), total organic carbon (TOC), and turbidity decreased over time. That salinity increased and turbidity and solutes decreased is expected because inflows that dilute seawater and deliver nutrients and sediments from the watershed had decreased. Temperature, pH, and DO did not change over the 27-year period.

Phytoplankton and zooplankton abundances (n/m^3^) were both near zero from 1993 through 2009. After 2009, phytoplankton and zooplankton counts ranged from close to zero to 6,000,000 n/m^3^ and from approximately 20,000,000 to 100,000,000 n/m^3^, respectively. Phytoplankton and zooplankton abundances spanned seven and eight orders of magnitude, respectively. Phytoplankton exhibited three abundance patterns: lower abundances (< 1,000/m^3^) from 1993 to 1999, moderate abundances (1,000/m^3^ to 10,000/m^3^) from 2000 to 2008, and higher abundances (100,000/m^3^ to 1,000,000/m^3^) from 2009 to 2020. Zooplankton also had three abundance patterns: lowest from 1993 to 1995, then higher from 1996 to 2009, and highest from 2010 to 2020. Phytoplankton richness begins low with values under 40 species/2 L from 1993 to 1997, rises to 85 species in 1999, and then gradually decreases until 2006 before increasing through 2012. For zooplankton richness, the initial period from 1993 to 1995 has lower values under 10 species, and then, from 1996 onward, values look to be consistently greater than 15 species. Because phytoplankton and zooplankton dynamics appear to vary outside expected ranges in the early parts of the study, only the data from 2009 to 2020 will be used in further analyses. From 2009, there is no difference over time for zooplankton, but phytoplankton are increasing.

Ichthyoplankton abundance (n/5-min tow) spanned 5 orders of magnitude. The highest ichthyoplankton richness occurred in 2002, 2004, 2006, 2012, and 2020 with 6 to 8 species per sample.

Benthic abundance (n/m^2^) spanned three orders of magnitude. Abundances began high in the thousands from 1993 to 2001, dropped to single digits in 2002, fluctuated up and down between 2005 and 2010, dropped once more in 2011, and then leveled out from 2012 onward. Benthic richness ranged from 1 to 23 species per sample and is high when abundance is high and low when abundance is low.

Gill net abundance (n/24-h) spanned five orders of magnitude. Abundance values appear to plot mostly with data in 1000 s but decrease for years 1994, 1996 to 1997, 2000 to 2002, 2007 to 2015, 2018, and 2020. Gill net richness fluctuated up and down regularly every 2 years with 15 species as the highest species count and one as the lowest.

Trawl abundance (n/10-min tow) spanned five orders of magnitude and gradually decreased over time. Abundance was lowest during 2015. Trawl richness spanned 12 orders of magnitude. For richness, 12 species were counted in 1993, and the number gradually decreased to one species counted in 2015. Species counted increased to 6 in 2016 then varied from 6 to 22 species accounted for from then on.

### Spatial differences

A one-way block ANOVA was performed to test for differences in distance, i.e., rings A, B, C, and R, from the diffuser (Table 3), and stations within a ring were used as replicates (see Fig. 1). Water column variables had the least number of differences with distance from the discharge. Distance influences both species richness and abundance for benthic, gill net, and phytoplankton samples. Zooplankton abundance and trawl richness are not influenced by distance from the discharge site but zooplankton richness and trawl abundance are. Ichthyoplankton and trawl abundances and richness did not differ between distances B and C. Sediment grain size and trace metal parameters change over distance.

**Table 3:** Probability values for one-way block ANOVA to test for spatial differences (trip as a block) for water column and biological variables. Linear contrasts used to test for differences between discharge stations (A–C) and reference stations (R). Abbreviations: Matrix—Wat, water; PW, porewater; Sed, sediment; and Bio, Biological; variables, as in Table 2. Highlighted cells are *P* > 0.05

**Matrix**	**Variable**	**Effects**		**Distance Linear Contrasts**					
		**Trip**	**Distance**	**A_vs_B**	**A_vs_C**	**A_vs_R**	**B_vs_C**	**B_vs_R**	**C_vs_R**
Wat	TP	0.0001	0.0001	0.4429	0.1220	0.0001	0.4308	0.0003	0.0050
Wat	PO4	0.0001	0.0001	0.2022	0.0012	0.0001	0.0472	0.0007	0.1547
Wat	SO4	0.0001	0.5271	0.2016	0.4782	0.2004	0.5787	0.9756	0.5650
Wat	DON	0.0001	0.1389	0.5475	0.0958	0.6248	0.0238	0.2814	0.2504
Wat	TOC	0.0001	0.0001	0.0003	0.0001	0.0001	0.0748	0.0044	0.2799
Wat	COD	0.0001	0.0070	0.5676	0.1461	0.0095	0.0436	0.0016	0.2506
Wat	DO	0.0001	0.0001	0.0001	0.0001	0.0001	0.0006	0.0001	0.0001
Wat	Salinity	0.0001	0.0368	0.0170	0.0104	0.0401	0.8342	0.7748	0.6255
Wat	TDS	0.0001	0.0267	0.0038	0.0297	0.1615	0.4880	0.1515	0.4566
Wat	Temperature	0.0001	0.0001	0.0001	0.0001	0.0001	0.1712	0.0001	0.0001
Wat	Turbidity	0.0001	0.1991	0.0950	0.0450	0.1866	0.7222	0.7545	0.5100
Wat	pH	0.0001	0.0001	0.0001	0.0001	0.0010	0.0009	0.3242	0.0001
PW	Chloride	0.0001	0.0001	0.1658	0.0001	0.0001	0.0112	0.0001	0.0070
PW	TDS	0.0001	0.0001	0.0417	0.0018	0.0001	0.2727	0.0001	0.0001
PW	NH4	0.0001	0.0001	0.0001	0.0001	0.0001	0.0001	0.0001	0.2422
PW	TP	0.0001	0.0001	0.0001	0.0001	0.0001	0.0001	0.0001	0.0002
PW	PO4	0.0001	0.0001	0.0001	0.0001	0.0001	0.0001	0.0001	0.0002
PW	DON	0.0001	0.0001	0.0001	0.0001	0.0001	0.0001	0.0012	0.5476
PW	TOC	0.0001	0.0001	0.0001	0.0001	0.0001	0.0001	0.0001	0.0765
PW	COD	0.0001	0.0001	0.2330	0.0001	0.0001	0.0056	0.0001	0.0143
Sed	SO4	0.0001	0.0001	0.0001	0.0001	0.0001	0.0001	0.0025	0.0016
Sed	AVS	0.0001	0.0001	0.0001	0.0001	0.0040	0.0002	0.0676	0.0001
Sed	NH4	0.0001	0.0001	0.0001	0.0001	0.0001	0.0001	0.1220	0.0001
Sed	Al	0.0001	0.0001	0.0001	0.0001	0.0001	0.0001	0.0001	0.0001
Sed	Cr	0.0001	0.0001	0.0001	0.0001	0.0001	0.0001	0.0001	0.0001
Sed	Cu	0.0001	0.0001	0.0001	0.0001	0.0001	0.0001	0.0001	0.0001
Sed	Hg	0.0001	0.0001	0.0001	0.0001	0.0001	0.5209	0.0001	0.0001
Sed	DON	0.0001	0.0001	0.0001	0.0001	0.2357	0.0001	0.0052	0.0001
Sed	TOC	0.0001	0.0001	0.0001	0.0001	0.0002	0.0001	0.0001	0.0001
Sed	Volatile	0.0001	0.0001	0.0002	0.0001	0.0012	0.0001	0.0001	0.0001
Sed	Clay	0.0001	0.0001	0.0001	0.0001	0.0001	0.0001	0.0001	0.0001
Sed	Sand	0.0001	0.0001	0.0001	0.0001	0.0001	0.0001	0.0001	0.0001
Sed	Silt	0.0001	0.0001	0.0001	0.0001	0.0001	0.0001	0.0001	0.0001
Bio	BN_R	0.0001	0.0001	0.0001	0.0001	0.0001	0.0001	0.0001	0.0001
Bio	BN_n	0.0001	0.0001	0.0001	0.0001	0.0178	0.0001	0.0001	0.0001
Bio	GN_R	0.0001	0.0050				0.5670	0.0122	0.0021
Bio	GN_n	0.0001	0.0001				0.0001	0.0001	0.0009
Bio	TR_R	0.0001	0.5365				0.4399	0.2793	0.7512
Bio	TR_n	0.0001	0.0001				0.1569	0.0001	0.0001
Bio	IP_R	0.0001	0.0018				0.1813	0.0004	0.0282
Bio	IP_n	0.0001	0.0079				0.4040	0.0025	0.0286
Bio	ZP_R	0.0001	0.0001					0.0001	
Bio	ZP_n	0.0001	0.2195					0.2195	
Bio	PP_R	0.0001	0.0001					0.0001	
Bio	PP_n	0.0001	0.0001					0.0001	

### Hydrography analysis

**Fig. 4:**
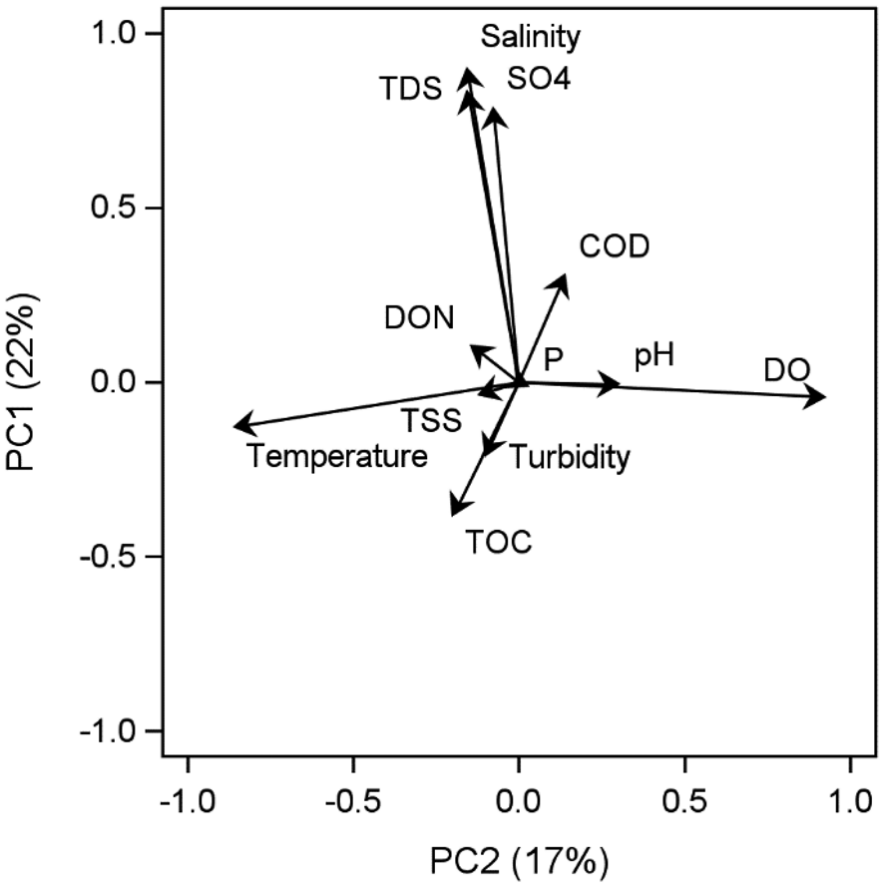
PCA vector loads for hydrographic variables for trip and station samples. Abbreviations as in Table 2

There were 1673 samples with complete water chemistry data from the 112 trips and 16 stations. The principal component (PC) loads for the first axis (PC1) and second axis (PC2) for hydrographic variables across trips and stations explained 22% and 17% (total 39%) of the variation among all hydrographic data, respectively (Fig. 4). The PC1 loads for the hydrographic data had the highest positive values for sulfate (SO4), total dissolved solids (TDS), and salinity and low negative values for TOC and turbidity. Thus, PC1 represents freshwater inflow effects because turbidity is low when salinity is high. The PC2 axis had values for temperature inversely correlated with values for DO and pH. The PC2 axis represents a seasonal effect because it is well known that the solubility of oxygen increases with decreasing temperatures and it is cooler in winter than summer.

**Fig. 5:**
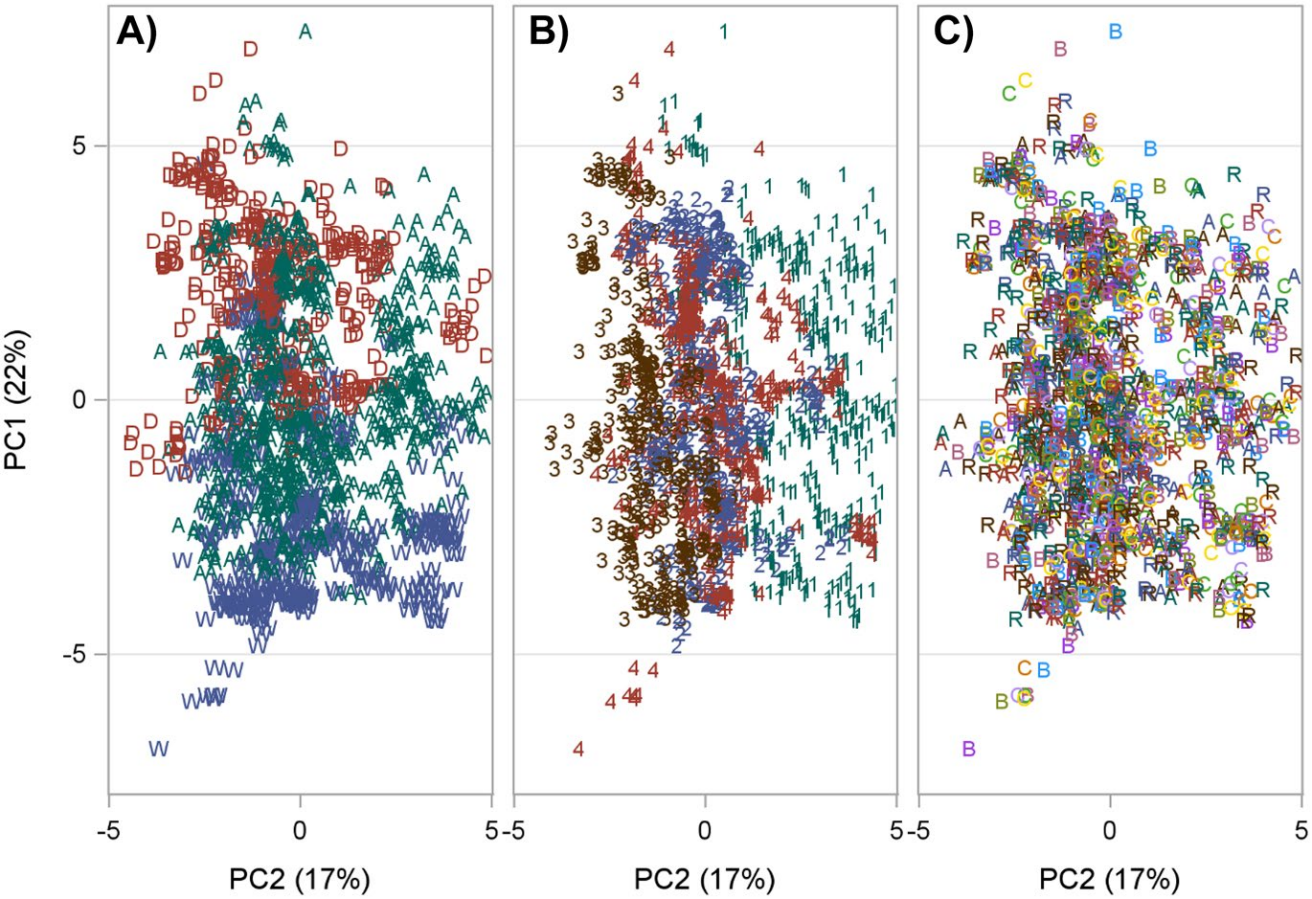
PCA sample scores for the hydrographic variables. **A** Climatic periods where A=average, D=dry, and W=wet. **B** Seasons where 1=winter, 2=spring, 3=summer, and 4=fall. **C** Distance and direction from discharge where A=15 m, B=61 m, C=183 m, R=3810 m and transect

The hydrographical trip-station scores exhibited no relationships with stations for either PC1 or PC2; however, trip-station scores had a distinct climatic and seasonal distribution pattern (Fig. 5). Negative PC1 scores representing low salinity and high TOC were associated with wet climatic periods (W), while positive scores with high salinity and low TOC were associated with average (A) and dry (D) climatic periods (Fig. 5A). The PC2 scores show an inverse relationship between DO and temperature representing seasonality as winter (1) samples plot positively, spring (2) and fall (4) seasons plot more neutrally with PC2 scores that are slightly negative but closer to zero, and summer (3) values plot negatively and furthest to the left (Fig. 5B).

### Sediment analyses

An initial PCA was performed using all detectable metals in 2054 samples, but all metal variables plotted at a 45° angle with no PC1 or PC2 relationships, so it is not presented here. The lack of a PC relationship indicates all metal variable values maintained similar relationships to one another over space and time. A subset of the most toxic metals (i.e., Cr, Cu, and Hg) that also had the highest variable loads are used in further sediment analyses.

All the sediment and porewater variables were different over sampling trips and distance from the discharge (Table 3). In sediment, ammonium (NH_4_) and acid volatile sulfides (AVS) show some similarities between stations B and R. In porewater, chloride (Cl) and COD were the same nearest the discharge (i.e., A vs B) but different with increasing distance from the discharge, while NH*4*, DON, and TOC were different nearest the discharge but similar in increasing distance from the discharge (i.e., C vs R).

**Fig. 6:**
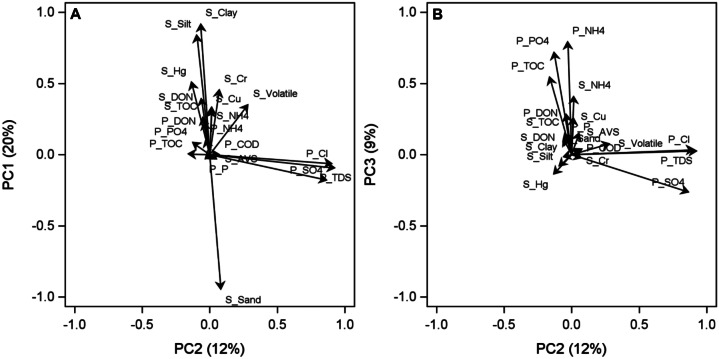
Sediment PC variable loads. Prefix: P = porewater, S = Sediment

A PCA was performed on 1668 samples from 112 trips and 16 stations for sediment porewater, grain size, and a subset of metals identified in the previous section (Fig. 6). The PC1 axis explained 20% of the variability in the dataset and shows an inverse relationship between silt, clay, HG, and chromium (Cr) in sediment (positive values) and sand (negative values) (Fig. 6A). The PC2 axis accounted for 12% of the variability, and positive values are associated with porewater salinity measures such as Cl, TDS, and SO4 (Fig. 6B).

**Fig. 7:**
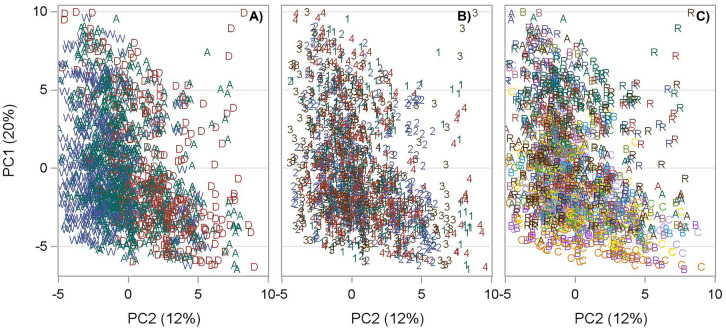
PC sample scores for the sediment variables. **A** Climatic periods where A=average, D=dry, and W=wet. **B** Seasons where 1=winter, 2=spring, 3=summer, and 4=fall. **C** Distance and direction from discharge where A=15 m, B=61 m, C=183 m, R=3810 m

Trip-station sample scores for the sediment PCA exhibit differences among stations by distance and direction (i.e., transects) along the PC1 axis and differences according to climatic patterns for PC2 (Fig. 7). The wet periods (W) cluster to the left with negative PC2 scores, and the dry periods (D) cluster to the right with positive scores that correspond to low salinity-indicator loads (Fig. 7A). Sample scores for PC1 and PC2 show no clear relationship with seasonality (Fig. 7B). Generally, the A, B, and C stations nearest the discharge site are mostly negative, and values become positive with increased distance from the discharge as R stations are the most positive values. The PCA results indicate that distance from the discharge (PC1) influences sediment grain size distribution but hydroclimatic conditions (PC2) do not. Furthermore, direction (i.e., transect) adds to the explanation of sediment variation. These trends with PC1 indicate muddier sediments with more contaminants (Fig. 7A) are more common further from the discharge site and the discharge site is sandier.

**Fig. 8:**
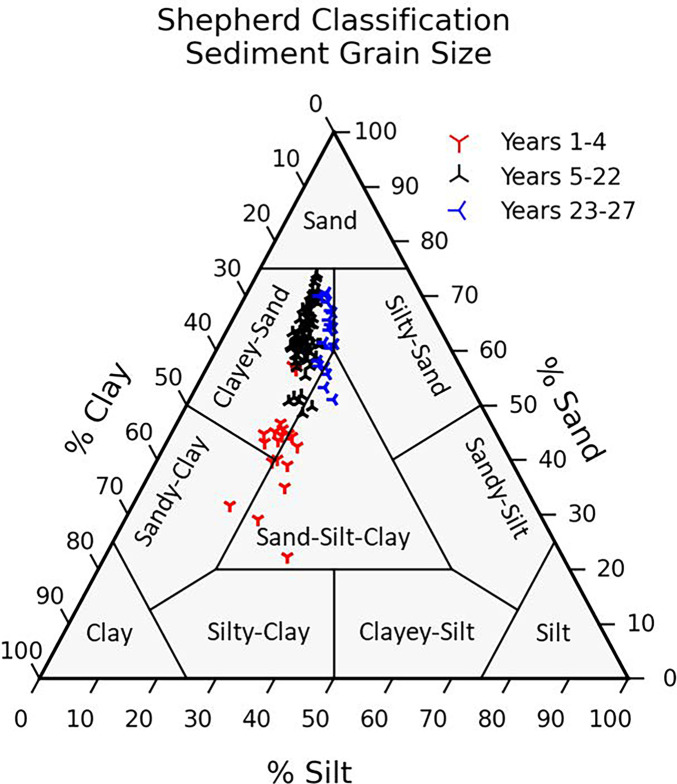
Sediment grain ternary plot using the Shepherd classification to categorize sediment grain size overtime

Sediment grain size distribution was not consistent over the study period. The average grain size distribution shifted 4 years into data collection (Fig. 8). Grain size characteristics began shifting in 1997 from a grain size distribution with clay content between 35 and 55% to a mixture of higher sand (48 to 75%) and lower clay (17 to 38%) composition until 2015. After 2015, there was a slight increase in silt content.

### Biological analyses

**Fig. 9:**
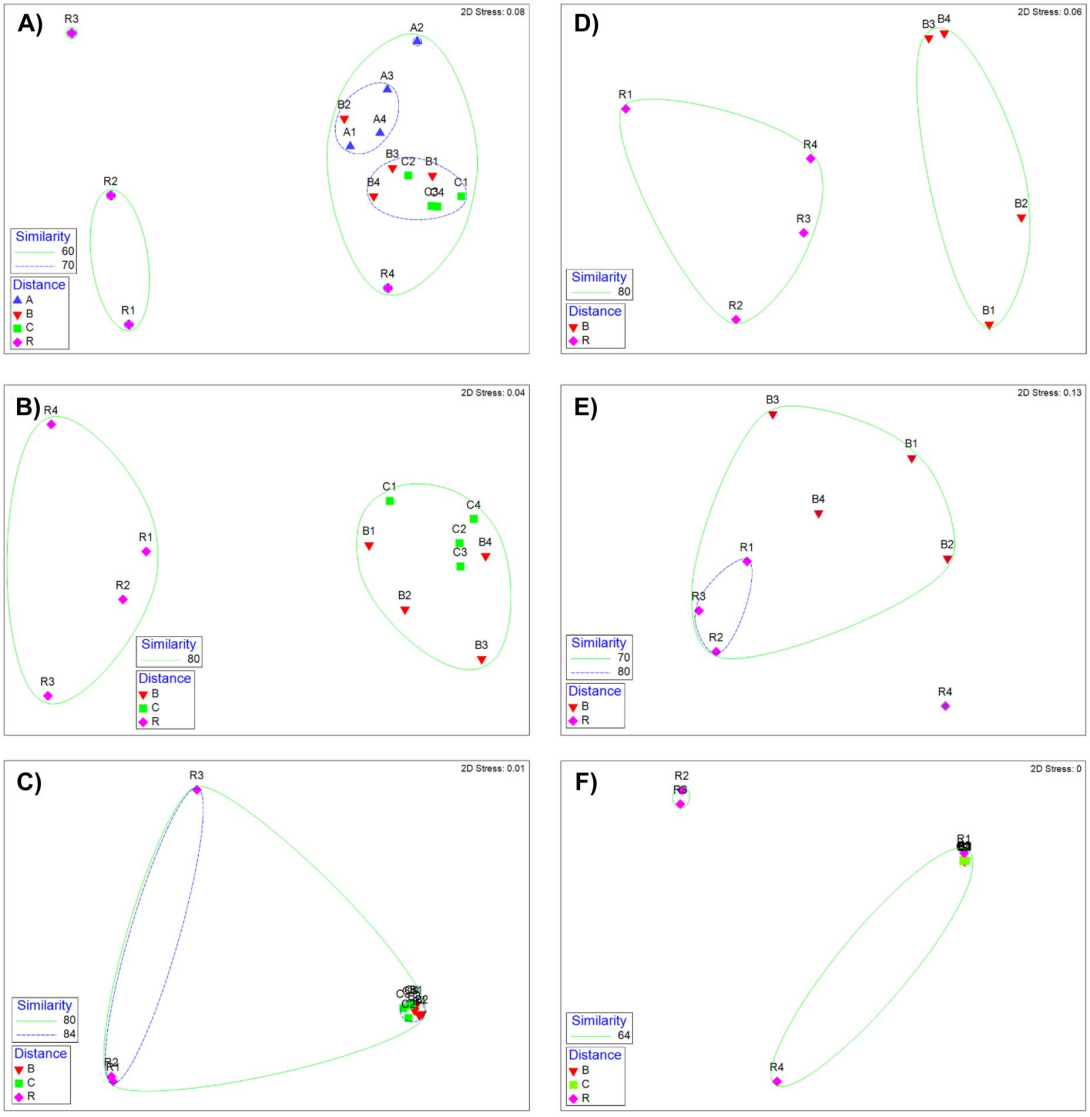
Multivariate analysis of community structure for taxa based on average abundance over time. **A** Benthos from cores. **B** Nekton from trawls. **C** Nekton from gill nets. **D** Phytoplankton from pumped water. **E** Zooplankton from tows. **F** Ichthyoplankton from tows

Community structure was analyzed using nMDS (Fig. 9). An initial nMDS was run on the two-way design of sampling period and stations, but there were changes over time (ANOSIM, *P* < 0.001) for all biological groups. Because the focus here is on station differences, species abundances were averaged over all periods by station to allow examination of the data by distance from the discharge where A = 15 m, B = 61 m, C = 183 m, and R = 3810 m. All *P* values reported in this section are based on rho (*R*) values calculated in the ANOSIM procedure. The species lists for all biota are found in Table S6.

Benthic community structure changed over distance from the discharge (*R* = 0.445, *P* ≤ 0.001, Fig. 9A). Stations near the discharge were generally similar and different from R stations except for station R4, which clustered with discharge stations. Benthic communities were similar in A and B stations and B and C stations (*P* = 0.114), but A and C stations were different (*P* = 0.029). A total of 471 benthic species were found over the sampling period, but the 22 most dominant species made up 75% of all individuals found. Only three species made up more than 5% of the species found: *Mulinia lateralis* = 23.8%, *Mediomastus ambiseta* = 10.7%, and *Rangia cuneata* = 5%.

Nekton community structure based on trawl samples was different with distance from the discharge (*R* = 0.609, *P* = 0.003, Fig. 9B). The R stations were different from the C and B stations (*P* = 0.029), but the C and B stations were similar (*P* = 0.229). A total of 129 species were found in the trawl samples over the sampling period. Three species, *Anchoa mitchilli* (60.2%), *Brevoortia patronus* (17.8%), and *Micropogonias undulatus* (10.5%), accounted for 88.5% of all individuals found.

Similar to the nekton trawl data, nekton caught using gill nets showed differences with distance from the discharge (*R* = 0.447, *P* = 0.001, Fig. 9C). Station R4 was different from all the other stations, sharing only 75% similarity compared to 82% among all other stations, so a subset nMDS was created (Fig. 9C). In the secondary nMDS, there is a tight cluster for all the B and C stations, which were similar (*P* = 0.057). There were 89 species found in gill net samples, but three species, *Ariopsis felis* (37.7%), *Brevoortia patronus* (23.4%), and *Bagre marinus* (14.1%), accounted for 75.2% of all individuals found.

Phytoplankton community structure was different between stations near the discharge (B) and stations furthest from the discharge (R stations) (*R* = 0.656, *P* = 0.029, Fig. 9D). A total of 300 species or taxa were found in the net samples over the sampling period with two taxa, Synechocystis (62.3%) and Cyanobacteria (30.6%), comprising 92.8% of all individuals found. A third taxa, Synechococcus, made up 3.0% of individuals (for a total of 95.9% of individuals), but all other taxa consisted less than 0.8% of individuals.

In contrast to phytoplankton, zooplankton community structure near the discharge (B) and furthest from the discharge (R) was similar (*R* = 0.375, *P* = 0.057), although the stations separated from one another at the 80% similarity level (Fig. 9E). There was a total of 153 taxa, but only two taxa, protozoans (45.5%) and ciliated protozoans (28.8%), comprised 74.3% of all individuals found. Four other taxa accounted for at least 2.0% of individuals (for a total of 94.1% of individuals): *Myrionecta rubra* (8.3%), Amoebacea (6.5%), *Eutintinnus tubulosa* (3.0%), and *Tintinnopsis parvula* (1.9%). All other taxa consisted less than 1% of individuals.

Ichthyoplankton community structure was different with distance from the discharge (*R* = 0.257, *P* = 0.011, Fig. 9F). The R stations were different from the B and C stations (*P* = 0.029). A total of 83 species or taxa were found in the net samples over the sampling period. Ichthyoplankton were more evenly distributed than other biotic groups, and five taxa accounted for 76.4% of the individuals found: *Anchoa mitchilli* (30.1%), Clupeidae (16.9%), *Brevoortia patronus* (12.6%), *Gobiosoma bosci* (11.4%), and Engraulidae (5.3%). Four taxa comprised between 2 and 3% of individuals, and six taxa were made up between 1 and 2% of taxa. So, altogether, these 15 taxa were 96.2% of all individuals found.

### Linking biotic response to abiotic drivers

The biological diversity and abundance responses were correlated with the hydrological PCA variables to determine if biology was responding to water column changes. The PC1 loads represented freshwater inflow for the hydrographical PCA analysis across trips and stations (Fig. 4). For all samples collected during and after 2009, freshwater inflows (i.e., PC1) negatively influenced phytoplankton diversity (*r* =  −0.72, *P* =  < 0.0001) and abundance (*r* =  −0.57, *P* = 0.0003), zooplankton diversity (*r* =  −0.59, *P* = 0.0002) and abundance (*r* =  −0.51, *P* = 0.0016), trawl diversity (*r* =  −0.21, *P* =  < 0.0001) and abundance (*r* =  −0.13, *P* =  < 0.0001), gill net abundance (*r* =  −0.06, *P* = 0.0355), and ichthyoplankton abundance (*r* =  −0.10, *P* = 0.0005). Freshwater inflows positively influenced benthic diversity (*r* = 0.38, *P* =  < 0.0001) and benthic abundance (*r* = 0.15, *P* =  < 0.0001). The PC2 loads represented seasons for the hydrographical PCA analysis across trips and stations (Fig. 4). Seasonality (PC2) influenced gill net diversity (*r* =  −0.56, *P* =  < 0.0001), gill net abundance (*r* =  −0.38, *P* =  < 0.0001), ichthyoplankton abundance (*r* =  −0.06, *P* = 0.0377), and trawl diversity (*r* =  −0.26, *P* =  < 0.0001) negatively.

The relationship linking abiotic and biotic variables with the potential latent variables of inflow and seasonal dynamics of rivers was examined using SEM and path analysis. Inflow differences are proxies for year-to-year variability in climatic influences. Models were created for water column dynamics and sediment dynamics.

**Fig. 10:**
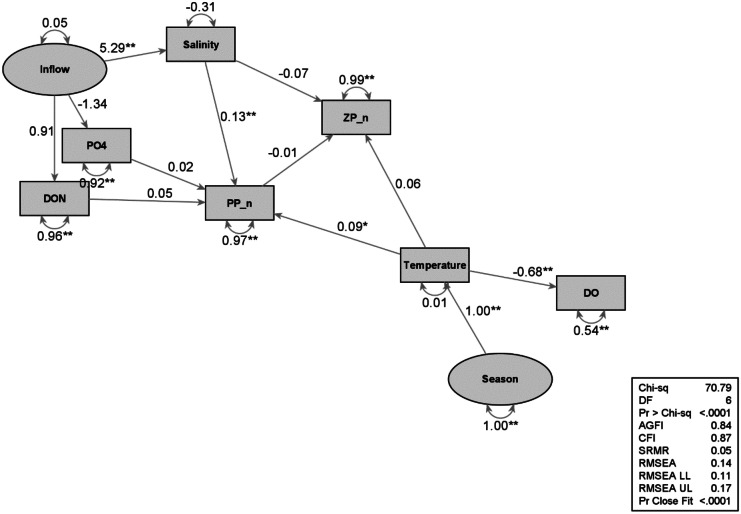
Water column path model. Abbreviations: PP_n = phytoplankton abundance, ZP_n = zooplankton abundance, ** = significant. Ovals = latent variables, rectangles = measurements

The water column model structure is based on the hypothesis that inflow dynamics drive nutrients, nutrients drive phytoplankton, phytoplankton drive zooplankton, and seasonal dynamics drive temperature (Fig. 10). Turbidity, as indicated by TSS, could decrease light availability and affect phytoplankton, but it had a zero effect so was dropped in the final model. Inflow drives salinity, and salinity was more important controlling phytoplankton than zooplankton. Inflow also drives nutrients, and DON was more important than phosphate (PO_4_) in driving phytoplankton. The link between phytoplankton and zooplankton was extremely weak (−0.01) and could have been dropped from the model. Temperature drives DO concentrations, but did not affect phytoplankton or zooplankton, likely due to samples being collected only in October, so those links were dropped from the model.

**Fig. 11:**
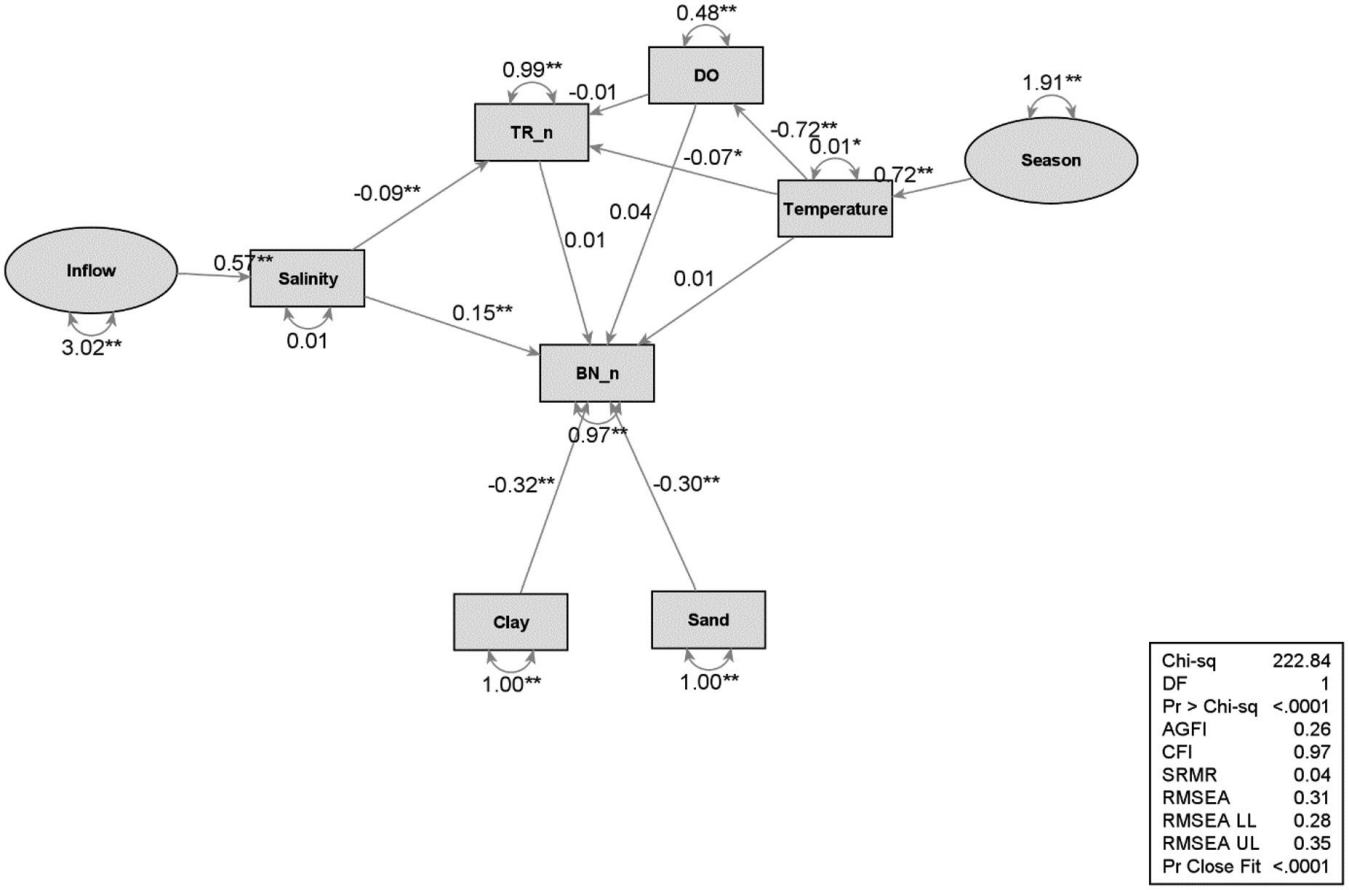
Benthic path model. Abbreviations: BN_n = benthic infauna abundance, TR_n = nekton abundance, ** = significant. Ovals = latent variables, rectangles = measurements

The benthic model structure is based on the hypotheses that (1) infauna are affected by salinity, DO, and sediments, (2) seasonal recruitment is likely, and (3) nekton, represented by trawl abundance, could prey upon infauna (Fig. 11). Inflow drives salinity, which has opposing effects on benthos and nekton. Nekton prefer decreasing salinity (i.e., high inflow), but benthos prefer increasing salinity (i.e., low inflow). The effect is stronger on benthos. Sediment structure has a stronger effect on benthos than salinity. Seasonality, which drives temperature and DO, does not influence benthos, but it does on nekton.

## Discussion

Previous environmental studies of Lavaca Bay focused on the distribution of Hg because it is an EPA Superfund site (Bissett et al., 2008; Bloom et al., 1999; Carr et al., 2001). The objective of the current study was to determine the relationships between natural stressors (e.g., differences in freshwater inflow regimes driven by climatic variability) and anthropogenic stressors (e.g., pollution caused by an industrial discharge) on biological communities and nonliving components in Lavaca Bay based on interdisciplinary, long-term, monitoring data. Previous, unpublished reports indicated the outfall had zero adverse effects on the ecological health and/or biological community structure dynamics in Lavaca Bay (FNI, 2020, p. 16). In contrast, the Carr et al. (2001) study observed elevated concentrations of contaminants and toxicity at several stations in Lavaca Bay. The different conclusions are explained by differences in sampling sites and choice of method detection limits.

For instance, the collection methods used to quantify PAHs in the current study, USEPA 8270 (USEPA, 1995), have detection limits of 150 μg/kg, whereas Carr et al. (2001) used the detection limit for PAHs of 0.5 μg/kg (Brooks et al., 1989; Wade et al., 1988). This is a 300-time difference. These detection limit differences were true for all organic contaminants. Carr et al. (2001) found total PAHs in seven of 24 stations exceeded either the probable effects level (PEL) or effects range median (ERM) (listed in Long et al., 1995; Macdonald et al., 1996) including a station adjacent to the ALCOA facility, which is about 5 km south of the FPC discharge site. The sample from the FPC discharge site, station 18 (near A1–A4, Fig. 1), had concentrations of total PCB 3.87 µg/kg, total DDT 0.47 µg/kg, and total PAH 65.9 µg/kg. Carr et al. (2001) stated that the “most toxic station overall in this survey was station 18 at the Formosa Plastics Co. outfall. It is apparently receiving contaminates from a different source [other than the Hg contamination from the ALCOA plant].” However, because of the high detection limits, about 99% of samples in the current study have non-detected contaminant quantities, and detectable concentrations of metals were observed in 37.76% of samples (Table 1). In contrast, Carr et al. (2001) found trace metals present with Hg, which was the only metal that exceeded both the PEL and the ERM. Without contaminant measurements at detectable concentrations, pollution cannot be assessed in the current study.

Although 99.9% of concentrations of organic chemicals collected for within sediment samples were non-detectable, the possibility for chemical pollution is still present. The MDL 8270 method for all organic chemicals is at or above 150 μg/kg for sediments. This method will not detect ambient chemical concentrations in Lavaca Bay. The National Oceanic and Atmospheric Administration effects range low (NOAA ERL) is 2- to tenfold lower than the detection limits in the current study for many contaminants (Burton, 2002). Thus, all non-detectable organic contaminants may still be posing a threat to the ecosystem because concentrations may be higher than the NOAA ERL (Table 4). Furthermore, even if chemical concentrations were below NOAA ERL, there is a possibility of synergistic effects of exposure to low concentrations of contaminants over time. The opposite may be said of trace metal detection limits. The EPA 200.7 method for sediment metals has detection limits well below NOAA ERL standards.

**Table 4:** Chemical contaminants where detection limits are above sediment quality standards

**Contaminant**	**NOAA ERL**	**Current MDL—METHOD**
Acenaphthene	16 ug/kg	150 μg/kg—8270 METHOD
Acenaphthylene	44 ug/kg	150 μg/kg—8270 METHOD
Anthracene	85.3 ug/kg	150 μg/kg—8270 METHOD
Fluorene	19 ug/kg	150 μg/kg—8270 METHOD
Naphthalene	160 ug/kg	150 μg/kg—8270 METHOD

High detection limits occur for other water quality variables. Approximately half of the nutrient data was non-detectable (Table 1). The SM 4500 method is a wastewater discharge method used to measure nutrient concentrations in the water column (Standard Methods Committee of the American Public Health Association, American Water Works Association, and Water Environment Federation, 2018; USEPA, 2019). The HRI has collected ambient water quality data for approximately 30 years (Kim & Montagna, 2009; Palmer & Montagna, 2015; Paudel et al., 2015, 2017; Pollack et al., 2009, 2011) (stations A, B, and FD in Fig. 1). The detection limits for nutrient data in the present and HRI studies differ. The current study uses SM 4500 with nutrient detection limits of 100 ug/L (i.e., *N* = 1.4 μM). In contrast, the HRI detection limits are 0.01 μM for nitrite + nitrate, 0.03 μM for ammonium, 0.01 μM for orthophosphate, and 0.07 μM for silicate (Montagna et al., 2018; Paudel et al., 2015, 2017). Thus, the HRI detection limits are 238 times more sensitive for ammonium and 714 times more sensitive for nitrite + nitrate compared to SM 4500 method. The HRI long-term average nutrient concentrations in Lavaca Bay were below the current study’s detection limits for NH_4_ (2.33 ± 5.23 μM) and NO_2_ (0.65 ± 0.73 μM), but not NO_3_ (4.73 ± 9.70 μM). Without water column nutrient and sediment contaminant data, the remaining discussion cannot include multi-stressor synergistic and antagonistic interactions and their influence on biological communities.

### Water column dynamics

Temporal dynamics of the water column indicate that salinity, DO, and COD increased over the course of the 27-year study, while DON decreased over time (Table 2). An inverse relationship between salinity and nutrients in Lavaca Bay has been observed in several past studies (Montagna et al., 2018; Palmer et al., 2011; Pollack et al., 2009; Shank et al., 2009). Estuaries are strongly influenced by the quantity, timing, frequency, and duration of freshwater pulses to coastal ecosystems (Montagna et al., 2013). As freshwater is introduced into Lavaca Bay from the Lavaca and Navidad River Basins, nutrients are introduced into the water body. Nutrient loading from local and upstream runoff as well as natural decomposition can lead to an increase in nutrients in the bay. With lower monthly freshwater inflow discharge rates, less nutrients have been introduced into the system.

Climatic wet and dry periods drive freshwater inflow effects (Douglas et al., 2021; Montagna, 2021; Montagna et al., 2013; Patrick et al., 2022; Pollack et al., 2011). Two hydrographical relationships were identified in Lavaca Bay by use of PCAs: freshwater inflow index (PC1) and seasonality (PC2) (Fig. 4). The freshwater inflow index shows dry climatic periods corresponded to high salinity and turbidity and wet climatic periods corresponded to low salinity and high TOC concentrations (Fig. 5), which likely loaded from the watershed. The seasonal trends show summer corresponds to high temperatures and low DO and winter corresponds to low temperatures and high DO with spring and fall falling in between.

Water column variable concentrations fluctuate among sampling trips (Table 3). However, total phosphorus and orthophosphate exhibit no substantial differences over time (Table 2), though seasonal variation of phosphorus concentrations does exist on a regional scale along the Texas coast (Kim et al., 2014; Montagna et al., 2018; Wetz et al., 2017). Freshwater inflow to estuaries from rivers and streams delivers nutrients, facilitates sedimentation, and dilutes seawater from the coastal ocean. The mixing of freshwater inflow and marine water occurs both spatially and temporally from climatic influences including tidal action, seasonal variation, and weather events (Pollack et al., 2011); thus, environmental flow is a driver of estuarine conditions and ecological responses to the varying estuarine conditions (Alber, 2002; Montagna et al., 2021). Hence, hydrographical trends changing over time is expected.

Of all variables measured, ambient water quality trends exhibited the fewest differences with distance from the discharge (Table 3). Overall, SO4, DON, and turbidity exhibited little or no influence from distance from the discharge site, while distance from the discharge site had greater influence on phosphorous, TOC, COD, DO, TDS, pH, salinity, and temperature. Spatial differences among salinity gradients, water temperatures, pH, nutrients, organic variables, and biological communities have been noted in previous studies (Bugica et al., 2020; Montagna, 2021; Montagna et al., 2020). The distance between sampling locations was small, 75% of stations (A–C) were within 183-m radius of the discharge site, and 25% of R stations were 3810 m away from the diffuser site (Fig. 1). Although this monitoring program is spatially intensive, it may not capture the actual trends in hydrography, biology, and sediments in the entire bay system.

Neither the change in the freshwater inflow index over time nor the seasonal changes influenced hydrographical parameter results among stations. Instead, the small scale of the site-specific sampling locations best explains these results. Differences between R stations and discharge stations are observed and can be attributable to larger distances that separate R stations from discharge stations (Fig. 1).

### Sediment dynamics

Presence of trace metals in Lavaca Bay was observed from 1993 to 2020 and is similar to results noted in previous studies (Carr et al., 2001; USEPA, 2001). Mercury was the only trace metal that exceeded effects range medium (ERM) and probable effects levels (PELs) but was discovered at sampling locations outside of the FPC study sampling radius and the FPC discharge sampling site (station 18) (Carr et al., 2001). Neither Carr et al. (2001) nor the current study found Hg concentrations exceeding ERM or PEL levels near the point of discharge. Both studies used the US Environmental Protection Agency (USEPA) 245.2 (USEPA, 1974) for mercury analyses. This method is applicable to surface water and may be applicable to saline waters, wastewaters, effluents, and domestic sewages providing potential interferences are not present. Mercury contamination was present near the ALCOA, Point Comfort site, which is about 5 km south of the discharge site. Carr et al. (2001) attributed mercury exceedances to contamination influence by the wastewater discharge from a chlor-alkali unit at ALCOA, which operated from 1965 to 1979. This unit used mercury to produce chlorine gas and sodium hydroxide. As a result, approximately 67 pounds of mercury per day was discharged into the bay prior to 1970 (ATSDR, 1999). Thus, high mercury concentrations in Lavaca Bay led to designation as one of 68 Superfund sites in Texas. Lavaca Bay’s Superfund designation was due to chlor-alkali production at the ALCOA Point Comfort Operations plant from the 1950s through the 1970s, which released potentially toxic levels of Hg that continue to persist in the bay (USEPA, 2006). However, the Superfund site is outside of the FPC study area.

Concentrations of metals that were detectable (Table 1) were decreasing over time, and the PCA indicates all the metals are changing similarly over time. One possible explanation for the concentration decreases is deposition of sediment over time capping older metal pollutants. Trace metals in sediments exhibit seasonal changes because the hot summer climate creates an oxygen-poor environment near the sediment–water interface, which causes chalcophilic metals (e.g., Cu, Hg, Zn) to precipitate from the water, resulting in high concentrations in the sediments near the source (Holmes, 1986). During winter, strong winds cause the entire water mass to become aerated, and oxidization and remobilization of some metals occur. However, in the current study, trace metal trends are changing among stations (Fig. 7C), but seasonality is not the main driver of trace metal trends (Fig. 7B). The metals located near the discharge site do not appear to pose a threat to the estuarine conditions of Lavaca Bay because the concentrations are below the ERM and PEL.

Sediment characteristics are changing over the long term (Fig. 8). Earlier, sand content was higher, and then, a shift occurred towards higher clay and silt content (mud) and less sand content. Sediment content also changed among stations with a higher mud content nearest to A stations and higher sand content nearest to C stations (further away from the discharge site). There is no evidence of seasonality and climatic wet-dry period changes on sediment parameter concentrations or characteristics. Currents, tides, and winds can move sediment in different ways and directions (Bloom et al., 2004; Heyes et al., 2004), but it appears the sediment change is due to the discharge.

The sediments nearest the discharge, A distance stations, have high clay and silt content and Hg, Cr, Cu, TOC, and NH4 concentrations. The change in sediment characteristics is closely tied to sedimentation via freshwater inflow events. Previous studies have found that sediment characteristics, such as sand and organic matter content, influence macrofaunal community dynamics (Chester et al., 1983; Flint & Kalke, 1985).

Freshwater inflow transports sediment, nutrients, and organic matter from the watershed to an estuary. Thus, the variability of freshwater inflow affects sediment, nutrient, and organic loading to estuaries (Russell et al., 2006). Oxygen demand, sulfates, and conventional water parameters in sediment increased with increasing freshwater inflow and were not influenced by sediment type. In contrast, inorganic and organic nutrients decreased when freshwater inflow was minimal and clay and silt sediments were predominant. A previous study observed that variability of suspended solids correlated with seasonal dynamics rather than variability of freshwater inflow in Lavaca Bay (Paudel et al., 2015). The same pattern occurred in the current study where nutrients and sedimentation were influenced by seasonal patterns and less so the freshwater inflow index.

### Biological dynamics

Benthic macrofaunal trends to climate variability, freshwater inflow trends, and contamination were primary study subjects historically and presently (Kim & Montagna, 2009; Locarnini & Presley, 1996; Montagna & Kalke, 1992, 1995; Pollack et al., 2011; Sager, 2002). These studies, and others, demonstrate that regional-scale processes and long-term hydrological cycles interact and regulate benthic abundance, productivity, diversity, and community structure. The current study is unique in that all trophic hierarchical levels are studied and examined for environmental influences or processes that interact and regulate the widespread biological community dynamics over time and space in efforts to gain an overview of the overall ecological condition of Lavaca Bay (Figs. 10 and 11).

Biological diversity and abundances commonly fluctuate over time and space on both large and minor scales (Magurran & Dornelas, 2010), and measuring species diversity and abundances of estuarine biological communities over long-term studies can be useful to assess estuarine environmental conditions (Bechtel & Copeland, 1970; Haedrich, 1975; Horn, 1980; Livingston, 1976; Yoklavich et al., 1991). Additionally, higher diversity corresponds with higher biological productivity, more resilient communities, and higher resistance to environmental stressors such as fluctuating freshwater inflow or presence of invasive species. The current dataset offers a long-term temporal scale to analyze trophic-level community dynamics on a small spatial scale. Assessment of small-scale spatial variation is essential to understanding the relationships between environmental factors and biological community structures in estuaries (Mannino & Montagna, 1997). To describe spatial variation patterns of biological communities, ANOVA, nMDS, and box plots were used. Benthic and zooplankton diversity and gill net and phytoplankton abundance and diversity were shown to change spatially (Table 3). The nMDS figures demonstrate the community structure of all biological groups were influenced by distance from discharge (Fig. 9).

There were differences among sampling trips for all biological trophic levels (Table 3). The temporal trends indicate phytoplankton and zooplankton abundance and diversity, and gill net diversity increases, trawl abundance and diversity decreases, and benthic abundance and diversity, ichthyoplankton abundance and diversity, and gill net abundance remain constant (Table 2).

Both phytoplankton and zooplankton abundance and log-abundance range over four orders of magnitude. Phytoplankton and zooplankton sample enumeration methods differed over time. Phytoplankton samples prior to 2007 were enumerated at a magnification of 400 × in a Palmer-Maloney counting chamber with a high dry (magnification of 40 ×) and, from 2008, were counted at a magnification of 100 × for larger celled phytoplankton and 1000 × for smaller sized phytoplankton. Thus, large-size phytoplankton counts were commonly low, but the smaller-size phytoplankton, typically referred to as picoplankton and two microns in size or smaller, yielded high counts from thousands to tens of thousands per millimeter. This accounts for the increase in magnitude of phytoplankton counts beginning in 2009. There was no change of zooplankton methodology despite a large change in abundance. Because of the shifts, analyses across trophic groups were limited to data collected after 2009.

Differences in benthic, gill net, and trawl communities are driven by climatic periods, a result evident in previous studies (Montagna & Kalke, 1995; Palmer & Montagna, 2015; Pollack et al., 2009, 2011). Seasonal patterns have been known to influence species populations within estuaries (De Ben et al., 1990; Horn, 1980), which affects both seasonal and annual species diversity patterns. Estuarine fish communities are known to have large temporal variation in species composition and abundance (Livingston, 1976, 1987; Rountree & Able, 1992; Tremain & Adams, 1995).

### Hydrographical influence on biological community trends

Salinity fluctuations and changing climatic conditions are integral to the structure and function of estuarine systems and have been found to influence biological community dynamics (MacKay et al., 2010; McLusky & Elliott, 2004; Pollack et al., 2011). A shift in biological community abundance and diversity occurred with changes in freshwater inflow and conventional water quality parameter trends in Lavaca Bay. The correlations between biological communities and hydrographical variables indicate freshwater inflow is positively influential on benthos diversity and abundance. Benthos are sessile organisms that continuously sample the overlying water conditions, and community composition fluctuations demonstrate a variety of consistent responses to the stress (Pollack et al., 2009). Soft-bottom infaunal macrofauna species possess a wide range of stress tolerances, including varying sensitivities to salinity fluctuations, and are often food for higher trophic levels (Kalke & Montagna, 1991; Longley, 1994). Thus, they are not the only important members of the estuarine community but are particularly useful in assessing freshwater inflow effects in estuarine systems, where salinity gradients can vary dramatically over time (Montagna, 2021). These shifts can occur rapidly as pulses and over long periods of time as presses. Contrary to the past study by Pollack et al. (2011), the benthic community in the present study is increasing rather than decreasing over time. An increase in salinity occurred in Lavaca Bay from 1993 to 2020. With high stress tolerance, benthos were resistant to salinity changes in the bay.

Trawl, gill net, and plankton groups abundances were negatively correlated with changing salinities. Freshwater transports nutrients and organic matter, which stimulates primary and secondary production in the water column and can enhance production of benthos (Montagna & Yoon, 1991). Salinity ranged from 0 to 35 psu in Lavaca Bay and varies frequently over time (Montagna et al., 2020; Pollack et al., 2011). The variation in salinity poses a threat to many biological communities by reducing diversity (Van Diggelen & Montagna, 2016), and with less freshwater inflow to the system, the less nutrient and sediment loading.

## Conclusion

Analyses of FPC data revealed four key findings: (1) Temporal variability driven by climate, inflow, and season is considerably more important than spatial variability with respect to distance from the discharge in explaining variability of the hydrographical and biological data, but not the sediment data. (2) All stations exhibited similar trends over time for all measured parameters except for sediments. (3) All biological groups exhibited different community structures in reference sites than in the discharge sites. (4) Some methods were inadequate to measure potential contaminant and water quality concentrations in the discharge and ambient waters and sediments. (5) There was no specific chemical marker for discharge effects.

## Supplementary Information

Below is the link to the electronic supplementary material.

Supplementary file1 (DOCX 5983 KB)
