# Supplementary material for: Influence of an industrial discharge on long-term dynamics of abiotic and biotic resources in Lavaca Bay, Texas, USA
Source: Environ Monit Assess. 2022 Oct 27;195(1):40. doi: 10.1007/s10661-022-10665-w (PMC9613729; doi:10.1007/s10661-022-10665-w)
Supplement: Supplementary file 1 — Supplementary file1 (DOCX 5983 KB) [file 10661_2022_10665_MOESM1_ESM.docx]

Electronic Supplementary Materials

**Influence of an industrial discharge on long-term dynamics of abiotic and biotic resources in Lavaca Bay, Texas, USA**

Elizabeth K. Harris^1^, Paul A. Montagna^1^*, Audrey R. Douglas^1^, Lisa Vitale^2^, David Buzan^2^

^1^Texas A&M University-Corpus Christi

Harte Research Institute for Gulf of Mexico Studies

6300 Ocean Drive, Unit 5869

Corpus Christi, Texas 78412 USA

^2^Freese and Nichols, Inc.

10431 Morado Circle, Bldg. 5, Suite 300

Austin, Texas 78759 USA

Office: (512) 617-3158

*Corresponding Author: Paul A. Montagna

August 2022

------------------------------------------------------------------------------------------------------

Contents

[Table S1: Subcontractor Groups and Assigned Analysis 2](#_Toc110850387)

[Table S2: Station Locations 3](#_Toc110850388)

[Table S3: Sampling Trips 4](#_Toc110850389)

[Table S4: Parameter Collection Methods 8](#_Toc110850390)

[Water 8](#_Toc110850391)

[Porewater 11](#_Toc110850392)

[Sediment and Tissue 14](#_Toc110850393)

[Table S5: Variable Detection and Non-Detection Rates 17](#_Toc110850394)

[Table S6: Species List 31](#_Toc110850395)

[Benthic Infauna 31](#_Toc110850396)

[Nekton in Gill Net Samples 40](#_Toc110850397)

[Nekton in Trawl Samples 42](#_Toc110850398)

[Phytoplankton 45](#_Toc110850399)

[Zooplankton 51](#_Toc110850400)

[Ichthyoplankton 54](#_Toc110850401)

[Figure S1: Time Series Plots 56](#_Toc110850402)

# Table S1: Subcontractor Groups and Assigned Analysis

| **Subcontractor** | **City, State** | **Abbreviation** | **Analysis** | **Trips** |
| --- | --- | --- | --- | --- |
| North Water District Laboratory Services, Inc. | Houston, TX; The Woodlands, TX | NWDLS | Chemical | 1-107 |
| Trace Element Research Laboratory of TAMU | College Station, TX | TAMU | Metal (Tissue) | 1-107 |
| NDRC Laboratories, Inc. | Houston, TX | NDRC | Chemical | 1 |
| AnalySys Inc. | Austin, TX | AnalySys | Chemical | 2-19 |
| CHEMTEX Laboratories Inc. | Corpus Christi, TX | CHEMTEX | Chemical | 20-21 |
| ANACON, Inc. | Houston, TX | ANACON | Chemical | 22-79 |
| A&B Labs | Houston, TX | A&B | Chemical | 80-107 |
| EH&A Bioassay Laboratory | Houston, TX | EH&A | Bioassay | 1-23 |
| PBS&J Bioassay Laboratory | Houston, TX | PBS&J | Bioassay | 24-27 |
| PBS&J Environmental Toxicology Laboratory | Houston, TX | PBS&J | Bioassay | 28-71 |
| Atkins Environmental Toxicology Laboratory | Houston, TX | Atkins | Bioassay | 72-95 |
| NWDLS Toxicology Laboratory (formerly Atkins Environmental Toxicology Laboratory) | Houston, TX | NWDLS | Bioassay | 96-107 |
| EH&A | Houston, TX | EH&A | Nekton | 1-23 |
| PBS&J | Austin, TX | PBS&J | Nekton | 24-71 |
| Atkins | Austin, TX | Atkins | Nekton | 72-87 |
| Scott Holt, UTMSI | Port Aransas, TX | UTMSI | Ichthyoplankton | 1-27, 39-67 |
| PBS&J | Austin, TX | PBS&J | Ichthyoplankton | 28-38 |
| Greg Stunz, TAMUCC | Corpus Christi, TX | TAMUCC | Ichthyoplankton | 64-107 |
| Paul Price & Associates | Austin, TX | Price | Benthos, Phytoplankton, Zoophytoplankton | 1-51 |
| HDR Engineering, Inc. (Formerly Paul Price & Associates, Inc.) | Austin, TX | HDR | Benthos, Phytoplankton, Zoophytoplankton | 52-63 |
| Dr. Jerry McLelland, University of Southern Mississippi, Gulf Research Laboratory | Ocean Springs, MS | USMGRL | Benthos | 64-107 |
| PBS&J | Austin, TX | PBS&J | Phytoplankton, Zooplankton | 64-71 |
| Atkins | Austin, TX | Atkins | Phytoplankton, Zooplankton | 72-87 |
| TAI Environmental Sciences, Inc. | Mobile, AL | TAI | Red Algae | 1-19 |
| Triangle Laboratories of RTP, Inc. | Durham, NC | Triangle | Dioxin | 1-6 |

# Table S2: Station Locations

The Formosa Discharge is located at 28.680963 N, -96.582103 W. Abbreviations: FPC = Formosa Plastics Corporation station name, TCEQ = Texas Commission on Environmental Quality station name, HRI = Harte Research Institute Station name, Distance= distance from discharge location, ft = feet, m = meters, Direction = direction from the discharge.

| **FPC** | **TCEQ** | **Distance (ft)** | **Distance (m)** | **Direction** | **Latitude** | **Longitude** |
| --- | --- | --- | --- | --- | --- | --- |
| A1 | 14116 | 50 | 15.24 | NW | 28.681056 | -96.582225 |
| A2 | 14118 | 50 | 15.24 | SW | 28.680833 | -96.582200 |
| A3 | 14119 | 50 | 15.24 | SE | 28.680883 | -96.581981 |
| A4 | 14120 | 50 | 15.24 | NE | 28.681078 | -96.582006 |
| B1 | 14121 | 250 | 76.2 | NW | 28.681483 | -96.582836 |
| B2 | 14122 | 200 | 60.96 | SW | 28.680539 | -96.582492 |
| B3 | 14123 | 200 | 60.96 | SE | 28.680628 | -96.581614 |
| B4 | 14124 | 200 | 60.96 | NE | 28.681400 | -96.581714 |
| C1 | 14125 | 600 | 182.88 | NW | 28.681997 | -96.583569 |
| C2 | 14126 | 600 | 182.88 | SW | 28.679678 | -96.583267 |
| C3 | 14127 | 600 | 182.88 | SE | 28.679944 | -96.580628 |
| C4 | 14128 | 600 | 182.88 | NE | 28.682264 | -96.580939 |
| D1 | 14129 | 1500 | 457.2 | NW | 28.683536 | -96.585767 |
| D2 | 14130 | 1500 | 457.2 | SW | 28.677742 | -96.585011 |
| D3 | 14131 | 1500 | 457.2 | SE | 28.678406 | -96.578439 |
| R1 | 14132 | 12500 | 3810 |  | 28.703267 | -96.612733 |
| R2 | 14133 | 12500 | 3810 |  | 28.675200 | -96.623150 |
| R3 | 14134 | 12500 | 3810 |  | 28.652150 | -96.596250 |
| R4 | 14135 | 12500 | 3810 |  | 28.714233 | -96.574100 |
| R5 | 14161 |  |  |  | 28.688341 | -96.564195 |
| M22 |  |  |  |  | 28.679720 | -96.582200 |
|  |  |  |  |  |  |  |
| **HRI** |  | **Distance (ft)** | **Distance (m)** | **Direction** | **Latitude** | **Longitude** |
| A |  | 2296 | 700 |  | 28.674670 | -96.582680 |
| B |  | 15392 | 4691 |  | 28.638680 | -96.584370 |
| FD |  | 25 | 8 |  | 28.680960 | -96.582180 |

# Table S3: Sampling Trips

Sampling trip names and dates and the annual report periods.

| **Monitoring Event** | **Annual Report/Sampling Dates** |
| --- | --- |
| **Year 1** | **1st Annual Report** |
| **Pre-Discharge** |  |
| Trip 1 | 17–26 May 1993 |
| Trip 2A | 26–29 July 1993 |
| Trip 2B | 21–23 Sept 1993 |
| **Post-Discharge** |  |
| Trip 3 | 11–13 Oct 1993 |
| Trip 4 | 6–8 December 1993 |
| Trip 5 | 21–25 Feb 1994 |
| Trip 6 | 18–22 April 1994 |
| **Year 2** | **2nd Annual Report** |
| Trip 7 | 20–24 June 1994 |
| Trip 8 | 22–25 Aug 1994 |
| Trip 9 | 17–26 Oct 1994 |
| Trip 10 | 16–19 Jan 1995 |
| Trip 11 | 17–20 April 1995 |
| **Year 3** | **3rd Annual Report** |
| Trip 12 | 24–27 July 1995 |
| Trip 13 | 16–19 Oct 1995 |
| Trip 14 | 8–11 Jan 1996 |
| Trip 15 | 15–18 April 1996 |
| **Year 4** | **4th Annual Report** |
| Trip 16 | 15 –19 July 1996 |
| Trip 17 | 14–17 Oct 1996 |
| Trip 18 | 20–24 Jan 1997 |
| Trip 19 | 21–24 April 1997 |
| **Year 5** | **5th Annual Report** |
| Trip 20 | 21–23 July 1997 |
| Trip 21 | 20–22 Oct 1997 |
| Trip 22 | 19–22 Jan 1998 |
| Trip 23 | 20–23 April 1998 |
| **Year 6** | **6th Annual Report** |
| Trip 24 | 20–23 July 1998 |
| Trip 25 | 27–30 Oct 1998 |
| Trip 26 | 18–22 Jan 1999 |
| Trip 27 | 17–21 May 1999 |
| **Year 7** | **7th Annual Report** |
| Trip 28 | 20–23 Sept 1999 |
| Trip 29 | 8–11 Nov 1999 & 8–10 Dec 1999 |
| Trip 30 | 10–13 Jan 2000 |
| Trip 31 | 1–7 April 2000 |
| **Year 8** | **8th Annual Report** |
| Trip 32 | 17–20 July 2000 |
| Trip 33 | 16–25 Oct 2000 |
| Trip 34 | 22–25 Jan 2001 |
| Trip 35 | 9–12 April 2001 |
| **Year 9** | **9th Annual Report** |
| Trip 36 | 23–26 July 2001 |
| Trip 37 | 22–26 Oct 2001 |
| Trip 38 | 21–24 Jan 2002 |
| Trip 39 | 15–18 April 2002 |
| **Year 10** | **10th Annual Report** |
| Trip 40 | 29 July–1 Aug 2002 |
| Trip 41 | 15–17 Oct 2002 |
| Trip 42 | 13–16 Jan 2003 |
| Trip 43 | 21–24 April 2003 |
| **Year 11** | **11th Annual Report** |
| Trip 44 | 28–31 July 2003 |
| Trip 45 | 20–23 Oct 2003 |
| Trip 46 | 19–22 Jan 2004 |
| Trip 47 | 19–22 April 2004 |
| **Year 12** | **12th Annual Report** |
| Trip 48 | 26–29 July 2004 |
| Trip 49 | 18–21 Oct 2004 |
| Trip 50 | 24–27 Jan 2005 |
| Trip 51 | 25–28 April 2005 |
| **Year 13** | **13th Annual Report** |
| Trip 52 | 25–28 July 2005 |
| Trip 53 | 10–13 Oct 2005 |
| Trip 54 | 23–26 Jan 2006 |
| Trip 55 | 17–21 April 2006 |
| **Year 14** | **14th Annual Report** |
| Trip 56 | 17–20 July 2006 |
| Trip 57 | 16–19 Oct 2006 |
| Trip 58 | 23–25 Jan 2007 |
| Trip 59 | 9–12 April 2007 |
| **Year 15** | **15th Annual Report** |
| Trip 60 | 23–26 July 2007 |
| Trip 61 | 22–25 Oct & 5-6 Nov 2007 |
| Trip 62 | 14–17 Jan 2008 |
| Trip 63 | 21–24 April 2008 |
| **Year 16** | **16th Annual Report** |
| Trip 64 | 21–24 July 2008 |
| Trip 65 | 20–23 Oct 2008 |
| Trip 66 | 19–22 Jan 2009 |
| Trip 67 | 6–9 April 2009 |
| **Year 17** | **17th Annual Report** |
| Trip 68 | 13–16 July 2009 |
| Trip 69 | 19–22 Oct 2009 |
| Trip 70 | 18–21 Jan 2010 |
| Trip 71 | 5–8 April 2010 |
| **Year 18** | **18th Annual Report** |
| Trip 72 | 26–29 July 2010 |
| Trip 73 | 25–28 Oct 2010 |
| Trip 74 | 17–20 Jan 2011 |
| Trip 75 | 4–7 April 2011 |
| **Year 19** | **19th Annual Report** |
| Trip 76 | 25–28 July 2011 |
| Trip 77 | 17–20 Oct 2011 |
| Trip 78 | 23–26 Jan 2012 |
| Trip 79 | 2–5 April 2012 |
| **Year 20** | **20th Annual Report** |
| Trip 80 | 23–26 Jul & 31 Jul–1 Aug 2012 |
| Trip 81 | 15–18 Oct 2012 |
| Trip 82 | 21–24 Jan 2013 |
| Trip 83 | 1–4 April 2013 |
| **Year 21** | **21st Annual Report** |
| Trip 84 | 19–22 Aug 2013 |
| Trip 85 | 21–24 Oct 2013 |
| Trip 86 | 20–23 Jan 2014 |
| Trip 87 | 31 Mar– April 2014 |
| **Year 22** | **22nd Annual Report** |
| Trip 88 | 6–9 Oct 2014 |
| Trip 89 | 10–13 Nov 2014 |
| Trip 90 | 19–22 Jan 2015 |
| Trip 91 | 6–9 April 2015 |
| **Year 23** | **23rd Annual Report** |
| Trip 92 | 20–23 July 2015 |
| Trip 93 | 19–22 Oct 2015 |
| Trip 94 | 11–14 Jan 2016 |
| Trip 95 | 4–7 April 2016 |
| **Year 24** | **24th Annual Report** |
| Trip 96 | 31 Oct–3 Nov 2016 |
| Trip 97 | 5–8 December 2016 |
| Trip 98 | 16–19 Jan 2017 |
| Trip 99 | 3–6 April 2017 |
| **Year 25** | **25th Annual Report** |
| Trip 100 | 10–13 July 2017 |
| Trip 101 | 23–26 Oct 2017 |
| Trip 102 | 22–25 Jan 2018 |
| Trip 103 | 2–5 April 2018 |
| **Year 26** | **26th Annual Report** |
| Trip 104 | 23–26 July 2018 |
| Trip 105 | 13–16 Nov 2018 |
| Trip 106 | 14–17 Jan 2019 |
| Trip 107 | 1–4 April 2019 |
| **Year 27** | **27th Annual Report** |
| Trip 108 | 22–25 July 2019 |
| Trip 109 | 8–10 Oct 2019 |
| Trip 110 | 20–23 Jan 2020 |
| Trip 111 | 30–31 Mar & 1–4 April 2020 |

# Table S4: Parameter Collection Methods

## Water

Analytical methodology and detection limits. Abbreviations: MAL = Method Analytical Levels, MDL = Method Detection Limits.

| **Parameter** | **MAL** | **Method** | **MDL** |
| --- | --- | --- | --- |
| *SUITE II* |  |  |  |
| BOD5 | 2 mg/l | SM 5210 | 2 |
| COD^1^ | 5 mg/l | Hach 8000 | 5 |
| Organic Nitrogen | 0.1 mg/l | 351.4 | 0.1 |
| Ammonia Nitrogen | 0.1 mg/l | SM 4500 NH3 D | 0.1 |
| Nitrate Nitrogen | 0.1 mg/l | SM 4500 NO3 D | 0.1 |
| Total Phosphorus | 0.01 mg/l | 365.2 | 0.01 |
| Ortho Phosphate | 0.01 mg/l | 365.2 | 0.01 |
| TOC | 1 mg/l | 415.2 | 1 |
| TSS | 4 mg/l | 160.2 | 4 |
| Chlorides^1^ | 5 mg/l | SM 4500 C1 B | 5 |
| Turbidity (NTU) | 0.1 mg/l | 180.1 | 0.1 |
| Oil and Grease^2^ | 5 mg/l | 1664 | 5 |
| Sulfate | 1 mg/l | 375.4 | 1 |
| Cyanide (Total) | 0.01 mg/l | SM 4500 CN E | 0.01 |
| TDS | 10 mg/l | 160.1 | 10 |
| Nitrite Nitrogen | 0.01 mg/l | Hach 8507 | 0.01 |
| *SUITE IV* |  |  |  |
| *METALS* |  |  |  |
| Total Chromium (total and dissolved) | 10 μg/l | 200.7 | 10 |
| Copper (total and dissolved) | 10 μg/l | 200.7 | 10 |
| Lead (total and dissolved) | 5 μg/l | 200.7 | 1.0 |
| Mercury (dissolved only) | 0.2 μg/l | 245.2 | 0.2 |
| Nickel (total and dissolved) | 10 μg/l | 200.7 | 10 |
| Zinc (total and dissolved) | 5 μg/l | 200.7 | 0.05 |
| *PAHs* |  |  |  |
| Acenaphthene | 10 μg/l | 625 | 10 |
| Acenaphthylene | 10 μg/l | 625 | 10 |
| Anthracene | 10 μg/l | 625 | 10 |
| Benzo(a)anthracene | 10 μg/l | 625 | 10 |
| Benzo(k)fluoranthene | 10 μg/l | 625 | 10 |
| 3,4-Benzofluoranthene | 10 μg/l | 625 | 10 |
| Benzo(a)pyrene | 10 μg/l | 625 | 10 |
| Chrysene | 10 μg/l | 625 | 10 |
| Fluoranthene | 10 μg/l | 625 | 10 |
| Fluorene | 10 μg/l | 625 | 10 |
| Naphthalene | 20 μg/l | 625 | 10 |
| Phenanthrene | 20 μg/l | 625 | 10 |
| Pyrene | 20 μg/l | 625 | 10 |

| **Parameter** | **MAL** | **Method** | MDL |
| --- | --- | --- | --- |
| *VOLATILES* |  |  |  |
| Acrylonitrile | 50 μg/l | 624 | 5 |
| Benzene | 10 μg/l | 624 | 5 |
| Carbon Tetrachloride | 10 μg/l | 624 | 5 |
| Chlorobenzene | 10 μg/l | 624 | 6 |
| Chloroethane | 10 μg/l | 624 | 5 |
| Chloroform | 10 μg/l | 624 | 5 |
| Chloromethane | 10 μg/l | 624 | 5 |
| 1,1-Dichloroethane | 10 μg/l | 624 | 5 |
| 1,2-Dichloroethane | 10 μg/l | 624 | 5 |
| 1,1-Dichloroethene | 10 μg/l | 624 | 5 |
| Trans-1,2-Dichloroethene | 10 μg/l | 624 | 5 |
| 1,2,-Dichloropropane | 10 μg/l | 624 | 5 |
| Cis-1,3-Dichloropropene | 10 μg/l | 624 | 5 |
| Trans-1,3-Dichloropropene | 10 μg/l | 624 | 5 |
| Ethylbenzene | 10 μg/l | 624 | 5 |
| Methylene Chloride | 20 μg/l | 624 | 5 |
| Tetrachloroethene | 10 μg/l | 624 | 5 |
| Toluene | 10 μg/l | 624 | 5 |
| 1,1,1-Trichloroethane | 10 μg/l | 624 | 5 |
| 1,1,2-Trichloroethane | 10 μg/l | 624 | 5 |
| Trichloroethene | 10 μg/l | 624 | 5 |
| Vinyl Chloride | 10 μg/l | 624 | 5 |
| *SEMI-VOLATILES* |  |  |  |
| Bis(2-Ethylhexyl)phthalate | 10 μg/l | 625 | 10 |
| Butylbenzyl Phthalate | 10 μg/l | 625 | 10 |
| 4-Chloro-3-cresol | 10 μg/l | 625 | 10 |
| 2-Chlorophenol | 10 μg/l | 625 | 10 |
| Di-N-Butyl Phthalate | 10 μg/l | 625 | 10 |
| 1,2-Dichlorobenzene | 10 μg/l | 625 | 10 |
| 1,3-Dichlorobenzene | 10 μg/l | 625 | 10 |
| 1,4-Dichlorobenzene | 10 μg/l | 625 | 10 |
| 2,4-Dichlorophenol | 10 μg/l | 625 | 10 |
| Diethyl Phthalate | 10 μg/l | 625 | 10 |
| 2,4-Dimethylphenol | 10 μg/l | 625 | 10 |
| Dimethyl Phthalate | 10 μg/l | 625 | 10 |
| 4,6-Dinitro-2-Methylphenol | 10 μg/l | 625 | 10 |
| 2,4-Dinitrophenol | 50 μg/l | 625 | 50 |
| 2,4-Dinitrotoluene | 10 μg/l | 625 | 10 |
| 2,6-Dinitrotoluene | 10 μg/l | 625 | 10 |
| Hexachlorobenzene | 10 μg/l | 625 | 10 |
| Hexachlorobutadiene | 10 μg/l | 625 | 10 |
| Hexachloroethane | 20 μg/l | 625 | 10 |
| Nitrobenzene | 20 μg/l | 625 | 10 |
| 2-Nitrophenol | 10 μg/l | 625 | 10 |
| 4-Nitrophenol | 10 μg/l | 625 | 10 |
| Phenol | 10 μg/l | 625 | 10 |
| 1,2,4-Trichlorobenzene | 10 μg/l | 625 | 10 |

**Notes:**

U.S. EPA, "Test Methods for the Evaluation of Solid Waste," SW-846, November 1990.

U.S. EPA, "Manual of Analytical Methods for the Analysis of Pesticide Residues in Human and Environmental Samples," 1977.

U.S. EPA, "Methods for Chemical Analysis of Water and Waste," EPA 600/4-79-020, 1970. Organochlorine Pesticides and PCB's, EPA method 608; Federal Register. Vol. 44, No. 233, 12-3-79. Acid Digestion of Aqueous Samples for Graphite Furnace. EPA Method 3020.

Acid Digestion of Sediments, Sludges and Soils. EPA Method 3050. Analysis of Acid, Base, Neutral. EPA Method 625.

Hach Water Analysis Handbook, 2nd Edition. 1992.

Riggin, R.M., and Strup, P.E. "Screening Methods for PAH priority pollutants in wastewater." EPA-600/4-84- 007, NTIS #PB84-132992. Environmental Monitoring and Support Laboratory, Office of Research and Development, Cincinnati, Ohio, 1984

1 1996 Scope specified MAL for chlorides of 0.5 was revised to 5.0 due to laboratory equipment changes (NWDLS only).

2 1996 Scope specified MAL for oil and grease of 0.2 was found to be erroneous and subsequently changed to 5.0 (NWDLS only).

## Porewater

| **Parameter** | **MAL** | **Method** | **MDL** |
| --- | --- | --- | --- |
| SUITE II |  |  |  |
| BOD5 | 2 mg/l | SM 5210 | 2 |
| COD | 5 mg/l | Hach 8000 | 5 |
| Organic Nitrogen | 0.1 mg/l | 351.4 | 0.1 |
| Ammonia Nitrogen | 0.1 mg/l | SM 4500 NH3 D | 0.1 |
| Nitrate Nitrogen | 0.1 mg/l | SM 4500 NO3 D | 0.1 |
| Total Phosphorus | 0.01 mg/l | 365.2 | 0.01 |
| Ortho Phosphate | 0.01 mg/l | 365.2 | 0.01 |
| TOC | 1 mg/l | 415.2 | 1 |
| TSS | 4 mg/l | 160.2 | 4 |
| Chlorides^1^ | 5 mg/l | SM 4500 C1 B | 5 |
| Turbidity (NTU) | 0.1 mg/l | 180.1 | 0.1 |
| Oil and Grease^2^ | 5 mg/l | 1664 | 5 |
| Sulfate | 1 mg/l | 375.4 | 1 |
| Cyanide (Total) | 0.01 mg/l | SM 4500 CN E | 0.01 |
| TDS | 10 mg/l | 160.1 | 10 |
| Nitrite Nitrogen | 0.01 mg/l | Hach 8507 | 0.01 |
| *SUITE IV* |  |  |  |
| *METALS* |  |  |  |
| Aluminum^3^ | 30 μg/l | 200.7 | 30 |
| Arsenic | 10 μg/l | 200.7 | 10 |
| Total Chromium | 10 μg/l | 200.7 | 10 |
| Hexavalent Chromium | 10 μg/l | Hach 8023 | 10 |
| Copper | 10 μg/l | 200.7 | 10 |
| Lead | 5 μg/l | 200.7 | 1.0 |
| Mercury | 0.2 μg/l | 200.7 | 0.2 |
| Nickel | 10 μg/l | 200.7 | 10 |
| Zinc | 5 μg/l | 200.7 | 0.05 |
| PAHs |  |  |  |
| Acenaphthene | 10 μg/l | 625 | 10 |
| Acenaphthylene | 10 μg/l | 625 | 10 |
| Anthracene | 10 μg/l | 625 | 10 |
| Benzo(a)anthracene | 10 μg/l | 625 | 10 |
| Benzo(k)fluoranthene | 10 μg/l | 625 | 10 |

| **Parameter** | **MAL** | **Method** | **MDL** |
| --- | --- | --- | --- |
| *SUITE IV (cont.)* |  | | |
| *PAHs (cont)* |  | | |
| 3,4-Benzofluoranthene | 10 μg/l | 625 | 10 |
| Benzo(a)pyrene | 10 μg/l | 625 | 10 |
| Chrysene | 10 μg/l | 625 | 10 |
| Fluoranthene | 10 μg/l | 625 | 10 |
| Fluorene | 10 μg/l | 625 | 10 |
| Naphthalene | 20 μg/l | 625 | 10 |
| Phenanthrene | 20 μg/l | 625 | 10 |
| Pyrene | 20 μg/l | 625 | 10 |
| *VOLATILES* |  |  |  |
| Acrylonitrile | 50 μg/l | 624 | 5 |
| Benzene | 10 μg/l | 624 | 5 |
| Carbon Tetrachloride | 10 μg/l | 624 | 5 |
| Chlorobenzene | 10 μg/l | 624 | 6 |
| Chloroethane | 10 μg/l | 624 | 5 |
| Chloroform | 10 μg/l | 624 | 5 |
| Chloromethane | 10 μg/l | 624 | 5 |
| 1,1-Dichloroethane | 10 μg/l | 624 | 5 |
| 1,2-Dichloroethane | 10 μg/l | 624 | 5 |
| 1,1-Dichloroethene | 10 μg/l | 624 | 5 |
| Trans-1,2-Dichloroethene | 10 μg/l | 624 | 5 |
| 1,2,-Dichloropropane | 10 μg/l | 624 | 5 |
| Cis-1,3-Dichloropropene | 10 μg/l | 624 | 5 |
| Trans-1,3-Dichloropropene | 10 μg/l | 624 | 5 |
| Ethylbenzene | 10 μg/l | 624 | 5 |
| Methylene Chloride | 20 μg/l | 624 | 5 |
| Tetrachloroethene | 10 μg/l | 624 | 5 |
| Toluene | 10 μg/l | 624 | 5 |
| 1,1,1-Trichloroethane | 10 μg/l | 624 | 5 |
| 1,1,2-Trichloroethane | 10 μg/l | 624 | 5 |
| Trichloroethene | 10 μg/l | 624 | 5 |
| Vinyl Chloride | 10 μg/l | 624 | 5 |
| *SEMI-VOLATILES* |  |  |  |
| Bis(2-Ethylhexyl)phthalate | 10 μg/l | 625 | 10 |
| Butylbenzyl Phthalate | 10 μg/l | 625 | 10 |
| 4-Chloro-3-cresol | 10 μg/l | 625 | 10 |
| 2-Chlorophenol | 10 μg/l | 625 | 10 |
| Di-N-Butyl Phthalate | 10 μg/l | 625 | 10 |
| 1,2-Dichlorobenzene | 10 μg/l | 625 | 10 |
| 1,3-Dichlorobenzene | 10 μg/l | 625 | 10 |

| **Parameter** | **MAL** | **Method** | **MDL** |
| --- | --- | --- | --- |
| *SUITE IV (cont.)* |  |  |  |
| *SEMI-VOLATILES* |  |  |  |
| 1,4-Dichlorobenzene | 10 μg/l | 625 | 10 |
| 2,4-Dichlorophenol | 10 μg/l | 625 | 10 |
| Diethyl Phthalate | 10 μg/l | 625 | 10 |
| 2,4-Dimethylphenol | 10 μg/l | 625 | 10 |
| Dimethyl Phthalate | 10 μg/l | 625 | 10 |
| 4,6-Dinitro-2-Methylphenol | 10 μg/l | 625 | 10 |
| 2,4-Dinitrophenol | 50 μg/l | 625 | 50 |
| 2,4-Dinitrotoluene | 10 μg/l | 625 | 10 |
| 2,6-Dinitrotoluene | 10 μg/l | 625 | 10 |
| Hexachlorobenzene | 10 μg/l | 625 | 10 |
| Hexachlorobutadiene | 10 μg/l | 625 | 10 |
| Hexachloroethane | 20 μg/l | 625 | 10 |
| Nitrobenzene | 20 μg/l | 625 | 10 |
| 2-Nitrophenol | 10 μg/l | 625 | 10 |
| 4-Nitrophenol | 10 μg/l | 625 | 10 |
| Phenol | 10 μg/l | 625 | 10 |
| 1,2,4-Trichlorobenzene | 10 μg/l | 625 | 10 |

U.S. EPA, "Test Methods for the Evaluation of Solid Waste," SW-846, November 1990.

U.S. EPA, "Manual of Analytical Methods for the Analysis of Pesticide Residues in Human and Environmental Samples," 1977.

U.S. EPA, "Methods for Chemical Analysis of Water and Waste," EPA 600/4-79-020, 1970. Organochlorine Pesticides and PCB's, EPA method 608; Federal Register. Vol. 44, No. 233, 12-3-79. Acid Digestion of Aqueous Samples for Graphite Furnace. EPA Method 3020.

Acid Digestion of Sediments, Sludges and Soils. EPA Method 3050. Analysis of Acid, Base, Neutral. EPA Method 625.

Hach Water Analysis Handbook, 2nd Edition. 1992.

Riggin, R.M., and Strup, P.E. "Screening Methods for PAH priority pollutants in wastewater." EPA-600/4-84- 007, NTIS #PB84-132992. Environmental Monitoring and Support Laboratory, Office of Research and Development, Cincinnati, Ohio, 1984.

1 1996 Scope specified MAL for chlorides of 0.5 was revised to 5.0 due to laboratory equipment changes.

2 1996 Scope specified MAL for oil and grease of 0.2 was found to be erroneous and subsequently changed to 5.0.

3 1996 Scope specified MAL for aluminum of 20 was revised to 30 due to laboratory equipment/method changes.

## Sediment and Tissue

| **Parameter** | **MAL** | **Method** | **MDL** |
| --- | --- | --- | --- |
| *SUITE III* |  |  |  |
| Oil and Grease | 10 mg/kg | 1664 | 10 |
| Ammonia - Nitrogen | 10 mg/kg | SM 4500 NH3 C | 10 |
| Organic - Nitrogen | 10 mg/kg | SM 4500 N C | 10 |
| Nitrate - Nitrogen | 10 mg/kg | SM 4500 NO3 D | 10 |
| Nitrite - Nitrogen | 1 mg/kg | Hach 8507 | 1 |
| Volatile Solids | 0.1 mg/kg | 160.4 | 0.1 |
| Total PAH | 0.5 mg/kg | SW-846 8270 | 0.5 |
| Acid - Volatile Sulfide | 1 mg/kg | SW-846 9030 | 1 |
| TOC | 0.1 mg/kg | 415.1 | 0.1 |
| Cyanide (Total) | 1 mg/kg | SM 4500 CN E | 1 |
| Grain Size Analysis | N/A | K&A, 1949 | N/A |
|  |  |  |  |
| *SUITE IV METALS* |  |  |  |
| Aluminum | 3 mg/kg | 200.7 | 3 |
| Arsenic | 1 mg/kg | 200.7 | 1 |
| Chromium | 1 mg/kg | 200.7 | 1 |
| Copper | 1 mg/kg | 200.7 | 1 |
| Iron | 3 mg/kg | 200.7 | 2 |
| Lead | 0.5 mg/kg | 200.7 | 0.5 |
| Mercury | 0.02 mg/kg | 200.7 | 0.05 |
| Nickel | 1 mg/kg | 200.7 | 1 |
| Zinc | 0.5 mg/kg | 200.7 | 0.5 |
|  |  |  |  |
| *SUITE IV PAHs* |  |  |  |
| Acenaphthene | 150 μg/kg | 8270 | 150 |
| Acenaphthylene | 150 μg/kg | 8270 | 150 |
| Anthracene | 150 μg/kg | 8270 | 150 |
| Benzo(a)anthracene | 150 μg/kg | 8270 | 150 |
| Benzo(k)fluoranthene | 150 μg/kg | 8270 | 150 |
| 3,4-Benzofluoranthene | 150 μg/kg | 8270 | 150 |
| Benzo(a)pyrene | 150 μg/kg | 8270 | 150 |
| Chrysene | 150 μg/kg | 8270 | 150 |
| Fluoranthene | 150 μg/kg | 8270 | 150 |
| Fluorene | 150 μg/kg | 8270 | 150 |
| Naphthalene | 150 μg/kg | 8270 | 150 |
| Phenanthrene | 150 μg/kg | 8270 | 150 |
| Pyrene | 150 μg/kg | 8270 | 150 |

| **Parameter** | **MAL** | **Method** | **MDL** |
| --- | --- | --- | --- |
| *VOLATILES** |  |  |  |
| Acrylonitrile | 100 μg/kg | 8260 | 100 |
| Benzene | 10 μg/kg | 8260 | 10 |
| Carbon Tetrachloride | 10 μg/kg | 8260 | 10 |
| Chlorobenzene | 10 μg/kg | 8260 | 10 |
| Chloroethane | 10 μg/kg | 8260 | 10 |
| Chloroform | 10 μg/kg | 8260 | 10 |
| Chloromethane | 10 μg/kg | 8260 | 10 |
| 1,1-Dichloroethane | 10 μg/kg | 8260 | 10 |
| 1,2-Dichloroethane | 10 μg/kg | 8260 | 10 |
| 1,1-Dichloroethene | 10 μg/kg | 8260 | 10 |
| Trans-1,2-Dichloroethene | 10 μg/kg | 8260 | 10 |
| 1,2,-Dichloropropane | 10 μg/kg | 8260 | 10 |
| Cis-1,3-Dichloropropene | 10 μg/kg | 8260 | 10 |
| Trans-1,3-Dichloropropene | 10 μg/kg | 8260 | 10 |
| Ethylbenzene | 10 μg/kg | 8260 | 10 |
| Methylene Chloride | 10 μg/kg | 8260 | 10 |
| Tetrachloroethene | 10 μg/kg | 8260 | 10 |
| Toluene | 10 μg/kg | 8260 | 10 |
| 1,1,1-Trichloroethane | 10 μg/kg | 8260 | 10 |
| 1,1,2-Trichloroethane | 10 μg/kg | 8260 | 10 |
| Trichloroethene | 10 μg/kg | 8260 | 10 |
| Vinyl Chloride | 10 μg/kg | 8260 | 10 |
| *SEMI-VOLATILES** |  |  |  |
| Bis(2-Ethylhexyl)phthalate | 150 μg/kg | 8270 | 150 |
| Butylbenzyl Phthalate | 150 μg/kg | 8270 | 150 |
| 4-Chloro-3-cresol | 300 μg/kg | 8270 | 300 |
| 2-Chlorophenol | 150 μg/kg | 8270 | 150 |
| Di-N-Butyl Phthalate | 150 μg/kg | 8270 | 150 |
| 1,2-Dichlorobenzene | 150 μg/kg | 8270 | 150 |
| 1,3-Dichlorobenzene | 150 μg/kg | 8270 | 150 |
| 1,4-Dichlorobenzene | 150 μg/kg | 8270 | 150 |
| 2,4-Dichlorophenol | 150 μg/kg | 8270 | 150 |
| Diethyl Phthalate | 150 μg/kg | 8270 | 150 |
| 2,4-Dimethylphenol | 150 μg/kg | 8270 | 150 |
| Dimethyl Phthalate | 150 μg/kg | 8270 | 150 |
| 4,6-Dinitro-2- | 710 μg/kg | 8270 | 710 |

| **Parameter** | **MAL** | **Method** | **MDL** |
| --- | --- | --- | --- |
| *SUITE IV (cont.)*  *SEMI-VOLATILES** |  | | |
| 2,4-Dinitrophenol | 710 μg/kg | 8270 | 710 |
| 2,4-Dinitrotoluene | 150 μg/kg | 8270 | 150 |
| 2,6-Dinitrotoluene | 150 μg/kg | 8270 | 150 |
| Hexachlorobenzene | 150 μg/kg | 8270 | 150 |
| Hexachlorobutadiene | 150 μg/kg | 8270 | 150 |
| Hexachloroethane | 150 μg/kg | 8270 | 150 |
| Nitrobenzene | 150 μg/kg | 8270 | 150 |
| 2-Nitrophenol | 150 μg/kg | 8270 | 150 |
| 4-Nitrophenol | 710 μg/kg | 8270 | 710 |
| Phenol | 150 μg/kg | 8270 | 150 |
| 1,2,4-Trichlorobenzene | 150 μg/kg | 8270 | 150 |

**Notes:**

* All samples reported on a dry weight basis.

U.S. EPA, "Methods for Chemical Analysis of Water and Waste," EPA 600/4-79-020, 1979.

U.S. EPA, "Manual of Analytical Methods for the Analysis of Pesticide Residues in Human and Environmental Samples," 1977.

U.S. EPA, "Test Methods for Evaluating Solid Waste, Physical/Chemical Methods," SW-846, 3rd Edition, November 1990.

Organochlorine Pesticides and PCB's, EPA method 608; Federal Register. Vol. 44, No. 233, 12-3-79. Analysis of Acid, Base, Neutral. EPA Method 625.

Riggin, R.M., and Strup, P.E. "Screening Methods for PAH priority pollutants in wastewater." EPA-600/4-84- 007, NTIS #PB84-132992. Environmental Monitoring and Support Laboratory, Office of Research and Development, Cincinnati, Ohio, 1984

1 1996 Scope specified MAL for oil and grease of 1.0 was not appropriate based on the EPA approved analytical method and therefore was revised to 10.0.

2 1996 Scope specified MAL for aluminum of 2.0 was revised to 3.0 due to laboratory equipment/method changes.

# Table S5: Variable Detection and Non-Detection Rates

Total number of measurements and percent (PCT) that are above or below detection limits. Abbreviations: Con = conventional, Ino = inorganic, Met = metal, Org = organic, Oxy = oxygen demand, PAH = polycyclic aromatic hydrocarbons, PCB = polychlorinated biphenyls, Pes = pesticide, Phy = physical, Sed = sediment, Sem = semi-volatile, Vol = volatile.

|  |  |  |  |  | **Detect** | | **Non-Detect** | |
| --- | --- | --- | --- | --- | --- | --- | --- | --- |
| **Group** | **Media** | **Code** | **Variable** | **Total** | **Count** | **PCT** | **Count** | **PCT** |
| Con | P | PWCHL | Chlorides | 1976 | 1973 | 99.9% | 3 | 0.2% |
| Con | P | PWTDS | TDS | 1987 | 1984 | 99.9% | 3 | 0.2% |
| Con | P | PWTSS | TSS | 1986 | 1040 | 52.4% | 946 | 47.6% |
| Con | P | PWTUR | Turbidity | 1953 | 1206 | 61.8% | 747 | 38.3% |
| Con | S | 00496 | Volatile Solids | 2410 | 2389 | 99.1% | 21 | 0.9% |
| Con | T | TSVOS | Volatile solids | 7 | 7 | 100.0% | 0 | 0.0% |
| Con | W | 00010 | Temperature | 4728 | 4728 | 100.0% | 0 | 0.0% |
| Con | W | 00076 | Turbidity | 2072 | 2065 | 99.7% | 7 | 0.3% |
| Con | W | 00300 | Oxygen, dissolved | 4704 | 4704 | 100.0% | 0 | 0.0% |
| Con | W | 00400 | pH | 4511 | 4511 | 100.0% | 0 | 0.0% |
| Con | W | 00480 | Salinity | 4726 | 4726 | 100.0% | 0 | 0.0% |
| Con | W | 00530 | TSS | 2074 | 2026 | 97.7% | 48 | 2.3% |
| Ino | P | P1004 | Nitrogen, Ammonia, | 1989 | 1809 | 91.0% | 180 | 9.1% |
| Ino | P | P1005 | Nitrate Nitrogen | 1989 | 696 | 35.0% | 1293 | 65.0% |
| Ino | P | P1006 | Nitrite Nitrogen | 1991 | 1229 | 61.7% | 762 | 38.3% |
| Ino | P | P1007 | Phosphorus, total | 1987 | 1966 | 98.9% | 21 | 1.1% |
| Ino | P | P1008 | Phosphate, Ortho as P | 1991 | 1869 | 93.9% | 122 | 6.1% |
| Ino | P | P1009 | Carbon, Organic, total | 1945 | 1932 | 99.3% | 13 | 0.7% |
| Ino | P | PWSUL | Sulfate | 1989 | 1981 | 99.6% | 8 | 0.4% |
| Ino | S | 00611 | Ammonia Nitrogen | 2500 | 2124 | 85.0% | 376 | 15.0% |
| Ino | S | 00616 | Nitrite-Nitrogen | 2416 | 778 | 32.2% | 1638 | 67.8% |
| Ino | S | 00747 | Acid Volatile Sulfide | 2496 | 1875 | 75.1% | 621 | 24.9% |
| Ino | S | 00943 | Chloride | 12 | 12 | 100.0% | 0 | 0.0% |
| Ino | S | SENO3 | Nitrate, sediment | 2423 | 501 | 20.7% | 1922 | 79.3% |
| Ino | T | TSAMM | Ammonia-N | 9 | 8 | 88.9% | 1 | 11.1% |
| Ino | T | TSAVS | Acid Volatile Sulfide | 2 | 2 | 100.0% | 0 | 0.0% |
| Ino | T | TSNAN | Nitrate+Nitrite-N | 2 | 1 | 50.0% | 1 | 50.0% |
| Ino | T | TSNO2 | Nitrite-N | 9 | 2 | 22.2% | 7 | 77.8% |
| Ino | T | TSSUL | Sulfide | 1 | 1 | 100.0% | 0 | 0.0% |
| Ino | T | TSTKN | TKN | 39 | 39 | 100.0% | 0 | 0.0% |
| Ino | W | 00610 | Ammonia Nitrogen | 2075 | 420 | 20.2% | 1655 | 79.8% |
| Ino | W | 00615 | Nitrite-Nitrogen | 2033 | 307 | 15.1% | 1726 | 84.9% |
| Ino | W | 00620 | Nitrate-Nitrogen | 2033 | 253 | 12.4% | 1780 | 87.6% |
| Ino | W | 00665 | Phosphorus, total | 2034 | 1807 | 88.8% | 227 | 11.2% |
| Ino | W | 00671 | Orthophosphate Phosphorus | 2038 | 1387 | 68.1% | 651 | 31.9% |
| Ino | W | 00940 | Chloride | 2037 | 2033 | 99.8% | 4 | 0.2% |
| Ino | W | 00945 | Sulfate | 2037 | 2026 | 99.5% | 11 | 0.5% |
| Ino | W | 89508 | Sulfide, Reactive | 6 | 5 | 83.3% | 1 | 16.7% |
| Met | P | P2001 | Aluminum | 1957 | 935 | 47.8% | 1022 | 52.2% |
| Met | P | P2002 | Antimony | 241 | 9 | 3.7% | 232 | 96.3% |
| Met | P | P2003 | Arsenic | 1959 | 724 | 37.0% | 1235 | 63.0% |
| Met | P | P2004 | Beryllium | 241 | 3 | 1.2% | 238 | 98.8% |
| Met | P | P2005 | Cadmium | 241 | 6 | 2.5% | 235 | 97.5% |
| Met | P | P2006 | Chromium | 1958 | 19 | 1.0% | 1939 | 99.0% |
| Met | P | P2007 | Chromium, VI | 1836 | 24 | 1.3% | 1812 | 98.7% |
| Met | P | P2008 | Copper | 1969 | 96 | 4.9% | 1873 | 95.1% |
| Met | P | P2009 | Iron | 9 | 8 | 88.9% | 1 | 11.1% |
| Met | P | P2010 | Lead | 1958 | 94 | 4.8% | 1864 | 95.2% |
| Met | P | P2011 | Mercury | 1956 | 42 | 2.2% | 1914 | 97.9% |
| Met | P | P2012 | Nickel | 1958 | 38 | 1.9% | 1920 | 98.1% |
| Met | P | P2013 | Selenium | 241 | 4 | 1.7% | 237 | 98.3% |
| Met | P | P2014 | Silver | 241 | 0 | 0.0% | 241 | 100.0% |
| Met | P | P2015 | Thallium | 241 | 3 | 1.2% | 238 | 98.8% |
| Met | P | P2016 | Zinc | 1963 | 835 | 42.5% | 1128 | 57.5% |
| Met | S | 01003 | Arsenic | 2558 | 2468 | 96.5% | 90 | 3.5% |
| Met | S | 01013 | Beryllium | 277 | 28 | 10.1% | 249 | 89.9% |
| Met | S | 01028 | Cadmium | 362 | 264 | 72.9% | 98 | 27.1% |
| Met | S | 01029 | Chromium | 2642 | 2616 | 99.0% | 26 | 1.0% |
| Met | S | 01043 | Copper | 2641 | 2607 | 98.7% | 34 | 1.3% |
| Met | S | 01052 | Lead | 2642 | 2594 | 98.2% | 48 | 1.8% |
| Met | S | 01068 | Nickel | 2642 | 2612 | 98.9% | 30 | 1.1% |
| Met | S | 01078 | Silver | 359 | 128 | 35.7% | 231 | 64.4% |
| Met | S | 01093 | Zinc | 2638 | 2632 | 99.8% | 6 | 0.2% |
| Met | S | 01098 | Antimony | 277 | 4 | 1.4% | 273 | 98.6% |
| Met | S | 01108 | Aluminum | 2639 | 2637 | 99.9% | 2 | 0.1% |
| Met | S | 01148 | Selenium | 277 | 140 | 50.5% | 137 | 49.5% |
| Met | S | 01170 | Iron | 2557 | 2553 | 99.8% | 4 | 0.2% |
| Met | S | 34480 | Thallium | 266 | 2 | 0.8% | 264 | 99.3% |
| Met | S | 71921 | Mercury | 2640 | 1805 | 68.4% | 835 | 31.6% |
| Met | S | SCRHX | Chromium, VI | 2080 | 59 | 2.8% | 2021 | 97.2% |
| Met | T | 01004 | Arsenic | 103 | 103 | 100.0% | 0 | 0.0% |
| Met | T | 01069 | Nickel, tissue | 353 | 15 | 4.3% | 338 | 95.8% |
| Met | T | 01073 | Thallium, tissue | 26 | 1 | 3.9% | 25 | 96.2% |
| Met | T | 01099 | Antimony, tissue | 32 | 0 | 0.0% | 32 | 100.0% |
| Met | T | 01149 | Selenium, tissue | 32 | 31 | 96.9% | 1 | 3.1% |
| Met | T | 34252 | Beryllium | 27 | 0 | 0.0% | 27 | 100.0% |
| Met | T | 34474 | Silver, tissue | 27 | 2 | 7.4% | 25 | 92.6% |
| Met | T | 71930 | Mercury, tissue | 353 | 199 | 56.4% | 154 | 43.6% |
| Met | T | 71936 | Lead, tissue | 353 | 30 | 8.5% | 323 | 91.5% |
| Met | T | 71937 | Copper, tissue | 342 | 273 | 79.8% | 69 | 20.2% |
| Met | T | 71938 | Zinc, tissue | 103 | 103 | 100.0% | 0 | 0.0% |
| Met | T | 71939 | Chromium, tissue | 103 | 63 | 61.2% | 40 | 38.8% |
| Met | T | 71940 | Cadmium, tissue | 27 | 17 | 63.0% | 10 | 37.0% |
| Met | T | 81666 | Aluminum, tissue | 353 | 320 | 90.7% | 33 | 9.4% |
| Met | T | TSCR6 | Cromium VI | 1 | 0 | 0.0% | 1 | 100.0% |
| Met | T | TSIRO | Iron, tissue | 13 | 13 | 100.0% | 0 | 0.0% |
| Met | W | 01000 | Arsenic, dissolved | 225 | 10 | 4.4% | 215 | 95.6% |
| Met | W | 01002 | Arsenic, total | 262 | 25 | 9.5% | 237 | 90.5% |
| Met | W | 01010 | Beryllium, dissolved | 225 | 0 | 0.0% | 225 | 100.0% |
| Met | W | 01012 | Beryllium, total | 258 | 0 | 0.0% | 258 | 100.0% |
| Met | W | 01025 | Cadmium, dissolved | 248 | 2 | 0.8% | 246 | 99.2% |
| Met | W | 01027 | Cadmium, total | 281 | 5 | 1.8% | 276 | 98.2% |
| Met | W | 01030 | Chromium, dissolved | 2010 | 9 | 0.5% | 2001 | 99.6% |
| Met | W | 01034 | Chromium, total | 1986 | 33 | 1.7% | 1953 | 98.3% |
| Met | W | 01040 | Copper, dissolved | 2012 | 17 | 0.8% | 1995 | 99.2% |
| Met | W | 01042 | Copper, total | 2023 | 57 | 2.8% | 1966 | 97.2% |
| Met | W | 01045 | Iron, total | 5 | 4 | 80.0% | 1 | 20.0% |
| Met | W | 01049 | Lead, dissolved | 1962 | 50 | 2.6% | 1912 | 97.5% |
| Met | W | 01051 | Lead, total | 2043 | 123 | 6.0% | 1920 | 94.0% |
| Met | W | 01057 | Thallium, dissolved | 225 | 0 | 0.0% | 225 | 100.0% |
| Met | W | 01059 | Thallium, total | 259 | 4 | 1.5% | 255 | 98.5% |
| Met | W | 01065 | Nickel, dissolved | 2008 | 7 | 0.4% | 2001 | 99.7% |
| Met | W | 01067 | Nickel, total | 2023 | 30 | 1.5% | 1993 | 98.5% |
| Met | W | 01075 | Silver, dissolved | 248 | 1 | 0.4% | 247 | 99.6% |
| Met | W | 01077 | Silver, total | 281 | 3 | 1.1% | 278 | 98.9% |
| Met | W | 01090 | Zinc, dissolved | 2009 | 196 | 9.8% | 1813 | 90.2% |
| Met | W | 01092 | Zinc, total | 2026 | 475 | 23.5% | 1551 | 76.6% |
| Met | W | 01095 | Antimony, dissolved | 225 | 0 | 0.0% | 225 | 100.0% |
| Met | W | 01097 | Antimony, total | 258 | 3 | 1.2% | 255 | 98.8% |
| Met | W | 01105 | Aluminum, total | 283 | 175 | 61.8% | 108 | 38.2% |
| Met | W | 01106 | Aluminum, dissolved | 248 | 45 | 18.2% | 203 | 81.9% |
| Met | W | 01145 | Selenium, dissolved | 225 | 0 | 0.0% | 225 | 100.0% |
| Met | W | 01147 | Selenium, total | 259 | 17 | 6.6% | 242 | 93.4% |
| Met | W | 01220 | Chromium VI, dissolved | 247 | 0 | 0.0% | 247 | 100.0% |
| Met | W | 71890 | Mercury, dissolved | 1990 | 10 | 0.5% | 1980 | 99.5% |
| Met | W | 71900 | Mercury, total | 303 | 2 | 0.7% | 301 | 99.3% |
| Org | P | PWCYN | Cyanide | 1989 | 39 | 2.0% | 1950 | 98.0% |
| Org | P | PWOAG | Oil and Grease | 1970 | 194 | 9.9% | 1776 | 90.2% |
| Org | P | PWORN | Organic Nitrogen | 1987 | 1668 | 84.0% | 319 | 16.1% |
| Org | S | 00561 | Oil & Grease | 2488 | 731 | 29.4% | 1757 | 70.6% |
| Org | S | 00626 | Nitrogen, Organic, Kjeldahl | 2424 | 2367 | 97.7% | 57 | 2.4% |
| Org | S | 00721 | Cyanide | 2418 | 11 | 0.5% | 2407 | 99.6% |
| Org | S | 81951 | Total Organic Carbon | 2496 | 2496 | 100.0% | 0 | 0.0% |
| Org | T | TSCYN | Cyanide, tissue | 2 | 1 | 50.0% | 1 | 50.0% |
| Org | T | TSOAG | Oil and Grease | 2 | 2 | 100.0% | 0 | 0.0% |
| Org | T | TSTOC | Total Organic Carbon, tissue | 2 | 2 | 100.0% | 0 | 0.0% |
| Org | W | 00556 | Oil & Grease | 2047 | 230 | 11.2% | 1817 | 88.8% |
| Org | W | 00605 | Organic Nitrogen | 2031 | 1751 | 86.2% | 280 | 13.8% |
| Org | W | 00680 | Total Organic Carbon | 2070 | 2034 | 98.3% | 36 | 1.7% |
| Org | W | 00720 | Cyanide | 2031 | 65 | 3.2% | 1966 | 96.8% |
| Oxy | P | PWBOD | BOD | 1986 | 528 | 26.6% | 1458 | 73.4% |
| Oxy | P | PWCOD | COD | 1989 | 1965 | 98.8% | 24 | 1.2% |
| Oxy | W | 00310 | BOD | 2075 | 724 | 34.9% | 1351 | 65.1% |
| Oxy | W | 00335 | COD | 2078 | 2073 | 99.8% | 5 | 0.2% |
| PAH | P | P6001 | Acenaphthene | 1925 | 1 | 0.1% | 1924 | 100.0% |
| PAH | P | P6002 | Acenaphthylene | 1925 | 1 | 0.1% | 1924 | 100.0% |
| PAH | P | P6003 | Anthracene | 1925 | 1 | 0.1% | 1924 | 100.0% |
| PAH | P | P6004 | Benzo(A)Anthracene | 1925 | 1 | 0.1% | 1924 | 100.0% |
| PAH | P | P6005 | Benzo(K)Fluoranthene | 1811 | 0 | 0.0% | 1811 | 100.0% |
| PAH | P | P6006 | Benzo(B)Fluoranthene | 1820 | 0 | 0.0% | 1820 | 100.0% |
| PAH | P | P6007 | Benzo(GHI)Perylene | 221 | 0 | 0.0% | 221 | 100.0% |
| PAH | P | P6008 | Benzo(A)Pyrene | 1925 | 1 | 0.1% | 1924 | 100.0% |
| PAH | P | P6009 | Chrysene | 1925 | 1 | 0.1% | 1924 | 100.0% |
| PAH | P | P6010 | Dibenzo(AH)Anthracence | 221 | 0 | 0.0% | 221 | 100.0% |
| PAH | P | P6011 | Fluoranthene | 1925 | 1 | 0.1% | 1924 | 100.0% |
| PAH | P | P6012 | Fluorene | 1925 | 1 | 0.1% | 1924 | 100.0% |
| PAH | P | P6013 | Indeno(1,2,3-CD)Pyrene | 221 | 0 | 0.0% | 221 | 100.0% |
| PAH | P | P6014 | Naphthalene | 1927 | 1 | 0.1% | 1926 | 100.0% |
| PAH | P | P6015 | Phenanthrene | 1927 | 1 | 0.1% | 1926 | 100.0% |
| PAH | P | P6016 | Pyrene | 1920 | 1 | 0.1% | 1919 | 100.0% |
| PAH | P | PBBKF | Benzo(B&K)Fluoranthene | 87 | 1 | 1.2% | 86 | 98.9% |
| PAH | S | 34203 | Acenaphthylene | 2289 | 1 | 0.0% | 2288 | 100.0% |
| PAH | S | 34208 | Acenaphthene | 2289 | 1 | 0.0% | 2288 | 100.0% |
| PAH | S | 34223 | Anthracene | 2289 | 1 | 0.0% | 2288 | 100.0% |
| PAH | S | 34233 | Benzo(B)Fluoranthene | 2197 | 1 | 0.1% | 2196 | 100.0% |
| PAH | S | 34245 | Benzo(k)Fluoranthene | 2224 | 1 | 0.0% | 2223 | 100.0% |
| PAH | S | 34250 | Benzo(a)Pyrene | 2289 | 2 | 0.1% | 2287 | 99.9% |
| PAH | S | 34323 | Chrysene | 2288 | 1 | 0.0% | 2287 | 100.0% |
| PAH | S | 34379 | Fluoranthene | 2291 | 1 | 0.0% | 2290 | 100.0% |
| PAH | S | 34384 | Fluorene | 2291 | 1 | 0.0% | 2290 | 100.0% |
| PAH | S | 34406 | Indeno(1,2,3-CD)Pyrene | 242 | 1 | 0.4% | 241 | 99.6% |
| PAH | S | 34445 | Naphthalene | 2289 | 1 | 0.0% | 2288 | 100.0% |
| PAH | S | 34464 | Phenanthrene | 2288 | 1 | 0.0% | 2287 | 100.0% |
| PAH | S | 34472 | Pyrene | 2284 | 1 | 0.0% | 2283 | 100.0% |
| PAH | S | 34524 | Benzo(GHI)Perylene1,12-Benzoperylen | 241 | 1 | 0.4% | 240 | 99.6% |
| PAH | S | 34529 | Benzo(A)Anthracene1,2-Benzanthracene | 2288 | 1 | 0.0% | 2287 | 100.0% |
| PAH | S | 34559 | 1,2,5,6-Dibenzanthracene | 240 | 1 | 0.4% | 239 | 99.6% |
| PAH | S | SBBKF | Benzo(b&k)fluoranthene | 105 | 0 | 0.0% | 105 | 100.0% |
| PAH | S | SEPAH | PAH's, total | 2393 | 3 | 0.1% | 2390 | 99.9% |
| PAH | T | 34204 | Acenaphthylene, tissue | 349 | 0 | 0.0% | 349 | 100.0% |
| PAH | T | 34209 | Acenaphthene, tissue | 349 | 0 | 0.0% | 349 | 100.0% |
| PAH | T | 34224 | Anthracene, tissue | 349 | 0 | 0.0% | 349 | 100.0% |
| PAH | T | 34234 | Benzo(B)Fluoranthene, tissue | 321 | 0 | 0.0% | 321 | 100.0% |
| PAH | T | 34246 | Benzo(k)fluoranthene, tissue | 322 | 0 | 0.0% | 322 | 100.0% |
| PAH | T | 34251 | Benzo(a)pyrene, tissue | 349 | 0 | 0.0% | 349 | 100.0% |
| PAH | T | 34324 | Chrysene, tissue | 349 | 0 | 0.0% | 349 | 100.0% |
| PAH | T | 34380 | Fluoranthene, tissue | 349 | 0 | 0.0% | 349 | 100.0% |
| PAH | T | 34385 | Fluorene, tissue | 349 | 0 | 0.0% | 349 | 100.0% |
| PAH | T | 34407 | Indeno(1,2,3-CD)Pyrene, tissue | 26 | 0 | 0.0% | 26 | 100.0% |
| PAH | T | 34446 | Naphthalene, tissue | 349 | 0 | 0.0% | 349 | 100.0% |
| PAH | T | 34465 | Phenanthrene, tissue | 349 | 1 | 0.3% | 348 | 99.7% |
| PAH | T | 34473 | Pyrene, tissue | 339 | 0 | 0.0% | 339 | 100.0% |
| PAH | T | 34525 | Benzo(GHI)Perylene1,12-Benzoperylen, tissue | 26 | 0 | 0.0% | 26 | 100.0% |
| PAH | T | 34530 | Benzo(A)Anthracene1,2-Benzanthracene, tissue | 349 | 0 | 0.0% | 349 | 100.0% |
| PAH | T | 79040 | Dibenz(A,H)Anthracene, tissue | 26 | 0 | 0.0% | 26 | 100.0% |
| PAH | T | TBBKF | Benzo(bk)fluoranthene, tissue | 22 | 0 | 0.0% | 22 | 100.0% |
| PAH | W | 34200 | Acenaphthylene, total | 1957 | 0 | 0.0% | 1957 | 100.0% |
| PAH | W | 34205 | Acenaphthene, total | 1958 | 0 | 0.0% | 1958 | 100.0% |
| PAH | W | 34220 | Anthracene | 1958 | 0 | 0.0% | 1958 | 100.0% |
| PAH | W | 34230 | Benzo(b)fluoranthene, total | 1868 | 0 | 0.0% | 1868 | 100.0% |
| PAH | W | 34242 | Benzo(k)fluoranthene | 1868 | 0 | 0.0% | 1868 | 100.0% |
| PAH | W | 34247 | Benzo(a)pyrene | 1957 | 0 | 0.0% | 1957 | 100.0% |
| PAH | W | 34320 | Chrysene, total | 1957 | 0 | 0.0% | 1957 | 100.0% |
| PAH | W | 34376 | Fluoranthene, total | 1958 | 0 | 0.0% | 1958 | 100.0% |
| PAH | W | 34381 | Fluorene, total | 1958 | 0 | 0.0% | 1958 | 100.0% |
| PAH | W | 34403 | Indeno(1,2,3-CD)Pyrene | 238 | 0 | 0.0% | 238 | 100.0% |
| PAH | W | 34461 | Phenanthrene, total | 1958 | 0 | 0.0% | 1958 | 100.0% |
| PAH | W | 34469 | Pyrene, total | 1958 | 0 | 0.0% | 1958 | 100.0% |
| PAH | W | 34521 | Benzo(GHI)Perylene1,12-Benzoperylene, total | 237 | 0 | 0.0% | 237 | 100.0% |
| PAH | W | 34526 | Benzo(A)anthracene, total | 1958 | 0 | 0.0% | 1958 | 100.0% |
| PAH | W | 34556 | 1,2,5,6-Dibenzanthracene, total | 237 | 0 | 0.0% | 237 | 100.0% |
| PAH | W | 34696 | Naphthalene, total | 1958 | 0 | 0.0% | 1958 | 100.0% |
| PAH | W | WBBKF | Benzo(bk)fluoranthene | 87 | 0 | 0.0% | 87 | 100.0% |
| PCB | P | P3501 | PCBs, total | 9 | 0 | 0.0% | 9 | 100.0% |
| PCB | P | P3502 | PCB-1016 | 211 | 0 | 0.0% | 211 | 100.0% |
| PCB | P | P3503 | PCB-1221 | 211 | 0 | 0.0% | 211 | 100.0% |
| PCB | P | P3504 | PCB-1232 | 211 | 0 | 0.0% | 211 | 100.0% |
| PCB | P | P3505 | PCB-1242 | 211 | 0 | 0.0% | 211 | 100.0% |
| PCB | P | P3506 | PCB-1248 | 211 | 0 | 0.0% | 211 | 100.0% |
| PCB | P | P3507 | PCB-1254 | 211 | 0 | 0.0% | 211 | 100.0% |
| PCB | P | P3508 | PCB-1260 | 211 | 0 | 0.0% | 211 | 100.0% |
| PCB | S | 39491 | PCB-1221 | 77 | 0 | 0.0% | 77 | 100.0% |
| PCB | S | 39495 | PCB-1232 | 77 | 0 | 0.0% | 77 | 100.0% |
| PCB | S | 39499 | PCB-1242 | 77 | 0 | 0.0% | 77 | 100.0% |
| PCB | S | 39503 | PCB-1248 | 77 | 0 | 0.0% | 77 | 100.0% |
| PCB | S | 39507 | PCB-1254 | 77 | 0 | 0.0% | 77 | 100.0% |
| PCB | S | 39511 | PCB-1260 | 76 | 0 | 0.0% | 76 | 100.0% |
| PCB | S | 39514 | PCB-1016 | 77 | 0 | 0.0% | 77 | 100.0% |
| PCB | S | 39519 | PCBs, total | 6 | 1 | 16.7% | 5 | 83.3% |
| PCB | T | 34664 | PCB-1221, tissue | 23 | 0 | 0.0% | 23 | 100.0% |
| PCB | T | 34667 | PCB-1232, tissue | 23 | 0 | 0.0% | 23 | 100.0% |
| PCB | T | 34669 | PCB-1248, tissue | 23 | 0 | 0.0% | 23 | 100.0% |
| PCB | T | 34670 | PCB-1260, tissue | 23 | 0 | 0.0% | 23 | 100.0% |
| PCB | T | 34674 | PCB-1016, tissue | 23 | 0 | 0.0% | 23 | 100.0% |
| PCB | T | 34689 | PCB-1242, tissue | 23 | 0 | 0.0% | 23 | 100.0% |
| PCB | T | 34690 | PCB-1254, tissue | 23 | 0 | 0.0% | 23 | 100.0% |
| PCB | T | 39515 | PCBs, tissue | 8 | 6 | 75.0% | 2 | 25.0% |
| PCB | W | 34671 | Aroclar-1016 | 291 | 0 | 0.0% | 291 | 100.0% |
| PCB | W | 39488 | PCB-1221 | 291 | 0 | 0.0% | 291 | 100.0% |
| PCB | W | 39492 | PCB-1232 | 291 | 0 | 0.0% | 291 | 100.0% |
| PCB | W | 39496 | PCB-1242 | 291 | 0 | 0.0% | 291 | 100.0% |
| PCB | W | 39500 | PCB-1248 | 291 | 0 | 0.0% | 291 | 100.0% |
| PCB | W | 39504 | PCB-1254 | 291 | 0 | 0.0% | 291 | 100.0% |
| PCB | W | 39508 | PCB-1260 | 291 | 0 | 0.0% | 291 | 100.0% |
| PCB | W | 39516 | PCBs, total | 10 | 0 | 0.0% | 10 | 100.0% |
| Pes | P | P3001 | Aldrin | 222 | 0 | 0.0% | 222 | 100.0% |
| Pes | P | P3002 | Alpha Benzene Hexachloride | 222 | 0 | 0.0% | 222 | 100.0% |
| Pes | P | P3003 | Beta Benzene Hexachloride | 222 | 0 | 0.0% | 222 | 100.0% |
| Pes | P | P3004 | Lindane | 213 | 0 | 0.0% | 213 | 100.0% |
| Pes | P | P3005 | Delta Benzene Hexachloride | 222 | 0 | 0.0% | 222 | 100.0% |
| Pes | P | P3006 | Chlordane, total | 210 | 0 | 0.0% | 210 | 100.0% |
| Pes | P | P3007 | DDD, total | 222 | 0 | 0.0% | 222 | 100.0% |
| Pes | P | P3008 | DDE, total | 222 | 0 | 0.0% | 222 | 100.0% |
| Pes | P | P3009 | DDD, total | 222 | 0 | 0.0% | 222 | 100.0% |
| Pes | P | P3011 | Dieldrin | 222 | 0 | 0.0% | 222 | 100.0% |
| Pes | P | P3012 | Endosulfan | 222 | 0 | 0.0% | 222 | 100.0% |
| Pes | P | P3013 | Endosulfan-II(Beta) | 222 | 0 | 0.0% | 222 | 100.0% |
| Pes | P | P3014 | Endosulfan Sulfate | 222 | 0 | 0.0% | 222 | 100.0% |
| Pes | P | P3015 | Endrin | 222 | 0 | 0.0% | 222 | 100.0% |
| Pes | P | P3016 | Endrin Aldehyde | 222 | 0 | 0.0% | 222 | 100.0% |
| Pes | P | P3017 | Heptachlor | 222 | 0 | 0.0% | 222 | 100.0% |
| Pes | P | P3018 | Heptachlor Epoxide | 222 | 0 | 0.0% | 222 | 100.0% |
| Pes | P | P3019 | Toxaphene | 221 | 0 | 0.0% | 221 | 100.0% |
| Pes | P | P5038 | Pentachlorophenol | 221 | 0 | 0.0% | 221 | 100.0% |
| Pes | S | 34213 | Acrolein | 255 | 0 | 0.0% | 255 | 100.0% |
| Pes | S | 34354 | Endosulfan Sulfate | 257 | 0 | 0.0% | 257 | 100.0% |
| Pes | S | 34359 | Endosulfan-II(Beta) | 257 | 0 | 0.0% | 257 | 100.0% |
| Pes | S | 34369 | Endrin Aldehyde | 257 | 0 | 0.0% | 257 | 100.0% |
| Pes | S | 34411 | Isophorone | 241 | 0 | 0.0% | 241 | 100.0% |
| Pes | S | 34660 | DNOC(4,6-Dinitro-Ortho-Cresol) | 2309 | 0 | 0.0% | 2309 | 100.0% |
| Pes | S | 39061 | Pentachlorophenol | 241 | 0 | 0.0% | 241 | 100.0% |
| Pes | S | 39076 | BHC-Alpha | 257 | 0 | 0.0% | 257 | 100.0% |
| Pes | S | 39333 | Aldrin | 257 | 0 | 0.0% | 257 | 100.0% |
| Pes | S | 39351 | Chlordane | 244 | 0 | 0.0% | 244 | 100.0% |
| Pes | S | 39363 | DDD | 257 | 0 | 0.0% | 257 | 100.0% |
| Pes | S | 39368 | DDE | 257 | 0 | 0.0% | 257 | 100.0% |
| Pes | S | 39373 | DDD | 257 | 0 | 0.0% | 257 | 100.0% |
| Pes | S | 39383 | Dieldrin | 257 | 0 | 0.0% | 257 | 100.0% |
| Pes | S | 39389 | Endosulfan | 257 | 0 | 0.0% | 257 | 100.0% |
| Pes | S | 39393 | Endrin | 257 | 0 | 0.0% | 257 | 100.0% |
| Pes | S | 39403 | Toxaphene | 257 | 0 | 0.0% | 257 | 100.0% |
| Pes | S | 39413 | Heptachlor | 257 | 0 | 0.0% | 257 | 100.0% |
| Pes | S | 39423 | Heptachlor Epoxide | 257 | 0 | 0.0% | 257 | 100.0% |
| Pes | S | 39481 | Methoxychlor | 10 | 5 | 50.0% | 5 | 50.0% |
| Pes | S | 39783 | BHC-Gamma, Lindane | 257 | 0 | 0.0% | 257 | 100.0% |
| Pes | S | 46290 | BHC-Beta | 257 | 0 | 0.0% | 257 | 100.0% |
| Pes | S | 46292 | BHC-Delta | 257 | 0 | 0.0% | 257 | 100.0% |
| Pes | S | 79749 | 1,2-Dibromomethane, EDB | 1 | 0 | 0.0% | 1 | 100.0% |
| Pes | T | 20284 | Trans Nanochlor | 10 | 0 | 0.0% | 10 | 100.0% |
| Pes | T | 34214 | Acrolein, tissue | 25 | 0 | 0.0% | 25 | 100.0% |
| Pes | T | 34258 | B-BHC-Beta, tissue | 25 | 0 | 0.0% | 25 | 100.0% |
| Pes | T | 34263 | BHC-Delta, tissue | 25 | 0 | 0.0% | 25 | 100.0% |
| Pes | T | 34355 | Endosulfan Sulfate, tissue | 25 | 0 | 0.0% | 25 | 100.0% |
| Pes | T | 34360 | Endosulfan II(Beta), tissue | 25 | 0 | 0.0% | 25 | 100.0% |
| Pes | T | 34370 | Endrin Aldehyde, tissue | 25 | 0 | 0.0% | 25 | 100.0% |
| Pes | T | 34412 | Isophorone, tissue | 25 | 0 | 0.0% | 25 | 100.0% |
| Pes | T | 34661 | DNOC(4,6-Dinitro-Ortho-Cresol), tissue | 344 | 0 | 0.0% | 344 | 100.0% |
| Pes | T | 34680 | Aldrin, tissue | 25 | 0 | 0.0% | 25 | 100.0% |
| Pes | T | 34682 | Chlordane, tissue | 23 | 0 | 0.0% | 23 | 100.0% |
| Pes | T | 34685 | Endrin, tissue | 25 | 0 | 0.0% | 25 | 100.0% |
| Pes | T | 34686 | Heptachlor Epoxide, tissue | 25 | 0 | 0.0% | 25 | 100.0% |
| Pes | T | 34687 | Heptachlor, tissue | 25 | 0 | 0.0% | 25 | 100.0% |
| Pes | T | 34691 | Toxaphene, tissue | 25 | 0 | 0.0% | 25 | 100.0% |
| Pes | T | 39060 | Pentachlorophenol, tissue | 25 | 0 | 0.0% | 25 | 100.0% |
| Pes | T | 39074 | BHC-Alpha, tissue | 25 | 0 | 0.0% | 25 | 100.0% |
| Pes | T | 39075 | BHC-Gamma, tissue | 24 | 0 | 0.0% | 24 | 100.0% |
| Pes | T | 39376 | DDT, total, tissue | 25 | 0 | 0.0% | 25 | 100.0% |
| Pes | T | 39406 | Dieldrin, tissue | 25 | 0 | 0.0% | 25 | 100.0% |
| Pes | T | 81759 | Endosulfan, tissue | 25 | 0 | 0.0% | 25 | 100.0% |
| Pes | T | 81896 | DDE, tissue | 25 | 0 | 0.0% | 25 | 100.0% |
| Pes | T | 81897 | DDD, tissue | 25 | 0 | 0.0% | 25 | 100.0% |
| Pes | W | 34210 | Acrolein, total | 237 | 0 | 0.0% | 237 | 100.0% |
| Pes | W | 34259 | BHC-Delta | 236 | 0 | 0.0% | 236 | 100.0% |
| Pes | W | 34351 | Endosulfan Sulfate | 236 | 0 | 0.0% | 236 | 100.0% |
| Pes | W | 34356 | Endosulfan-II(Beta) | 236 | 0 | 0.0% | 236 | 100.0% |
| Pes | W | 34366 | Endrin Aldehyde | 236 | 0 | 0.0% | 236 | 100.0% |
| Pes | W | 34657 | DNOC(4,6-Dinitro-Ortho-Cresol), total | 1958 | 0 | 0.0% | 1958 | 100.0% |
| Pes | W | 39032 | Pentachlorophenol | 238 | 0 | 0.0% | 238 | 100.0% |
| Pes | W | 39330 | Aldrin | 236 | 0 | 0.0% | 236 | 100.0% |
| Pes | W | 39337 | Alpha Benzene Hexachloride | 236 | 0 | 0.0% | 236 | 100.0% |
| Pes | W | 39338 | Beta Benzene Hexachloride | 236 | 0 | 0.0% | 236 | 100.0% |
| Pes | W | 39350 | Chlordane, total | 222 | 0 | 0.0% | 222 | 100.0% |
| Pes | W | 39360 | p,p' -DDD, total | 236 | 0 | 0.0% | 236 | 100.0% |
| Pes | W | 39365 | p,p'-DDE, total | 236 | 0 | 0.0% | 236 | 100.0% |
| Pes | W | 39370 | p,p'-DDT, total | 236 | 0 | 0.0% | 236 | 100.0% |
| Pes | W | 39380 | Dieldrin, total | 236 | 0 | 0.0% | 236 | 100.0% |
| Pes | W | 39388 | Endosulfan, total | 236 | 0 | 0.0% | 236 | 100.0% |
| Pes | W | 39390 | Endrin | 236 | 0 | 0.0% | 236 | 100.0% |
| Pes | W | 39400 | Toxaphene, total | 236 | 0 | 0.0% | 236 | 100.0% |
| Pes | W | 39410 | Heptachlor | 236 | 0 | 0.0% | 236 | 100.0% |
| Pes | W | 39420 | Heptachlor Epoxide | 236 | 0 | 0.0% | 236 | 100.0% |
| Pes | W | 39782 | BHC-Gamma, Lindane | 236 | 0 | 0.0% | 236 | 100.0% |
| Phy | W | 00094 | Specific Conductance, Field | 4591 | 4591 | 100.0% | 0 | 0.0% |
| Phy | W | 70300 | Total Dissolved Solids | 2033 | 2032 | 100.0% | 1 | 0.1% |
| Sed | S | 82008 | Sediment Particle Size Class, 0039-.0625, Silt | 2111 | 2111 | 100.0% | 0 | 0.0% |
| Sed | S | 82009 | Sediment Particle Size Class, <.0039, Clay | 2111 | 2111 | 100.0% | 0 | 0.0% |
| Sed | S | 89991 | Sediment Prctl.Size Class, Sand .0625-2mm | 2111 | 2111 | 100.0% | 0 | 0.0% |
| Sem | P | P5001 | Benzidine | 222 | 0 | 0.0% | 222 | 100.0% |
| Sem | P | P5002 | BIS(2-Chloroethoxy)Methane | 222 | 0 | 0.0% | 222 | 100.0% |
| Sem | P | P5003 | BIS(2-Chloroethyl)Ether | 222 | 0 | 0.0% | 222 | 100.0% |
| Sem | P | P5004 | BIS(2-Chloroisopropyl)Ether | 222 | 0 | 0.0% | 222 | 100.0% |
| Sem | P | P5005 | BIS(2-Ethylhexyl)Phthalate | 1896 | 102 | 5.4% | 1794 | 94.6% |
| Sem | P | P5006 | 4-Bromophenylphenyl Ether | 221 | 0 | 0.0% | 221 | 100.0% |
| Sem | P | P5007 | Butylbenzyl Phthalate | 1925 | 4 | 0.2% | 1921 | 99.8% |
| Sem | P | P5008 | 3-Methyl-4-Chlorophenol | 1925 | 0 | 0.0% | 1925 | 100.0% |
| Sem | P | P5009 | 2-Chloronaphthalene | 221 | 0 | 0.0% | 221 | 100.0% |
| Sem | P | P5010 | 2-Chlorophenol | 1925 | 1 | 0.1% | 1924 | 100.0% |
| Sem | P | P5011 | 4-Chlororphenylphenyl Ether | 221 | 0 | 0.0% | 221 | 100.0% |
| Sem | P | P5012 | Di-N-Butl Phthalate | 1925 | 32 | 1.7% | 1893 | 98.3% |
| Sem | P | P5013 | 1,2-Dichlorobenzene | 1925 | 1 | 0.1% | 1924 | 100.0% |
| Sem | P | P5014 | 1,3-Dichlorobenzene | 1925 | 1 | 0.1% | 1924 | 100.0% |
| Sem | P | P5015 | 1,4-Dichlorobenzene | 1925 | 1 | 0.1% | 1924 | 100.0% |
| Sem | P | P5016 | 3,3-Dichlorobenzidine | 221 | 0 | 0.0% | 221 | 100.0% |
| Sem | P | P5017 | 2,4-Dichlorophenol | 1925 | 1 | 0.1% | 1924 | 100.0% |
| Sem | P | P5018 | Diethyl Phthalate | 1925 | 4 | 0.2% | 1921 | 99.8% |
| Sem | P | P5019 | 2,4-Dimethylphenol | 1927 | 1 | 0.1% | 1926 | 100.0% |
| Sem | P | P5020 | Dimethyl Phthalate | 1925 | 1 | 0.1% | 1924 | 100.0% |
| Sem | P | P5021 | 4,6-Dinitro-2-Methylphenol | 1896 | 1 | 0.1% | 1895 | 100.0% |
| Sem | P | P5022 | 2,4-Dinitrophenol | 1927 | 1 | 0.1% | 1926 | 100.0% |
| Sem | P | P5023 | 2,4-Dinitrotoluene | 1925 | 1 | 0.1% | 1924 | 100.0% |
| Sem | P | P5024 | 2,6-Dinitrotoluene | 1925 | 1 | 0.1% | 1924 | 100.0% |
| Sem | P | P5025 | Di-N-Octyl Phthalate | 221 | 0 | 0.0% | 221 | 100.0% |
| Sem | P | P5026 | 1,2-Diphenylhydrazine | 221 | 0 | 0.0% | 221 | 100.0% |
| Sem | P | P5027 | Hexachlorobenzene | 1925 | 1 | 0.1% | 1924 | 100.0% |
| Sem | P | P5028 | Hexachlorobutadiene | 1925 | 1 | 0.1% | 1924 | 100.0% |
| Sem | P | P5029 | Hexachlorocyclopentadiene | 221 | 0 | 0.0% | 221 | 100.0% |
| Sem | P | P5030 | Hexachloroethane | 1925 | 1 | 0.1% | 1924 | 100.0% |
| Sem | P | P5031 | Isophorone | 221 | 0 | 0.0% | 221 | 100.0% |
| Sem | P | P5032 | Nitrobenzene | 1925 | 1 | 0.1% | 1924 | 100.0% |
| Sem | P | P5033 | 2-Nitrophenol | 1950 | 1 | 0.1% | 1949 | 100.0% |
| Sem | P | P5034 | 4-Nitrophenol | 1925 | 1 | 0.1% | 1924 | 100.0% |
| Sem | P | P5035 | N-Nitrosodimethylamine | 221 | 0 | 0.0% | 221 | 100.0% |
| Sem | P | P5036 | N-Nitrosodiphenylamine | 221 | 0 | 0.0% | 221 | 100.0% |
| Sem | P | P5037 | N-Nitrosodi-N-Propylamine | 221 | 0 | 0.0% | 221 | 100.0% |
| Sem | P | P5039 | Phenol | 1924 | 3 | 0.2% | 1921 | 99.8% |
| Sem | P | P5040 | 1,2,4-Trichlorobenzene | 1925 | 1 | 0.1% | 1924 | 100.0% |
| Sem | P | P5041 | 2,4,6-Trichlorophenol | 221 | 0 | 0.0% | 221 | 100.0% |
| Sem | S | 34276 | Bis(2-Chloroethyl)Ether | 240 | 0 | 0.0% | 240 | 100.0% |
| Sem | S | 34281 | Bis(2-Chloroethoxy)Methane | 240 | 0 | 0.0% | 240 | 100.0% |
| Sem | S | 34286 | Bis(2-Chloroisopropyl)Ether | 240 | 0 | 0.0% | 240 | 100.0% |
| Sem | S | 34295 | N-Butyl Benzyl Phthalate | 2307 | 2 | 0.1% | 2305 | 99.9% |
| Sem | S | 34339 | Diethyl Phthalate | 2308 | 0 | 0.0% | 2308 | 100.0% |
| Sem | S | 34344 | Dimethyl Phthalate | 2308 | 17 | 0.7% | 2291 | 99.3% |
| Sem | S | 34349 | 1,2-Diphenylhydrazine | 241 | 0 | 0.0% | 241 | 100.0% |
| Sem | S | 34389 | Hexachlorocyclopentadiene | 241 | 0 | 0.0% | 241 | 100.0% |
| Sem | S | 34399 | Hexachloroethane | 2309 | 0 | 0.0% | 2309 | 100.0% |
| Sem | S | 34431 | N-Nitrosodi-N-Propylamine | 241 | 0 | 0.0% | 241 | 100.0% |
| Sem | S | 34436 | N-Nitrosodiphenylamine | 241 | 0 | 0.0% | 241 | 100.0% |
| Sem | S | 34441 | N-Nitrosodimethylamine | 241 | 0 | 0.0% | 241 | 100.0% |
| Sem | S | 34450 | Nitrobenzene | 2199 | 80 | 3.6% | 2119 | 96.4% |
| Sem | S | 34455 | Parachlorometa Cresol | 2308 | 1 | 0.0% | 2307 | 100.0% |
| Sem | S | 34539 | 1,2-Dichlorobenzene | 2309 | 0 | 0.0% | 2309 | 100.0% |
| Sem | S | 34554 | 1,2,4-Trichlorobenzene | 2310 | 0 | 0.0% | 2310 | 100.0% |
| Sem | S | 34569 | 1,3-Dichlorobenzene | 2309 | 0 | 0.0% | 2309 | 100.0% |
| Sem | S | 34574 | 1,4-Dichlorobenzene | 2284 | 0 | 0.0% | 2284 | 100.0% |
| Sem | S | 34584 | 2-Chloronaphthalene | 241 | 0 | 0.0% | 241 | 100.0% |
| Sem | S | 34589 | 2-Chlorophenol | 2309 | 0 | 0.0% | 2309 | 100.0% |
| Sem | S | 34594 | 2-Nitrophenol | 2284 | 0 | 0.0% | 2284 | 100.0% |
| Sem | S | 34599 | Di-N-Octyl Phthalate | 240 | 0 | 0.0% | 240 | 100.0% |
| Sem | S | 34604 | 2,4-Dichlorophenol | 2309 | 0 | 0.0% | 2309 | 100.0% |
| Sem | S | 34609 | 2,4-Dimethylphenol | 2309 | 0 | 0.0% | 2309 | 100.0% |
| Sem | S | 34614 | 2,4-Dinitrotoluene | 2311 | 0 | 0.0% | 2311 | 100.0% |
| Sem | S | 34619 | 2,4-Dinitrophenol | 2309 | 0 | 0.0% | 2309 | 100.0% |
| Sem | S | 34624 | 2,4,6-Trichlorophenol | 241 | 0 | 0.0% | 241 | 100.0% |
| Sem | S | 34629 | 2,6-Dinitrotoluene | 2309 | 0 | 0.0% | 2309 | 100.0% |
| Sem | S | 34634 | 3,3'-Dichlorobenzidine | 241 | 1 | 0.4% | 240 | 99.6% |
| Sem | S | 34639 | 4-Bromophenyl Phenyl Ether | 241 | 0 | 0.0% | 241 | 100.0% |
| Sem | S | 34644 | 4-Chlorophenyl Phenyl Ether | 241 | 0 | 0.0% | 241 | 100.0% |
| Sem | S | 34649 | 4-Nitrophenol | 2309 | 0 | 0.0% | 2309 | 100.0% |
| Sem | S | 34695 | Phenol | 2305 | 0 | 0.0% | 2305 | 100.0% |
| Sem | S | 39102 | BIS(2-Ethylhexyl) Phthalate | 2307 | 17 | 0.7% | 2290 | 99.3% |
| Sem | S | 39112 | Di-N-Butyl Phthalate | 2310 | 16 | 0.7% | 2294 | 99.3% |
| Sem | S | 39121 | Benzidine | 241 | 0 | 0.0% | 241 | 100.0% |
| Sem | S | 39701 | Hexachlorobenzene | 2309 | 0 | 0.0% | 2309 | 100.0% |
| Sem | S | 39705 | Hexachlorobutadiene | 2309 | 0 | 0.0% | 2309 | 100.0% |
| Sem | T | 34241 | Benzidine, tissue | 25 | 0 | 0.0% | 25 | 100.0% |
| Sem | T | 34277 | Bis(2-Chloroethyl)Ether, tissue | 25 | 0 | 0.0% | 25 | 100.0% |
| Sem | T | 34282 | Bis(2-Chloroethoxy)Methane, tissue | 25 | 0 | 0.0% | 25 | 100.0% |
| Sem | T | 34287 | Bis(2-Chloroisopropyl)Ether, tissue | 42 | 0 | 0.0% | 42 | 100.0% |
| Sem | T | 34296 | N-Butyl Benzyl Phthalate, tissue | 343 | 0 | 0.0% | 343 | 100.0% |
| Sem | T | 34340 | Diethyl Phthalate, tissue | 344 | 0 | 0.0% | 344 | 100.0% |
| Sem | T | 34345 | Dimethyl Phthalate, tissue | 344 | 8 | 2.3% | 336 | 97.7% |
| Sem | T | 34350 | 1,2-Diphenylhydrazine, tissue | 25 | 0 | 0.0% | 25 | 100.0% |
| Sem | T | 34390 | Hexachlorocyclopentadiene, tissue | 25 | 0 | 0.0% | 25 | 100.0% |
| Sem | T | 34395 | Hexachlorobutadiene, tissue | 348 | 0 | 0.0% | 348 | 100.0% |
| Sem | T | 34400 | Hexachloroethane, tissue | 348 | 0 | 0.0% | 348 | 100.0% |
| Sem | T | 34432 | N-Nitrosodi-N-Propylamine, tissue | 25 | 0 | 0.0% | 25 | 100.0% |
| Sem | T | 34437 | N-Nitrosodiphenylamine, tissue | 25 | 0 | 0.0% | 25 | 100.0% |
| Sem | T | 34442 | N-Nitrosodimethylamine, tissue | 25 | 0 | 0.0% | 25 | 100.0% |
| Sem | T | 34451 | Nitrobenzene, tissue | 348 | 0 | 0.0% | 348 | 100.0% |
| Sem | T | 34456 | Parachlorometa Cresol, tissue | 343 | 2 | 0.6% | 341 | 99.4% |
| Sem | T | 34468 | Phenol, tissue | 348 | 18 | 5.2% | 330 | 94.8% |
| Sem | T | 34540 | 1,2-Dichlorobenzene, tissue | 343 | 0 | 0.0% | 343 | 100.0% |
| Sem | T | 34555 | 1,2,4-Trichlorobenzene, tissue | 348 | 0 | 0.0% | 348 | 100.0% |
| Sem | T | 34570 | 1,3-Dichlorobenzene, tissue | 344 | 0 | 0.0% | 344 | 100.0% |
| Sem | T | 34575 | 1,4-Dichlorobenzene, tissue | 344 | 0 | 0.0% | 344 | 100.0% |
| Sem | T | 34585 | 2-Chloronaphthalene, tissue | 25 | 0 | 0.0% | 25 | 100.0% |
| Sem | T | 34590 | 2-Chlorophenol, tissue | 343 | 0 | 0.0% | 343 | 100.0% |
| Sem | T | 34595 | 2-Nitrophenol, tissue | 354 | 0 | 0.0% | 354 | 100.0% |
| Sem | T | 34600 | Di-N-Octyl Phthalate, tissue | 25 | 0 | 0.0% | 25 | 100.0% |
| Sem | T | 34605 | 2,4-Dichlorophenol, tissue | 344 | 0 | 0.0% | 344 | 100.0% |
| Sem | T | 34610 | 2,4-Dimethylphenol, tissue | 344 | 0 | 0.0% | 344 | 100.0% |
| Sem | T | 34615 | 2,4-Dinitrotoluene, tissue | 348 | 0 | 0.0% | 348 | 100.0% |
| Sem | T | 34620 | 2,4-Dinitrophenol, tissue | 349 | 0 | 0.0% | 349 | 100.0% |
| Sem | T | 34625 | 2,4,6-Trichlorophenol, tissue | 25 | 0 | 0.0% | 25 | 100.0% |
| Sem | T | 34630 | 2,6-Dinitrotoluene, tissue | 348 | 0 | 0.0% | 348 | 100.0% |
| Sem | T | 34635 | 3,3 Dichlorobenzidine, tissue | 25 | 0 | 0.0% | 25 | 100.0% |
| Sem | T | 34640 | 4-Bromophenyl Phenyl Ether, tissue | 25 | 0 | 0.0% | 25 | 100.0% |
| Sem | T | 34645 | 4-Chlorophenyl Phenyl Ether, tissue | 25 | 0 | 0.0% | 25 | 100.0% |
| Sem | T | 34650 | 4-Nitrophenol, tissue | 354 | 0 | 0.0% | 354 | 100.0% |
| Sem | T | 34683 | Di-N-Butyl Phthalate, tissue | 343 | 1 | 0.3% | 342 | 99.7% |
| Sem | T | 34688 | Hexachlorobenzene, tissue | 348 | 0 | 0.0% | 348 | 100.0% |
| Sem | T | 39099 | Bis(2-Ethylhexyl)phthalate, tissue | 343 | 0 | 0.0% | 343 | 100.0% |
| Sem | W | 34273 | Bis(2-Chloroethyl)ether | 237 | 0 | 0.0% | 237 | 100.0% |
| Sem | W | 34278 | Bis(2-Chloroethoxy)methane | 233 | 0 | 0.0% | 233 | 100.0% |
| Sem | W | 34283 | Bis(2-Chloroisopropyl)ether | 237 | 0 | 0.0% | 237 | 100.0% |
| Sem | W | 34292 | Butylbenzyl Phthalate | 1958 | 4 | 0.2% | 1954 | 99.8% |
| Sem | W | 34336 | Diethyl Phthalate | 1957 | 0 | 0.0% | 1957 | 100.0% |
| Sem | W | 34341 | Dimethyl Phthalate, total | 1957 | 0 | 0.0% | 1957 | 100.0% |
| Sem | W | 34346 | 1,2-Diphenylhydrazine, total | 238 | 0 | 0.0% | 238 | 100.0% |
| Sem | W | 34386 | Hexachlorocyclopentadiene | 238 | 0 | 0.0% | 238 | 100.0% |
| Sem | W | 34391 | Hexachlorobutadiene | 1958 | 0 | 0.0% | 1958 | 100.0% |
| Sem | W | 34396 | Hexachloroethane | 1958 | 0 | 0.0% | 1958 | 100.0% |
| Sem | W | 34408 | Isophorone | 238 | 0 | 0.0% | 238 | 100.0% |
| Sem | W | 34428 | N-Nitroso-Di-N-Propylamine, total | 238 | 0 | 0.0% | 238 | 100.0% |
| Sem | W | 34433 | N-Nitrosodiphenylamine, total | 238 | 0 | 0.0% | 238 | 100.0% |
| Sem | W | 34438 | N-Nitrosodimethylamine | 238 | 0 | 0.0% | 238 | 100.0% |
| Sem | W | 34447 | Nitrobenzene, total | 1958 | 0 | 0.0% | 1958 | 100.0% |
| Sem | W | 34452 | 4-Chloro-3-cresol (3-methyl-4-chlorophenol) | 1957 | 0 | 0.0% | 1957 | 100.0% |
| Sem | W | 34536 | 1,2-Dichlorobenzene, total | 1957 | 0 | 0.0% | 1957 | 100.0% |
| Sem | W | 34551 | 1,2,4-Trichlorobenzene | 1959 | 0 | 0.0% | 1959 | 100.0% |
| Sem | W | 34566 | 1,3-Dichlorobenzene, total | 1957 | 0 | 0.0% | 1957 | 100.0% |
| Sem | W | 34571 | 1,4-Dichlorobenzene, total | 1923 | 0 | 0.0% | 1923 | 100.0% |
| Sem | W | 34581 | 2-Chloronaphthalene, total | 238 | 0 | 0.0% | 238 | 100.0% |
| Sem | W | 34586 | 2-Chlorophenol | 1958 | 0 | 0.0% | 1958 | 100.0% |
| Sem | W | 34591 | 2-Nitrophenol, total | 1957 | 0 | 0.0% | 1957 | 100.0% |
| Sem | W | 34596 | Di-N-octyl Phthalate | 237 | 0 | 0.0% | 237 | 100.0% |
| Sem | W | 34601 | 2,4-Dichlorophenol, total | 1958 | 0 | 0.0% | 1958 | 100.0% |
| Sem | W | 34606 | 2,4-Dimethylphenol, total | 1957 | 0 | 0.0% | 1957 | 100.0% |
| Sem | W | 34611 | 2,4-Dinitrotoluene, total | 1958 | 0 | 0.0% | 1958 | 100.0% |
| Sem | W | 34616 | 2,4-Dinitrophenol, total | 1957 | 0 | 0.0% | 1957 | 100.0% |
| Sem | W | 34621 | 2,4,6-Trichlorophenol, total | 238 | 0 | 0.0% | 238 | 100.0% |
| Sem | W | 34626 | 2,6-Dinitrotoluene, total | 1958 | 0 | 0.0% | 1958 | 100.0% |
| Sem | W | 34631 | 3,3'-Dichlorobenzidine, total | 246 | 0 | 0.0% | 246 | 100.0% |
| Sem | W | 34636 | 4-Bromophenyl Phenyl Ether, total | 236 | 0 | 0.0% | 236 | 100.0% |
| Sem | W | 34641 | 4-Chlorophenyl Phenyl Ether, total | 238 | 0 | 0.0% | 238 | 100.0% |
| Sem | W | 34646 | 4-Nitrophenol, total | 1957 | 0 | 0.0% | 1957 | 100.0% |
| Sem | W | 34694 | Phenol | 1964 | 0 | 0.0% | 1964 | 100.0% |
| Sem | W | 39100 | BIS(2-Ethylhexyl) Phthalate | 1956 | 8 | 0.4% | 1948 | 99.6% |
| Sem | W | 39110 | Di-N-Butyl Phthalate | 1954 | 14 | 0.7% | 1940 | 99.3% |
| Sem | W | 39120 | Benzidine | 238 | 0 | 0.0% | 238 | 100.0% |
| Sem | W | 39700 | Hexachlorobenzene | 1958 | 0 | 0.0% | 1958 | 100.0% |
| Vol | P | P4001 | Acrolein | 223 | 0 | 0.0% | 223 | 100.0% |
| Vol | P | P4002 | Acrylonitrile | 1930 | 0 | 0.0% | 1930 | 100.0% |
| Vol | P | P4003 | Benzene | 1931 | 0 | 0.0% | 1931 | 100.0% |
| Vol | P | P4004 | Bromodichloromethane | 223 | 0 | 0.0% | 223 | 100.0% |
| Vol | P | P4005 | Bromoform | 223 | 0 | 0.0% | 223 | 100.0% |
| Vol | P | P4006 | Bromomethane | 223 | 0 | 0.0% | 223 | 100.0% |
| Vol | P | P4007 | Carbon Tetrachloride | 1923 | 0 | 0.0% | 1923 | 100.0% |
| Vol | P | P4008 | Chlorobenzene | 1931 | 2 | 0.1% | 1929 | 99.9% |
| Vol | P | P4009 | Chloroethane | 1931 | 0 | 0.0% | 1931 | 100.0% |
| Vol | P | P4010 | 2-Chloroethyl Vinyl Ether | 223 | 0 | 0.0% | 223 | 100.0% |
| Vol | P | P4011 | Chloroform | 1931 | 0 | 0.0% | 1931 | 100.0% |
| Vol | P | P4012 | Chloromethane | 1931 | 0 | 0.0% | 1931 | 100.0% |
| Vol | P | P4013 | Dibromochloromethane | 223 | 0 | 0.0% | 223 | 100.0% |
| Vol | P | P4014 | 1,1-Dichloroethane | 1901 | 0 | 0.0% | 1901 | 100.0% |
| Vol | P | P4015 | 1,2-Dichloroethane | 1931 | 2 | 0.1% | 1929 | 99.9% |
| Vol | P | P4016 | 1,1-Dichloroethane | 1930 | 0 | 0.0% | 1930 | 100.0% |
| Vol | P | P4017 | Trans-1,2-Dichloroethene | 1853 | 0 | 0.0% | 1853 | 100.0% |
| Vol | P | P4018 | 1,2-Dichloropropane | 1862 | 0 | 0.0% | 1862 | 100.0% |
| Vol | P | P4019 | CIS-1,3-Dichloropropene | 1931 | 0 | 0.0% | 1931 | 100.0% |
| Vol | P | P4020 | Trans-1,3-Dichloropropene | 1922 | 0 | 0.0% | 1922 | 100.0% |
| Vol | P | P4021 | Ethylbenzene | 1931 | 0 | 0.0% | 1931 | 100.0% |
| Vol | P | P4022 | Methylene Chloride | 1932 | 4 | 0.2% | 1928 | 99.8% |
| Vol | P | P4023 | 1,1,2,2-Tetrachloroethane | 223 | 0 | 0.0% | 223 | 100.0% |
| Vol | P | P4024 | Tetrachloroethene | 1931 | 0 | 0.0% | 1931 | 100.0% |
| Vol | P | P4025 | Toluene | 1931 | 0 | 0.0% | 1931 | 100.0% |
| Vol | P | P4026 | 1,1,1-Trichloroethane | 1931 | 0 | 0.0% | 1931 | 100.0% |
| Vol | P | P4027 | 1,1,2-Trichloroethane | 1931 | 0 | 0.0% | 1931 | 100.0% |
| Vol | P | P4028 | Trichloroethene | 1901 | 0 | 0.0% | 1901 | 100.0% |
| Vol | P | P4029 | Vinyl Chloride | 1900 | 1 | 0.1% | 1899 | 100.0% |
| Vol | S | 34218 | Acrylonitrile | 2322 | 0 | 0.0% | 2322 | 100.0% |
| Vol | S | 34237 | Benzene | 2322 | 0 | 0.0% | 2322 | 100.0% |
| Vol | S | 34290 | Bromoform | 255 | 0 | 0.0% | 255 | 100.0% |
| Vol | S | 34299 | Carbon Tetrachloride | 2319 | 0 | 0.0% | 2319 | 100.0% |
| Vol | S | 34304 | Chlorobenzene | 2322 | 0 | 0.0% | 2322 | 100.0% |
| Vol | S | 34309 | Dibromochloromethane | 255 | 0 | 0.0% | 255 | 100.0% |
| Vol | S | 34314 | Chloroethane | 2322 | 0 | 0.0% | 2322 | 100.0% |
| Vol | S | 34318 | Chloroform | 2322 | 0 | 0.0% | 2322 | 100.0% |
| Vol | S | 34330 | Bromodichloromethane | 255 | 0 | 0.0% | 255 | 100.0% |
| Vol | S | 34374 | Ethylbenzene | 2322 | 0 | 0.0% | 2322 | 100.0% |
| Vol | S | 34426 | Methylene Chloride | 2322 | 0 | 0.0% | 2322 | 100.0% |
| Vol | S | 34478 | Tetrachloroethylene | 2322 | 0 | 0.0% | 2322 | 100.0% |
| Vol | S | 34483 | Toluene | 2325 | 5 | 0.2% | 2320 | 99.8% |
| Vol | S | 34487 | Trichloroethylene | 2322 | 0 | 0.0% | 2322 | 100.0% |
| Vol | S | 34495 | Vinyl Chloride | 2351 | 0 | 0.0% | 2351 | 100.0% |
| Vol | S | 34499 | 1,1-Dichloroethane | 2322 | 0 | 0.0% | 2322 | 100.0% |
| Vol | S | 34504 | 1,1-Dichloroethylene | 2322 | 0 | 0.0% | 2322 | 100.0% |
| Vol | S | 34509 | 1,1,1-Trichloroethane | 2323 | 1 | 0.0% | 2322 | 100.0% |
| Vol | S | 34514 | 1,1,2-Trichloroethane | 2322 | 0 | 0.0% | 2322 | 100.0% |
| Vol | S | 34519 | 1,1,2,2-Tetrachloroethane | 265 | 0 | 0.0% | 265 | 100.0% |
| Vol | S | 34534 | 1,2-Dichloroethane | 2322 | 0 | 0.0% | 2322 | 100.0% |
| Vol | S | 34544 | 1,2-Dichloropropane | 2214 | 0 | 0.0% | 2214 | 100.0% |
| Vol | S | 34549 | Trans-1,2-Dichloroethene | 2214 | 0 | 0.0% | 2214 | 100.0% |
| Vol | S | 34564 | 1,3-Dichloropropene | 1 | 0 | 0.0% | 1 | 100.0% |
| Vol | S | 34579 | 2-Chloroethyl Vinyl Ether | 255 | 0 | 0.0% | 255 | 100.0% |
| Vol | S | 34697 | Trans-1,3-Dichloropropene | 2322 | 0 | 0.0% | 2322 | 100.0% |
| Vol | S | 34702 | CIS-1,3-Dichloropropene | 2322 | 0 | 0.0% | 2322 | 100.0% |
| Vol | S | 88802 | Bromomethane | 255 | 0 | 0.0% | 255 | 100.0% |
| Vol | S | 88835 | Chloromethane | 2322 | 0 | 0.0% | 2322 | 100.0% |
| Vol | T | 34219 | Acrylonitrile, tissue | 354 | 0 | 0.0% | 354 | 100.0% |
| Vol | T | 34238 | Benzene, tissue | 354 | 0 | 0.0% | 354 | 100.0% |
| Vol | T | 34291 | Bromoform, tissue | 25 | 0 | 0.0% | 25 | 100.0% |
| Vol | T | 34300 | Carbon Tetrachloride, tissue | 353 | 0 | 0.0% | 353 | 100.0% |
| Vol | T | 34305 | Chlorobenzene, tissue | 354 | 0 | 0.0% | 354 | 100.0% |
| Vol | T | 34310 | Dibromochloromethane, tissue | 25 | 0 | 0.0% | 25 | 100.0% |
| Vol | T | 34315 | Chlorethane, tissue | 344 | 0 | 0.0% | 344 | 100.0% |
| Vol | T | 34319 | Chloroform, tissue | 354 | 4 | 1.1% | 350 | 98.9% |
| Vol | T | 34331 | Bromodichloromethane, tissue | 25 | 0 | 0.0% | 25 | 100.0% |
| Vol | T | 34375 | Ethylbenzene, tissue | 344 | 0 | 0.0% | 344 | 100.0% |
| Vol | T | 34427 | Methylene Chloride, tissue | 344 | 13 | 3.8% | 331 | 96.2% |
| Vol | T | 34479 | Tetrachloroethylene, tissue | 334 | 0 | 0.0% | 334 | 100.0% |
| Vol | T | 34484 | Toluene, tissue | 344 | 1 | 0.3% | 343 | 99.7% |
| Vol | T | 34500 | 1,1-Dichloroethane, tissue | 344 | 0 | 0.0% | 344 | 100.0% |
| Vol | T | 34505 | 1,1-Dichloroethylene, tissue | 354 | 0 | 0.0% | 354 | 100.0% |
| Vol | T | 34510 | 1,1,1-Trichloroethane, tissue | 344 | 0 | 0.0% | 344 | 100.0% |
| Vol | T | 34515 | 1,1,2-Trichloroethane, tissue | 344 | 0 | 0.0% | 344 | 100.0% |
| Vol | T | 34520 | 1,1,2,2-Tetrachloroethane, tissue | 25 | 0 | 0.0% | 25 | 100.0% |
| Vol | T | 34535 | 1,2-Dichloroethane, tissue | 344 | 0 | 0.0% | 344 | 100.0% |
| Vol | T | 34545 | 1,2-Dichloropropane, tissue | 344 | 0 | 0.0% | 344 | 100.0% |
| Vol | T | 34550 | Trans-1,2-Dichloroethene, tissue | 354 | 0 | 0.0% | 354 | 100.0% |
| Vol | T | 34580 | 2-Chloroethyl Vinyl Ether, tissue | 25 | 0 | 0.0% | 25 | 100.0% |
| Vol | T | 34692 | Trichloroethylene, tissue | 354 | 0 | 0.0% | 354 | 100.0% |
| Vol | T | 34693 | Vinyl Chloride, tissue | 343 | 0 | 0.0% | 343 | 100.0% |
| Vol | T | 34698 | Trans-1,3-Dichloropropene, tissue | 344 | 0 | 0.0% | 344 | 100.0% |
| Vol | T | 34703 | CIS-1,3-Dichloropropene, tissue | 344 | 0 | 0.0% | 344 | 100.0% |
| Vol | T | 88803 | Bromomethane, tissue | 25 | 0 | 0.0% | 25 | 100.0% |
| Vol | T | 88836 | Chloromethane, tissue | 344 | 1 | 0.3% | 343 | 99.7% |
| Vol | W | 30201 | Chloromethane | 1972 | 0 | 0.0% | 1972 | 100.0% |
| Vol | W | 30202 | Bromomethane | 237 | 0 | 0.0% | 237 | 100.0% |
| Vol | W | 32101 | Bromodichloromethane | 237 | 0 | 0.0% | 237 | 100.0% |
| Vol | W | 32102 | Carbon Tetrachloride | 1956 | 0 | 0.0% | 1956 | 100.0% |
| Vol | W | 32104 | Bromoform | 237 | 0 | 0.0% | 237 | 100.0% |
| Vol | W | 32106 | Chloroform | 1962 | 0 | 0.0% | 1962 | 100.0% |
| Vol | W | 32730 | Phenolics, total | 6 | 2 | 33.3% | 4 | 66.7% |
| Vol | W | 34010 | Toluene | 1927 | 0 | 0.0% | 1927 | 100.0% |
| Vol | W | 34030 | Benzene | 1927 | 1 | 0.1% | 1926 | 100.0% |
| Vol | W | 34215 | Acrylonitrile, total | 1961 | 0 | 0.0% | 1961 | 100.0% |
| Vol | W | 34301 | Chlorobenzene | 1962 | 0 | 0.0% | 1962 | 100.0% |
| Vol | W | 34306 | Chlorodibromomethane, total | 237 | 0 | 0.0% | 237 | 100.0% |
| Vol | W | 34311 | Chloroethane | 1972 | 0 | 0.0% | 1972 | 100.0% |
| Vol | W | 34371 | Ethylbenzene | 1972 | 0 | 0.0% | 1972 | 100.0% |
| Vol | W | 34423 | Methylene Chloride | 1972 | 0 | 0.0% | 1972 | 100.0% |
| Vol | W | 34475 | Tetrachloroethene | 1972 | 0 | 0.0% | 1972 | 100.0% |
| Vol | W | 34496 | 1,1-Dichloroethane, total | 1973 | 0 | 0.0% | 1973 | 100.0% |
| Vol | W | 34501 | 1,1-Dichloroethene | 1987 | 0 | 0.0% | 1987 | 100.0% |
| Vol | W | 34506 | 1,1,1-Trichloroethane, total | 1973 | 0 | 0.0% | 1973 | 100.0% |
| Vol | W | 34511 | 1,1,2-Trichloroethane, total | 1972 | 0 | 0.0% | 1972 | 100.0% |
| Vol | W | 34513 | 1,1,2-Trichloroethane, suspended | 10 | 0 | 0.0% | 10 | 100.0% |
| Vol | W | 34516 | 1,1,2,2-Tetrachloroethane, total | 237 | 0 | 0.0% | 237 | 100.0% |
| Vol | W | 34531 | 1,2-Dichloroethane, total | 1972 | 0 | 0.0% | 1972 | 100.0% |
| Vol | W | 34541 | 1,2-Dichloropropane, total | 1891 | 0 | 0.0% | 1891 | 100.0% |
| Vol | W | 34546 | Trans-l,2-Dichloroethene | 1881 | 0 | 0.0% | 1881 | 100.0% |
| Vol | W | 34576 | 2-Chloroethyl Vinyl Ether, total | 237 | 0 | 0.0% | 237 | 100.0% |
| Vol | W | 34699 | Trans-l,3-Dichloropropene | 1962 | 0 | 0.0% | 1962 | 100.0% |
| Vol | W | 34704 | CIS-1,3-Dichloropropene | 1972 | 0 | 0.0% | 1972 | 100.0% |
| Vol | W | 39175 | Vinyl Chloride | 1958 | 0 | 0.0% | 1958 | 100.0% |
| Vol | W | 39180 | Trichloroethylene | 1963 | 0 | 0.0% | 1963 | 100.0% |
| Vol | W | 77226 | 1,3,5-Trimethylbenzene, total | 1 | 0 | 0.0% | 1 | 100.0% |
| Vol | W | 78124 | Benzene | 35 | 0 | 0.0% | 35 | 100.0% |
| Vol | W | 78131 | Toluene | 45 | 0 | 0.0% | 45 | 100.0% |

# Table S6: Species List

## Benthic Infauna

Average abundance (n/m^2^) of benthic infauna caught in cores over all sampling periods.

| **Rank** | **Taxa/Species Name** | **Distance from Discharge** | | | | **Mean** | **Pct** | **CumPct** |
| --- | --- | --- | --- | --- | --- | --- | --- | --- |
|  |  | **A** | **B** | **C** | **R** |  |  |  |
| 1 | Mulinia lateralis | 109.1606 | 199.1237 | 155.1022 | 174.9941 | 159.5952 | 23.824% | 23.824% |
| 2 | Mediomastus ambiseta | 34.2322 | 71.4947 | 143.9977 | 36.6490 | 71.5934 | 10.687% | 34.512% |
| 3 | Rangia cuneata | 23.2833 | 25.5496 | 30.9917 | 54.9634 | 33.6970 | 5.030% | 39.542% |
| 4 | Acteocina canaliculata | 19.4819 | 32.0960 | 25.8415 | 29.1754 | 26.6487 | 3.978% | 43.520% |
| 5 | Ampelisca | 13.4341 | 30.5778 | 37.5675 | 7.4695 | 22.2622 | 3.323% | 46.843% |
| 6 | Hydrobiidae | 9.2773 | 23.8340 | 28.9980 | 25.4388 | 21.8870 | 3.267% | 50.111% |
| 7 | Capitella capitata | 17.6886 | 25.7099 | 24.9114 | 7.0394 | 18.8373 | 2.812% | 52.923% |
| 8 | Ameritella mitchelli | 14.0488 | 15.1686 | 17.1167 | 14.3952 | 15.1823 | 2.266% | 55.189% |
| 9 | Corophium | 32.8301 | 13.1839 | 11.1578 | 3.3009 | 15.1182 | 2.257% | 57.446% |
| 10 | Texadina sphinctostoma | 7.1893 | 8.2187 | 11.8224 | 30.0159 | 14.3116 | 2.136% | 59.582% |
| 11 | Loandalia americana | 7.3909 | 11.7793 | 16.6578 | 9.4119 | 11.3100 | 1.688% | 61.271% |
| 12 | Texadina | 6.4929 | 12.0377 | 5.2859 | 19.8854 | 10.9255 | 1.631% | 62.902% |
| 13 | Lymnaeidae | 1.6229 | 1.6674 | 1.4713 | 35.8676 | 10.1573 | 1.516% | 64.418% |
| 14 | Streblospio gyrobranchiata | 4.5022 | 8.4169 | 16.1306 | 9.7095 | 9.6898 | 1.446% | 65.865% |
| 15 | Macoma | 10.4740 | 11.9628 | 6.6119 | 6.4754 | 8.8810 | 1.326% | 67.190% |
| 16 | Texadina barretti | 1.3118 | 1.9666 | 5.0806 | 24.4168 | 8.1940 | 1.223% | 68.414% |
| 17 | Nemertea | 4.2147 | 8.6806 | 15.3845 | 4.2122 | 8.1230 | 1.213% | 69.626% |
| 18 | Molgula | 3.0835 | 13.2929 | 11.9736 | 1.4745 | 7.4561 | 1.113% | 70.739% |
| 19 | Tellina | 7.0492 | 8.1909 | 3.5954 | 8.1272 | 6.7407 | 1.006% | 71.745% |
| 20 | Mediomastus californiensis | 7.2921 | 9.6770 | 6.7252 | 2.2365 | 6.4827 | 0.968% | 72.713% |
| 21 | Glycinde solitaria | 3.4578 | 7.2287 | 10.4891 | 4.3032 | 6.3697 | 0.951% | 73.664% |
| 22 | Hermundura fauveli | 1.6848 | 3.3893 | 15.6028 | 4.0310 | 6.1770 | 0.922% | 74.586% |
| 23 | Littoridina | 4.5368 | 2.9349 | 3.7208 | 13.4999 | 6.1731 | 0.922% | 75.508% |
| 24 | Laeonereis culveri | 1.7768 | 5.4459 | 11.4464 | 5.2804 | 5.9874 | 0.894% | 76.401% |
| 25 | Leitoscoloplos fragilis | 2.4493 | 4.4792 | 9.2938 | 5.2356 | 5.3645 | 0.801% | 77.202% |
| 26 | Balanus | 6.0306 | 0.7636 | 1.7752 | 9.4893 | 4.5147 | 0.674% | 77.876% |
| 27 | Tagelus divisus | 5.0449 | 3.6070 | 3.1698 | 4.5647 | 4.0966 | 0.612% | 78.488% |
| 28 | Mediomastus | 2.5807 | 6.9722 | 3.6253 | 3.0653 | 4.0609 | 0.606% | 79.094% |
| 29 | Monocorophium ascherusicum | 14.4165 | 1.2307 | 0.4803 | 0.0000 | 4.0319 | 0.602% | 79.696% |
| 30 | Neanthes succinea | 3.5411 | 4.3106 | 4.1000 | 3.9905 | 3.9856 | 0.595% | 80.291% |
| 31 | Idotea | 9.2565 | 1.6286 | 2.4995 | 0.0704 | 3.3637 | 0.502% | 80.793% |
| 32 | Leitoscoloplus | 1.6672 | 3.6248 | 5.2365 | 2.4358 | 3.2411 | 0.484% | 81.277% |
| 33 | Heteromastus filiformis | 1.5809 | 2.8806 | 7.1802 | 0.5727 | 3.0536 | 0.456% | 81.733% |
| 34 | Spiochaetopterus costarum | 2.3871 | 4.8567 | 3.7702 | 0.5933 | 2.9018 | 0.433% | 82.166% |
| 35 | Ensis minor | 2.3160 | 3.7837 | 5.2677 | 0.1450 | 2.8781 | 0.430% | 82.595% |
| 36 | Polydora cornuta | 1.6466 | 3.4602 | 4.8676 | 1.2642 | 2.8096 | 0.419% | 83.015% |
| 37 | Glycinde multidens | 1.6152 | 3.0945 | 5.0351 | 1.4041 | 2.7872 | 0.416% | 83.431% |
| 38 | Polydora | 5.5173 | 2.7623 | 1.8159 | 0.9961 | 2.7729 | 0.414% | 83.845% |
| 39 | Clymenella torquata | 0.9436 | 3.0591 | 5.7547 | 0.8946 | 2.6630 | 0.398% | 84.242% |
| 40 | Diopatra cuprea | 2.0746 | 2.9937 | 4.2474 | 1.0771 | 2.5982 | 0.388% | 84.630% |
| 41 | Scolelepis texana | 2.0183 | 3.7626 | 4.0754 | 0.1812 | 2.5094 | 0.375% | 85.005% |
| 42 | Cerapus benthophilus | 1.2721 | 4.6537 | 2.9793 | 0.4095 | 2.3287 | 0.348% | 85.352% |
| 43 | Tagelus plebus | 2.2247 | 2.4949 | 4.0627 | 0.4073 | 2.2974 | 0.343% | 85.695% |
| 44 | Ampelisca vadorum | 2.8786 | 0.5986 | 5.1396 | 0.2154 | 2.2080 | 0.330% | 86.025% |
| 45 | Hermundura tricuspis | 0.4011 | 0.7302 | 4.7639 | 2.6074 | 2.1257 | 0.317% | 86.342% |
| 46 | Parandalia americana | 0.5374 | 1.8619 | 4.1495 | 1.8968 | 2.1114 | 0.315% | 86.658% |
| 47 | Cossura delta | 2.1169 | 2.7288 | 1.5741 | 1.9798 | 2.0999 | 0.313% | 86.971% |
| 48 | Streblospio benedicti | 1.6133 | 2.2455 | 1.6816 | 2.7710 | 2.0778 | 0.310% | 87.281% |
| 49 | Platyhelminthes | 1.8818 | 2.0525 | 2.7153 | 1.3532 | 2.0007 | 0.299% | 87.580% |
| 50 | Polydora socialis | 2.8996 | 2.6703 | 1.4746 | 0.4711 | 1.8789 | 0.280% | 87.860% |
| 51 | Rangia | 3.2304 | 0.6319 | 0.6842 | 2.8714 | 1.8545 | 0.277% | 88.137% |
| 52 | Capitellidae | 2.1163 | 1.7760 | 2.3930 | 0.8689 | 1.7885 | 0.267% | 88.404% |
| 53 | Hyalella azteca | 1.3183 | 2.4237 | 3.1490 | 0.1024 | 1.7483 | 0.261% | 88.665% |
| 54 | Cyclaspis varians | 2.3915 | 1.3294 | 2.5686 | 0.2112 | 1.6252 | 0.243% | 88.908% |
| 55 | Littoridinidae | 0.4008 | 0.0678 | 0.0000 | 5.6462 | 1.5287 | 0.228% | 89.136% |
| 56 | Brachidontes | 4.6040 | 0.0444 | 0.2227 | 1.2157 | 1.5217 | 0.227% | 89.363% |
| 57 | Bivalvia | 0.6049 | 1.9504 | 2.7395 | 0.5550 | 1.4624 | 0.218% | 89.581% |
| 58 | Lysonia haylina | 1.5833 | 2.0305 | 1.7301 | 0.3945 | 1.4346 | 0.214% | 89.796% |
| 59 | Chironomidae | 2.1596 | 1.4589 | 1.4370 | 0.6119 | 1.4169 | 0.212% | 90.007% |
| 60 | Neanthes | 1.6829 | 1.2326 | 1.4739 | 1.1110 | 1.3751 | 0.205% | 90.212% |
| 61 | Gammarus | 2.3224 | 0.9676 | 1.5078 | 0.3147 | 1.2781 | 0.191% | 90.403% |
| 62 | Amphipoda | 1.0796 | 1.1293 | 2.4052 | 0.4711 | 1.2713 | 0.190% | 90.593% |
| 63 | Glycera | 1.5791 | 1.1312 | 1.7119 | 0.4492 | 1.2178 | 0.182% | 90.775% |
| 64 | Eteone heteropoda | 1.8153 | 1.1851 | 1.3037 | 0.4879 | 1.1980 | 0.179% | 90.954% |
| 65 | Edotea triloba | 0.9778 | 1.3648 | 2.1573 | 0.2446 | 1.1861 | 0.177% | 91.131% |
| 66 | Odostomia impressa | 0.2184 | 0.5263 | 1.1155 | 2.8546 | 1.1787 | 0.176% | 91.307% |
| 67 | Littoridinops monroensis | 0.0995 | 0.0000 | 0.0333 | 4.4762 | 1.1523 | 0.172% | 91.479% |
| 68 | Pachydiplax | 1.7058 | 0.5654 | 1.8146 | 0.0342 | 1.0300 | 0.154% | 91.632% |
| 69 | Amygdalum papyria | 1.4178 | 1.2987 | 1.1297 | 0.2280 | 1.0185 | 0.152% | 91.784% |
| 70 | Aligena texasiana | 0.1015 | 1.2298 | 2.3288 | 0.3759 | 1.0090 | 0.151% | 91.935% |
| 71 | Apocorophium louisianum | 1.4476 | 0.9314 | 1.3366 | 0.2837 | 0.9998 | 0.149% | 92.084% |
| 72 | Grandidierella bonnieroides | 0.5719 | 0.7992 | 2.0551 | 0.5716 | 0.9994 | 0.149% | 92.234% |
| 73 | Acetocina | 0.8778 | 1.4743 | 1.0986 | 0.4766 | 0.9818 | 0.147% | 92.380% |
| 74 | Prostoma | 0.6701 | 0.8298 | 1.6461 | 0.7407 | 0.9717 | 0.145% | 92.525% |
| 75 | Odostomia | 0.5550 | 0.0838 | 0.0977 | 3.0314 | 0.9420 | 0.141% | 92.666% |
| 76 | Potamilla | 0.3034 | 1.6617 | 1.5064 | 0.0362 | 0.8769 | 0.131% | 92.797% |
| 77 | Brachidontes exustus | 2.7897 | 0.2324 | 0.2555 | 0.2154 | 0.8732 | 0.130% | 92.927% |
| 78 | Tubulanus pellucidus | 0.6725 | 0.7647 | 1.7151 | 0.3200 | 0.8681 | 0.130% | 93.057% |
| 79 | Pectinaria gouldi | 0.4375 | 0.5980 | 1.6546 | 0.7271 | 0.8543 | 0.128% | 93.184% |
| 80 | Glycinde | 0.3027 | 0.6653 | 1.4723 | 0.8594 | 0.8249 | 0.123% | 93.307% |
| 81 | Streblospio | 0.8726 | 1.3291 | 0.6850 | 0.3624 | 0.8123 | 0.121% | 93.429% |
| 82 | Aphelochaeta | 0.1009 | 0.3993 | 2.6396 | 0.0000 | 0.7850 | 0.117% | 93.546% |
| 83 | Capitella | 1.0008 | 0.5357 | 1.1566 | 0.3868 | 0.7700 | 0.115% | 93.661% |
| 84 | Mactridae | 1.6538 | 0.5329 | 0.8897 | 0.0000 | 0.7691 | 0.115% | 93.775% |
| 85 | Tanypus | 0.4668 | 0.5325 | 0.2737 | 1.7906 | 0.7659 | 0.114% | 93.890% |
| 86 | Paraprionospio yokoyamai | 0.3689 | 0.4651 | 1.1657 | 0.9899 | 0.7474 | 0.112% | 94.001% |
| 87 | Turbellaria | 0.7051 | 0.8637 | 0.8230 | 0.5632 | 0.7388 | 0.110% | 94.112% |
| 88 | Rictaxis punctostriatus | 0.5381 | 0.3328 | 1.0952 | 0.8646 | 0.7077 | 0.106% | 94.217% |
| 89 | Cumacea | 0.5789 | 0.3763 | 1.2800 | 0.5693 | 0.7011 | 0.105% | 94.322% |
| 90 | Leitoscoloplos robustus | 0.4692 | 0.5648 | 1.5432 | 0.1729 | 0.6875 | 0.103% | 94.425% |
| 91 | Ensis megistus | 0.5058 | 1.0333 | 1.0970 | 0.0704 | 0.6766 | 0.101% | 94.526% |
| 92 | Podarkeopsis levifuscina | 0.4369 | 0.5657 | 0.9939 | 0.6378 | 0.6586 | 0.098% | 94.624% |
| 93 | Ampelisca abdita | 0.1677 | 0.2329 | 2.0870 | 0.0362 | 0.6309 | 0.094% | 94.718% |
| 94 | Paracaprella tenuis | 1.0756 | 0.1661 | 1.1306 | 0.0704 | 0.6107 | 0.091% | 94.809% |
| 95 | Tagelus | 1.6056 | 0.4348 | 0.0670 | 0.2413 | 0.5872 | 0.088% | 94.897% |
| 96 | Ameroculodes miltoni | 0.5394 | 0.5323 | 1.0277 | 0.1812 | 0.5701 | 0.085% | 94.982% |
| 97 | Bittium varium | 0.2235 | 0.3646 | 0.8002 | 0.8372 | 0.5564 | 0.083% | 95.065% |
| 98 | Nuculana acuta | 0.1365 | 0.4311 | 0.4192 | 1.2154 | 0.5505 | 0.082% | 95.147% |
| 99 | Anthozoa | 1.6814 | 0.3657 | 0.0687 | 0.0683 | 0.5460 | 0.082% | 95.229% |
| 100 | Ensis | 0.9076 | 0.6992 | 0.4800 | 0.0725 | 0.5398 | 0.081% | 95.309% |
| 101 | Odostomia laevigata | 0.8770 | 0.0764 | 0.2976 | 0.8416 | 0.5232 | 0.078% | 95.387% |
| 102 | Mysis | 0.3683 | 0.7296 | 0.6814 | 0.2844 | 0.5159 | 0.077% | 95.464% |
| 103 | Melinna maculata | 0.4633 | 0.3838 | 0.4131 | 0.7794 | 0.5099 | 0.076% | 95.541% |
| 104 | Tanypus clavatus | 0.2015 | 0.6315 | 0.5152 | 0.6865 | 0.5087 | 0.076% | 95.617% |
| 105 | Cyclopoida | 0.0338 | 0.1993 | 0.2063 | 1.5439 | 0.4958 | 0.074% | 95.691% |
| 106 | Sabellidae | 0.0671 | 0.1993 | 1.6796 | 0.0000 | 0.4865 | 0.073% | 95.763% |
| 107 | Polychaeta | 0.6816 | 0.4322 | 0.5908 | 0.2395 | 0.4860 | 0.073% | 95.836% |
| 108 | Goniadidae | 0.4195 | 0.4755 | 0.3860 | 0.6515 | 0.4831 | 0.072% | 95.908% |
| 109 | Chironominae | 0.0768 | 0.8809 | 0.6188 | 0.3262 | 0.4757 | 0.071% | 95.979% |
| 110 | Batea catharinensis | 0.0674 | 0.5654 | 1.1312 | 0.0362 | 0.4501 | 0.067% | 96.046% |
| 111 | Nassarius vibex | 0.0756 | 0.3238 | 0.3344 | 1.0637 | 0.4494 | 0.067% | 96.113% |
| 112 | Nereis | 0.2145 | 0.4792 | 0.8775 | 0.1899 | 0.4403 | 0.066% | 96.179% |
| 113 | Membranipora | 0.2341 | 0.0995 | 0.2395 | 1.1723 | 0.4364 | 0.065% | 96.244% |
| 114 | Pandora trilineata | 0.1336 | 0.3334 | 0.5822 | 0.5262 | 0.3939 | 0.059% | 96.303% |
| 115 | Japonactaeon punctostriatus | 0.2025 | 0.4996 | 0.5809 | 0.2796 | 0.3906 | 0.058% | 96.361% |
| 116 | Paraprionospio pinnata | 0.0664 | 0.1274 | 0.3418 | 1.0222 | 0.3895 | 0.058% | 96.419% |
| 117 | Oligochaeta | 0.4046 | 0.6000 | 0.3678 | 0.1812 | 0.3884 | 0.058% | 96.477% |
| 118 | Loandalia | 0.0397 | 0.3079 | 0.7490 | 0.4525 | 0.3873 | 0.058% | 96.535% |
| 119 | Oxyurostylis smithi | 0.1012 | 0.3314 | 1.0259 | 0.0000 | 0.3646 | 0.054% | 96.589% |
| 120 | Physa | 1.3549 | 0.0664 | 0.0000 | 0.0000 | 0.3553 | 0.053% | 96.642% |
| 121 | Xenanthura brevitelson | 0.3027 | 0.3999 | 0.6154 | 0.0683 | 0.3466 | 0.052% | 96.694% |
| 122 | Hargeria rapax | 0.1344 | 0.5995 | 0.6499 | 0.0000 | 0.3460 | 0.052% | 96.746% |
| 123 | Kirsteueriella biocellata | 0.2695 | 0.4986 | 0.4794 | 0.1087 | 0.3391 | 0.051% | 96.796% |
| 124 | Corophiidae | 0.5061 | 0.1332 | 0.6170 | 0.0725 | 0.3322 | 0.050% | 96.846% |
| 125 | Cerebratulus lacteus | 0.2718 | 0.3484 | 0.5711 | 0.1362 | 0.3319 | 0.050% | 96.896% |
| 126 | Listriella barnardi | 0.0332 | 0.2990 | 0.9597 | 0.0342 | 0.3315 | 0.049% | 96.945% |
| 127 | Megalomma bioculatum | 0.1341 | 0.3338 | 0.8579 | 0.0000 | 0.3314 | 0.049% | 96.995% |
| 128 | Leptosynapta crassipatina | 0.1677 | 0.2667 | 0.7182 | 0.0683 | 0.3052 | 0.046% | 97.040% |
| 129 | Arabella | 0.1700 | 0.2943 | 0.2562 | 0.4811 | 0.3004 | 0.045% | 97.085% |
| 130 | Cerapus | 0.1015 | 0.5657 | 0.5132 | 0.0000 | 0.2951 | 0.044% | 97.129% |
| 131 | Decapoda | 0.4451 | 0.2975 | 0.1804 | 0.2525 | 0.2939 | 0.044% | 97.173% |
| 132 | Annelida | 0.1337 | 0.3981 | 0.4449 | 0.1699 | 0.2866 | 0.043% | 97.216% |
| 133 | Nassarius acutus | 0.0000 | 0.3322 | 0.5477 | 0.2392 | 0.2798 | 0.042% | 97.257% |
| 134 | Mysella planulata | 0.1012 | 0.0997 | 0.7189 | 0.1708 | 0.2727 | 0.041% | 97.298% |
| 135 | Mydisops | 0.3695 | 0.1661 | 0.4468 | 0.1025 | 0.2712 | 0.040% | 97.339% |
| 136 | Tellinidae | 0.5713 | 0.0995 | 0.3420 | 0.0675 | 0.2701 | 0.040% | 97.379% |
| 137 | Copepoda | 0.0000 | 0.0335 | 0.1708 | 0.8729 | 0.2693 | 0.040% | 97.419% |
| 138 | Ampharetidae | 0.2659 | 0.2617 | 0.3163 | 0.2316 | 0.2689 | 0.040% | 97.459% |
| 139 | Oxyurostylis | 0.2350 | 0.2325 | 0.4465 | 0.0704 | 0.2461 | 0.037% | 97.496% |
| 140 | Bugula neritina | 0.2689 | 0.1996 | 0.4107 | 0.1025 | 0.2454 | 0.037% | 97.533% |
| 141 | Tubificidae | 0.1361 | 0.0664 | 0.0683 | 0.6852 | 0.2390 | 0.036% | 97.568% |
| 142 | Obelia bicuspidata | 0.1351 | 0.2993 | 0.3769 | 0.0725 | 0.2209 | 0.033% | 97.601% |
| 143 | Amathia convoluta | 0.2354 | 0.3331 | 0.2398 | 0.0675 | 0.2190 | 0.033% | 97.634% |
| 144 | Ogyrides alphaerostris | 0.3334 | 0.1334 | 0.0342 | 0.3521 | 0.2132 | 0.032% | 97.666% |
| 145 | Odostomia weberi | 0.1003 | 0.2996 | 0.1708 | 0.2816 | 0.2131 | 0.032% | 97.698% |
| 146 | Nemertina | 0.1338 | 0.4322 | 0.1370 | 0.1408 | 0.2110 | 0.031% | 97.729% |
| 147 | Ischadium recurvum | 0.3375 | 0.0335 | 0.0342 | 0.4349 | 0.2100 | 0.031% | 97.761% |
| 148 | Hirudinea | 0.1273 | 0.1413 | 0.2156 | 0.3538 | 0.2095 | 0.031% | 97.792% |
| 149 | Littorina | 0.1682 | 0.0000 | 0.0000 | 0.6548 | 0.2057 | 0.031% | 97.823% |
| 150 | Crepidula | 0.4697 | 0.0332 | 0.0342 | 0.2777 | 0.2037 | 0.030% | 97.853% |
| 151 | Ceratonereis longicirrata | 0.3351 | 0.3649 | 0.0687 | 0.0342 | 0.2007 | 0.030% | 97.883% |
| 152 | Tubificoides heterochaetus | 0.0338 | 0.0000 | 0.2737 | 0.4711 | 0.1947 | 0.029% | 97.912% |
| 153 | Amphibalanus | 0.0338 | 0.1338 | 0.0345 | 0.5757 | 0.1945 | 0.029% | 97.941% |
| 154 | Eupolymnia | 0.1686 | 0.1999 | 0.2398 | 0.1388 | 0.1868 | 0.028% | 97.969% |
| 155 | Cossura | 0.0000 | 0.0671 | 0.0571 | 0.6161 | 0.1851 | 0.028% | 97.996% |
| 156 | Glycera americana | 0.1418 | 0.1780 | 0.2988 | 0.1046 | 0.1808 | 0.027% | 98.023% |
| 157 | Amphicteis gunneri | 0.0842 | 0.3171 | 0.2551 | 0.0455 | 0.1755 | 0.026% | 98.050% |
| 158 | Turbonilla | 0.0335 | 0.0000 | 0.2764 | 0.3790 | 0.1722 | 0.026% | 98.075% |
| 159 | Spionidae | 0.1551 | 0.2911 | 0.1696 | 0.0683 | 0.1710 | 0.026% | 98.101% |
| 160 | Polinices duplicatus | 0.0359 | 0.1082 | 0.1015 | 0.4328 | 0.1696 | 0.025% | 98.126% |
| 161 | Panopeidae | 0.3034 | 0.0997 | 0.2053 | 0.0696 | 0.1695 | 0.025% | 98.152% |
| 162 | Pinnixa | 0.1004 | 0.1329 | 0.3762 | 0.0683 | 0.1695 | 0.025% | 98.177% |
| 163 | Pectinaria | 0.0327 | 0.0997 | 0.1028 | 0.4328 | 0.1670 | 0.025% | 98.202% |
| 164 | Nereidae | 0.1692 | 0.2338 | 0.2037 | 0.0362 | 0.1607 | 0.024% | 98.226% |
| 165 | Ervilia concentrica | 0.2689 | 0.1329 | 0.2402 | 0.0000 | 0.1605 | 0.024% | 98.250% |
| 166 | Synidotea | 0.4052 | 0.1670 | 0.0687 | 0.0000 | 0.1602 | 0.024% | 98.274% |
| 167 | Callianassidae | 0.3377 | 0.1656 | 0.1358 | 0.0000 | 0.1598 | 0.024% | 98.297% |
| 168 | Diopatra | 0.2021 | 0.3321 | 0.1025 | 0.0000 | 0.1592 | 0.024% | 98.321% |
| 169 | Polinices | 0.2695 | 0.2318 | 0.1020 | 0.0333 | 0.1592 | 0.024% | 98.345% |
| 170 | Nuculana concentrica | 0.0000 | 0.0997 | 0.0342 | 0.4982 | 0.1580 | 0.024% | 98.369% |
| 171 | Ascidiacea | 0.5315 | 0.1000 | 0.0000 | 0.0000 | 0.1579 | 0.024% | 98.392% |
| 172 | Neritidae | 0.0000 | 0.0997 | 0.0687 | 0.4566 | 0.1562 | 0.023% | 98.415% |
| 173 | Cymadusa compta | 0.3382 | 0.1667 | 0.1025 | 0.0000 | 0.1518 | 0.023% | 98.438% |
| 174 | Odostomia gibbossa | 0.1004 | 0.1274 | 0.1929 | 0.1731 | 0.1484 | 0.022% | 98.460% |
| 175 | Enchytraeidae | 0.5409 | 0.0335 | 0.0000 | 0.0000 | 0.1436 | 0.021% | 98.482% |
| 176 | Phyllodocidae | 0.1351 | 0.3328 | 0.0687 | 0.0362 | 0.1432 | 0.021% | 98.503% |
| 177 | Arcuatula papyria | 0.1680 | 0.2993 | 0.0000 | 0.1046 | 0.1430 | 0.021% | 98.524% |
| 178 | Tricladida | 0.0338 | 0.2676 | 0.1708 | 0.0725 | 0.1362 | 0.020% | 98.545% |
| 179 | Harpacticoida | 0.1012 | 0.1332 | 0.2392 | 0.0704 | 0.1360 | 0.020% | 98.565% |
| 180 | Gammarus mucronatus | 0.0335 | 0.1329 | 0.3433 | 0.0342 | 0.1360 | 0.020% | 98.585% |
| 181 | Palaemonetes | 0.0332 | 0.3296 | 0.1025 | 0.0725 | 0.1345 | 0.020% | 98.605% |
| 182 | Portunidae | 0.2681 | 0.1555 | 0.0683 | 0.0342 | 0.1315 | 0.020% | 98.625% |
| 183 | Amphilbalanus amphitrite | 0.0000 | 0.1993 | 0.1025 | 0.2112 | 0.1283 | 0.019% | 98.644% |
| 184 | Polydora websteri | 0.0000 | 0.0324 | 0.0000 | 0.4711 | 0.1259 | 0.019% | 98.663% |
| 185 | Ogyrides | 0.0000 | 0.0332 | 0.0342 | 0.4245 | 0.1230 | 0.018% | 98.681% |
| 186 | Rithropanopeus harrisii | 0.0338 | 0.0997 | 0.0683 | 0.2899 | 0.1229 | 0.018% | 98.700% |
| 187 | Cerebratulus | 0.0094 | 0.0695 | 0.2532 | 0.1524 | 0.1211 | 0.018% | 98.718% |
| 188 | Eteone | 0.3710 | 0.0000 | 0.0024 | 0.1087 | 0.1205 | 0.018% | 98.736% |
| 189 | Schistomeringos rudolphi | 0.1686 | 0.0671 | 0.1715 | 0.0704 | 0.1194 | 0.018% | 98.754% |
| 190 | Tellina texana | 0.1338 | 0.1319 | 0.0690 | 0.1377 | 0.1181 | 0.018% | 98.771% |
| 191 | Odostomia acutidens | 0.0000 | 0.2325 | 0.0683 | 0.1708 | 0.1179 | 0.018% | 98.789% |
| 192 | Gammaridae | 0.0281 | 0.0257 | 0.3033 | 0.0983 | 0.1139 | 0.017% | 98.806% |
| 193 | Tanypodinae | 0.1645 | 0.0882 | 0.0821 | 0.1141 | 0.1122 | 0.017% | 98.823% |
| 194 | Ophiuroidea | 0.0674 | 0.0664 | 0.1712 | 0.1429 | 0.1120 | 0.017% | 98.839% |
| 195 | Scoloplos | 0.0000 | 0.1003 | 0.0683 | 0.2754 | 0.1110 | 0.017% | 98.856% |
| 196 | Gastropoda | 0.1009 | 0.1329 | 0.0000 | 0.2070 | 0.1102 | 0.016% | 98.872% |
| 197 | Axiothella mucosa | 0.0000 | 0.0332 | 0.1373 | 0.2516 | 0.1055 | 0.016% | 98.888% |
| 198 | Sigambra | 0.3634 | 0.0000 | 0.0000 | 0.0571 | 0.1051 | 0.016% | 98.904% |
| 199 | Edotea montosa | 0.0327 | 0.3201 | 0.0667 | 0.0000 | 0.1049 | 0.016% | 98.919% |
| 200 | Crepidula plana | 0.0000 | 0.0332 | 0.0345 | 0.3501 | 0.1045 | 0.016% | 98.935% |
| 201 | Anachis obesa | 0.0433 | 0.0000 | 0.0190 | 0.3492 | 0.1029 | 0.015% | 98.950% |
| 202 | Bowerbankia gracilis | 0.1012 | 0.1000 | 0.1373 | 0.0704 | 0.1022 | 0.015% | 98.966% |
| 203 | Hydroidolina | 0.0671 | 0.2325 | 0.1025 | 0.0000 | 0.1005 | 0.015% | 98.981% |
| 204 | Melita | 0.0000 | 0.0000 | 0.0000 | 0.3986 | 0.0997 | 0.015% | 98.996% |
| 205 | Mitrella lunata | 0.0654 | 0.0324 | 0.1006 | 0.1872 | 0.0964 | 0.014% | 99.010% |
| 206 | Micropholis atra | 0.0332 | 0.0335 | 0.0000 | 0.3137 | 0.0951 | 0.014% | 99.024% |
| 207 | Pyrogocythara plicosa | 0.0000 | 0.0997 | 0.1632 | 0.0805 | 0.0858 | 0.013% | 99.037% |
| 208 | Lepidophthalmus louisianaensis | 0.0000 | 0.0332 | 0.1028 | 0.2071 | 0.0858 | 0.013% | 99.050% |
| 209 | Paraprionospio | 0.1677 | 0.0332 | 0.1025 | 0.0354 | 0.0847 | 0.013% | 99.062% |
| 210 | Obelia dichotoma | 0.2018 | 0.0671 | 0.0342 | 0.0342 | 0.0843 | 0.013% | 99.075% |
| 211 | Cardiomya | 0.1348 | 0.1329 | 0.0687 | 0.0000 | 0.0841 | 0.013% | 99.088% |
| 212 | Apocorophium lacustre | 0.0000 | 0.1996 | 0.1367 | 0.0000 | 0.0841 | 0.013% | 99.100% |
| 213 | Hobsonia florida | 0.0000 | 0.0332 | 0.1370 | 0.1429 | 0.0783 | 0.012% | 99.112% |
| 214 | Marphysa | 0.0000 | 0.1329 | 0.1032 | 0.0725 | 0.0771 | 0.012% | 99.123% |
| 215 | Ceratonereis | 0.0677 | 0.0000 | 0.2043 | 0.0362 | 0.0770 | 0.012% | 99.135% |
| 216 | Dyspanopeus texanus | 0.0000 | 0.0668 | 0.1025 | 0.1067 | 0.0690 | 0.010% | 99.145% |
| 217 | Angulus merus | 0.0671 | 0.0671 | 0.0687 | 0.0725 | 0.0688 | 0.010% | 99.155% |
| 218 | Nereis grayi | 0.1343 | 0.0000 | 0.0000 | 0.1386 | 0.0682 | 0.010% | 99.166% |
| 219 | Mysidae | 0.0671 | 0.0664 | 0.0675 | 0.0716 | 0.0682 | 0.010% | 99.176% |
| 220 | Geukensia granosissima | 0.1348 | 0.0332 | 0.1025 | 0.0000 | 0.0676 | 0.010% | 99.186% |
| 221 | Parasabella microphthalma | 0.1015 | 0.1000 | 0.0687 | 0.0000 | 0.0675 | 0.010% | 99.196% |
| 222 | Physella | 0.2697 | 0.0000 | 0.0000 | 0.0000 | 0.0674 | 0.010% | 99.206% |
| 223 | Amathia gracilis | 0.0335 | 0.0988 | 0.1028 | 0.0333 | 0.0671 | 0.010% | 99.216% |
| 224 | Orbiniidae | 0.0000 | 0.2065 | 0.0167 | 0.0333 | 0.0641 | 0.010% | 99.226% |
| 225 | Crassostrea virginica | 0.1014 | 0.0335 | 0.0000 | 0.1067 | 0.0604 | 0.009% | 99.235% |
| 226 | Sesarma | 0.1001 | 0.0000 | 0.0667 | 0.0732 | 0.0600 | 0.009% | 99.244% |
| 227 | Periploma margaritaceum | 0.0000 | 0.0332 | 0.2048 | 0.0000 | 0.0595 | 0.009% | 99.252% |
| 228 | Astyris lunata | 0.0677 | 0.0997 | 0.0687 | 0.0000 | 0.0590 | 0.009% | 99.261% |
| 229 | Paranais literalis | 0.0664 | 0.1664 | 0.0000 | 0.0000 | 0.0582 | 0.009% | 99.270% |
| 230 | Amygdalum | 0.1686 | 0.0186 | 0.0262 | 0.0167 | 0.0575 | 0.009% | 99.279% |
| 231 | Eulimastoma weberi | 0.0000 | 0.0332 | 0.0683 | 0.1067 | 0.0521 | 0.008% | 99.286% |
| 232 | Tornidae | 0.0000 | 0.0000 | 0.1712 | 0.0362 | 0.0519 | 0.008% | 99.294% |
| 233 | Cerithidea | 0.0000 | 0.0996 | 0.0342 | 0.0725 | 0.0516 | 0.008% | 99.302% |
| 234 | Ostracoda | 0.0000 | 0.0000 | 0.0342 | 0.1708 | 0.0513 | 0.008% | 99.309% |
| 235 | Americamysis bahia | 0.0000 | 0.1000 | 0.0687 | 0.0362 | 0.0512 | 0.008% | 99.317% |
| 236 | Alcyonidum | 0.0674 | 0.0000 | 0.1367 | 0.0000 | 0.0510 | 0.008% | 99.325% |
| 237 | Alcyonidium polydoum | 0.0000 | 0.0664 | 0.0687 | 0.0683 | 0.0509 | 0.008% | 99.332% |
| 238 | Armandia maculata | 0.0332 | 0.0332 | 0.1367 | 0.0000 | 0.0508 | 0.008% | 99.340% |
| 239 | Chlamydopleon dissimile | 0.0664 | 0.0000 | 0.1367 | 0.0000 | 0.0508 | 0.008% | 99.347% |
| 240 | Onuphidae | 0.0664 | 0.0000 | 0.0683 | 0.0683 | 0.0508 | 0.008% | 99.355% |
| 241 | Corophium acherusicum | 0.2028 | 0.0000 | 0.0000 | 0.0000 | 0.0507 | 0.008% | 99.363% |
| 242 | Jassa falcata | 0.0000 | 0.1647 | 0.0333 | 0.0000 | 0.0495 | 0.007% | 99.370% |
| 243 | Isopoda | 0.1016 | 0.0255 | 0.0286 | 0.0362 | 0.0480 | 0.007% | 99.377% |
| 244 | Polydora ligni | 0.0566 | 0.0995 | 0.0342 | 0.0000 | 0.0476 | 0.007% | 99.384% |
| 245 | Cyclostremella humilus | 0.0259 | 0.0000 | 0.1024 | 0.0565 | 0.0462 | 0.007% | 99.391% |
| 246 | Gonida | 0.0338 | 0.0000 | 0.1485 | 0.0000 | 0.0456 | 0.007% | 99.398% |
| 247 | Caecum pulchellum | 0.0000 | 0.0000 | 0.0619 | 0.1167 | 0.0446 | 0.007% | 99.405% |
| 248 | Polypedilum | 0.0332 | 0.0335 | 0.0345 | 0.0725 | 0.0434 | 0.006% | 99.411% |
| 249 | Loimia | 0.0000 | 0.0000 | 0.1373 | 0.0362 | 0.0434 | 0.006% | 99.418% |
| 250 | Sayella | 0.0000 | 0.0000 | 0.0697 | 0.1037 | 0.0434 | 0.006% | 99.424% |
| 251 | Apocorophium | 0.0332 | 0.0000 | 0.1035 | 0.0362 | 0.0432 | 0.006% | 99.430% |
| 252 | Maldanidae | 0.0000 | 0.0335 | 0.0345 | 0.1046 | 0.0432 | 0.006% | 99.437% |
| 253 | Zoobotryon verticillatum | 0.0000 | 0.0332 | 0.0687 | 0.0704 | 0.0431 | 0.006% | 99.443% |
| 254 | Phoronis | 0.0000 | 0.0332 | 0.1028 | 0.0362 | 0.0431 | 0.006% | 99.450% |
| 255 | Bowmaniella | 0.0000 | 0.0000 | 0.1712 | 0.0000 | 0.0428 | 0.006% | 99.456% |
| 256 | Clytia cylindrica | 0.0000 | 0.0332 | 0.1370 | 0.0000 | 0.0426 | 0.006% | 99.463% |
| 257 | Eudendrium | 0.0664 | 0.0332 | 0.0000 | 0.0704 | 0.0425 | 0.006% | 99.469% |
| 258 | Neverita duplicata | 0.0332 | 0.0335 | 0.1025 | 0.0000 | 0.0423 | 0.006% | 99.475% |
| 259 | Prionospio | 0.0000 | 0.0332 | 0.1358 | 0.0000 | 0.0423 | 0.006% | 99.481% |
| 260 | Coelotanypus | 0.1006 | 0.0000 | 0.0683 | 0.0000 | 0.0422 | 0.006% | 99.488% |
| 261 | Paranaitis gardineri | 0.0332 | 0.0664 | 0.0342 | 0.0342 | 0.0420 | 0.006% | 99.494% |
| 262 | Pyconogonidae | 0.0000 | 0.1329 | 0.0342 | 0.0000 | 0.0418 | 0.006% | 99.500% |
| 263 | Bowmaniella dissimilis | 0.0674 | 0.0997 | 0.0000 | 0.0000 | 0.0418 | 0.006% | 99.507% |
| 264 | Callinectes sapidus | 0.0669 | 0.0656 | 0.0342 | 0.0000 | 0.0417 | 0.006% | 99.513% |
| 265 | Hyalella | 0.0770 | 0.0000 | 0.0438 | 0.0458 | 0.0416 | 0.006% | 99.519% |
| 266 | Hydroptilidae | 0.0556 | 0.0324 | 0.0000 | 0.0606 | 0.0371 | 0.006% | 99.525% |
| 267 | Grapsidae | 0.0792 | 0.0332 | 0.0345 | 0.0000 | 0.0367 | 0.005% | 99.530% |
| 268 | Teinostoma lerema | 0.0093 | 0.0255 | 0.1119 | 0.0000 | 0.0367 | 0.005% | 99.535% |
| 269 | Ficopomatus miamiensis | 0.0000 | 0.0000 | 0.0000 | 0.1450 | 0.0362 | 0.005% | 99.541% |
| 270 | Syllidae | 0.0338 | 0.0000 | 0.0333 | 0.0732 | 0.0351 | 0.005% | 99.546% |
| 271 | Gammaroidea | 0.0000 | 0.0000 | 0.1025 | 0.0362 | 0.0347 | 0.005% | 99.551% |
| 272 | Pagurus | 0.0000 | 0.0000 | 0.0342 | 0.1046 | 0.0347 | 0.005% | 99.556% |
| 273 | Stenoninereis martini | 0.0000 | 0.0000 | 0.0000 | 0.1388 | 0.0347 | 0.005% | 99.562% |
| 274 | Branchiura | 0.0338 | 0.0000 | 0.0342 | 0.0705 | 0.0346 | 0.005% | 99.567% |
| 275 | Cyrenoida floridana | 0.0000 | 0.0332 | 0.0345 | 0.0704 | 0.0345 | 0.005% | 99.572% |
| 276 | Tubulanus | 0.0000 | 0.0000 | 0.1380 | 0.0000 | 0.0345 | 0.005% | 99.577% |
| 277 | Rhithropanopeus harrisii | 0.1015 | 0.0000 | 0.0000 | 0.0362 | 0.0344 | 0.005% | 99.582% |
| 278 | Magelona pettiboneae | 0.0000 | 0.0000 | 0.1373 | 0.0000 | 0.0343 | 0.005% | 99.587% |
| 279 | Anachis | 0.0000 | 0.0000 | 0.0000 | 0.1367 | 0.0342 | 0.005% | 99.593% |
| 280 | Teinostoma megastoma | 0.0000 | 0.0000 | 0.1367 | 0.0000 | 0.0342 | 0.005% | 99.598% |
| 281 | Eteone foliosa | 0.0000 | 0.0332 | 0.1028 | 0.0000 | 0.0340 | 0.005% | 99.603% |
| 282 | Truncatella caribaecnsis | 0.0000 | 0.0000 | 0.1027 | 0.0333 | 0.0340 | 0.005% | 99.608% |
| 283 | Leptochela | 0.0338 | 0.0332 | 0.0683 | 0.0000 | 0.0339 | 0.005% | 99.613% |
| 284 | Cirratulidae | 0.1019 | 0.0332 | 0.0000 | 0.0000 | 0.0338 | 0.005% | 99.618% |
| 285 | Crepidula depressa | 0.0000 | 0.1006 | 0.0345 | 0.0000 | 0.0338 | 0.005% | 99.623% |
| 286 | Dipolydora socialis | 0.0000 | 0.0664 | 0.0683 | 0.0000 | 0.0337 | 0.005% | 99.628% |
| 287 | Epitonium apiculatum | 0.1342 | 0.0000 | 0.0000 | 0.0000 | 0.0336 | 0.005% | 99.633% |
| 288 | Myrophis punctatus | 0.0335 | 0.0664 | 0.0342 | 0.0000 | 0.0335 | 0.005% | 99.638% |
| 289 | Arenicola | 0.0330 | 0.0998 | 0.0000 | 0.0000 | 0.0332 | 0.005% | 99.643% |
| 290 | Spio | 0.0324 | 0.0324 | 0.0670 | 0.0000 | 0.0330 | 0.005% | 99.648% |
| 291 | Epitonium | 0.0432 | 0.0093 | 0.0095 | 0.0669 | 0.0322 | 0.005% | 99.653% |
| 292 | Nectonema | 0.0604 | 0.0000 | 0.0000 | 0.0626 | 0.0307 | 0.005% | 99.657% |
| 293 | Cassidinidea ovalis | 0.0000 | 0.0000 | 0.0000 | 0.1087 | 0.0272 | 0.004% | 99.661% |
| 294 | Travisia hobsonae | 0.0000 | 0.0000 | 0.0024 | 0.1060 | 0.0271 | 0.004% | 99.665% |
| 295 | Malacoceros | 0.0000 | 0.0000 | 0.0000 | 0.1067 | 0.0267 | 0.004% | 99.669% |
| 296 | Diplothyra smythi | 0.0330 | 0.0000 | 0.0000 | 0.0725 | 0.0264 | 0.004% | 99.673% |
| 297 | Hydroides dianthus | 0.0338 | 0.0000 | 0.0000 | 0.0704 | 0.0261 | 0.004% | 99.677% |
| 298 | Mesogastropoda | 0.0000 | 0.0000 | 0.0697 | 0.0333 | 0.0258 | 0.004% | 99.681% |
| 299 | Sigambra bassi | 0.0000 | 0.0332 | 0.0697 | 0.0000 | 0.0257 | 0.004% | 99.685% |
| 300 | Drilonereis | 0.0000 | 0.0324 | 0.0342 | 0.0362 | 0.0257 | 0.004% | 99.689% |
| 301 | Laevicardium mortoni | 0.0000 | 0.0335 | 0.0687 | 0.0000 | 0.0255 | 0.004% | 99.692% |
| 302 | Crepidula convexa | 0.0000 | 0.0000 | 0.0000 | 0.1020 | 0.0255 | 0.004% | 99.696% |
| 303 | Aceton candens | 0.0332 | 0.0000 | 0.0687 | 0.0000 | 0.0255 | 0.004% | 99.700% |
| 304 | Nothria | 0.0000 | 0.0335 | 0.0683 | 0.0000 | 0.0255 | 0.004% | 99.704% |
| 305 | Terebellidae | 0.0332 | 0.0000 | 0.0683 | 0.0000 | 0.0254 | 0.004% | 99.708% |
| 306 | Gitanopsis laguna | 0.1015 | 0.0000 | 0.0000 | 0.0000 | 0.0254 | 0.004% | 99.711% |
| 307 | Berosus | 0.0677 | 0.0000 | 0.0337 | 0.0000 | 0.0253 | 0.004% | 99.715% |
| 308 | Notomastus | 0.0332 | 0.0335 | 0.0342 | 0.0000 | 0.0252 | 0.004% | 99.719% |
| 309 | Hourstonius laguna | 0.0000 | 0.0332 | 0.0342 | 0.0333 | 0.0252 | 0.004% | 99.723% |
| 310 | Pinnixa pearsei | 0.0000 | 0.0332 | 0.0342 | 0.0333 | 0.0252 | 0.004% | 99.727% |
| 311 | Maldane sarsi | 0.0000 | 0.0664 | 0.0342 | 0.0000 | 0.0252 | 0.004% | 99.730% |
| 312 | Paranaitis speciosa | 0.0000 | 0.0664 | 0.0000 | 0.0342 | 0.0252 | 0.004% | 99.734% |
| 313 | Rangia flexuosa | 0.0000 | 0.0664 | 0.0342 | 0.0000 | 0.0252 | 0.004% | 99.738% |
| 314 | Megalops Unid | 0.0674 | 0.0332 | 0.0000 | 0.0000 | 0.0251 | 0.004% | 99.742% |
| 315 | Nereis tridentata | 0.0671 | 0.0335 | 0.0000 | 0.0000 | 0.0251 | 0.004% | 99.745% |
| 316 | Ceratonereis mirabilis | 0.0000 | 0.0000 | 0.0000 | 0.0939 | 0.0235 | 0.004% | 99.749% |
| 317 | Callianassa atlantica | 0.0000 | 0.0324 | 0.0571 | 0.0000 | 0.0224 | 0.003% | 99.752% |
| 318 | Micropanope sculptipes | 0.0000 | 0.0000 | 0.0096 | 0.0732 | 0.0207 | 0.003% | 99.755% |
| 319 | Psectrotanypus | 0.0280 | 0.0093 | 0.0000 | 0.0439 | 0.0203 | 0.003% | 99.758% |
| 320 | Triphora | 0.0000 | 0.0000 | 0.0762 | 0.0000 | 0.0190 | 0.003% | 99.761% |
| 321 | Atyidae | 0.0400 | 0.0358 | 0.0000 | 0.0000 | 0.0189 | 0.003% | 99.764% |
| 322 | Americamysis alleni | 0.0000 | 0.0000 | 0.0000 | 0.0725 | 0.0181 | 0.003% | 99.767% |
| 323 | Boonea impressa | 0.0000 | 0.0000 | 0.0000 | 0.0725 | 0.0181 | 0.003% | 99.769% |
| 324 | Corbula | 0.0000 | 0.0000 | 0.0000 | 0.0725 | 0.0181 | 0.003% | 99.772% |
| 325 | Ficopmatus | 0.0000 | 0.0000 | 0.0000 | 0.0725 | 0.0181 | 0.003% | 99.775% |
| 326 | Malmgreniella taylori | 0.0000 | 0.0000 | 0.0000 | 0.0725 | 0.0181 | 0.003% | 99.777% |
| 327 | Naididae | 0.0000 | 0.0000 | 0.0000 | 0.0725 | 0.0181 | 0.003% | 99.780% |
| 328 | Turbonilla aequalis | 0.0425 | 0.0093 | 0.0167 | 0.0025 | 0.0177 | 0.003% | 99.783% |
| 329 | Leitoscoloplos | 0.0000 | 0.0000 | 0.0345 | 0.0362 | 0.0177 | 0.003% | 99.785% |
| 330 | Mysidopsis almyra | 0.0000 | 0.0000 | 0.0345 | 0.0362 | 0.0177 | 0.003% | 99.788% |
| 331 | Stylactis | 0.0000 | 0.0000 | 0.0345 | 0.0362 | 0.0177 | 0.003% | 99.791% |
| 332 | Cliona celata | 0.0338 | 0.0000 | 0.0000 | 0.0362 | 0.0175 | 0.003% | 99.793% |
| 333 | Diadora cayenensis | 0.0000 | 0.0000 | 0.0000 | 0.0690 | 0.0173 | 0.003% | 99.796% |
| 334 | Rangianella flexuosa | 0.0000 | 0.0000 | 0.0690 | 0.0000 | 0.0172 | 0.003% | 99.799% |
| 335 | Capitellida | 0.0000 | 0.0000 | 0.0687 | 0.0000 | 0.0172 | 0.003% | 99.801% |
| 336 | Chione elevata | 0.0000 | 0.0000 | 0.0687 | 0.0000 | 0.0172 | 0.003% | 99.804% |
| 337 | Hesionidae | 0.0000 | 0.0000 | 0.0687 | 0.0000 | 0.0172 | 0.003% | 99.806% |
| 338 | Hippolytidae | 0.0000 | 0.0000 | 0.0345 | 0.0342 | 0.0172 | 0.003% | 99.809% |
| 339 | Maldane | 0.0000 | 0.0000 | 0.0687 | 0.0000 | 0.0172 | 0.003% | 99.811% |
| 340 | Probezzia | 0.0000 | 0.0000 | 0.0687 | 0.0000 | 0.0172 | 0.003% | 99.814% |
| 341 | Semele nuculoides | 0.0000 | 0.0000 | 0.0687 | 0.0000 | 0.0172 | 0.003% | 99.816% |
| 342 | Carazziella hobsonae | 0.0000 | 0.0000 | 0.0683 | 0.0000 | 0.0171 | 0.003% | 99.819% |
| 343 | Edwardsia | 0.0000 | 0.0000 | 0.0683 | 0.0000 | 0.0171 | 0.003% | 99.822% |
| 344 | Eudendriidae | 0.0000 | 0.0000 | 0.0342 | 0.0342 | 0.0171 | 0.003% | 99.824% |
| 345 | Zebina | 0.0000 | 0.0000 | 0.0000 | 0.0683 | 0.0171 | 0.003% | 99.827% |
| 346 | Lovenella | 0.0332 | 0.0000 | 0.0345 | 0.0000 | 0.0169 | 0.003% | 99.829% |
| 347 | Zygonemertes virescens | 0.0000 | 0.0335 | 0.0342 | 0.0000 | 0.0169 | 0.003% | 99.832% |
| 348 | Geukensia demissa | 0.0677 | 0.0000 | 0.0000 | 0.0000 | 0.0169 | 0.003% | 99.834% |
| 349 | Hemiptera | 0.0677 | 0.0000 | 0.0000 | 0.0000 | 0.0169 | 0.003% | 99.837% |
| 350 | Hexagenia | 0.0000 | 0.0000 | 0.0675 | 0.0000 | 0.0169 | 0.003% | 99.839% |
| 351 | Batea | 0.0338 | 0.0000 | 0.0337 | 0.0000 | 0.0169 | 0.003% | 99.842% |
| 352 | Insecta | 0.0332 | 0.0000 | 0.0342 | 0.0000 | 0.0168 | 0.003% | 99.844% |
| 353 | Scolelepis | 0.0338 | 0.0332 | 0.0000 | 0.0000 | 0.0168 | 0.003% | 99.847% |
| 354 | Capitellides jonesi | 0.0000 | 0.0671 | 0.0000 | 0.0000 | 0.0168 | 0.003% | 99.849% |
| 355 | Teinostoma | 0.0669 | 0.0000 | 0.0000 | 0.0000 | 0.0167 | 0.002% | 99.852% |
| 356 | Nudibranchia | 0.0332 | 0.0335 | 0.0000 | 0.0000 | 0.0167 | 0.002% | 99.854% |
| 357 | Pyrgocythara plicosa | 0.0000 | 0.0000 | 0.0333 | 0.0333 | 0.0167 | 0.002% | 99.857% |
| 358 | Hexapanopeus | 0.0332 | 0.0000 | 0.0333 | 0.0000 | 0.0166 | 0.002% | 99.859% |
| 359 | Nematonereis | 0.0000 | 0.0664 | 0.0000 | 0.0000 | 0.0166 | 0.002% | 99.862% |
| 360 | Obelia | 0.0332 | 0.0332 | 0.0000 | 0.0000 | 0.0166 | 0.002% | 99.864% |
| 361 | Salvatoria clavata | 0.0000 | 0.0664 | 0.0000 | 0.0000 | 0.0166 | 0.002% | 99.867% |
| 362 | Scolilipis squamata | 0.0000 | 0.0664 | 0.0000 | 0.0000 | 0.0166 | 0.002% | 99.869% |
| 363 | Spilocuma | 0.0000 | 0.0664 | 0.0000 | 0.0000 | 0.0166 | 0.002% | 99.872% |
| 364 | Coleoptera | 0.0659 | 0.0000 | 0.0000 | 0.0000 | 0.0165 | 0.002% | 99.874% |
| 365 | Arthropoda | 0.0330 | 0.0327 | 0.0000 | 0.0000 | 0.0164 | 0.002% | 99.877% |
| 366 | Mysidacea | 0.0117 | 0.0417 | 0.0000 | 0.0101 | 0.0159 | 0.002% | 99.879% |
| 367 | Ceratopogonidae | 0.0000 | 0.0000 | 0.0620 | 0.0000 | 0.0155 | 0.002% | 99.881% |
| 368 | Ninoe nigripes | 0.0000 | 0.0000 | 0.0000 | 0.0571 | 0.0143 | 0.002% | 99.883% |
| 369 | Crepidula fornicata | 0.0556 | 0.0000 | 0.0000 | 0.0000 | 0.0139 | 0.002% | 99.885% |
| 370 | Clinotanypus | 0.0116 | 0.0394 | 0.0024 | 0.0000 | 0.0133 | 0.002% | 99.887% |
| 371 | Ceratium | 0.0000 | 0.0000 | 0.0263 | 0.0262 | 0.0131 | 0.002% | 99.889% |
| 372 | Penaeus | 0.0332 | 0.0000 | 0.0000 | 0.0167 | 0.0125 | 0.002% | 99.891% |
| 373 | Pectinariidae | 0.0338 | 0.0093 | 0.0000 | 0.0000 | 0.0108 | 0.002% | 99.893% |
| 374 | Eulimastoma | 0.0000 | 0.0093 | 0.0337 | 0.0000 | 0.0107 | 0.002% | 99.894% |
| 375 | Nuculana | 0.0000 | 0.0000 | 0.0000 | 0.0429 | 0.0107 | 0.002% | 99.896% |
| 376 | Aegathoa | 0.0000 | 0.0000 | 0.0000 | 0.0362 | 0.0091 | 0.001% | 99.897% |
| 377 | Araneidae | 0.0000 | 0.0000 | 0.0000 | 0.0362 | 0.0091 | 0.001% | 99.899% |
| 378 | Callianassa | 0.0000 | 0.0000 | 0.0000 | 0.0362 | 0.0091 | 0.001% | 99.900% |
| 379 | Cliona | 0.0000 | 0.0000 | 0.0000 | 0.0362 | 0.0091 | 0.001% | 99.902% |
| 380 | Dugesia | 0.0000 | 0.0000 | 0.0000 | 0.0362 | 0.0091 | 0.001% | 99.903% |
| 381 | Marphysa sanguinea | 0.0000 | 0.0000 | 0.0000 | 0.0362 | 0.0091 | 0.001% | 99.904% |
| 382 | Eteone longa | 0.0093 | 0.0000 | 0.0262 | 0.0000 | 0.0089 | 0.001% | 99.906% |
| 383 | Caprellidae | 0.0000 | 0.0000 | 0.0345 | 0.0000 | 0.0086 | 0.001% | 99.907% |
| 384 | Chama congregata | 0.0000 | 0.0000 | 0.0345 | 0.0000 | 0.0086 | 0.001% | 99.908% |
| 385 | Heteromastus califoriensis | 0.0000 | 0.0000 | 0.0345 | 0.0000 | 0.0086 | 0.001% | 99.909% |
| 386 | Hydroides | 0.0000 | 0.0000 | 0.0345 | 0.0000 | 0.0086 | 0.001% | 99.911% |
| 387 | Magelona polydentata | 0.0000 | 0.0000 | 0.0345 | 0.0000 | 0.0086 | 0.001% | 99.912% |
| 388 | Owenia | 0.0000 | 0.0000 | 0.0345 | 0.0000 | 0.0086 | 0.001% | 99.913% |
| 389 | Pista quadrilobata | 0.0000 | 0.0000 | 0.0345 | 0.0000 | 0.0086 | 0.001% | 99.915% |
| 390 | Callinectes | 0.0000 | 0.0000 | 0.0000 | 0.0342 | 0.0085 | 0.001% | 99.916% |
| 391 | Campanulariidae | 0.0000 | 0.0000 | 0.0342 | 0.0000 | 0.0085 | 0.001% | 99.917% |
| 392 | Carveia | 0.0000 | 0.0000 | 0.0342 | 0.0000 | 0.0085 | 0.001% | 99.918% |
| 393 | Cyclostremiscus | 0.0000 | 0.0000 | 0.0342 | 0.0000 | 0.0085 | 0.001% | 99.920% |
| 394 | Diptera | 0.0000 | 0.0000 | 0.0000 | 0.0342 | 0.0085 | 0.001% | 99.921% |
| 395 | Eunicidae | 0.0000 | 0.0000 | 0.0000 | 0.0342 | 0.0085 | 0.001% | 99.922% |
| 396 | Megabalanus antillensis | 0.0000 | 0.0000 | 0.0342 | 0.0000 | 0.0085 | 0.001% | 99.923% |
| 397 | Neanthes acuminata | 0.0000 | 0.0000 | 0.0000 | 0.0342 | 0.0085 | 0.001% | 99.925% |
| 398 | Parandalia | 0.0000 | 0.0000 | 0.0342 | 0.0000 | 0.0085 | 0.001% | 99.926% |
| 399 | Paraprionospio alata | 0.0000 | 0.0000 | 0.0000 | 0.0342 | 0.0085 | 0.001% | 99.927% |
| 400 | Periploma orbiculare | 0.0000 | 0.0000 | 0.0342 | 0.0000 | 0.0085 | 0.001% | 99.929% |
| 401 | Pilargidae | 0.0000 | 0.0000 | 0.0000 | 0.0342 | 0.0085 | 0.001% | 99.930% |
| 402 | Amathia alternata | 0.0338 | 0.0000 | 0.0000 | 0.0000 | 0.0085 | 0.001% | 99.931% |
| 403 | Caridea | 0.0338 | 0.0000 | 0.0000 | 0.0000 | 0.0085 | 0.001% | 99.932% |
| 404 | Dorvillea | 0.0338 | 0.0000 | 0.0000 | 0.0000 | 0.0085 | 0.001% | 99.934% |
| 405 | Eteone lactea | 0.0338 | 0.0000 | 0.0000 | 0.0000 | 0.0085 | 0.001% | 99.935% |
| 406 | Galathowenia oculata | 0.0338 | 0.0000 | 0.0000 | 0.0000 | 0.0085 | 0.001% | 99.936% |
| 407 | Lumbrineridae | 0.0338 | 0.0000 | 0.0000 | 0.0000 | 0.0085 | 0.001% | 99.937% |
| 408 | Lumbrineriopsis paradoxa | 0.0338 | 0.0000 | 0.0000 | 0.0000 | 0.0085 | 0.001% | 99.939% |
| 409 | Spiophanes bombyx | 0.0338 | 0.0000 | 0.0000 | 0.0000 | 0.0085 | 0.001% | 99.940% |
| 410 | Tanaidacea | 0.0338 | 0.0000 | 0.0000 | 0.0000 | 0.0085 | 0.001% | 99.941% |
| 411 | Probythinella protera | 0.0000 | 0.0000 | 0.0337 | 0.0000 | 0.0084 | 0.001% | 99.942% |
| 412 | Bougainvillia inaequalis | 0.0000 | 0.0335 | 0.0000 | 0.0000 | 0.0084 | 0.001% | 99.944% |
| 413 | Emerita | 0.0335 | 0.0000 | 0.0000 | 0.0000 | 0.0084 | 0.001% | 99.945% |
| 414 | Penaeidae | 0.0335 | 0.0000 | 0.0000 | 0.0000 | 0.0084 | 0.001% | 99.946% |
| 415 | Penaeus setiferus | 0.0000 | 0.0335 | 0.0000 | 0.0000 | 0.0084 | 0.001% | 99.947% |
| 416 | Unsegmented worm | 0.0000 | 0.0335 | 0.0000 | 0.0000 | 0.0084 | 0.001% | 99.949% |
| 417 | Austromacoma constricta | 0.0000 | 0.0000 | 0.0333 | 0.0000 | 0.0083 | 0.001% | 99.950% |
| 418 | Cerithiopsis greeni | 0.0000 | 0.0000 | 0.0333 | 0.0000 | 0.0083 | 0.001% | 99.951% |
| 419 | Exosphaeroma diminutum | 0.0000 | 0.0000 | 0.0333 | 0.0000 | 0.0083 | 0.001% | 99.952% |
| 420 | Polinices neverita | 0.0000 | 0.0000 | 0.0333 | 0.0000 | 0.0083 | 0.001% | 99.954% |
| 421 | Sayella livida | 0.0000 | 0.0000 | 0.0333 | 0.0000 | 0.0083 | 0.001% | 99.955% |
| 422 | Seila adamsi | 0.0000 | 0.0000 | 0.0000 | 0.0333 | 0.0083 | 0.001% | 99.956% |
| 423 | Spiophanes | 0.0000 | 0.0000 | 0.0333 | 0.0000 | 0.0083 | 0.001% | 99.957% |
| 424 | Websterinereis tridentata | 0.0000 | 0.0000 | 0.0333 | 0.0000 | 0.0083 | 0.001% | 99.959% |
| 425 | Agriopoma texasina | 0.0000 | 0.0332 | 0.0000 | 0.0000 | 0.0083 | 0.001% | 99.960% |
| 426 | Autolytinae | 0.0000 | 0.0332 | 0.0000 | 0.0000 | 0.0083 | 0.001% | 99.961% |
| 427 | Chaoborus | 0.0000 | 0.0332 | 0.0000 | 0.0000 | 0.0083 | 0.001% | 99.962% |
| 428 | Chironomus | 0.0000 | 0.0332 | 0.0000 | 0.0000 | 0.0083 | 0.001% | 99.964% |
| 429 | Clibanarius vittatus | 0.0000 | 0.0332 | 0.0000 | 0.0000 | 0.0083 | 0.001% | 99.965% |
| 430 | Erichthoius brasilensis | 0.0000 | 0.0332 | 0.0000 | 0.0000 | 0.0083 | 0.001% | 99.966% |
| 431 | Leitoscoloplos foliosus | 0.0332 | 0.0000 | 0.0000 | 0.0000 | 0.0083 | 0.001% | 99.967% |
| 432 | Leptochelia dubia | 0.0000 | 0.0332 | 0.0000 | 0.0000 | 0.0083 | 0.001% | 99.969% |
| 433 | Microphthalmus sczelkowii | 0.0000 | 0.0332 | 0.0000 | 0.0000 | 0.0083 | 0.001% | 99.970% |
| 434 | Neopanope | 0.0000 | 0.0332 | 0.0000 | 0.0000 | 0.0083 | 0.001% | 99.971% |
| 435 | Ophiophragmus | 0.0000 | 0.0332 | 0.0000 | 0.0000 | 0.0083 | 0.001% | 99.972% |
| 436 | Pagurus longicarpus | 0.0000 | 0.0332 | 0.0000 | 0.0000 | 0.0083 | 0.001% | 99.974% |
| 437 | Palaemonetes paludosus | 0.0000 | 0.0332 | 0.0000 | 0.0000 | 0.0083 | 0.001% | 99.975% |
| 438 | Parvanachis obesa | 0.0000 | 0.0332 | 0.0000 | 0.0000 | 0.0083 | 0.001% | 99.976% |
| 439 | Phyllodoce arenae | 0.0332 | 0.0000 | 0.0000 | 0.0000 | 0.0083 | 0.001% | 99.977% |
| 440 | Tubificoides | 0.0000 | 0.0332 | 0.0000 | 0.0000 | 0.0083 | 0.001% | 99.979% |
| 441 | Uca | 0.0000 | 0.0332 | 0.0000 | 0.0000 | 0.0083 | 0.001% | 99.980% |
| 442 | Episcynia inornata | 0.0330 | 0.0000 | 0.0000 | 0.0000 | 0.0083 | 0.001% | 99.981% |
| 443 | Mytilopsis | 0.0330 | 0.0000 | 0.0000 | 0.0000 | 0.0083 | 0.001% | 99.982% |
| 444 | Neomediomastus glabrus | 0.0330 | 0.0000 | 0.0000 | 0.0000 | 0.0083 | 0.001% | 99.983% |
| 445 | Progomphus | 0.0330 | 0.0000 | 0.0000 | 0.0000 | 0.0083 | 0.001% | 99.985% |
| 446 | Psephenidae | 0.0330 | 0.0000 | 0.0000 | 0.0000 | 0.0083 | 0.001% | 99.986% |
| 447 | Tellina tampaensis | 0.0330 | 0.0000 | 0.0000 | 0.0000 | 0.0083 | 0.001% | 99.987% |
| 448 | Albinia cerithidioides | 0.0000 | 0.0327 | 0.0000 | 0.0000 | 0.0082 | 0.001% | 99.988% |
| 449 | Gomphus | 0.0327 | 0.0000 | 0.0000 | 0.0000 | 0.0082 | 0.001% | 99.990% |
| 450 | Mytilidae | 0.0327 | 0.0000 | 0.0000 | 0.0000 | 0.0082 | 0.001% | 99.991% |
| 451 | Anomura | 0.0000 | 0.0324 | 0.0000 | 0.0000 | 0.0081 | 0.001% | 99.992% |
| 452 | Crustacea | 0.0000 | 0.0324 | 0.0000 | 0.0000 | 0.0081 | 0.001% | 99.993% |
| 453 | Macropelopiini | 0.0210 | 0.0023 | 0.0000 | 0.0000 | 0.0058 | 0.001% | 99.994% |
| 454 | Procladius | 0.0093 | 0.0046 | 0.0024 | 0.0024 | 0.0047 | 0.001% | 99.995% |
| 455 | Alotanypus | 0.0000 | 0.0000 | 0.0000 | 0.0167 | 0.0042 | 0.001% | 99.995% |
| 456 | Podarkeopsis brevipalpa | 0.0000 | 0.0000 | 0.0167 | 0.0000 | 0.0042 | 0.001% | 99.996% |
| 457 | Cleotanypus | 0.0162 | 0.0000 | 0.0000 | 0.0000 | 0.0041 | 0.001% | 99.997% |
| 458 | Megaluropus | 0.0142 | 0.0000 | 0.0000 | 0.0000 | 0.0035 | 0.001% | 99.997% |
| 459 | Anachis avara | 0.0000 | 0.0000 | 0.0119 | 0.0000 | 0.0030 | 0.000% | 99.998% |
| 460 | Caecum johnsoni | 0.0000 | 0.0093 | 0.0024 | 0.0000 | 0.0029 | 0.000% | 99.998% |
| 461 | Parallorchestes | 0.0000 | 0.0000 | 0.0096 | 0.0000 | 0.0024 | 0.000% | 99.998% |
| 462 | Pectinaria moorei | 0.0000 | 0.0000 | 0.0095 | 0.0000 | 0.0024 | 0.000% | 99.999% |
| 463 | Arabellidae | 0.0000 | 0.0046 | 0.0000 | 0.0024 | 0.0018 | 0.000% | 99.999% |
| 464 | Sergestidae | 0.0000 | 0.0069 | 0.0000 | 0.0000 | 0.0017 | 0.000% | 99.999% |
| 465 | Chaoborus americana | 0.0000 | 0.0000 | 0.0048 | 0.0000 | 0.0012 | 0.000% | 99.999% |
| 466 | Eurylax nitida | 0.0000 | 0.0000 | 0.0024 | 0.0000 | 0.0006 | 0.000% | 100.000% |
| 467 | Acteon punctostrictus | 0.0000 | 0.0000 | 0.0024 | 0.0000 | 0.0006 | 0.000% | 100.000% |
| 468 | Ancylidae | 0.0000 | 0.0000 | 0.0024 | 0.0000 | 0.0006 | 0.000% | 100.000% |
| 469 | Hydrachnidia | 0.0000 | 0.0000 | 0.0024 | 0.0000 | 0.0006 | 0.000% | 100.000% |
| 470 | Nanocladius | 0.0000 | 0.0000 | 0.0024 | 0.0000 | 0.0006 | 0.000% | 100.000% |
| 471 | Amphiuridae | 0.0024 | 0.0000 | 0.0000 | 0.0000 | 0.0006 | 0.000% | 100.000% |
|  | **Total** | **506.3273** | **698.5312** | **821.4588** | **653.2143** | **669.8829** |  |  |

## Nekton in Gill Net Samples

Average abundance of nekton caught in gill net samples (n/24 h) over all sampling periods.

| **Rank** | **Taxa/Species Name** | **Distance From Discharge** | | | **Mean** | **Pct** | **CumPct** |
| --- | --- | --- | --- | --- | --- | --- | --- |
|  |  | **B** | **C** | **R** |  |  |  |
| 1 | Arius felis | 22.7023 | 30.1739 | 36.0345 | 29.6369 | 37.724% | 37.724% |
| 2 | Brevoortia patronus | 17.9886 | 17.7168 | 19.3571 | 18.3542 | 23.362% | 61.086% |
| 3 | Bagre marinus | 7.3305 | 9.8616 | 16.1434 | 11.1118 | 14.144% | 75.230% |
| 4 | Ictalurus furcatus | 2.5550 | 2.3551 | 4.0947 | 3.0016 | 3.821% | 79.051% |
| 5 | Leiostomus xanthurus | 2.3905 | 2.0736 | 1.9758 | 2.1466 | 2.732% | 81.783% |
| 6 | Menticirrhus americanus | 1.7760 | 1.8553 | 1.1302 | 1.5872 | 2.020% | 83.804% |
| 7 | Cynoscion arenarius | 1.3455 | 1.6710 | 1.6302 | 1.5489 | 1.972% | 85.775% |
| 8 | Cynoscion nebulosus | 1.4893 | 1.6226 | 1.3669 | 1.4929 | 1.900% | 87.676% |
| 9 | Dorosoma cepedianum | 1.4069 | 1.3302 | 1.0559 | 1.2643 | 1.609% | 89.285% |
| 10 | Rhinoptera bonasus | 0.6621 | 1.0006 | 0.9797 | 0.8808 | 1.121% | 90.406% |
| 11 | Callinectes sapidus | 0.9835 | 0.7933 | 0.8061 | 0.8610 | 1.096% | 91.502% |
| 12 | Pogonias cromis | 0.5397 | 0.6581 | 1.0626 | 0.7535 | 0.959% | 92.461% |
| 13 | Bairdiella chrysoura | 0.5506 | 0.7915 | 0.5741 | 0.6387 | 0.813% | 93.274% |
| 14 | Opisthonema oglinum | 0.8120 | 0.6405 | 0.3824 | 0.6116 | 0.779% | 94.053% |
| 15 | Dasyatis sabina | 0.5448 | 0.5883 | 0.2795 | 0.4709 | 0.599% | 94.652% |
| 16 | Mugil cephalus | 0.5639 | 0.4460 | 0.1666 | 0.3922 | 0.499% | 95.151% |
| 17 | Micropogonias undulatus | 0.4255 | 0.3293 | 0.3770 | 0.3773 | 0.480% | 95.631% |
| 18 | Carcharhinus leucas | 0.2048 | 0.3579 | 0.5253 | 0.3627 | 0.462% | 96.093% |
| 19 | Elops saurus | 0.5186 | 0.3796 | 0.1780 | 0.3587 | 0.457% | 96.550% |
| 20 | Lagodon rhomboides | 0.4877 | 0.3827 | 0.0950 | 0.3218 | 0.410% | 96.959% |
| 21 | Archosargus probatocephalus | 0.3804 | 0.3745 | 0.1494 | 0.3014 | 0.384% | 97.343% |
| 22 | Scomberomorus maculatus | 0.2408 | 0.3014 | 0.2525 | 0.2649 | 0.337% | 97.680% |
| 23 | Rhizoprionodon terraenova | 0.1926 | 0.1902 | 0.3391 | 0.2406 | 0.306% | 97.986% |
| 24 | Paralichthys lethostigma | 0.2499 | 0.2183 | 0.1940 | 0.2207 | 0.281% | 98.267% |
| 25 | Caranx hippos | 0.2336 | 0.2404 | 0.1784 | 0.2174 | 0.277% | 98.544% |
| 26 | Sciaenops ocellata | 0.0287 | 0.0767 | 0.1774 | 0.0943 | 0.120% | 98.664% |
| 27 | Trachinotus carolinus | 0.1309 | 0.0987 | 0.0393 | 0.0896 | 0.114% | 98.778% |
| 28 | Lepisosteus spatula | 0.0738 | 0.0623 | 0.1160 | 0.0840 | 0.107% | 98.885% |
| 29 | Penaeus setiferus | 0.0808 | 0.0839 | 0.0725 | 0.0791 | 0.101% | 98.986% |
| 30 | Alosa chrysochloris | 0.0739 | 0.0818 | 0.0643 | 0.0733 | 0.093% | 99.079% |
| 31 | Lepisosteus osseus | 0.0525 | 0.0745 | 0.0812 | 0.0694 | 0.088% | 99.167% |
| 32 | Sardenella anchovia | 0.0833 | 0.0670 | 0.0221 | 0.0575 | 0.073% | 99.241% |
| 33 | Menippe mercenaria | 0.0791 | 0.0790 | 0.0126 | 0.0569 | 0.072% | 99.313% |
| 34 | Dorosoma petenense | 0.0571 | 0.0647 | 0.0296 | 0.0504 | 0.064% | 99.377% |
| 35 | Sphyrna tiburo | 0.0357 | 0.0673 | 0.0403 | 0.0478 | 0.061% | 99.438% |
| 36 | Peprilus alepidotus | 0.0547 | 0.0457 | 0.0350 | 0.0451 | 0.057% | 99.496% |
| 37 | Chaetodipterus faber | 0.0646 | 0.0433 | 0.0176 | 0.0418 | 0.053% | 99.549% |
| 38 | Ictiobus bubalus | 0.0238 | 0.0265 | 0.0687 | 0.0397 | 0.050% | 99.599% |
| 39 | Pomatomus saltatrix | 0.0428 | 0.0409 | 0.0266 | 0.0368 | 0.047% | 99.646% |
| 40 | Peprilus burti | 0.0263 | 0.0409 | 0.0223 | 0.0298 | 0.038% | 99.684% |
| 41 | Menticirrhus littoralis | 0.0262 | 0.0289 | 0.0250 | 0.0267 | 0.034% | 99.718% |
| 42 | Micropogon undulatus | 0.0191 | 0.0264 | 0.0196 | 0.0217 | 0.028% | 99.746% |
| 43 | Stellifer lanceolatus | 0.0334 | 0.0144 | 0.0099 | 0.0192 | 0.024% | 99.770% |
| 44 | Sphyrna lewini | 0.0024 | 0.0024 | 0.0440 | 0.0163 | 0.021% | 99.791% |
| 45 | Atractosteus spatula | 0.0095 | 0.0072 | 0.0248 | 0.0138 | 0.018% | 99.808% |
| 46 | Rachycentron canadum | 0.0024 | 0.0388 | 0.0000 | 0.0137 | 0.017% | 99.826% |
| 47 | Libinia dubia | 0.0215 | 0.0073 | 0.0073 | 0.0120 | 0.015% | 99.841% |
| 48 | Achirus lineatus | 0.0072 | 0.0120 | 0.0126 | 0.0106 | 0.014% | 99.855% |
| 49 | Carcharhinus lumbatus | 0.0000 | 0.0097 | 0.0152 | 0.0083 | 0.011% | 99.865% |
| 50 | Polydactylus octonemus | 0.0142 | 0.0072 | 0.0024 | 0.0080 | 0.010% | 99.876% |
| 51 | Anchoa hepsetus | 0.0096 | 0.0024 | 0.0098 | 0.0073 | 0.009% | 99.885% |
| 52 | Lepisosteus oculatus | 0.0096 | 0.0000 | 0.0122 | 0.0073 | 0.009% | 99.894% |
| 53 | Trinectes maculatus | 0.0071 | 0.0024 | 0.0098 | 0.0065 | 0.008% | 99.902% |
| 54 | Symphurus plagiusa | 0.0119 | 0.0000 | 0.0051 | 0.0057 | 0.007% | 99.909% |
| 55 | Peprilus paru | 0.0000 | 0.0096 | 0.0074 | 0.0057 | 0.007% | 99.917% |
| 56 | Porichthys plectrodon | 0.0072 | 0.0072 | 0.0026 | 0.0057 | 0.007% | 99.924% |
| 57 | Etrumens teres | 0.0048 | 0.0000 | 0.0077 | 0.0042 | 0.005% | 99.929% |
| 58 | Porichthys porosissimus | 0.0000 | 0.0072 | 0.0050 | 0.0041 | 0.005% | 99.934% |
| 59 | Menippe adina | 0.0048 | 0.0000 | 0.0051 | 0.0033 | 0.004% | 99.938% |
| 60 | Trichiurus lepturus | 0.0024 | 0.0048 | 0.0026 | 0.0033 | 0.004% | 99.943% |
| 61 | Morone saxatilis | 0.0024 | 0.0024 | 0.0050 | 0.0032 | 0.004% | 99.947% |
| 62 | Prionotus tribulus | 0.0024 | 0.0049 | 0.0024 | 0.0032 | 0.004% | 99.951% |
| 63 | Trachinotus falcatus | 0.0024 | 0.0049 | 0.0024 | 0.0032 | 0.004% | 99.955% |
| 64 | Sardinella aurita | 0.0000 | 0.0024 | 0.0050 | 0.0025 | 0.003% | 99.958% |
| 65 | Lutjanus griseus | 0.0000 | 0.0024 | 0.0050 | 0.0025 | 0.003% | 99.961% |
| 66 | Lobotes surinamensis | 0.0000 | 0.0024 | 0.0048 | 0.0024 | 0.003% | 99.964% |
| 67 | Clupeidae | 0.0000 | 0.0000 | 0.0072 | 0.0024 | 0.003% | 99.967% |
| 68 | Echeneis naucrates | 0.0072 | 0.0000 | 0.0000 | 0.0024 | 0.003% | 99.970% |
| 69 | Strongylura marina | 0.0072 | 0.0000 | 0.0000 | 0.0024 | 0.003% | 99.973% |
| 70 | Prionotus | 0.0048 | 0.0024 | 0.0000 | 0.0024 | 0.003% | 99.977% |
| 71 | Synodus foetens | 0.0000 | 0.0024 | 0.0026 | 0.0017 | 0.002% | 99.979% |
| 72 | Anchoa mitchilli | 0.0000 | 0.0049 | 0.0000 | 0.0016 | 0.002% | 99.981% |
| 73 | Rhithropanopeus harrissii | 0.0048 | 0.0000 | 0.0000 | 0.0016 | 0.002% | 99.983% |
| 74 | Seriola rivoliana | 0.0024 | 0.0000 | 0.0024 | 0.0016 | 0.002% | 99.985% |
| 75 | Brevoortia gunteri | 0.0000 | 0.0000 | 0.0024 | 0.0008 | 0.001% | 99.986% |
| 76 | Syngnathus louisianae | 0.0000 | 0.0024 | 0.0000 | 0.0008 | 0.001% | 99.987% |
| 77 | Cynoscion xanphulus | 0.0024 | 0.0000 | 0.0000 | 0.0008 | 0.001% | 99.988% |
| 78 | Gobiesox strumosus | 0.0024 | 0.0000 | 0.0000 | 0.0008 | 0.001% | 99.989% |
| 79 | Gobioides broussonneti | 0.0024 | 0.0000 | 0.0000 | 0.0008 | 0.001% | 99.990% |
| 80 | Harengula jaguana | 0.0024 | 0.0000 | 0.0000 | 0.0008 | 0.001% | 99.991% |
| 81 | Libinia emerginata | 0.0024 | 0.0000 | 0.0000 | 0.0008 | 0.001% | 99.992% |
| 82 | Penaeus aztecus | 0.0000 | 0.0000 | 0.0024 | 0.0008 | 0.001% | 99.993% |
| 83 | Centropomus undecimalis | 0.0000 | 0.0024 | 0.0000 | 0.0008 | 0.001% | 99.994% |
| 84 | Chloroscombrus chrysurus | 0.0000 | 0.0024 | 0.0000 | 0.0008 | 0.001% | 99.995% |
| 85 | Megalops atlanticus | 0.0000 | 0.0024 | 0.0000 | 0.0008 | 0.001% | 99.996% |
| 86 | Neoelmis | 0.0000 | 0.0024 | 0.0000 | 0.0008 | 0.001% | 99.997% |
| 87 | Citharichthys spilopterus | 0.0024 | 0.0000 | 0.0000 | 0.0008 | 0.001% | 99.998% |
| 88 | Malaclemmys terrapin | 0.0024 | 0.0000 | 0.0000 | 0.0008 | 0.001% | 99.999% |
| 89 | Neoconger mucronatus | 0.0024 | 0.0000 | 0.0000 | 0.0008 | 0.001% | 100.000% |
|  | Total | 67.7288 | 77.5352 | 90.4236 | 78.5625 |  |  |

## Nekton in Trawl Samples

Average abundance of nekton caught in trawl samples (n/10 min) over all sampling periods.

| **Rank** | **Taxa/Species Name** | **Distance from Discharge** | | | **Mean** | **Pct** | **CumPct** |
| --- | --- | --- | --- | --- | --- | --- | --- |
|  |  | **B** | **C** | **R** |  |  |  |
| 1 | Anchoa mitchilli | 317.432 | 293.384 | 235.454 | 282.090 | 60.150% | 60.150% |
| 2 | Brevoortia patronus | 94.993 | 138.315 | 17.440 | 83.583 | 17.822% | 77.973% |
| 3 | Micropogonias undulatus | 61.246 | 44.491 | 42.150 | 49.296 | 10.511% | 88.484% |
| 4 | Leiostomus xanthurus | 18.724 | 16.136 | 3.591 | 12.817 | 2.733% | 91.217% |
| 5 | Penaeus aztecus | 11.962 | 11.324 | 7.862 | 10.383 | 2.214% | 93.431% |
| 6 | Penaeus setiferus | 8.308 | 11.740 | 7.010 | 9.019 | 1.923% | 95.354% |
| 7 | Micropogon undulatus | 11.489 | 9.291 | 3.518 | 8.099 | 1.727% | 97.081% |
| 8 | Callinectes sapidus | 2.937 | 2.600 | 1.603 | 2.380 | 0.507% | 97.589% |
| 9 | Palaemonetes | 1.889 | 1.872 | 0.534 | 1.432 | 0.305% | 97.894% |
| 10 | Anchoa hepsetus | 0.502 | 0.065 | 2.610 | 1.059 | 0.226% | 98.120% |
| 11 | Rangia cuneata | 2.833 | 0.000 | 0.000 | 0.944 | 0.201% | 98.321% |
| 12 | Lolliguncula brevis | 0.721 | 0.628 | 1.339 | 0.896 | 0.191% | 98.512% |
| 13 | Cynoscion arenAriopsis | 0.970 | 0.468 | 1.209 | 0.882 | 0.188% | 98.700% |
| 14 | Acetes americanus | 0.721 | 0.729 | 0.790 | 0.746 | 0.159% | 98.860% |
| 15 | Lagodon rhomboides | 0.745 | 0.867 | 0.200 | 0.604 | 0.129% | 98.988% |
| 16 | Penaeus | 0.589 | 0.682 | 0.537 | 0.603 | 0.129% | 99.117% |
| 17 | Bagre marinus | 0.552 | 0.486 | 0.630 | 0.556 | 0.119% | 99.235% |
| 18 | Sphoeroides parvus | 0.404 | 0.421 | 0.417 | 0.414 | 0.088% | 99.324% |
| 19 | Ariopsis felis | 0.388 | 0.272 | 0.486 | 0.382 | 0.081% | 99.405% |
| 20 | Ictalurus furcatus | 0.311 | 0.167 | 0.621 | 0.366 | 0.078% | 99.483% |
| 21 | Bairdiella chrysoura | 0.255 | 0.219 | 0.482 | 0.319 | 0.068% | 99.551% |
| 22 | Citharichthys spilopterus | 0.436 | 0.240 | 0.248 | 0.308 | 0.066% | 99.617% |
| 23 | Callinectes | 0.226 | 0.168 | 0.055 | 0.150 | 0.032% | 99.649% |
| 24 | Symphurus plagiusa | 0.160 | 0.096 | 0.100 | 0.118 | 0.025% | 99.674% |
| 25 | Prionotus tribulus | 0.136 | 0.126 | 0.037 | 0.099 | 0.021% | 99.695% |
| 26 | Macrobranchium ohione | 0.107 | 0.084 | 0.067 | 0.086 | 0.018% | 99.714% |
| 27 | Mugil cephalus | 0.115 | 0.044 | 0.067 | 0.076 | 0.016% | 99.730% |
| 28 | Dorosoma petenense | 0.101 | 0.074 | 0.034 | 0.070 | 0.015% | 99.745% |
| 29 | Chloroscombrus chrysurus | 0.041 | 0.014 | 0.149 | 0.068 | 0.015% | 99.759% |
| 30 | Gobiosoma bosci | 0.034 | 0.054 | 0.104 | 0.064 | 0.014% | 99.773% |
| 31 | Paralichthys lethostigma | 0.085 | 0.049 | 0.053 | 0.062 | 0.013% | 99.786% |
| 32 | Tagelus divisus | 0.181 | 0.000 | 0.000 | 0.060 | 0.013% | 99.799% |
| 33 | Synodus foetens | 0.053 | 0.049 | 0.059 | 0.054 | 0.011% | 99.810% |
| 34 | Alosa chrysochloris | 0.070 | 0.049 | 0.026 | 0.048 | 0.010% | 99.821% |
| 35 | Achirus lineatus | 0.055 | 0.042 | 0.047 | 0.048 | 0.010% | 99.831% |
| 36 | Gobionellus boleosoma | 0.069 | 0.021 | 0.041 | 0.043 | 0.009% | 99.840% |
| 37 | Sphoeroides | 0.049 | 0.063 | 0.015 | 0.042 | 0.009% | 99.849% |
| 38 | Loandalia americana | 0.124 | 0.000 | 0.000 | 0.041 | 0.009% | 99.858% |
| 39 | Trinectes maculatus | 0.046 | 0.044 | 0.029 | 0.040 | 0.008% | 99.866% |
| 40 | Rhithropanopeus harrissii | 0.046 | 0.058 | 0.000 | 0.035 | 0.007% | 99.874% |
| 41 | Peprilus burti | 0.034 | 0.032 | 0.023 | 0.030 | 0.006% | 99.880% |
| 42 | Caranx hippos | 0.050 | 0.012 | 0.017 | 0.026 | 0.006% | 99.886% |
| 43 | Palaemonetes vulgaris | 0.051 | 0.007 | 0.020 | 0.026 | 0.006% | 99.891% |
| 44 | Menippe mercenaria | 0.027 | 0.028 | 0.022 | 0.026 | 0.005% | 99.897% |
| 45 | Palaemonetes pugio | 0.054 | 0.019 | 0.000 | 0.024 | 0.005% | 99.902% |
| 46 | Cynoscion nebulosus | 0.019 | 0.012 | 0.041 | 0.024 | 0.005% | 99.907% |
| 47 | Opisthonema oglinum | 0.028 | 0.014 | 0.028 | 0.023 | 0.005% | 99.912% |
| 48 | Cyprinodon variegatus | 0.018 | 0.033 | 0.007 | 0.019 | 0.004% | 99.916% |
| 49 | Menidia beryllina | 0.021 | 0.021 | 0.010 | 0.017 | 0.004% | 99.920% |
| 50 | Prionotus | 0.021 | 0.014 | 0.016 | 0.017 | 0.004% | 99.923% |
| 51 | Gammaridae | 0.050 | 0.000 | 0.000 | 0.017 | 0.004% | 99.927% |
| 52 | Larimus faciatus | 0.009 | 0.000 | 0.039 | 0.016 | 0.003% | 99.930% |
| 53 | Dorosoma cepedianum | 0.007 | 0.026 | 0.014 | 0.015 | 0.003% | 99.933% |
| 54 | Penaeus duorarum | 0.012 | 0.028 | 0.007 | 0.015 | 0.003% | 99.937% |
| 55 | Microgobius gulosus | 0.002 | 0.000 | 0.040 | 0.014 | 0.003% | 99.940% |
| 56 | Eucinostomus argenteus | 0.012 | 0.012 | 0.017 | 0.013 | 0.003% | 99.943% |
| 57 | Porichthys plectrodon | 0.021 | 0.000 | 0.019 | 0.013 | 0.003% | 99.945% |
| 58 | Leptocephalus larvae | 0.009 | 0.016 | 0.014 | 0.013 | 0.003% | 99.948% |
| 59 | Trichiurus lepturus | 0.016 | 0.002 | 0.021 | 0.013 | 0.003% | 99.951% |
| 60 | Archosargus probatocephalus | 0.009 | 0.014 | 0.012 | 0.012 | 0.002% | 99.954% |
| 61 | Chaetodipterus faber | 0.014 | 0.016 | 0.005 | 0.012 | 0.002% | 99.956% |
| 62 | Menippe adina | 0.011 | 0.005 | 0.012 | 0.009 | 0.002% | 99.958% |
| 63 | Gobiesox strumosus | 0.009 | 0.012 | 0.007 | 0.009 | 0.002% | 99.960% |
| 64 | Xanthidae | 0.019 | 0.002 | 0.005 | 0.009 | 0.002% | 99.962% |
| 65 | Goniadidae | 0.025 | 0.000 | 0.000 | 0.008 | 0.002% | 99.964% |
| 66 | Myrophis punctatus | 0.005 | 0.005 | 0.015 | 0.008 | 0.002% | 99.965% |
| 67 | Dasyatis sabina | 0.007 | 0.009 | 0.007 | 0.008 | 0.002% | 99.967% |
| 68 | Peprilus alepidotus | 0.000 | 0.014 | 0.009 | 0.008 | 0.002% | 99.969% |
| 69 | Dyspanopeus texanus | 0.005 | 0.019 | 0.000 | 0.008 | 0.002% | 99.970% |
| 70 | Polydactylus octonemus | 0.000 | 0.005 | 0.016 | 0.007 | 0.002% | 99.972% |
| 71 | Pogonias cromis | 0.007 | 0.012 | 0.002 | 0.007 | 0.002% | 99.973% |
| 72 | Loligo | 0.000 | 0.002 | 0.014 | 0.006 | 0.001% | 99.975% |
| 73 | Anchoa lyolepis | 0.000 | 0.000 | 0.016 | 0.005 | 0.001% | 99.976% |
| 74 | Hemicaranx amblyrhynchus | 0.002 | 0.009 | 0.002 | 0.005 | 0.001% | 99.977% |
| 75 | Synodus | 0.005 | 0.009 | 0.000 | 0.005 | 0.001% | 99.978% |
| 76 | Lutjanus griseus | 0.009 | 0.000 | 0.005 | 0.005 | 0.001% | 99.979% |
| 77 | Ophichthus gomesi | 0.007 | 0.007 | 0.000 | 0.005 | 0.001% | 99.980% |
| 78 | Sphoeroides nephelus | 0.014 | 0.000 | 0.000 | 0.005 | 0.001% | 99.981% |
| 79 | Paralichthys | 0.002 | 0.007 | 0.002 | 0.004 | 0.001% | 99.981% |
| 80 | Prionotus roseus | 0.002 | 0.005 | 0.005 | 0.004 | 0.001% | 99.982% |
| 81 | Sygnathus | 0.007 | 0.005 | 0.000 | 0.004 | 0.001% | 99.983% |
| 82 | Syngnathus scovelli | 0.000 | 0.012 | 0.000 | 0.004 | 0.001% | 99.984% |
| 83 | Syngnathus louisianae | 0.007 | 0.002 | 0.002 | 0.004 | 0.001% | 99.985% |
| 84 | Poecilia latipinna | 0.000 | 0.000 | 0.010 | 0.003 | 0.001% | 99.985% |
| 85 | Gobionellus oceanicus | 0.002 | 0.005 | 0.002 | 0.003 | 0.001% | 99.986% |
| 86 | Harengula pensacolae | 0.002 | 0.007 | 0.000 | 0.003 | 0.001% | 99.987% |
| 87 | Tozeuma carolinense | 0.007 | 0.000 | 0.002 | 0.003 | 0.001% | 99.987% |
| 88 | Opsanus beta | 0.005 | 0.000 | 0.005 | 0.003 | 0.001% | 99.988% |
| 89 | Elops saurus | 0.007 | 0.002 | 0.000 | 0.003 | 0.001% | 99.989% |
| 90 | Capitellidae | 0.009 | 0.000 | 0.000 | 0.003 | 0.001% | 99.989% |
| 91 | Cumacea | 0.009 | 0.000 | 0.000 | 0.003 | 0.001% | 99.990% |
| 92 | Ensis minor | 0.009 | 0.000 | 0.000 | 0.003 | 0.001% | 99.991% |
| 93 | Alpheus heterochaelis | 0.002 | 0.002 | 0.002 | 0.002 | 0.001% | 99.991% |
| 94 | Selene vomer | 0.002 | 0.000 | 0.005 | 0.002 | 0.000% | 99.992% |
| 95 | Microphis brachyurus | 0.000 | 0.007 | 0.000 | 0.002 | 0.000% | 99.992% |
| 96 | Lepophidium graellsi | 0.000 | 0.000 | 0.007 | 0.002 | 0.000% | 99.993% |
| 97 | Macrobrachium | 0.002 | 0.005 | 0.000 | 0.002 | 0.000% | 99.993% |
| 98 | Urophycis floridanus | 0.000 | 0.002 | 0.002 | 0.002 | 0.000% | 99.994% |
| 99 | Chasmodes saburrae | 0.000 | 0.005 | 0.000 | 0.002 | 0.000% | 99.994% |
| 100 | Centropomus undecimalis | 0.002 | 0.000 | 0.002 | 0.002 | 0.000% | 99.994% |
| 101 | Menticirrhus americanus | 0.000 | 0.005 | 0.000 | 0.002 | 0.000% | 99.995% |
| 102 | Hippolyte pleuracanthus | 0.002 | 0.002 | 0.000 | 0.002 | 0.000% | 99.995% |
| 103 | Peprilus paru | 0.002 | 0.000 | 0.002 | 0.002 | 0.000% | 99.995% |
| 104 | Squilla empusa | 0.000 | 0.000 | 0.005 | 0.002 | 0.000% | 99.996% |
| 105 | Libinia dubia | 0.002 | 0.000 | 0.002 | 0.002 | 0.000% | 99.996% |
| 106 | Microgobius thalassinus | 0.002 | 0.002 | 0.000 | 0.002 | 0.000% | 99.996% |
| 107 | Gerres cinereus | 0.000 | 0.000 | 0.002 | 0.001 | 0.000% | 99.996% |
| 108 | Lutjanus jocu | 0.000 | 0.000 | 0.002 | 0.001 | 0.000% | 99.997% |
| 109 | Fundulus grandis | 0.000 | 0.002 | 0.000 | 0.001 | 0.000% | 99.997% |
| 110 | Lutjanus apodus | 0.000 | 0.002 | 0.000 | 0.001 | 0.000% | 99.997% |
| 111 | Neopanope | 0.000 | 0.002 | 0.000 | 0.001 | 0.000% | 99.997% |
| 112 | Gobiosoma robustum | 0.002 | 0.000 | 0.000 | 0.001 | 0.000% | 99.997% |
| 113 | Menidia peninsulae | 0.002 | 0.000 | 0.000 | 0.001 | 0.000% | 99.997% |
| 114 | Neoconger mucronatus | 0.002 | 0.000 | 0.000 | 0.001 | 0.000% | 99.998% |
| 115 | Ophidioin | 0.000 | 0.002 | 0.000 | 0.001 | 0.000% | 99.998% |
| 116 | Ameiurus natalis | 0.000 | 0.000 | 0.002 | 0.001 | 0.000% | 99.998% |
| 117 | Callinectes similis | 0.000 | 0.002 | 0.000 | 0.001 | 0.000% | 99.998% |
| 118 | Gambusia affinis | 0.000 | 0.000 | 0.002 | 0.001 | 0.000% | 99.998% |
| 119 | Gobioides broussonneti | 0.002 | 0.000 | 0.000 | 0.001 | 0.000% | 99.998% |
| 120 | Lepophidum brevibarbe | 0.000 | 0.000 | 0.002 | 0.001 | 0.000% | 99.999% |
| 121 | Porichthys poroissimus | 0.000 | 0.000 | 0.002 | 0.001 | 0.000% | 99.999% |
| 122 | Scomberomorus maculatus | 0.000 | 0.000 | 0.002 | 0.001 | 0.000% | 99.999% |
| 123 | Uca | 0.000 | 0.000 | 0.002 | 0.001 | 0.000% | 99.999% |
| 124 | Eucinostomus | 0.000 | 0.002 | 0.000 | 0.001 | 0.000% | 99.999% |
| 125 | Oligoplites saurus | 0.000 | 0.002 | 0.000 | 0.001 | 0.000% | 99.999% |
| 126 | Symphurus | 0.000 | 0.002 | 0.000 | 0.001 | 0.000% | 100.000% |
| 127 | Trachinotus falcatus | 0.002 | 0.000 | 0.000 | 0.001 | 0.000% | 100.000% |
| 128 | Chilomycterus schoepfi | 0.002 | 0.000 | 0.000 | 0.001 | 0.000% | 100.000% |
| 129 | Rachycentron canadum | 0.002 | 0.000 | 0.000 | 0.001 | 0.000% | 100.000% |
|  | Total | **540.814** | **535.937** | **330.172** | **468.974** |  |  |

## Phytoplankton

Phytoplankton abundance (cells/m^3^) averaged over all sampling periods.

| **Rank** | **Taxa/Species Name** | **Distance From Discharge** | | **Mean** | **Pct** | **CumPct** |
| --- | --- | --- | --- | --- | --- | --- |
|  |  | **B** | **R** |  |  |  |
| 1 | Synechocystis | 275089.5780523 | 194794.2213307 | 234941.8996915 | 62.258% | 62.258% |
| 2 | Cyanobacteria | 154846.2429887 | 75940.4416378 | 115393.3423132 | 30.578% | 92.836% |
| 3 | Synechococcus | 10441.0218718 | 12671.8852421 | 11556.4535570 | 3.062% | 95.899% |
| 4 | Cell | 2313.2540333 | 3715.8830084 | 3014.5685209 | 0.799% | 96.698% |
| 5 | Chroococcus | 1632.6049718 | 4131.0952559 | 2881.8501138 | 0.764% | 97.461% |
| 6 | Cryptomonas | 2319.0556819 | 1948.7593950 | 2133.9075385 | 0.565% | 98.027% |
| 7 | No Flagella | 1545.7185714 | 2059.0991288 | 1802.4088501 | 0.478% | 98.504% |
| 8 | Flagellate Unid | 1359.2958399 | 2060.4160990 | 1709.8559695 | 0.453% | 98.957% |
| 9 | Nannochloris | 732.7182378 | 131.5771793 | 432.1477085 | 0.115% | 99.072% |
| 10 | Carteria | 46.3888983 | 704.3229044 | 375.3559013 | 0.099% | 99.171% |
| 11 | Limnothrix | 289.8951429 | 388.1209695 | 339.0080562 | 0.090% | 99.261% |
| 12 | Fistulifera | 288.6000000 | 341.1144481 | 314.8572240 | 0.083% | 99.345% |
| 13 | Pennate Diatom | 467.3510160 | 156.4617384 | 311.9063772 | 0.083% | 99.427% |
| 14 | Cyclotella | 145.1655568 | 466.0347753 | 305.6001661 | 0.081% | 99.508% |
| 15 | Pseudoanabaena | 64.2717143 | 394.7239854 | 229.4978499 | 0.061% | 99.569% |
| 16 | Spores | 125.3071429 | 265.7182089 | 195.5126759 | 0.052% | 99.621% |
| 17 | Kirchneriella | 180.6365627 | 88.0676123 | 134.3520875 | 0.036% | 99.657% |
| 18 | Stephanodiscus | 24.6678078 | 236.9219258 | 130.7948668 | 0.035% | 99.691% |
| 19 | Pseudodidymocystis planctonica | 9.6363571 | 153.0799749 | 81.3581660 | 0.022% | 99.713% |
| 20 | Chlorella | 56.0713556 | 105.1130266 | 80.5921911 | 0.021% | 99.734% |
| 21 | Snowella | 0.0000000 | 160.5586630 | 80.2793315 | 0.021% | 99.755% |
| 22 | Centric Diatom | 90.0443475 | 60.1743291 | 75.1093383 | 0.020% | 99.775% |
| 23 | Ochromonas | 43.8651959 | 64.4570732 | 54.1611346 | 0.014% | 99.790% |
| 24 | Chlamydomonas | 40.2296793 | 52.5749587 | 46.4023190 | 0.012% | 99.802% |
| 25 | Algae Unid | 0.1214286 | 87.5625000 | 43.8419643 | 0.012% | 99.814% |
| 26 | Navicula | 66.6756127 | 20.4848331 | 43.5802229 | 0.012% | 99.825% |
| 27 | Tetraselmis | 55.5710672 | 27.7822511 | 41.6766592 | 0.011% | 99.836% |
| 28 | Glaucospira | 47.6882857 | 26.3111147 | 36.9997002 | 0.010% | 99.846% |
| 29 | Chroomonas | 10.7358791 | 57.8005624 | 34.2682207 | 0.009% | 99.855% |
| 30 | Planktothrix | 47.5860000 | 20.8728403 | 34.2294202 | 0.009% | 99.864% |
| 31 | Chrysochromulina | 3.8845714 | 59.2845974 | 31.5845844 | 0.008% | 99.872% |
| 32 | Chlorophyta | 37.2894354 | 22.7664714 | 30.0279534 | 0.008% | 99.880% |
| 33 | Nitzschia | 20.1195546 | 34.8374276 | 27.4784911 | 0.007% | 99.888% |
| 34 | Gymnodinium estuariale | 20.5888571 | 27.2528423 | 23.9208497 | 0.006% | 99.894% |
| 35 | Glenodinium | 37.7473141 | 8.1342759 | 22.9407950 | 0.006% | 99.900% |
| 36 | Peridinium | 44.7498435 | 0.4675776 | 22.6087106 | 0.006% | 99.906% |
| 37 | Dinoflagellata | 25.6485097 | 15.7668817 | 20.7076957 | 0.005% | 99.912% |
| 38 | Gymnodinium | 19.8585304 | 19.9675546 | 19.9130425 | 0.005% | 99.917% |
| 39 | Komma | 20.4800000 | 16.2147186 | 18.3473593 | 0.005% | 99.922% |
| 40 | Apedinella spinifera | 13.9319286 | 14.7591958 | 14.3455622 | 0.004% | 99.926% |
| 41 | Chaetoceros minimus | 11.1755000 | 16.8331109 | 14.0043055 | 0.004% | 99.929% |
| 42 | Planktolyngbya | 19.0642857 | 6.0433171 | 12.5538014 | 0.003% | 99.933% |
| 43 | Cryptomonadaceae | 8.4142857 | 16.0538826 | 12.2340841 | 0.003% | 99.936% |
| 44 | Romeria | 9.5142857 | 13.6233766 | 11.5688312 | 0.003% | 99.939% |
| 45 | Cylindrotheca closterium | 6.9069545 | 14.6302298 | 10.7685922 | 0.003% | 99.942% |
| 46 | Chaetoceros simplex | 20.9603893 | 0.2963715 | 10.6283804 | 0.003% | 99.945% |
| 47 | Chaetoceros tenuissimus | 12.2557857 | 8.8459337 | 10.5508597 | 0.003% | 99.947% |
| 48 | Chlamydomonas 2 | 10.0946882 | 9.5019056 | 9.7982969 | 0.003% | 99.950% |
| 49 | Euglena | 7.5510292 | 10.9754508 | 9.2632400 | 0.002% | 99.952% |
| 50 | Selenastrum | 7.4386046 | 9.5565680 | 8.4975863 | 0.002% | 99.955% |
| 51 | Ankistrodesmus | 6.0379297 | 9.7978502 | 7.9178900 | 0.002% | 99.957% |
| 52 | Chrysochromulina parva | 1.1165714 | 14.4162277 | 7.7663996 | 0.002% | 99.959% |
| 53 | Chlorococcum | 6.4086625 | 8.5790588 | 7.4938606 | 0.002% | 99.961% |
| 54 | Monoraphidium | 7.1165714 | 5.6844156 | 6.4004935 | 0.002% | 99.962% |
| 55 | Nitzschia acicularis | 11.3335563 | 1.4165177 | 6.3750370 | 0.002% | 99.964% |
| 56 | Scenedesmus | 5.6103440 | 6.2326694 | 5.9215067 | 0.002% | 99.966% |
| 57 | Nitzschia 2 | 4.9389195 | 6.4625190 | 5.7007192 | 0.002% | 99.967% |
| 58 | Chaetoceros subtilis | 6.3428571 | 4.6815341 | 5.5121956 | 0.001% | 99.969% |
| 59 | Oscillatoria | 3.7901241 | 6.7433542 | 5.2667392 | 0.001% | 99.970% |
| 60 | Pyraminonas | 1.6605714 | 8.4967742 | 5.0786728 | 0.001% | 99.971% |
| 61 | Achnanthes | 4.5606633 | 4.5217585 | 4.5412109 | 0.001% | 99.973% |
| 62 | Chaetoceros | 1.9636814 | 5.4716181 | 3.7176498 | 0.001% | 99.974% |
| 63 | Protoperidinium | 7.0530895 | 0.2943055 | 3.6736975 | 0.001% | 99.975% |
| 64 | Merismopedia tenuissima | 1.7158070 | 5.2349188 | 3.4753629 | 0.001% | 99.976% |
| 65 | Raphidiopsis | 6.4986372 | 0.1802676 | 3.3394524 | 0.001% | 99.976% |
| 66 | Characium | 2.5938633 | 3.7866640 | 3.1902636 | 0.001% | 99.977% |
| 67 | Keratococcus | 4.5320000 | 1.7749107 | 3.1534554 | 0.001% | 99.978% |
| 68 | Eutreptia | 4.3620333 | 1.5508647 | 2.9564490 | 0.001% | 99.979% |
| 69 | Merismopedia | 2.0380737 | 3.8007886 | 2.9194311 | 0.001% | 99.980% |
| 70 | Navicula 2 | 3.3728229 | 2.3544142 | 2.8636185 | 0.001% | 99.980% |
| 71 | Skeletonema costatum | 1.0706561 | 4.0682611 | 2.5694586 | 0.001% | 99.981% |
| 72 | Navicula minutula | 1.4373954 | 3.4417480 | 2.4395717 | 0.001% | 99.982% |
| 73 | Micractinium belenophorum | 0.0000000 | 4.7901786 | 2.3950893 | 0.001% | 99.982% |
| 74 | Schizothrix | 1.3714286 | 3.2548160 | 2.3131223 | 0.001% | 99.983% |
| 75 | Dinophyceae | 0.0000000 | 4.5335498 | 2.2667749 | 0.001% | 99.984% |
| 76 | Gyrodinium spirale | 2.2598156 | 2.2003080 | 2.2300618 | 0.001% | 99.984% |
| 77 | Nitzschia longissima | 1.9153527 | 2.3019573 | 2.1086550 | 0.001% | 99.985% |
| 78 | Cocconeis | 2.2713455 | 1.7428016 | 2.0070735 | 0.001% | 99.985% |
| 79 | Aphanocapsa | 0.1067692 | 3.3453935 | 1.7260814 | 0.000% | 99.986% |
| 80 | Amphora | 2.3589526 | 0.8833259 | 1.6211393 | 0.000% | 99.986% |
| 81 | Gymnodinium sanguineum | 1.7753237 | 1.2618207 | 1.5185722 | 0.000% | 99.987% |
| 82 | Sphaerocystis | 1.4460757 | 1.1966541 | 1.3213649 | 0.000% | 99.987% |
| 83 | Oocystis | 0.0000000 | 2.4514217 | 1.2257108 | 0.000% | 99.987% |
| 84 | Skeletonema | 1.3901694 | 0.9990781 | 1.1946237 | 0.000% | 99.988% |
| 85 | Leptocylindrus minimus | 0.6948859 | 1.6819499 | 1.1884179 | 0.000% | 99.988% |
| 86 | Diploneis | 1.3041114 | 1.0266291 | 1.1653702 | 0.000% | 99.988% |
| 87 | Nitzschia palacea | 1.2021467 | 1.1113431 | 1.1567449 | 0.000% | 99.988% |
| 88 | Monoraphidium contortum | 1.5857143 | 0.6682224 | 1.1269683 | 0.000% | 99.989% |
| 89 | Rhizosolenia | 0.5860247 | 1.6624736 | 1.1242491 | 0.000% | 99.989% |
| 90 | Cyclotella meneghiniana | 0.9714286 | 1.0950081 | 1.0332183 | 0.000% | 99.989% |
| 91 | Nitzschia closterium | 0.9626942 | 1.0280153 | 0.9953548 | 0.000% | 99.990% |
| 92 | Cymbella | 0.9109758 | 1.0791996 | 0.9950877 | 0.000% | 99.990% |
| 93 | Coscinodiscus | 0.6283682 | 1.3409294 | 0.9846488 | 0.000% | 99.990% |
| 94 | Actinastrum | 0.5353706 | 1.4278980 | 0.9816343 | 0.000% | 99.990% |
| 95 | Haptophyte | 1.9514286 | 0.0000000 | 0.9757143 | 0.000% | 99.991% |
| 96 | Scenedesmus quadricauda | 0.5078720 | 1.4073145 | 0.9575932 | 0.000% | 99.991% |
| 97 | Nephroselmis | 1.3694745 | 0.4851174 | 0.9272959 | 0.000% | 99.991% |
| 98 | Tetraedron | 0.8181503 | 0.9819452 | 0.9000478 | 0.000% | 99.991% |
| 99 | Desmodesmus communis | 1.5857143 | 0.2102273 | 0.8979708 | 0.000% | 99.992% |
| 100 | Dinoflagellates | 1.3571429 | 0.4326299 | 0.8948864 | 0.000% | 99.992% |
| 101 | Euglenophyta | 1.4818179 | 0.3022863 | 0.8920521 | 0.000% | 99.992% |
| 102 | Marsionella | 1.1614634 | 0.6120303 | 0.8867469 | 0.000% | 99.992% |
| 103 | Pinnularia | 0.8460018 | 0.8748889 | 0.8604454 | 0.000% | 99.993% |
| 104 | Asterionella | 1.1008640 | 0.4993085 | 0.8000863 | 0.000% | 99.993% |
| 105 | Diatoma | 0.0571429 | 1.4909399 | 0.7740414 | 0.000% | 99.993% |
| 106 | Thalassionema | 0.9405129 | 0.5941628 | 0.7673379 | 0.000% | 99.993% |
| 107 | Coccolithophores | 1.2857143 | 0.2430682 | 0.7643912 | 0.000% | 99.993% |
| 108 | Stichococcus | 0.0285714 | 1.4714011 | 0.7499863 | 0.000% | 99.994% |
| 109 | Prymnesium | 0.7928571 | 0.7057630 | 0.7493101 | 0.000% | 99.994% |
| 110 | Gyrosigma | 0.7086608 | 0.6863717 | 0.6975162 | 0.000% | 99.994% |
| 111 | Tetraedron muticum | 0.7928571 | 0.5905709 | 0.6917140 | 0.000% | 99.994% |
| 112 | Campylodiscus | 0.0041321 | 1.3655939 | 0.6848630 | 0.000% | 99.994% |
| 113 | Tetrastrum | 0.4391593 | 0.8823813 | 0.6607703 | 0.000% | 99.995% |
| 114 | Melosira | 0.3997633 | 0.8808897 | 0.6403265 | 0.000% | 99.995% |
| 115 | Rhizosolenia setigera | 0.6397295 | 0.6222699 | 0.6309997 | 0.000% | 99.995% |
| 116 | Synedra | 0.8536623 | 0.3510997 | 0.6023810 | 0.000% | 99.995% |
| 117 | Anabaena | 0.4036478 | 0.7829796 | 0.5933137 | 0.000% | 99.995% |
| 118 | Spirulina | 0.9285714 | 0.2058149 | 0.5671931 | 0.000% | 99.995% |
| 119 | Cylindrotheca | 0.4136661 | 0.6013350 | 0.5075006 | 0.000% | 99.995% |
| 120 | Gonyaulax | 0.3010670 | 0.6922176 | 0.4966423 | 0.000% | 99.996% |
| 121 | Asterionellopsis glacialis | 0.0951916 | 0.8517117 | 0.4734516 | 0.000% | 99.996% |
| 122 | Bacillaria | 0.5217373 | 0.3778443 | 0.4497908 | 0.000% | 99.996% |
| 123 | Isochrysis | 0.6898389 | 0.1994152 | 0.4446270 | 0.000% | 99.996% |
| 124 | Crucigenia tetrapedia | 0.7928571 | 0.0000000 | 0.3964286 | 0.000% | 99.996% |
| 125 | Ankistrodesmus falcatus | 0.3785714 | 0.3984037 | 0.3884876 | 0.000% | 99.996% |
| 126 | Kirchneriella obesa | 0.2285714 | 0.5455222 | 0.3870468 | 0.000% | 99.996% |
| 127 | Scenedesmus bijuga | 0.1285714 | 0.6217456 | 0.3751585 | 0.000% | 99.996% |
| 128 | Schroederia setigera | 0.0642857 | 0.6826299 | 0.3734578 | 0.000% | 99.996% |
| 129 | Thalassiosira | 0.1571429 | 0.5883010 | 0.3727219 | 0.000% | 99.997% |
| 130 | Prorocentrum | 0.0037143 | 0.7195020 | 0.3616081 | 0.000% | 99.997% |
| 131 | Ditylum | 0.3913757 | 0.2838085 | 0.3375921 | 0.000% | 99.997% |
| 132 | Elakatothrix | 0.2041661 | 0.4699818 | 0.3370739 | 0.000% | 99.997% |
| 133 | Aphanothece | 0.0000000 | 0.6306818 | 0.3153409 | 0.000% | 99.997% |
| 134 | Asterionellopsis | 0.2437001 | 0.3797387 | 0.3117194 | 0.000% | 99.997% |
| 135 | Asterococcus | 0.0000000 | 0.6160112 | 0.3080056 | 0.000% | 99.997% |
| 136 | Nitzschia palea | 0.4214286 | 0.1885552 | 0.3049919 | 0.000% | 99.997% |
| 137 | Rhizosolenia fragillissima | 0.4642857 | 0.1404221 | 0.3023539 | 0.000% | 99.997% |
| 138 | Pseudonitzschia | 0.0590332 | 0.5366911 | 0.2978622 | 0.000% | 99.997% |
| 139 | Cosmarium | 0.0571429 | 0.5367364 | 0.2969396 | 0.000% | 99.997% |
| 140 | Bacillariophyceae | 0.3285714 | 0.2581487 | 0.2933600 | 0.000% | 99.997% |
| 141 | Amphiprora | 0.3512100 | 0.2128142 | 0.2820121 | 0.000% | 99.998% |
| 142 | Bacillaria paradoxa | 0.2285714 | 0.3299513 | 0.2792614 | 0.000% | 99.998% |
| 143 | Oscillatoria 2 | 0.0000000 | 0.5547325 | 0.2773663 | 0.000% | 99.998% |
| 144 | Surirella | 0.2821614 | 0.2709482 | 0.2765548 | 0.000% | 99.998% |
| 145 | Guinardia | 0.0042857 | 0.5363781 | 0.2703319 | 0.000% | 99.998% |
| 146 | Aulacosira | 0.0000000 | 0.5285013 | 0.2642506 | 0.000% | 99.998% |
| 147 | Asterionella japonica | 0.5071429 | 0.0000000 | 0.2535714 | 0.000% | 99.998% |
| 148 | Microcytis | 0.3571429 | 0.1473365 | 0.2522397 | 0.000% | 99.998% |
| 149 | Euglena acusformis | 0.2500000 | 0.2139340 | 0.2319670 | 0.000% | 99.998% |
| 150 | Hemiaulus | 0.2142857 | 0.2336111 | 0.2239484 | 0.000% | 99.998% |
| 151 | Microspora | 0.3115601 | 0.1228953 | 0.2172277 | 0.000% | 99.998% |
| 152 | Fragilaria | 0.1089591 | 0.3201798 | 0.2145694 | 0.000% | 99.998% |
| 153 | Thoracomonas | 0.2428571 | 0.1862148 | 0.2145360 | 0.000% | 99.998% |
| 154 | Diatom Unid | 0.0568488 | 0.3653405 | 0.2110947 | 0.000% | 99.998% |
| 155 | Cymatopleura | 0.4208161 | 0.0000000 | 0.2104080 | 0.000% | 99.998% |
| 156 | Oxytoxum | 0.1739208 | 0.2170681 | 0.1954945 | 0.000% | 99.999% |
| 157 | Dictyospacium | 0.3657143 | 0.0189394 | 0.1923268 | 0.000% | 99.999% |
| 158 | Merismopedia convoluta | 0.1928571 | 0.1747835 | 0.1838203 | 0.000% | 99.999% |
| 159 | Merismpoedia glauca | 0.2285714 | 0.1361607 | 0.1823661 | 0.000% | 99.999% |
| 160 | Pandorina | 0.0805931 | 0.2591771 | 0.1698851 | 0.000% | 99.999% |
| 161 | Chaetoceros didymum | 0.0000516 | 0.3380787 | 0.1690651 | 0.000% | 99.999% |
| 162 | Protoperidinium minimum | 0.3237143 | 0.0008608 | 0.1622875 | 0.000% | 99.999% |
| 163 | Navicula 3 | 0.2394165 | 0.0742852 | 0.1568509 | 0.000% | 99.999% |
| 164 | Eremosphaera | 0.1857143 | 0.1245942 | 0.1551542 | 0.000% | 99.999% |
| 165 | Scenedesmus armatus | 0.1214286 | 0.1734984 | 0.1474635 | 0.000% | 99.999% |
| 166 | Thalassiosiraceae | 0.1357143 | 0.1544913 | 0.1451028 | 0.000% | 99.999% |
| 167 | Phacus | 0.1192336 | 0.1527875 | 0.1360106 | 0.000% | 99.999% |
| 168 | Oxyphysis oxytoxoides | 0.1094224 | 0.1617880 | 0.1356052 | 0.000% | 99.999% |
| 169 | Scenedesmus dimorphus | 0.2129906 | 0.0549919 | 0.1339912 | 0.000% | 99.999% |
| 170 | Chlorogonium | 0.0000000 | 0.2677538 | 0.1338769 | 0.000% | 99.999% |
| 171 | Pleurosigma | 0.1271489 | 0.1367886 | 0.1319688 | 0.000% | 99.999% |
| 172 | Diogenes | 0.1557801 | 0.1079917 | 0.1318859 | 0.000% | 99.999% |
| 173 | Closterium | 0.1357143 | 0.0865124 | 0.1111134 | 0.000% | 99.999% |
| 174 | Furcilla | 0.0000000 | 0.2200067 | 0.1100033 | 0.000% | 99.999% |
| 175 | Gleocystis | 0.0662510 | 0.1528930 | 0.1095720 | 0.000% | 99.999% |
| 176 | Striatella | 0.1500000 | 0.0623647 | 0.1061824 | 0.000% | 99.999% |
| 177 | Acutodesmus dimorphus | 0.0000000 | 0.2102273 | 0.1051136 | 0.000% | 99.999% |
| 178 | Coscinodiscaceae | 0.1928571 | 0.0104843 | 0.1016707 | 0.000% | 99.999% |
| 179 | Chrysophyta | 0.0000000 | 0.2026515 | 0.1013258 | 0.000% | 99.999% |
| 180 | Brachymonas | 0.0000000 | 0.1864534 | 0.0932267 | 0.000% | 99.999% |
| 181 | Dinophysis | 0.1709499 | 0.0000000 | 0.0854749 | 0.000% | 99.999% |
| 182 | Tropedoneis | 0.0212321 | 0.1342718 | 0.0777519 | 0.000% | 99.999% |
| 183 | Schroederia | 0.0071429 | 0.1450570 | 0.0760999 | 0.000% | 99.999% |
| 184 | Coelosphaerium | 0.0000000 | 0.1484295 | 0.0742147 | 0.000% | 99.999% |
| 185 | Epipyxis | 0.0000000 | 0.1480374 | 0.0740187 | 0.000% | 99.999% |
| 186 | Actinoptychus senAriopsis | 0.0844093 | 0.0570309 | 0.0707201 | 0.000% | 99.999% |
| 187 | Spermatazoa | 0.0928571 | 0.0467397 | 0.0697984 | 0.000% | 100.000% |
| 188 | Psammodictyon | 0.1214286 | 0.0026553 | 0.0620419 | 0.000% | 100.000% |
| 189 | Spermatazoopis exultans | 0.0642857 | 0.0570887 | 0.0606872 | 0.000% | 100.000% |
| 190 | Nitzschia 3 | 0.1194018 | 0.0000000 | 0.0597009 | 0.000% | 100.000% |
| 191 | Campylosira | 0.0363073 | 0.0777031 | 0.0570052 | 0.000% | 100.000% |
| 192 | Nitzshia reversa | 0.0422078 | 0.0707257 | 0.0564668 | 0.000% | 100.000% |
| 193 | Botryococcus | 0.0000000 | 0.1078571 | 0.0539286 | 0.000% | 100.000% |
| 194 | Agmenellum | 0.0428571 | 0.0622294 | 0.0525433 | 0.000% | 100.000% |
| 195 | Anabaenopsis | 0.0000000 | 0.1049540 | 0.0524770 | 0.000% | 100.000% |
| 196 | Lithodesmium undulatum | 0.0180000 | 0.0861255 | 0.0520628 | 0.000% | 100.000% |
| 197 | Ceratium | 0.0238335 | 0.0802770 | 0.0520553 | 0.000% | 100.000% |
| 198 | Nitzschia sigmoidea | 0.0219528 | 0.0806886 | 0.0513207 | 0.000% | 100.000% |
| 199 | Golenkinia | 0.0000000 | 0.1025548 | 0.0512774 | 0.000% | 100.000% |
| 200 | Amphidinium | 0.0162321 | 0.0858333 | 0.0510327 | 0.000% | 100.000% |
| 201 | Bacillariophyta | 0.0363073 | 0.0612350 | 0.0487712 | 0.000% | 100.000% |
| 202 | Gymnodium mikimotoi | 0.0032464 | 0.0884157 | 0.0458311 | 0.000% | 100.000% |
| 203 | Staurastrum | 0.0071429 | 0.0831242 | 0.0451335 | 0.000% | 100.000% |
| 204 | Karenia brevis | 0.0000000 | 0.0875549 | 0.0437775 | 0.000% | 100.000% |
| 205 | Gloeactinium | 0.0000000 | 0.0854821 | 0.0427410 | 0.000% | 100.000% |
| 206 | Protoperidinium pellucidum | 0.0000065 | 0.0755109 | 0.0377587 | 0.000% | 100.000% |
| 207 | Diploneis chersonensis | 0.0000000 | 0.0729167 | 0.0364583 | 0.000% | 100.000% |
| 208 | Arthrospira | 0.0357143 | 0.0370671 | 0.0363907 | 0.000% | 100.000% |
| 209 | Centrodinium | 0.0097464 | 0.0612520 | 0.0354992 | 0.000% | 100.000% |
| 210 | Cosmeineis | 0.0000000 | 0.0701434 | 0.0350717 | 0.000% | 100.000% |
| 211 | Chaetoceros subtilis | 0.0194805 | 0.0429572 | 0.0312189 | 0.000% | 100.000% |
| 212 | Gloeocapsa | 0.0287863 | 0.0320721 | 0.0304292 | 0.000% | 100.000% |
| 213 | Lyngbya | 0.0000000 | 0.0585312 | 0.0292656 | 0.000% | 100.000% |
| 214 | Biddulphia | 0.0000000 | 0.0546540 | 0.0273270 | 0.000% | 100.000% |
| 215 | Treubaria | 0.0333189 | 0.0204312 | 0.0268751 | 0.000% | 100.000% |
| 216 | Nitzschia tryblionella | 0.0000000 | 0.0530303 | 0.0265152 | 0.000% | 100.000% |
| 217 | Filamentous Diatom | 0.0523170 | 0.0000000 | 0.0261585 | 0.000% | 100.000% |
| 218 | Phaeocystis | 0.0515668 | 0.0000000 | 0.0257834 | 0.000% | 100.000% |
| 219 | Gyrosigma scalproides | 0.0071429 | 0.0438988 | 0.0255208 | 0.000% | 100.000% |
| 220 | Nitzschia holsatica | 0.0000000 | 0.0505149 | 0.0252574 | 0.000% | 100.000% |
| 221 | Ulothrix | 0.0071429 | 0.0382170 | 0.0226799 | 0.000% | 100.000% |
| 222 | Nitzshia tryblionella | 0.0285714 | 0.0132576 | 0.0209145 | 0.000% | 100.000% |
| 223 | Crucigenia | 0.0142857 | 0.0274841 | 0.0208849 | 0.000% | 100.000% |
| 224 | Gonium | 0.0000000 | 0.0401685 | 0.0200843 | 0.000% | 100.000% |
| 225 | Cerataulina pelagica | 0.0000000 | 0.0379464 | 0.0189732 | 0.000% | 100.000% |
| 226 | Gyrodinium | 0.0000000 | 0.0379464 | 0.0189732 | 0.000% | 100.000% |
| 227 | Entomoneis | 0.0103978 | 0.0246249 | 0.0175113 | 0.000% | 100.000% |
| 228 | Euglena elastica | 0.0285714 | 0.0022321 | 0.0154018 | 0.000% | 100.000% |
| 229 | Gyrosigma macrum | 0.0000000 | 0.0290179 | 0.0145089 | 0.000% | 100.000% |
| 230 | Thalassiothrix | 0.0000000 | 0.0290179 | 0.0145089 | 0.000% | 100.000% |
| 231 | Volvocacae | 0.0287863 | 0.0000000 | 0.0143931 | 0.000% | 100.000% |
| 232 | Pediastrum simplex | 0.0071429 | 0.0175189 | 0.0123309 | 0.000% | 100.000% |
| 233 | Bacteriastrum delicatulum | 0.0238674 | 0.0000022 | 0.0119348 | 0.000% | 100.000% |
| 234 | Characium limneticum | 0.0142857 | 0.0094697 | 0.0118777 | 0.000% | 100.000% |
| 235 | Melosira mummuloides | 0.0064935 | 0.0101498 | 0.0083217 | 0.000% | 100.000% |
| 236 | Bacteriastrum | 0.0014286 | 0.0129796 | 0.0072041 | 0.000% | 100.000% |
| 237 | Ditylum brightwelli | 0.0142857 | 0.0000000 | 0.0071429 | 0.000% | 100.000% |
| 238 | Volvox | 0.0142857 | 0.0000000 | 0.0071429 | 0.000% | 100.000% |
| 239 | Procentrum caribbaeum | 0.0137143 | 0.0000000 | 0.0068571 | 0.000% | 100.000% |
| 240 | Denticula | 0.0000000 | 0.0134688 | 0.0067344 | 0.000% | 100.000% |
| 241 | Melosira granulata | 0.0000000 | 0.0133929 | 0.0066964 | 0.000% | 100.000% |
| 242 | Dictyosphaerium | 0.0000000 | 0.0132576 | 0.0066288 | 0.000% | 100.000% |
| 243 | Nitzschia granulata | 0.0000000 | 0.0120400 | 0.0060200 | 0.000% | 100.000% |
| 244 | Aphanizomenon | 0.0071429 | 0.0047840 | 0.0059634 | 0.000% | 100.000% |
| 245 | Arthrodesmus phimus | 0.0071429 | 0.0044643 | 0.0058036 | 0.000% | 100.000% |
| 246 | Rhizoclonium | 0.0000000 | 0.0113636 | 0.0056818 | 0.000% | 100.000% |
| 247 | Spirogyra | 0.0071429 | 0.0041278 | 0.0056353 | 0.000% | 100.000% |
| 248 | Trachelomonas | 0.0071429 | 0.0041261 | 0.0056345 | 0.000% | 100.000% |
| 249 | Gyrosigma balticum | 0.0000000 | 0.0111607 | 0.0055804 | 0.000% | 100.000% |
| 250 | Chaetoceros peruvianum | 0.0000353 | 0.0109837 | 0.0055095 | 0.000% | 100.000% |
| 251 | Caloneis | 0.0000000 | 0.0101914 | 0.0050957 | 0.000% | 100.000% |
| 252 | Guinardia striata | 0.0000000 | 0.0101461 | 0.0050731 | 0.000% | 100.000% |
| 253 | Ceratium fusus | 0.0000698 | 0.0096964 | 0.0048831 | 0.000% | 100.000% |
| 254 | Chaetoceros aequatorialis | 0.0005162 | 0.0091281 | 0.0048221 | 0.000% | 100.000% |
| 255 | Pediastrum | 0.0000000 | 0.0096269 | 0.0048135 | 0.000% | 100.000% |
| 256 | Protoperidinium conicum | 0.0083571 | 0.0007813 | 0.0045692 | 0.000% | 100.000% |
| 257 | Ceratium hircus | 0.0039379 | 0.0051559 | 0.0045469 | 0.000% | 100.000% |
| 258 | Lepocinclis | 0.0000000 | 0.0089286 | 0.0044643 | 0.000% | 100.000% |
| 259 | Prymnesiodhyceae | 0.0000000 | 0.0089248 | 0.0044624 | 0.000% | 100.000% |
| 260 | Prorocentrum micans | 0.0000000 | 0.0079552 | 0.0039776 | 0.000% | 100.000% |
| 261 | Chlorophyceae | 0.0000000 | 0.0076369 | 0.0038184 | 0.000% | 100.000% |
| 262 | Leptocylindricus | 0.0000000 | 0.0075790 | 0.0037895 | 0.000% | 100.000% |
| 263 | Plagiogramma tessellatum | 0.0071429 | 0.0000000 | 0.0035714 | 0.000% | 100.000% |
| 264 | Tropidonteis lepidoptera | 0.0071429 | 0.0000000 | 0.0035714 | 0.000% | 100.000% |
| 265 | Leptocylindricus danicus | 0.0000000 | 0.0060877 | 0.0030438 | 0.000% | 100.000% |
| 266 | Thalassionema nitzchioides | 0.0000000 | 0.0060877 | 0.0030438 | 0.000% | 100.000% |
| 267 | Hemiaulus hauckii | 0.0000000 | 0.0057802 | 0.0028901 | 0.000% | 100.000% |
| 268 | Pyrrophyta | 0.0000000 | 0.0056818 | 0.0028409 | 0.000% | 100.000% |
| 269 | Odontella mobiliensis | 0.0026518 | 0.0024457 | 0.0025487 | 0.000% | 100.000% |
| 270 | Stauroneis | 0.0000000 | 0.0041261 | 0.0020630 | 0.000% | 100.000% |
| 271 | Aulacosiera granulata | 0.0000000 | 0.0031807 | 0.0015903 | 0.000% | 100.000% |
| 272 | Hemiaulus sinensis | 0.0000082 | 0.0027364 | 0.0013723 | 0.000% | 100.000% |
| 273 | Pheopolykrikos | 0.0000000 | 0.0020292 | 0.0010146 | 0.000% | 100.000% |
| 274 | Tychonema | 0.0000071 | 0.0018939 | 0.0009505 | 0.000% | 100.000% |
| 275 | Micrasterias | 0.0000000 | 0.0018939 | 0.0009470 | 0.000% | 100.000% |
| 276 | Oocardium | 0.0000000 | 0.0018939 | 0.0009470 | 0.000% | 100.000% |
| 277 | Ceratium furca | 0.0000000 | 0.0018793 | 0.0009396 | 0.000% | 100.000% |
| 278 | Lithodesmium | 0.0012697 | 0.0002425 | 0.0007561 | 0.000% | 100.000% |
| 279 | Odontella rhombus | 0.0000172 | 0.0012275 | 0.0006223 | 0.000% | 100.000% |
| 280 | Netrium | 0.0000000 | 0.0010156 | 0.0005078 | 0.000% | 100.000% |
| 281 | Closteriopsis acicularis | 0.0000000 | 0.0010146 | 0.0005073 | 0.000% | 100.000% |
| 282 | Coelastrum microporum | 0.0000000 | 0.0010146 | 0.0005073 | 0.000% | 100.000% |
| 283 | Chaetoceros thorondensi | 0.0000000 | 0.0008609 | 0.0004304 | 0.000% | 100.000% |
| 284 | Bacteriastrum hyalinum | 0.0000000 | 0.0008252 | 0.0004126 | 0.000% | 100.000% |
| 285 | Nitzschia gyrosignma | 0.0000000 | 0.0007813 | 0.0003906 | 0.000% | 100.000% |
| 286 | Dactyliosolen | 0.0000000 | 0.0004464 | 0.0002232 | 0.000% | 100.000% |
| 287 | Chaetoceros curvisetus | 0.0000000 | 0.0003503 | 0.0001751 | 0.000% | 100.000% |
| 288 | Procentrum gracile | 0.0000000 | 0.0003220 | 0.0001610 | 0.000% | 100.000% |
| 289 | Chaetoceros danicus | 0.0000000 | 0.0001534 | 0.0000767 | 0.000% | 100.000% |
| 290 | Dilichospermum | 0.0000251 | 0.0000566 | 0.0000409 | 0.000% | 100.000% |
| 291 | Fliamentous Green | 0.0000395 | 0.0000038 | 0.0000217 | 0.000% | 100.000% |
| 292 | Astermophalus | 0.0000302 | 0.0000000 | 0.0000151 | 0.000% | 100.000% |
| 293 | Chaetoceros diadema | 0.0000000 | 0.0000147 | 0.0000074 | 0.000% | 100.000% |
| 294 | Chaetoceros affinis | 0.0000000 | 0.0000123 | 0.0000061 | 0.000% | 100.000% |
| 295 | Lioloma pacificum | 0.0000000 | 0.0000078 | 0.0000039 | 0.000% | 100.000% |
| 296 | Ceratium lineatum | 0.0000000 | 0.0000026 | 0.0000013 | 0.000% | 100.000% |
| 297 | Protoperidinium quaraerense | 0.0000000 | 0.0000023 | 0.0000012 | 0.000% | 100.000% |
| 298 | Pleurotaenium | 0.0000000 | 0.0000013 | 0.0000007 | 0.000% | 100.000% |
| 299 | Actinoptychus undulatus | 0.0000000 | 0.0000000 | 0.0000000 | 0.000% | 100.000% |
| 300 | Dinobryon | 0.0000000 | 0.0000000 | 0.0000000 | 0.000% | 100.000% |
|  | Total | 452891.9991590 | 301845.4900803 | 377368.7446197 |  |  |

## Zooplankton

Zooplankton abundance (n/m^3^) averaged over all sample collection periods.

| **Rank** | **Taxa/Species Name** | **Distance from Discharge** | | **Mean** | **Pct** | **CumPct** |
| --- | --- | --- | --- | --- | --- | --- |
|  |  | **B** | **R** |  |  |  |
| 1 | Protozoa | 3143847.60 | 6431070.41 | 4787459.01 | 45.51% | 45.51% |
| 2 | Ciliata | 2347500.98 | 3709737.27 | 3028619.12 | 28.79% | 74.30% |
| 3 | Myrionecta rubra | 810006.49 | 938042.19 | 874024.34 | 8.31% | 82.61% |
| 4 | Amoebacea | 0.00 | 1376909.72 | 688454.86 | 6.54% | 89.16% |
| 5 | Eutintinnus tubulosa | 346254.23 | 284584.05 | 315419.14 | 3.00% | 92.15% |
| 6 | Tintinnopsis parvula | 119988.47 | 288857.91 | 204423.19 | 1.94% | 94.10% |
| 7 | Tintinnidium | 97403.38 | 131781.72 | 114592.55 | 1.09% | 95.19% |
| 8 | Eutintinnus tenuis | 48710.08 | 105922.37 | 77316.22 | 0.73% | 95.92% |
| 9 | Tintinnopsis tubulosa | 83932.13 | 60994.88 | 72463.51 | 0.69% | 96.61% |
| 10 | Rotifera | 45784.51 | 49044.91 | 47414.71 | 0.45% | 97.06% |
| 11 | Tintinnopsis beroidea | 22997.84 | 64789.82 | 43893.83 | 0.42% | 97.48% |
| 12 | Tintinnopsis tocantinensis | 34189.35 | 41631.47 | 37910.41 | 0.36% | 97.84% |
| 13 | Strobilidium | 29220.42 | 36432.34 | 32826.38 | 0.31% | 98.15% |
| 14 | Euplotes | 22725.97 | 40020.75 | 31373.36 | 0.30% | 98.45% |
| 15 | Didinium | 22734.34 | 33205.59 | 27969.96 | 0.27% | 98.71% |
| 16 | Strobilidiidae | 9739.29 | 38983.82 | 24361.55 | 0.23% | 98.95% |
| 17 | Copepoda | 22387.27 | 24820.50 | 23603.88 | 0.22% | 99.17% |
| 18 | Bivalve larvae | 24100.38 | 3834.28 | 13967.33 | 0.13% | 99.30% |
| 19 | Strombidium | 8203.79 | 14918.24 | 11561.01 | 0.11% | 99.41% |
| 20 | Tintinnopsis | 16431.93 | 4976.51 | 10704.22 | 0.10% | 99.52% |
| 21 | Brachionus rubens | 1932.90 | 8404.80 | 5168.85 | 0.05% | 99.56% |
| 22 | Tintinnopsis levigata | 6521.44 | 3401.37 | 4961.41 | 0.05% | 99.61% |
| 23 | Tintinnopsis mortensis | 3105.36 | 5643.80 | 4374.58 | 0.04% | 99.65% |
| 24 | Cyclopoida | 3718.81 | 4040.64 | 3879.73 | 0.04% | 99.69% |
| 25 | Cell | 3316.13 | 2619.95 | 2968.04 | 0.03% | 99.72% |
| 26 | Polychaeta | 2572.67 | 3136.77 | 2854.72 | 0.03% | 99.75% |
| 27 | Filinia cornuta | 3246.75 | 2185.31 | 2716.03 | 0.03% | 99.77% |
| 28 | Calanoida | 2108.06 | 3093.91 | 2600.99 | 0.02% | 99.80% |
| 29 | Foraminifera | 3662.03 | 1187.39 | 2424.71 | 0.02% | 99.82% |
| 30 | Coxliella longa | 12.94 | 4208.37 | 2110.65 | 0.02% | 99.84% |
| 31 | Amoeba | 0.00 | 3281.78 | 1640.89 | 0.02% | 99.85% |
| 32 | Trichocerca | 3259.85 | 16.16 | 1638.00 | 0.02% | 99.87% |
| 33 | Synchaeta | 877.31 | 1696.46 | 1286.88 | 0.01% | 99.88% |
| 34 | Astacidae | 3.84 | 2166.93 | 1085.38 | 0.01% | 99.89% |
| 35 | Eutintinnus | 0.00 | 2104.38 | 1052.19 | 0.01% | 99.90% |
| 36 | Gastropoda Larva | 1029.64 | 1021.67 | 1025.65 | 0.01% | 99.91% |
| 37 | Zooplankton Unid | 266.31 | 1348.04 | 807.18 | 0.01% | 99.92% |
| 38 | Balanus | 899.24 | 714.03 | 806.63 | 0.01% | 99.93% |
| 39 | Oikopleura | 272.66 | 1228.73 | 750.69 | 0.01% | 99.94% |
| 40 | Synchaeta bicornis | 43.90 | 1178.32 | 611.11 | 0.01% | 99.94% |
| 41 | Brachionus angularis | 0.00 | 1183.79 | 591.90 | 0.01% | 99.95% |
| 42 | Tintinnopsis brandti | 0.00 | 1092.55 | 546.27 | 0.01% | 99.95% |
| 43 | Petalotricha | 763.17 | 324.46 | 543.81 | 0.01% | 99.96% |
| 44 | Brachionus urceolaris | 452.83 | 620.85 | 536.84 | 0.01% | 99.96% |
| 45 | Ceratium fusus | 130.58 | 648.92 | 389.75 | 0.00% | 99.97% |
| 46 | Trochophore larvae | 506.86 | 139.48 | 323.17 | 0.00% | 99.97% |
| 47 | Caligus | 468.51 | 133.56 | 301.04 | 0.00% | 99.97% |
| 48 | Pelecypoda larvae | 316.54 | 202.75 | 259.64 | 0.00% | 99.97% |
| 49 | Brachionus | 186.38 | 329.76 | 258.07 | 0.00% | 99.98% |
| 50 | Cirripedia Nauplius | 274.51 | 231.99 | 253.25 | 0.00% | 99.98% |
| 51 | Egg | 245.05 | 165.91 | 205.48 | 0.00% | 99.98% |
| 52 | Favella | 206.85 | 202.99 | 204.92 | 0.00% | 99.98% |
| 53 | Asplanchna | 217.40 | 152.36 | 184.88 | 0.00% | 99.98% |
| 54 | Planula | 112.20 | 185.70 | 148.95 | 0.00% | 99.99% |
| 55 | Cnidaria planula | 0.00 | 262.23 | 131.12 | 0.00% | 99.99% |
| 56 | Codonella | 140.22 | 112.05 | 126.13 | 0.00% | 99.99% |
| 57 | Nematoda | 204.96 | 38.13 | 121.55 | 0.00% | 99.99% |
| 58 | Ceratium | 77.95 | 135.78 | 106.87 | 0.00% | 99.99% |
| 59 | Harpacticoida | 176.96 | 23.31 | 100.13 | 0.00% | 99.99% |
| 60 | Acartia tonsa | 59.53 | 131.17 | 95.35 | 0.00% | 99.99% |
| 61 | Oithona | 95.60 | 64.70 | 80.15 | 0.00% | 99.99% |
| 62 | Annelida | 89.18 | 68.98 | 79.08 | 0.00% | 99.99% |
| 63 | Flavella panamensis | 69.24 | 44.32 | 56.78 | 0.00% | 99.99% |
| 64 | Lecane | 38.51 | 71.86 | 55.19 | 0.00% | 100.00% |
| 65 | Condonellopsis | 54.62 | 49.80 | 52.21 | 0.00% | 100.00% |
| 66 | Vorticella | 79.84 | 13.72 | 46.78 | 0.00% | 100.00% |
| 67 | Oithiona colcarva | 25.32 | 63.08 | 44.20 | 0.00% | 100.00% |
| 68 | Monostyla | 84.52 | 1.78 | 43.15 | 0.00% | 100.00% |
| 69 | Microcyclops | 43.60 | 34.26 | 38.93 | 0.00% | 100.00% |
| 70 | Oligotrichida | 17.96 | 39.75 | 28.85 | 0.00% | 100.00% |
| 71 | Bdelloidea | 31.01 | 19.12 | 25.06 | 0.00% | 100.00% |
| 72 | Udella | 11.90 | 35.82 | 23.86 | 0.00% | 100.00% |
| 73 | Bryocamptus | 16.43 | 28.69 | 22.56 | 0.00% | 100.00% |
| 74 | Saphirella | 20.93 | 21.82 | 21.38 | 0.00% | 100.00% |
| 75 | Dinoflagellates | 21.29 | 14.56 | 17.93 | 0.00% | 100.00% |
| 76 | Conchoecia | 13.39 | 8.89 | 11.14 | 0.00% | 100.00% |
| 77 | Calocalanus | 20.83 | 1.45 | 11.14 | 0.00% | 100.00% |
| 78 | Coconella | 0.00 | 22.26 | 11.13 | 0.00% | 100.00% |
| 79 | Ceratium hircus | 13.39 | 8.39 | 10.89 | 0.00% | 100.00% |
| 80 | Keratella | 8.35 | 10.18 | 9.27 | 0.00% | 100.00% |
| 81 | Neritidae | 6.60 | 9.47 | 8.04 | 0.00% | 100.00% |
| 82 | Appendicularia larvae | 3.72 | 9.48 | 6.60 | 0.00% | 100.00% |
| 83 | Epistylis | 10.71 | 0.00 | 5.36 | 0.00% | 100.00% |
| 84 | Parafavella | 4.46 | 5.85 | 5.16 | 0.00% | 100.00% |
| 85 | Tintinnopsella | 0.00 | 9.10 | 4.55 | 0.00% | 100.00% |
| 86 | Keratella valga | 7.97 | 0.00 | 3.99 | 0.00% | 100.00% |
| 87 | Hexarthra | 0.00 | 7.39 | 3.69 | 0.00% | 100.00% |
| 88 | Gastropus | 4.46 | 2.29 | 3.38 | 0.00% | 100.00% |
| 89 | Mnemiopsis leidyi | 0.30 | 6.21 | 3.26 | 0.00% | 100.00% |
| 90 | Actinolaimus | 0.00 | 6.08 | 3.04 | 0.00% | 100.00% |
| 91 | Ostracoda | 5.58 | 0.42 | 3.00 | 0.00% | 100.00% |
| 92 | Hydracarina | 4.48 | 1.45 | 2.97 | 0.00% | 100.00% |
| 93 | Cumacea | 0.00 | 5.71 | 2.85 | 0.00% | 100.00% |
| 94 | Rhabdonella | 1.49 | 3.00 | 2.25 | 0.00% | 100.00% |
| 95 | Daphnia | 3.86 | 0.50 | 2.18 | 0.00% | 100.00% |
| 96 | Notommata | 1.49 | 2.30 | 1.89 | 0.00% | 100.00% |
| 97 | Sida | 0.00 | 3.23 | 1.61 | 0.00% | 100.00% |
| 98 | Crustacea | 0.00 | 3.15 | 1.57 | 0.00% | 100.00% |
| 99 | Paracalanus crassirostris | 1.49 | 1.37 | 1.43 | 0.00% | 100.00% |
| 100 | Cladocera | 0.00 | 2.78 | 1.39 | 0.00% | 100.00% |
| 101 | Hydromedusa | 0.68 | 2.03 | 1.36 | 0.00% | 100.00% |
| 102 | Cyprid larva | 0.00 | 2.64 | 1.32 | 0.00% | 100.00% |
| 103 | Mytilina | 1.49 | 0.97 | 1.23 | 0.00% | 100.00% |
| 104 | Epiplocylis | 1.49 | 0.81 | 1.15 | 0.00% | 100.00% |
| 105 | Filinia | 0.73 | 1.47 | 1.10 | 0.00% | 100.00% |
| 106 | Notholca | 0.00 | 2.18 | 1.09 | 0.00% | 100.00% |
| 107 | Surirella | 0.00 | 1.97 | 0.98 | 0.00% | 100.00% |
| 108 | Acartia | 0.00 | 1.91 | 0.95 | 0.00% | 100.00% |
| 109 | Polyarthra | 0.00 | 1.76 | 0.88 | 0.00% | 100.00% |
| 110 | Filinia longiseta | 0.00 | 1.75 | 0.88 | 0.00% | 100.00% |
| 111 | Nereis | 0.00 | 1.70 | 0.85 | 0.00% | 100.00% |
| 112 | Amphorellopsis acuta | 0.00 | 1.69 | 0.84 | 0.00% | 100.00% |
| 113 | Gastrotricha | 0.00 | 1.63 | 0.81 | 0.00% | 100.00% |
| 114 | Zooflagella | 0.00 | 1.63 | 0.81 | 0.00% | 100.00% |
| 115 | Lecane luna | 0.00 | 1.59 | 0.79 | 0.00% | 100.00% |
| 116 | Kellicottia | 0.00 | 1.50 | 0.75 | 0.00% | 100.00% |
| 117 | Proplectella | 0.00 | 1.50 | 0.75 | 0.00% | 100.00% |
| 118 | Halocypris | 1.49 | 0.00 | 0.74 | 0.00% | 100.00% |
| 119 | Squatinella | 1.49 | 0.00 | 0.74 | 0.00% | 100.00% |
| 120 | Corixidae | 0.00 | 1.30 | 0.65 | 0.00% | 100.00% |
| 121 | Ploesoma | 0.00 | 1.15 | 0.58 | 0.00% | 100.00% |
| 122 | Brachionus calyciflorus | 0.00 | 1.14 | 0.57 | 0.00% | 100.00% |
| 123 | Decapoda Zoea | 0.01 | 0.94 | 0.47 | 0.00% | 100.00% |
| 124 | Cyphonaute | 0.00 | 0.94 | 0.47 | 0.00% | 100.00% |
| 125 | Ophiura larvae | 0.00 | 0.94 | 0.47 | 0.00% | 100.00% |
| 126 | Undella | 0.00 | 0.81 | 0.41 | 0.00% | 100.00% |
| 127 | Ploima | 0.33 | 0.47 | 0.40 | 0.00% | 100.00% |
| 128 | Bosmidia | 0.00 | 0.58 | 0.29 | 0.00% | 100.00% |
| 129 | Amphorides | 0.00 | 0.50 | 0.25 | 0.00% | 100.00% |
| 130 | Dictyocysta | 0.00 | 0.50 | 0.25 | 0.00% | 100.00% |
| 131 | Stebosemella | 0.00 | 0.50 | 0.25 | 0.00% | 100.00% |
| 132 | Ascampbelliella | 0.00 | 0.48 | 0.24 | 0.00% | 100.00% |
| 133 | Cymatocylis | 0.00 | 0.48 | 0.24 | 0.00% | 100.00% |
| 134 | Xystonellopsis | 0.00 | 0.48 | 0.24 | 0.00% | 100.00% |
| 135 | Euchlanis | 0.00 | 0.41 | 0.20 | 0.00% | 100.00% |
| 136 | Paragrolaimus | 0.00 | 0.41 | 0.20 | 0.00% | 100.00% |
| 137 | Thysanoptera | 0.00 | 0.35 | 0.17 | 0.00% | 100.00% |
| 138 | Arthropoda | 0.00 | 0.21 | 0.10 | 0.00% | 100.00% |
| 139 | Scyphozoa | 0.00 | 0.12 | 0.06 | 0.00% | 100.00% |
| 140 | Hymenoptera | 0.00 | 0.08 | 0.04 | 0.00% | 100.00% |
| 141 | Paracalanus | 0.00 | 0.05 | 0.02 | 0.00% | 100.00% |
| 142 | Oithona nana | 0.01 | 0.02 | 0.01 | 0.00% | 100.00% |
| 143 | Tunicate larvae | 0.00 | 0.01 | 0.01 | 0.00% | 100.00% |
| 144 | Prionchulus | 0.00 | 0.01 | 0.00 | 0.00% | 100.00% |
| 145 | Tetramastix | 0.00 | 0.01 | 0.00 | 0.00% | 100.00% |
| 146 | Ceriodaphnia | 0.00 | 0.00 | 0.00 | 0.00% | 100.00% |
| 147 | Bosmina | 0.00 | 0.00 | 0.00 | 0.00% | 100.00% |
| 148 | Moina | 0.00 | 0.00 | 0.00 | 0.00% | 100.00% |
| 149 | Oithiona simplex | 0.00 | 0.00 | 0.00 | 0.00% | 100.00% |
| 150 | Isopoda | 0.00 | 0.00 | 0.00 | 0.00% | 100.00% |
| 151 | Clausocalonus | 0.00 | 0.00 | 0.00 | 0.00% | 100.00% |
| 152 | Cypridopsis | 0.00 | 0.00 | 0.00 | 0.00% | 100.00% |
| 153 | Microstella | 0.00 | 0.00 | 0.00 | 0.00% | 100.00% |
|  | Total | 7298369.04 | 13740300.09 | 10519334.57 |  |  |

## Ichthyoplankton

Abundance (n/5-min tow) of ichthyoplankton averaged over all sampling periods.

| **Rank** | **Taxa/Species Name** | **Distance from Discharge** | | | **Mean** | **Pct** | **CumPct** |
| --- | --- | --- | --- | --- | --- | --- | --- |
|  |  | **B** | **C** | **R** |  |  |  |
| 1 | Anchoa mitchilli | 11.1391 | 10.1292 | 8.2462 | 9.8382 | 30.117% | 30.117% |
| 2 | Clupeidae | 5.7605 | 5.1770 | 5.6313 | 5.5230 | 16.907% | 47.023% |
| 3 | Brevoortia patronus | 3.6055 | 3.1589 | 5.5925 | 4.1190 | 12.609% | 59.632% |
| 4 | Gobiosoma bosci | 4.9332 | 4.5420 | 1.7231 | 3.7328 | 11.427% | 71.059% |
| 5 | Engraulidae | 1.3539 | 1.1173 | 2.7586 | 1.7433 | 5.336% | 76.396% |
| 6 | Clupeid egg | 0.0000 | 0.0000 | 2.7557 | 0.9186 | 2.812% | 79.207% |
| 7 | Menidia beryllina | 1.1717 | 0.8454 | 0.6279 | 0.8817 | 2.699% | 81.906% |
| 8 | Anchoa egg | 0.0000 | 0.0000 | 2.6216 | 0.8739 | 2.675% | 84.581% |
| 9 | Gobiesox strumosus | 0.5614 | 0.3450 | 1.2059 | 0.7041 | 2.155% | 86.737% |
| 10 | Micropogonias undulatus | 0.6128 | 1.1948 | 0.1424 | 0.6500 | 1.990% | 88.727% |
| 11 | Sciaenid egg | 0.0000 | 0.0000 | 1.8117 | 0.6039 | 1.849% | 90.575% |
| 12 | Lagodon rhomboides | 0.1724 | 0.2749 | 1.0836 | 0.5103 | 1.562% | 92.137% |
| 13 | Hypsoblennius hentz | 0.4428 | 0.4579 | 0.4803 | 0.4603 | 1.409% | 93.547% |
| 14 | Anchoa | 0.3840 | 0.3825 | 0.5947 | 0.4537 | 1.389% | 94.935% |
| 15 | Tetradontidae | 0.3846 | 0.2509 | 0.5899 | 0.4085 | 1.250% | 96.186% |
| 16 | Microgobius thalassinus | 0.3498 | 0.2361 | 0.0470 | 0.2110 | 0.646% | 96.832% |
| 17 | Anchoa hepsetus | 0.2327 | 0.2488 | 0.0491 | 0.1769 | 0.541% | 97.373% |
| 18 | Menidia peninsulae | 0.1912 | 0.1036 | 0.1037 | 0.1329 | 0.407% | 97.780% |
| 19 | Blenniidae | 0.0677 | 0.0963 | 0.0790 | 0.0810 | 0.248% | 98.028% |
| 20 | Leiostomus xanthurus | 0.0513 | 0.0507 | 0.1128 | 0.0716 | 0.219% | 98.247% |
| 21 | Acetes americanus | 0.0000 | 0.0000 | 0.1439 | 0.0480 | 0.147% | 98.394% |
| 22 | Sphoeroides parvus | 0.0466 | 0.0434 | 0.0518 | 0.0473 | 0.145% | 98.539% |
| 23 | Micropogon undulatus | 0.0606 | 0.0650 | 0.0048 | 0.0435 | 0.133% | 98.672% |
| 24 | Bairdiella chrysoura | 0.0349 | 0.0193 | 0.0516 | 0.0353 | 0.108% | 98.780% |
| 25 | Menidia | 0.0047 | 0.0240 | 0.0733 | 0.0340 | 0.104% | 98.884% |
| 26 | Membras martinica | 0.0442 | 0.0169 | 0.0298 | 0.0303 | 0.093% | 98.977% |
| 27 | Goby | 0.0000 | 0.0048 | 0.0738 | 0.0262 | 0.080% | 99.057% |
| 28 | Cynoscion nebulosus | 0.0396 | 0.0266 | 0.0098 | 0.0253 | 0.078% | 99.134% |
| 29 | Menidia penisulae | 0.0232 | 0.0120 | 0.0397 | 0.0250 | 0.076% | 99.211% |
| 30 | Hypsoblennius | 0.0256 | 0.0121 | 0.0272 | 0.0216 | 0.066% | 99.277% |
| 31 | Lolliguncula brevis | 0.0186 | 0.0096 | 0.0314 | 0.0199 | 0.061% | 99.338% |
| 32 | Gobiidae | 0.0140 | 0.0217 | 0.0198 | 0.0185 | 0.057% | 99.395% |
| 33 | Syngnathidae | 0.0186 | 0.0169 | 0.0196 | 0.0184 | 0.056% | 99.451% |
| 34 | Elops saurus | 0.0210 | 0.0193 | 0.0144 | 0.0182 | 0.056% | 99.507% |
| 35 | Synodus foetens | 0.0163 | 0.0193 | 0.0100 | 0.0152 | 0.047% | 99.553% |
| 36 | Gobionellus boleosoma | 0.0000 | 0.0072 | 0.0350 | 0.0141 | 0.043% | 99.596% |
| 37 | Mugil cephalus | 0.0093 | 0.0048 | 0.0171 | 0.0104 | 0.032% | 99.628% |
| 38 | Pogonias cromis | 0.0093 | 0.0169 | 0.0049 | 0.0103 | 0.032% | 99.660% |
| 39 | Archosargus probatocephalus | 0.0093 | 0.0096 | 0.0075 | 0.0088 | 0.027% | 99.687% |
| 40 | Gobiosoma robustum | 0.0164 | 0.0072 | 0.0024 | 0.0087 | 0.027% | 99.713% |
| 41 | Synodontidae | 0.0070 | 0.0048 | 0.0125 | 0.0081 | 0.025% | 99.738% |
| 42 | Myrophis punctatus | 0.0047 | 0.0000 | 0.0171 | 0.0073 | 0.022% | 99.761% |
| 43 | Trinectes maculatus | 0.0070 | 0.0073 | 0.0050 | 0.0064 | 0.020% | 99.780% |
| 44 | Skeletonema costatum | 0.0116 | 0.0073 | 0.0000 | 0.0063 | 0.019% | 99.799% |
| 45 | Ophichthus gomesi | 0.0070 | 0.0048 | 0.0051 | 0.0056 | 0.017% | 99.817% |
| 46 | Orthopristis chrysopterus | 0.0093 | 0.0072 | 0.0000 | 0.0055 | 0.017% | 99.833% |
| 47 | Oligoplites saurus | 0.0140 | 0.0000 | 0.0000 | 0.0047 | 0.014% | 99.848% |
| 48 | Cyprinodon variegatus | 0.0070 | 0.0024 | 0.0000 | 0.0031 | 0.010% | 99.857% |
| 49 | Cyprinodon | 0.0093 | 0.0000 | 0.0000 | 0.0031 | 0.009% | 99.867% |
| 50 | Symphurus plagiusa | 0.0000 | 0.0000 | 0.0077 | 0.0026 | 0.008% | 99.875% |
| 51 | Anguilliformes | 0.0000 | 0.0024 | 0.0050 | 0.0025 | 0.008% | 99.882% |
| 52 | Citharichthys spilopterus | 0.0023 | 0.0024 | 0.0026 | 0.0024 | 0.007% | 99.890% |
| 53 | Chloroscombrus chrysurus | 0.0023 | 0.0024 | 0.0025 | 0.0024 | 0.007% | 99.897% |
| 54 | Astroscopus ygraecum | 0.0000 | 0.0000 | 0.0073 | 0.0024 | 0.007% | 99.904% |
| 55 | Brevoortia | 0.0000 | 0.0072 | 0.0000 | 0.0024 | 0.007% | 99.912% |
| 56 | Achirus lineatus | 0.0023 | 0.0000 | 0.0049 | 0.0024 | 0.007% | 99.919% |
| 57 | Cynoscion arenAriopsis | 0.0000 | 0.0000 | 0.0051 | 0.0017 | 0.005% | 99.924% |
| 58 | Atherinidae | 0.0000 | 0.0024 | 0.0024 | 0.0016 | 0.005% | 99.929% |
| 59 | Paralichthys lethostigma | 0.0000 | 0.0000 | 0.0048 | 0.0016 | 0.005% | 99.934% |
| 60 | Balistidae | 0.0023 | 0.0024 | 0.0000 | 0.0016 | 0.005% | 99.939% |
| 61 | Sciaenops ocellata | 0.0023 | 0.0024 | 0.0000 | 0.0016 | 0.005% | 99.944% |
| 62 | Penaeus setiferus | 0.0047 | 0.0000 | 0.0000 | 0.0016 | 0.005% | 99.949% |
| 63 | Ctenogobius boleosoma | 0.0000 | 0.0000 | 0.0026 | 0.0009 | 0.003% | 99.951% |
| 64 | Eucinostomus argenteus | 0.0000 | 0.0000 | 0.0026 | 0.0009 | 0.003% | 99.954% |
| 65 | Caranx hippos | 0.0000 | 0.0000 | 0.0025 | 0.0008 | 0.003% | 99.956% |
| 66 | Hogchoker egg | 0.0000 | 0.0000 | 0.0025 | 0.0008 | 0.003% | 99.959% |
| 67 | Hyporhamphus meeki | 0.0000 | 0.0024 | 0.0000 | 0.0008 | 0.002% | 99.961% |
| 68 | Strongylura marina | 0.0000 | 0.0000 | 0.0024 | 0.0008 | 0.002% | 99.964% |
| 69 | Ariopsis felis | 0.0000 | 0.0024 | 0.0000 | 0.0008 | 0.002% | 99.966% |
| 70 | Centrarchid | 0.0000 | 0.0024 | 0.0000 | 0.0008 | 0.002% | 99.969% |
| 71 | Chilomycterus schoepfi | 0.0000 | 0.0000 | 0.0024 | 0.0008 | 0.002% | 99.971% |
| 72 | Lucania parva | 0.0000 | 0.0024 | 0.0000 | 0.0008 | 0.002% | 99.974% |
| 73 | Microgobius gulosus | 0.0000 | 0.0024 | 0.0000 | 0.0008 | 0.002% | 99.976% |
| 74 | Syngnathus scovelli | 0.0000 | 0.0000 | 0.0024 | 0.0008 | 0.002% | 99.979% |
| 75 | Triglidae | 0.0000 | 0.0024 | 0.0000 | 0.0008 | 0.002% | 99.981% |
| 76 | Carangidae | 0.0023 | 0.0000 | 0.0000 | 0.0008 | 0.002% | 99.983% |
| 77 | Microdesmus longipinnis | 0.0023 | 0.0000 | 0.0000 | 0.0008 | 0.002% | 99.986% |
| 78 | Plankton Unid | 0.0023 | 0.0000 | 0.0000 | 0.0008 | 0.002% | 99.988% |
| 79 | Prionotus tribulus | 0.0023 | 0.0000 | 0.0000 | 0.0008 | 0.002% | 99.991% |
| 80 | Tetradesmus | 0.0023 | 0.0000 | 0.0000 | 0.0008 | 0.002% | 99.993% |
| 81 | Dorosoma petenense | 0.0023 | 0.0000 | 0.0000 | 0.0008 | 0.002% | 99.995% |
| 82 | Leptocephalus larvae | 0.0023 | 0.0000 | 0.0000 | 0.0008 | 0.002% | 99.998% |
| 83 | Syngnathus louisianae | 0.0023 | 0.0000 | 0.0000 | 0.0008 | 0.002% | 100.000% |
|  | Total | 31.9389 | 29.0379 | 37.0241 | 32.6670 |  |  |

# Figure S1: Time Series Plots

Figure S1a. Aluminum (Al) values in sediment overtime (mg/kg) 57

Figure S1b Copper(Cu) values in sediment overtime (mg/kg) 58

Figure S1c. Mercury (Hg) values in sediment overtime (mg/kg) 59

Figure S1d. Lead (Pb) values in sediment overtime (mg/kg) 60

Figure S1e. Zinc (Zn) concentrations in sediment overtime (mg/kg) 61

Figure S1f. Chemical oxygen demand (COD) values in water overtime (mg/L) 62

Figure S1g. Dissolved oxygen (DO) concentrations in water overtime (mg/L) 63

Figure S1h. Dissolved organic nitrogen (DON) values in water overtime (mg-N/kg) 64

Figure S1i. Orthophosphate (PO4) values in water overtime (mg P/L) 65

Figure S1j. pH values in water overtime (S.U.) 66

Figure S1k. Sulphate (SO4) concentrations in water overtime (mg S/L) 67

Figure S1l. Salinity values in water overtime (PSU) 68

Figure S1m. Temperature values in water overtime (C°) 69

Figure S1n. Turbidity values in water overtime (NTU) 70

Figure S1o. Total organic carbon (TOC) values in water overtime (mg/L). 71

Figure S1p. Sand, silt, and clay sediment content overtime (%). 72

Figure S1q. Benthic abundance (log_10_ n/m2) and richness (S/0.023 m2 sample) from 1993 to 2020 73

Figure S1r. Phytoplankton abundance (n/mL), log abundance (log10 n/mL) and richness (S/2 L sample) over time 75

Figure S1s. Zooplankton abundance (log_10_ n/m3) and richness (S/48 L sample) overtime 77

Figure S1t. Gillnet abundance (log_10_ n/24 h set) and richness (S/sample) overtime 78

Figure S1w. Ichthyoplankton abundance (log_10_ n/5 min tow) and richness (S/sample) overtime 79

Figure S1x. Trawl abundance (log_10_ n/5 min tow) and richness (S/sample) overtime 80

Figure S1y. Water quality PC1 sample scores for sampling dates. Symbols represent climatic periods, where W = wet, A = average, D = dry. PC2 sample scores for sampling dates where symbols represent seasons, 1 = winter, 2 = spring, 3= s summer, 4 = fall 81

Figure S1z. Sediment PC1 for all trips. A) Sampling scores for PC1 where symbols represent climatic periods. W = wet, A = average, D = dry. B) Seasonality sample scores for PC1 where symbols represent seasonality. 1 = winter, 2 = spring, 3= summer, 4 = fall 82

Figure S1aa. Sediment PC2 for all trips. A) Sampling scores for PC2 where symbols represent climatic periods. W = wet, A = average, D = dry. B) Seasonality sample scores for PC2 where symbols represent seasonality. 1 = winter, 2 = spring, 3= summer, 4. 83

Aluminum values fluctuated between low and high from 1993 to 2004 and then began to level off after 2004 (Fig. S1a).


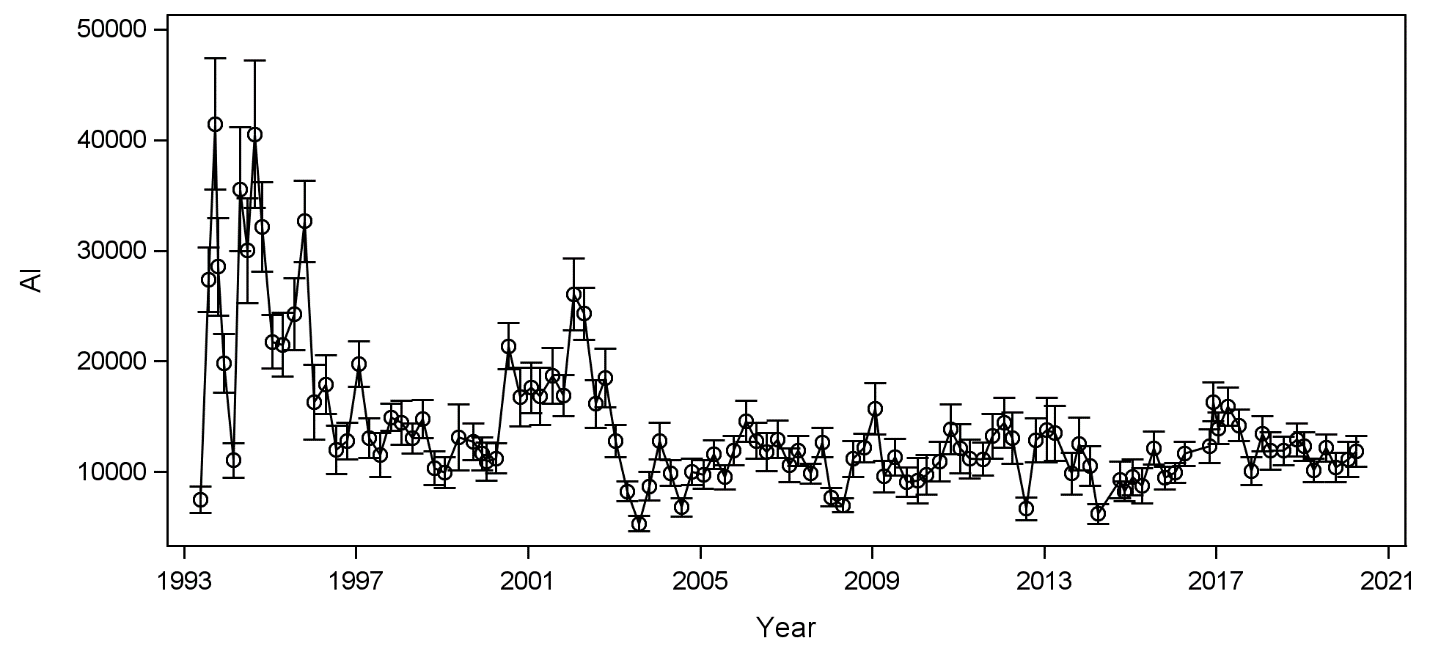


Figure S1a. Aluminum (Al) values in sediment overtime (mg/kg).

Figure S1b Copper(Cu) values in sediment overtime (mg/kg).

Figure S2a. Aluminum (Al) values in sediment overtime (mg/kg).

Copper values show elevated copper concentration spikes in 1995 and 2020 but a general decrease over time (Figure Figure S1b Copper(Cu) values in sediment overtime (mg/kg).

S1b).


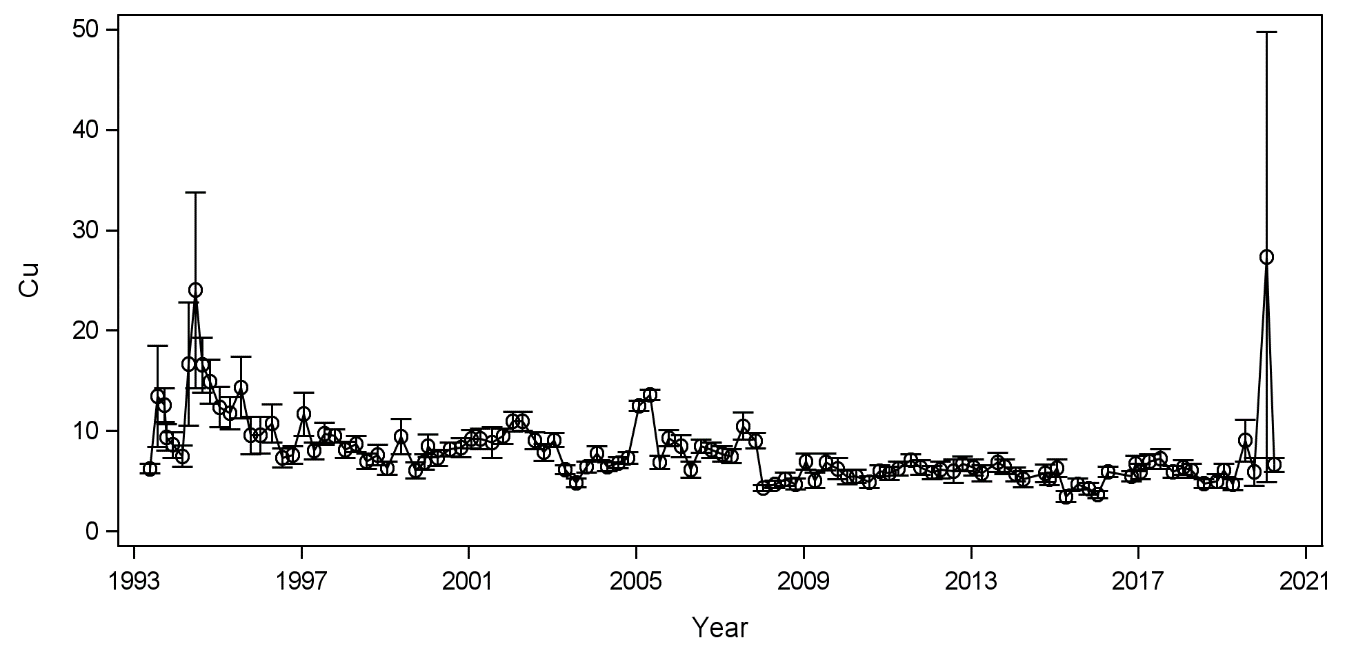


Figure S1b Copper(Cu) values in sediment overtime (mg/kg).

Mercury concentrations begin at ~0.06 in 1993, rise to the largest concentration documented for mercury at ~0.65 mg/kg in 1994, and then gradually decline in concentration overtime until 2015 with a spike of 0.58 mg/kg. Mercury concentrations plumet back down in 2016 and remain low into 2020 (Figure S1c)


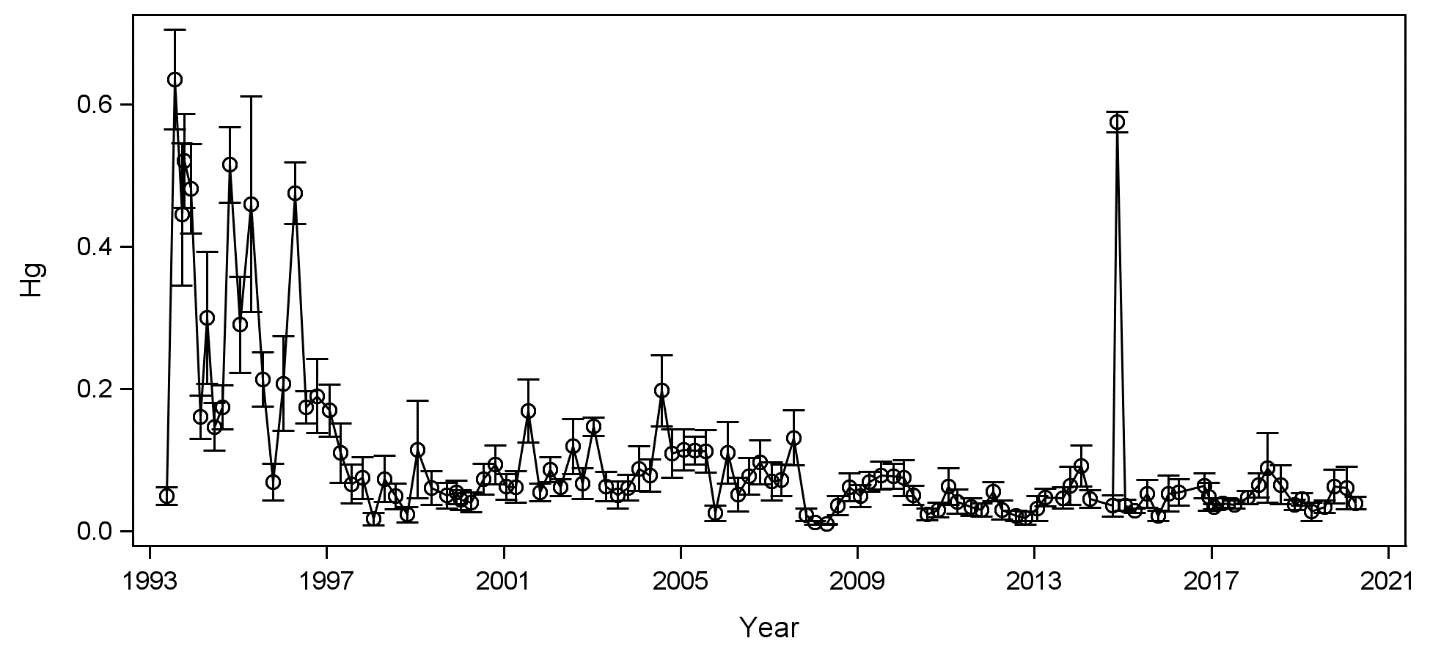


Figure S1c. Mercury (Hg) values in sediment overtime (mg/kg).

Lead concentrations begin at 3 mg/kg in 1993, increase to 30 mg/kg in 1995, decrease to 10 to 15 mg/kg from 1996 to 2003, increase to 25 mg/kg in 2002, and then remain between 5 to 10 mg/kg from 2003 till 2020 (Figure S1d


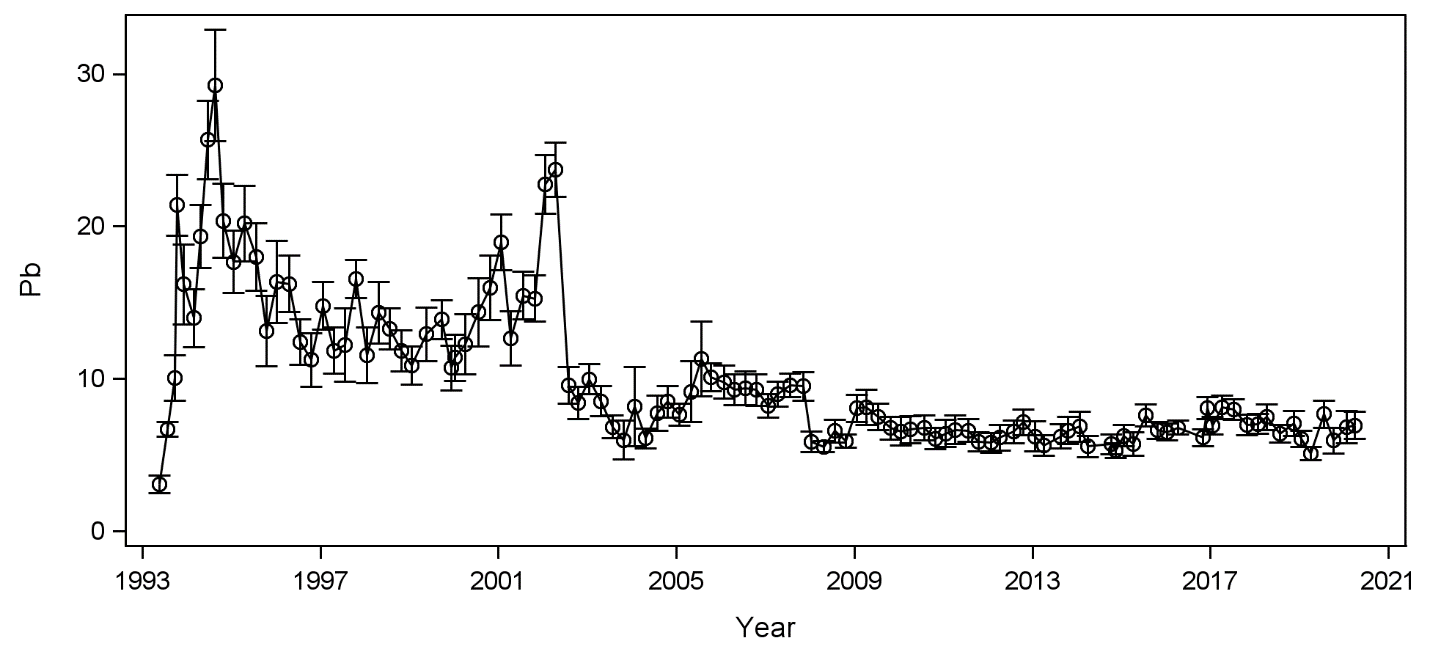


Figure S1d. Lead (Pb) values in sediment overtime (mg/kg).

Zinc concentrations show to be decreasing overtime with larger spikes in 1994, 1995, 2001, and 2020 (Figure S1e).


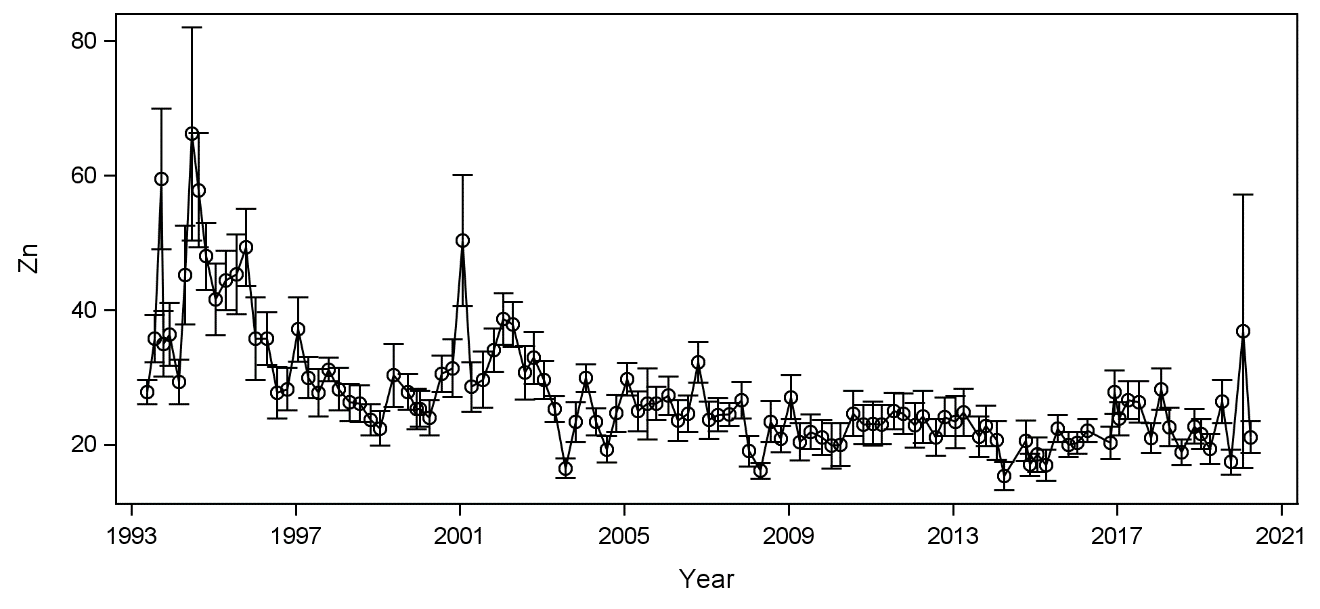


Figure S1e. Zinc (Zn) concentrations in sediment overtime (mg/kg).

Chemical oxygen demand (COD) fluctuates minimally from 1993 to 2015 with COD values ranging from 20 to 160 mg/L. COD values begin increasing significantly in 2016 till 2020 with a large spike in 2019 of ~530 mg/L (Figure S1f).


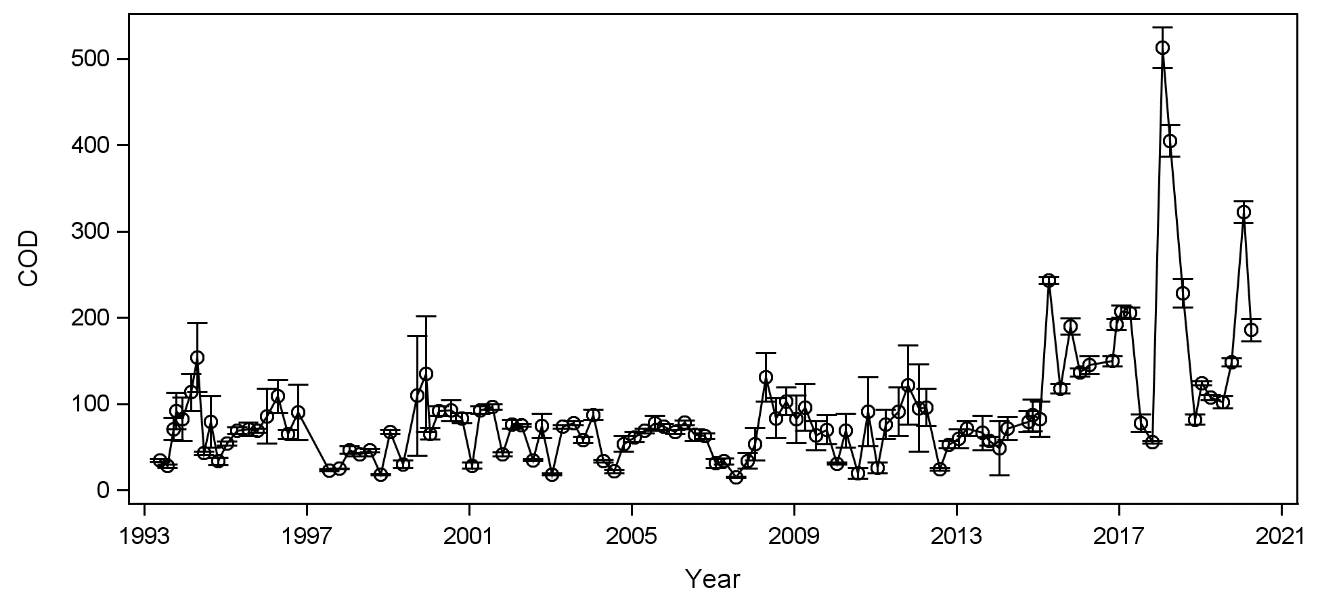


Figure S1f. Chemical oxygen demand (COD) values in water overtime (mg/L).

Dissolved oxygen (DO) values fluctuate yearly with 5.75 mg/L being the lowest DO value recorded and 11.90 mg/L as the highest DO value recorded (Figure S1g)


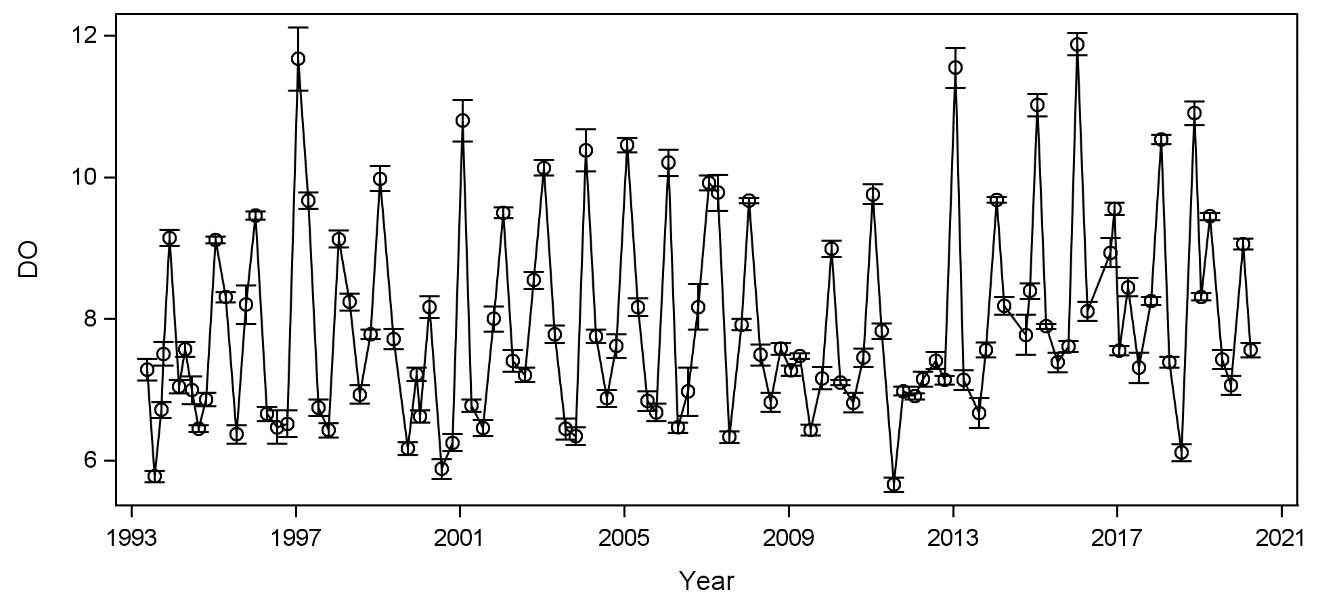


FigureS1g. Dissolved oxygen (DO) concentrations in water overtime (mg/L).

Dissolved Organic Nitrogen (DON) concentrations began at 0.5 mg-N/kg in 1993 then gradually increased to 3 mg-N/kg in 2002 (Figure S1h). DON concentrations were then <1 mg-N/kg in late 2002 till 2010. In late 2010, early 2011, DON concentrations rose to 1.7 mg-N/kg and fluctuated up and down till 2020. One spike is shown for DON concentrations in 2017 at 3.5 mg-N/kg.


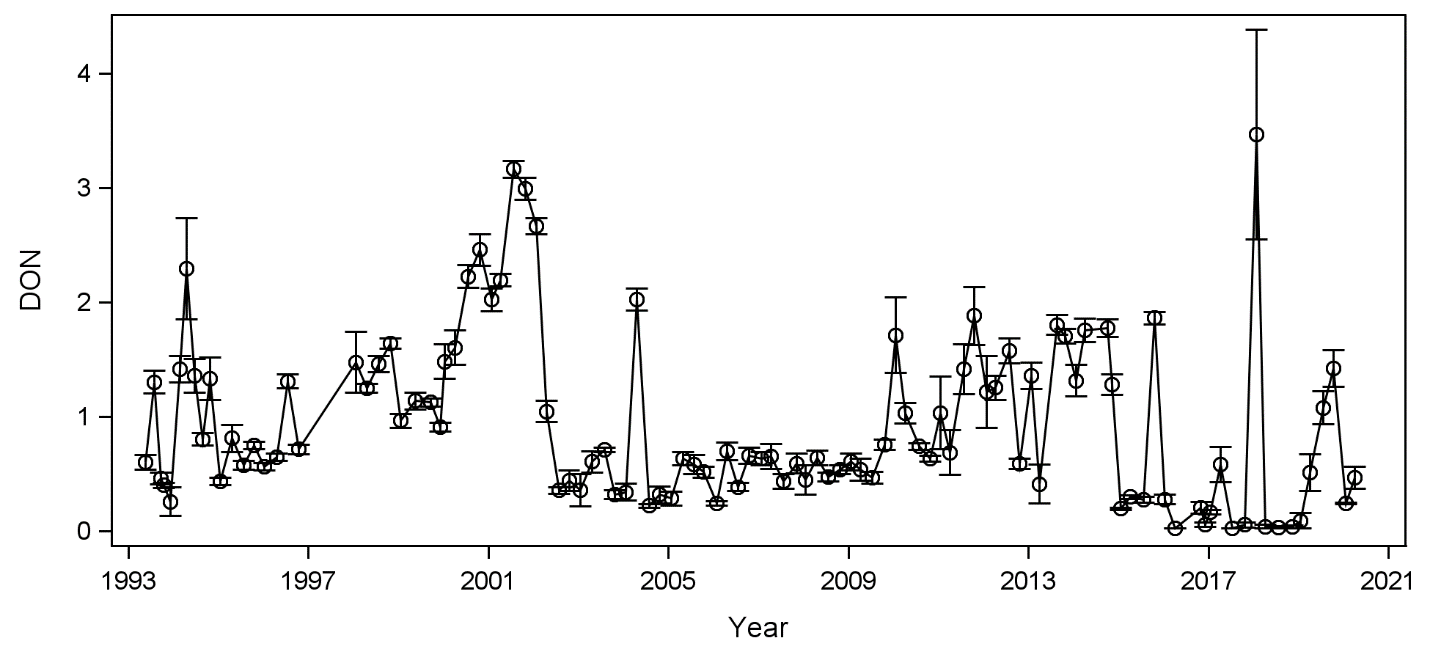


FigureS1h. Dissolved organic nitrogen (DON) values in water overtime (mg-N/kg).

Phosphate (PO4) concentrations fluctuated minimally overtime and remained at or below 0.2 mg/L but did present three noticeable spikes in 2001 (0.3 mg/L), late 2002 (0.4 mg/L), and late 2008 (1.3 mg/L) (Figure S1i).


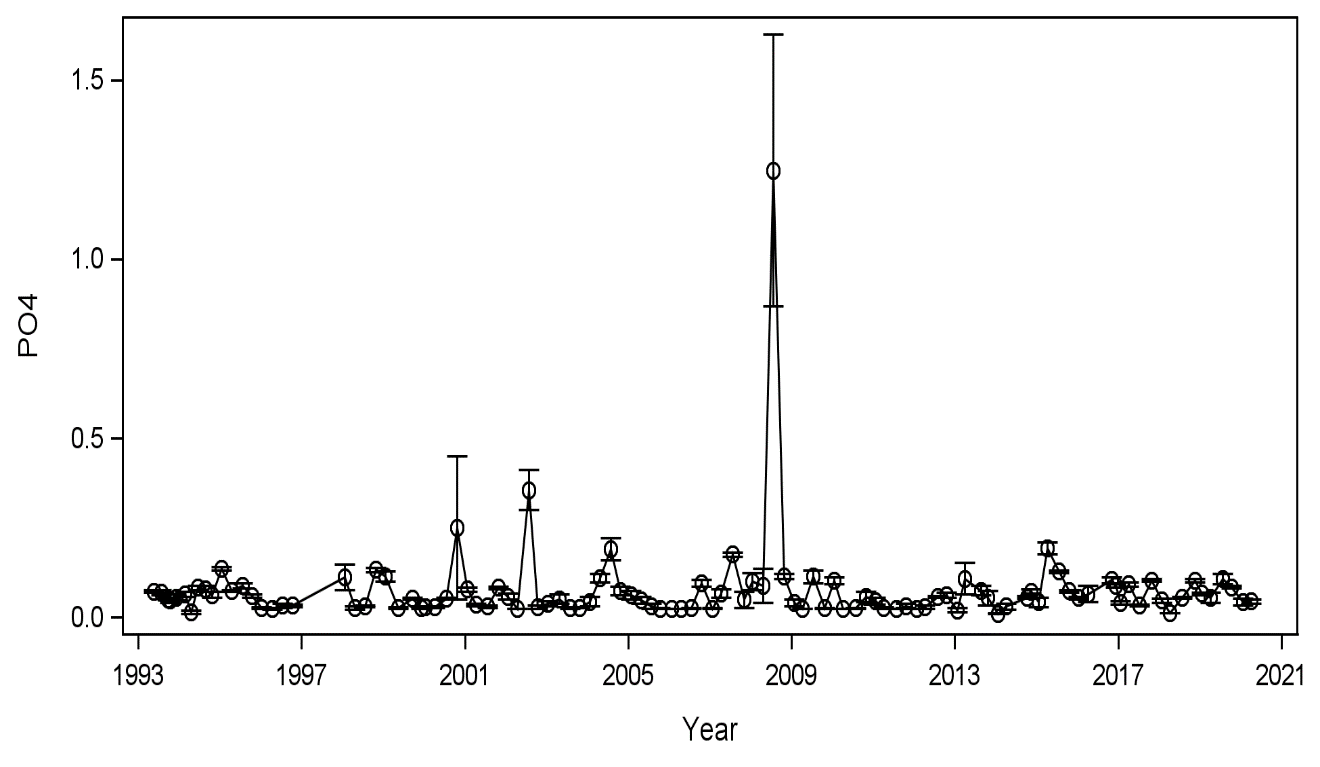


Figure S1i. Orthophosphate (PO4) values in water overtime (mg P/L).

Units of pH fluctuated between 7.5 and 8.5 as expected over the course of data collection from 1993 to 2020 (Figure S1j). Spikes were seen in pH in years 1995 (7.25 S. U.), 2010 (7.1 S.U.), late 2012 and 2013 (8.7 S.U.), and 2016 (9.3 S.U.).


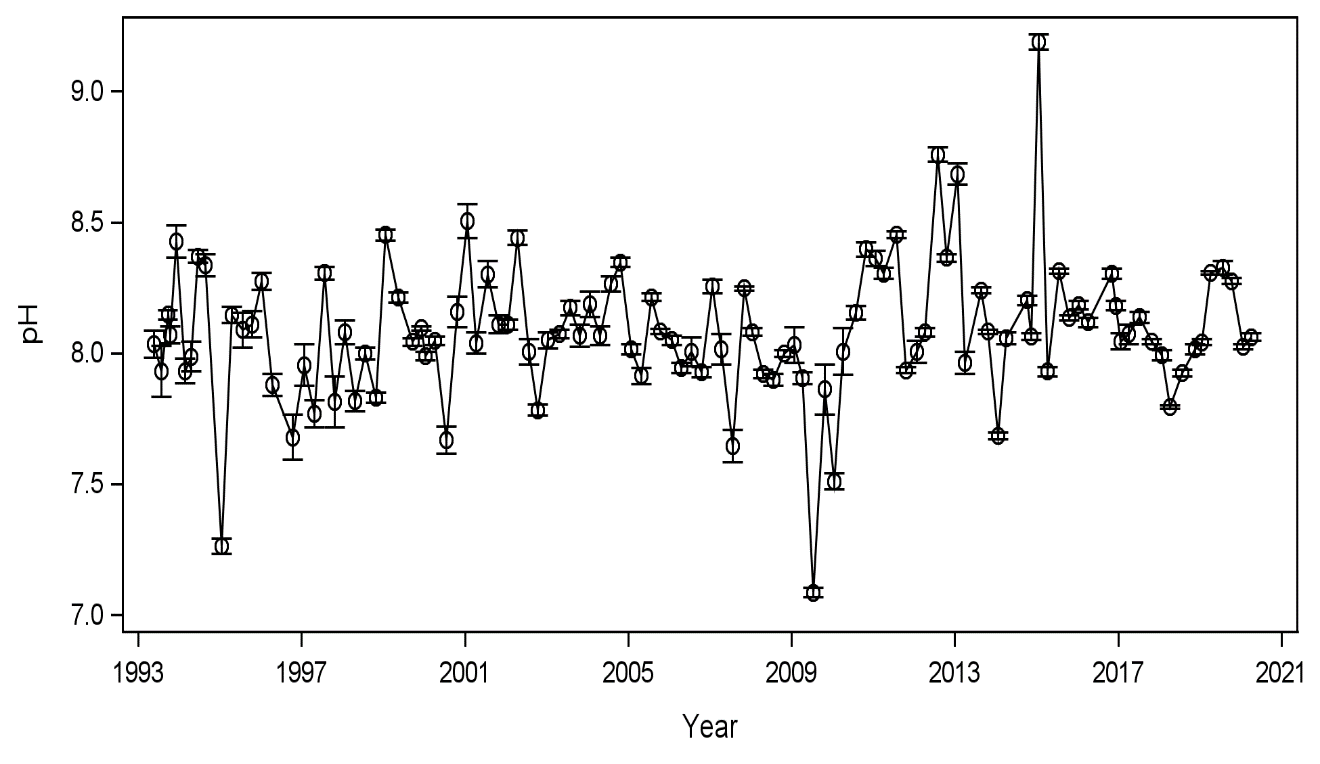


Figure S1j. pH values in water overtime (S.U.).

Sulfate (SO4) concentrations fluctuated from 0 to approximately 2400 mg/L from 1993 to early 2008. Beginning in late 2008 concentrations began increasing to a maximum of 3,300 and then began decreasing once more in 2014 (Figure S1k).


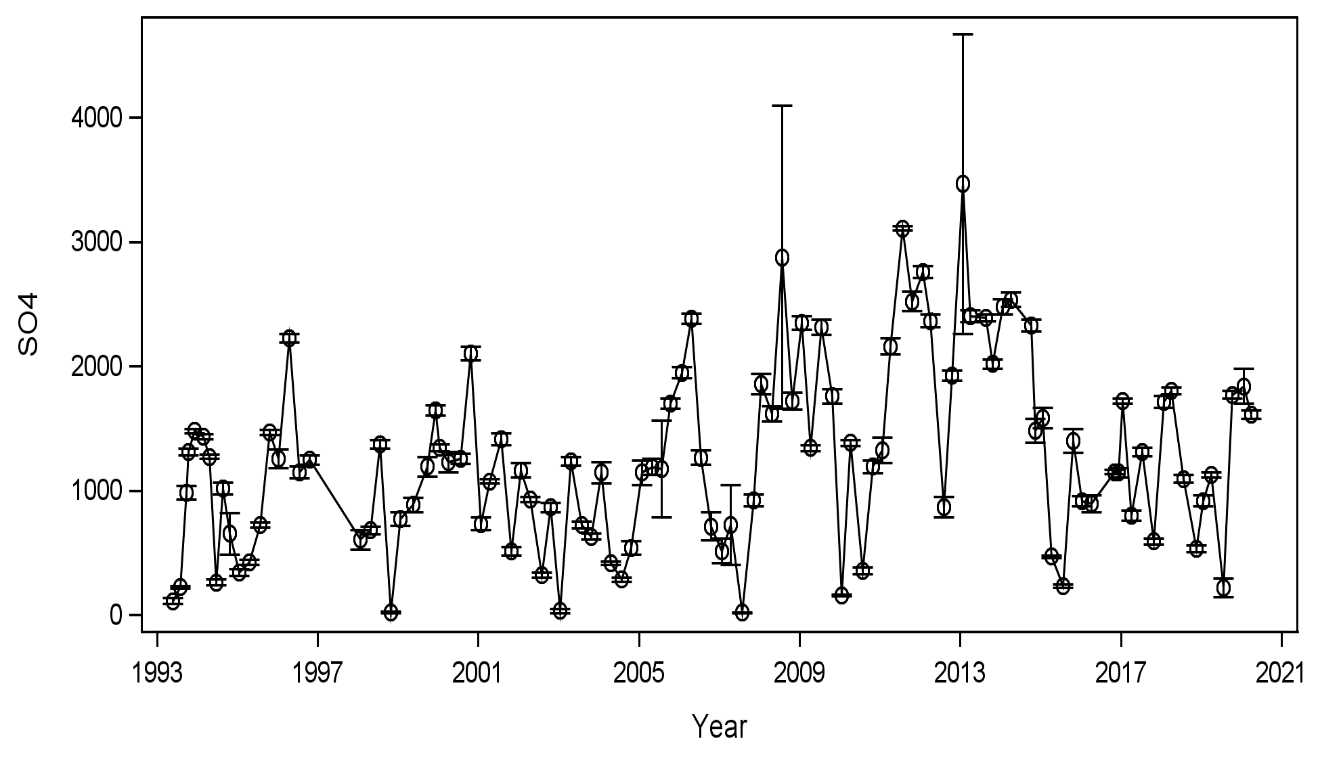


Figure S1k. Sulphate (SO4) concentrations in water overtime (mg S/L).

Salinity fluctuated seasonally each year (Figure S1l). The lowest salinity value recorded was in 1999, 2008, and 2010 with a salinity of 0 PSU.


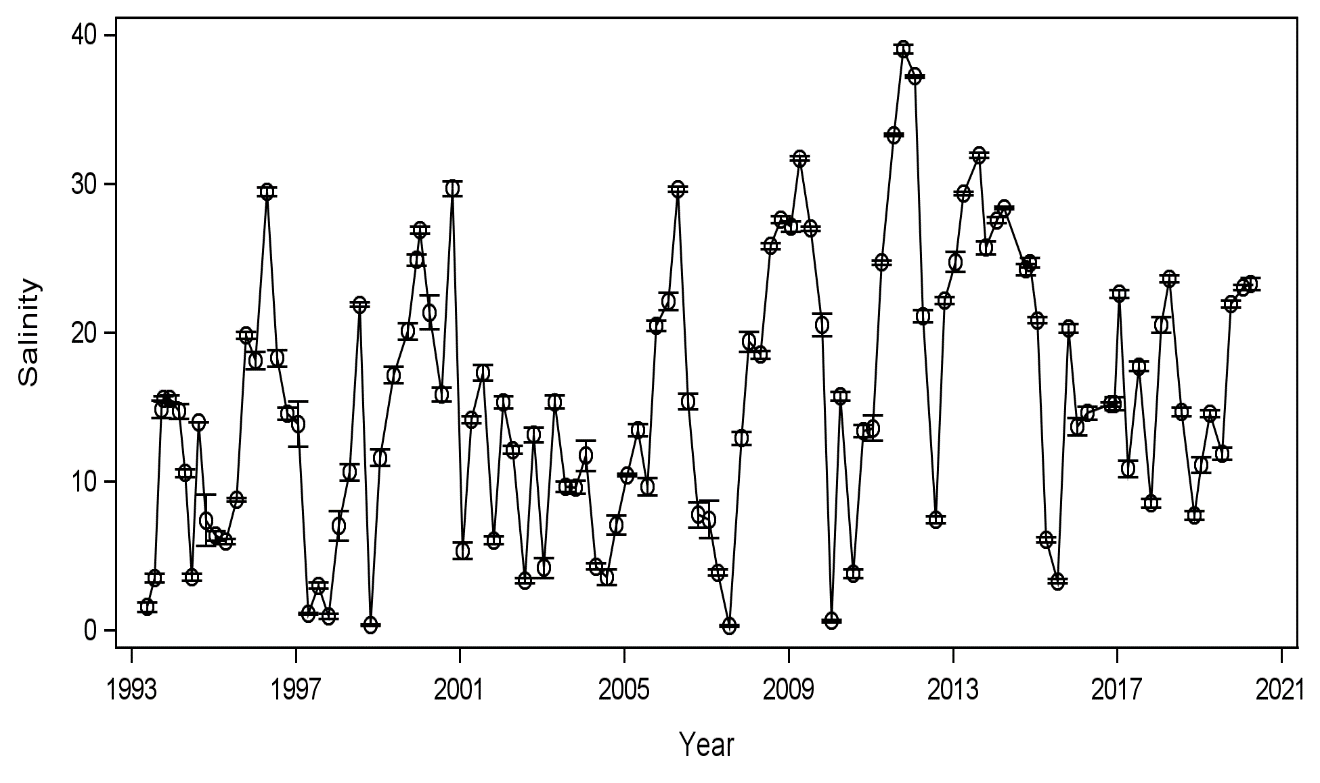


Figure S1l. Salinity values in water overtime (PSU).

Temperature fluctuated throughout each year (Figure S1m). The highest temperature recorded was 33 degrees Celsius and the lowest temperature was 5 degrees Celsius.


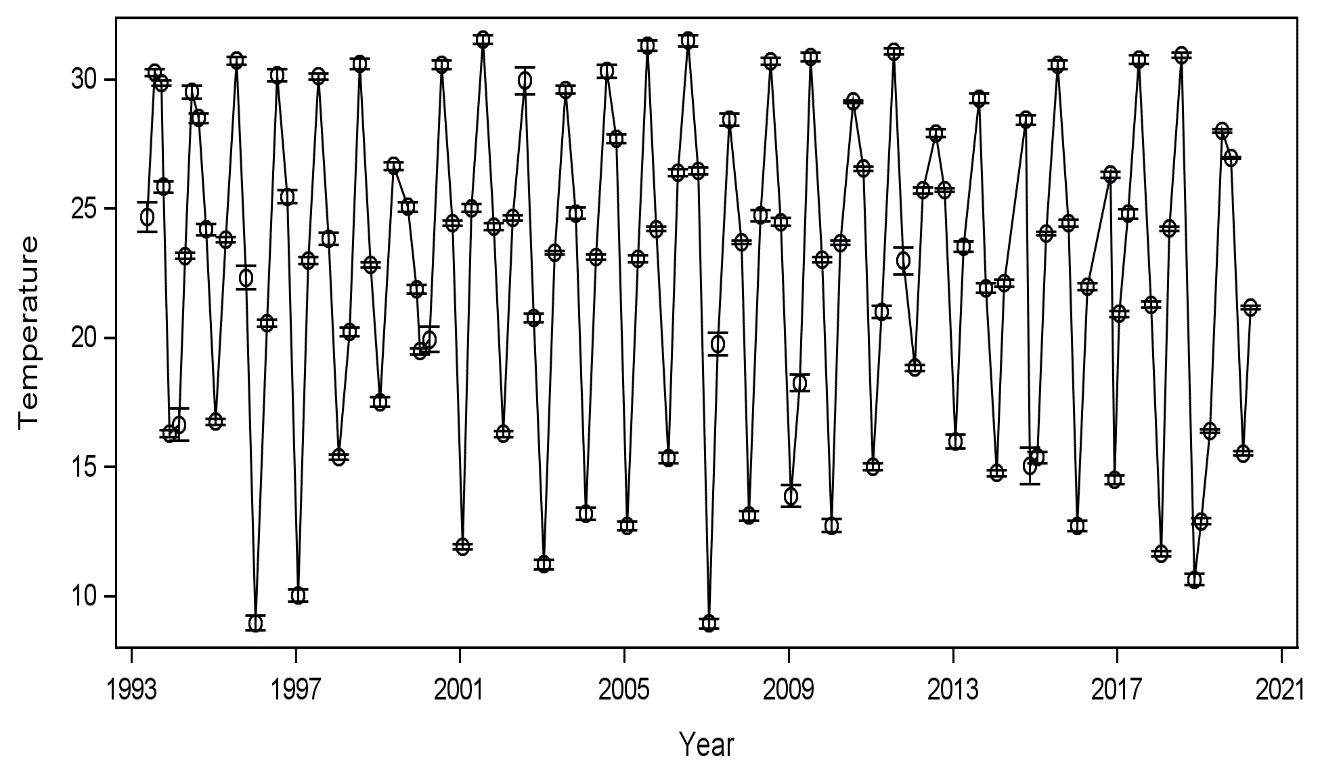


FigureS1m. Temperature values in water overtime (C°).

Turbidity values remain low (less than or equal to 100 NTU) over the course of time data was collected; however, turbidity increased in late 1995 (240 NTU), 2003 (120 NTU), late 2004 (180 NTU), and 2010 (210 NTU) (Figure S1n).


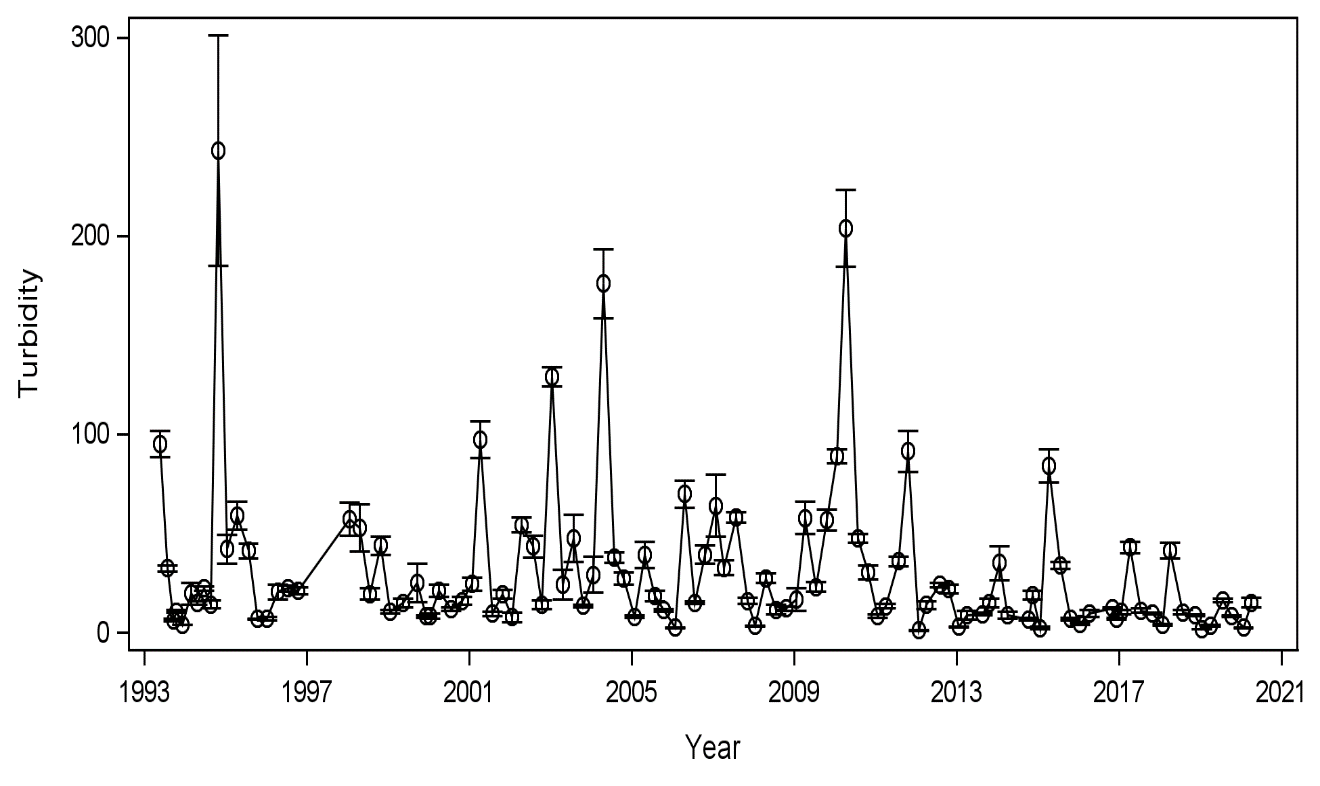


Figure S1n. Turbidity values in water overtime (NTU).

Total Organic Carbon (TOC) concentrations show three primary events throughout the span of data collection (S1o). TOC gradually increases from 3 mg/L to 45 mg/L from 1993 to 2002. After 2002 TOC concentrations begin to decrease to 10 mg/L or less and remains at a lower concentration until 2019 with a large spike at 35 mg/L. After 2019 TOC concentration lowers back down to 6 mg/L.


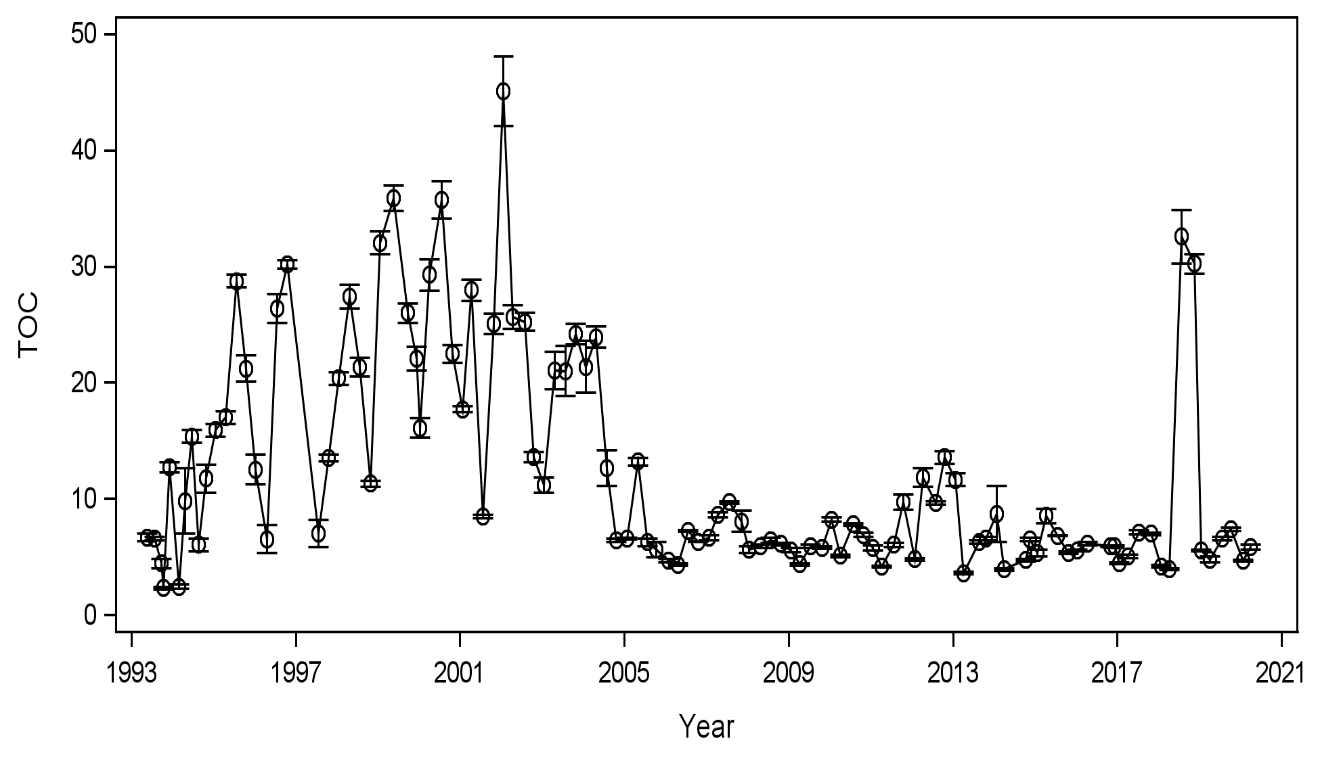


Figure S1o. Total organic carbon (TOC) values in water overtime (mg/L).

Sand increased over time while silt and clay content decreased (Figure S1p). After 1997, sand content began to rise from a lower percentage of 40% to 60% and silt and clay content lowered from a percentage in the 40’s to less than 10% by 2020.


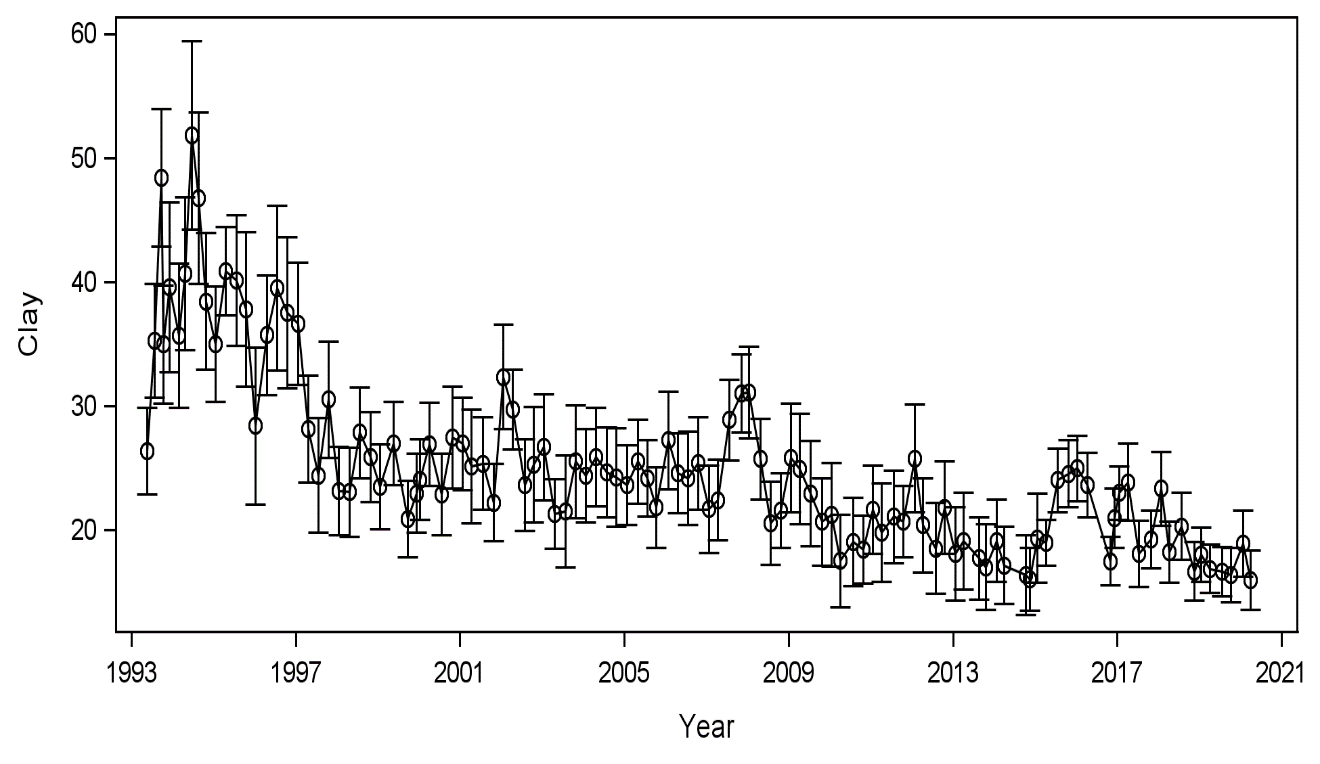

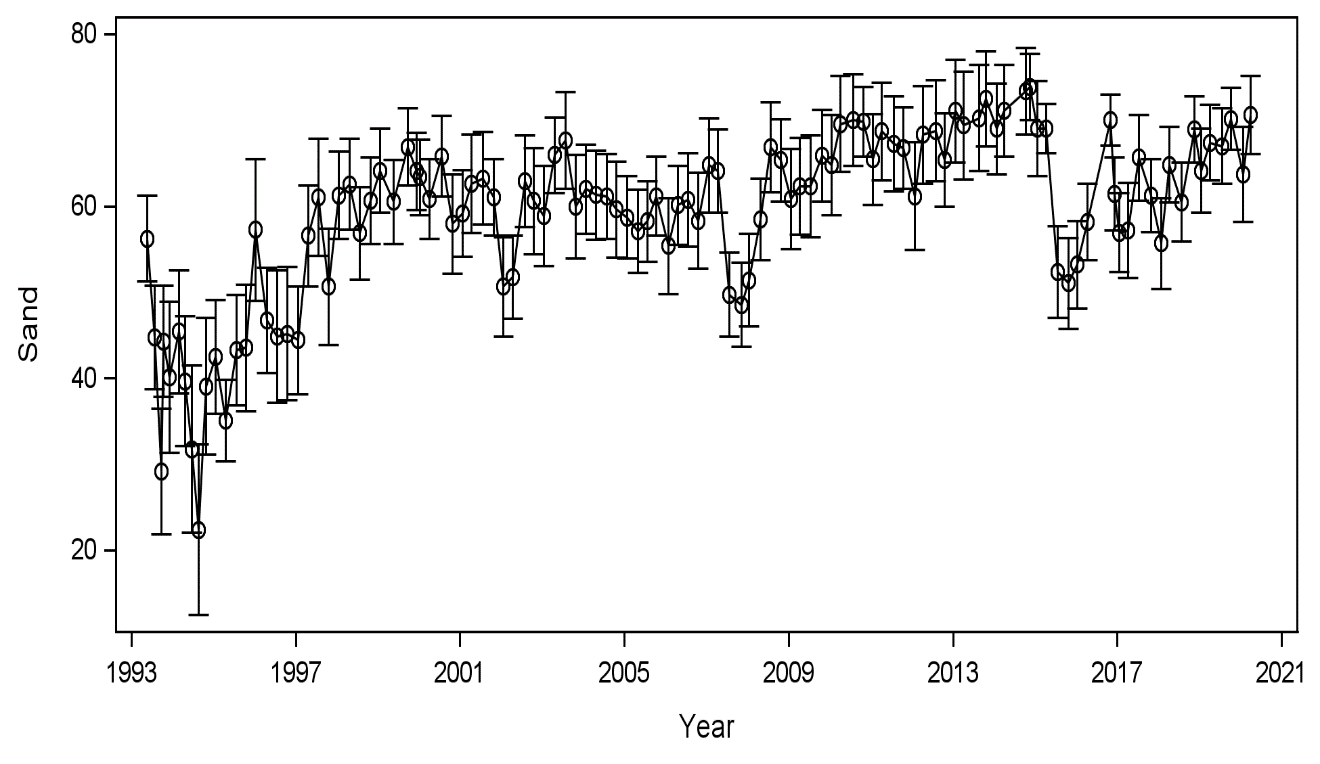

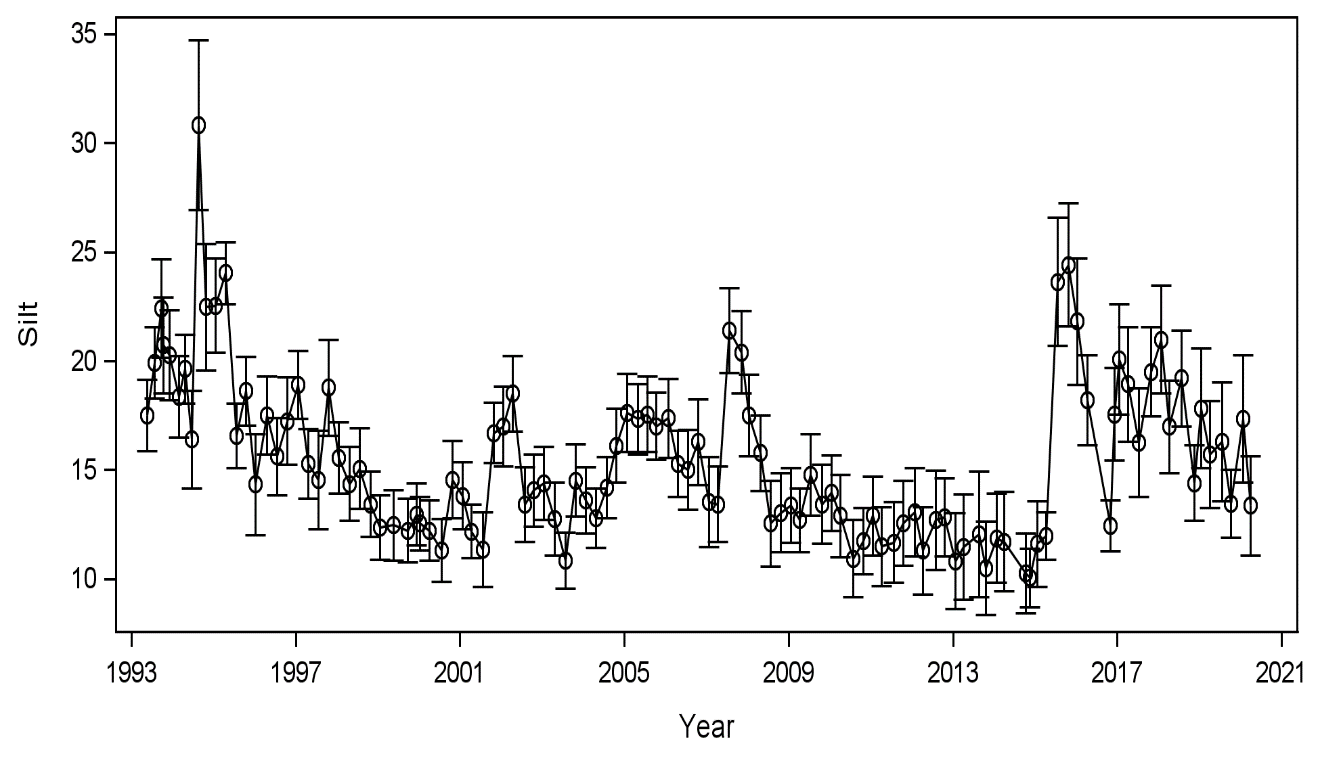


Figure S1p. Sand, silt, and clay sediment content overtime (%).

Benthic abundance spanned three orders of magnitude (Figure S1q). Abundance begins high in the thousands from 1993 to 2001, drops to single digits in 2002, fluctuates up and down between 2005 and 2010, drops once more in 2011 and then begins leveling back out in 2012. Benthic richness spans from 1 to 23 species per sample and is high when abundance is high and low when abundance is low.


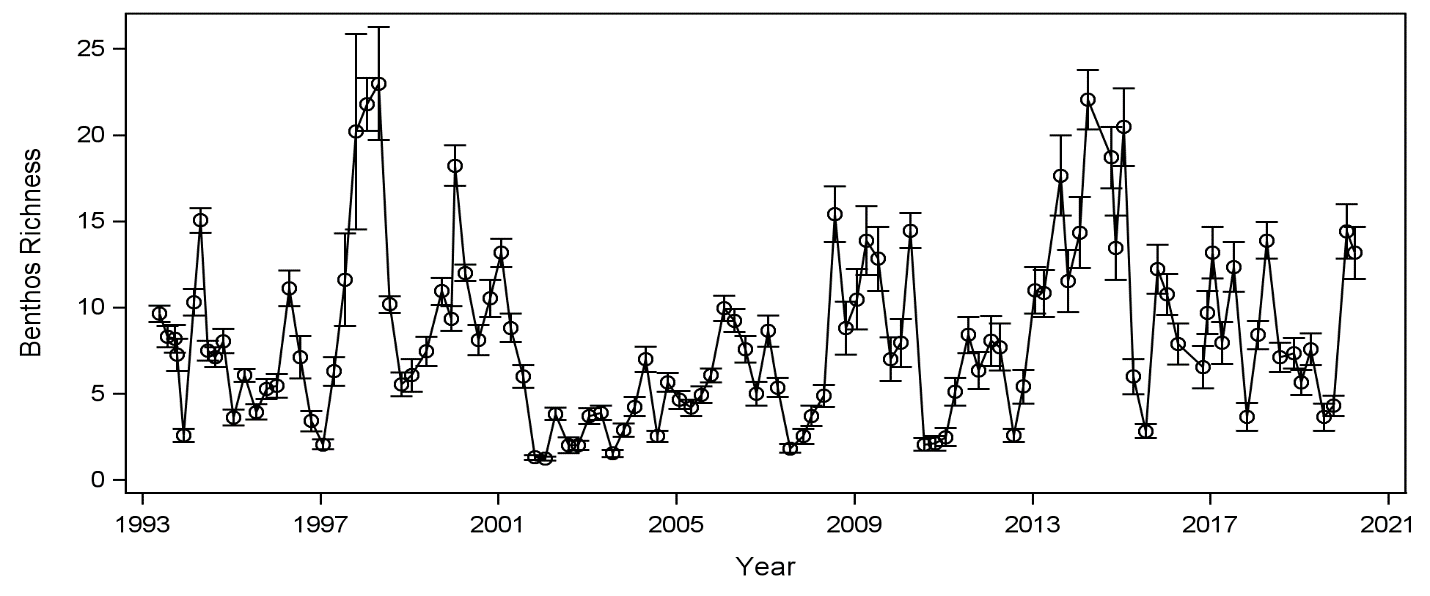

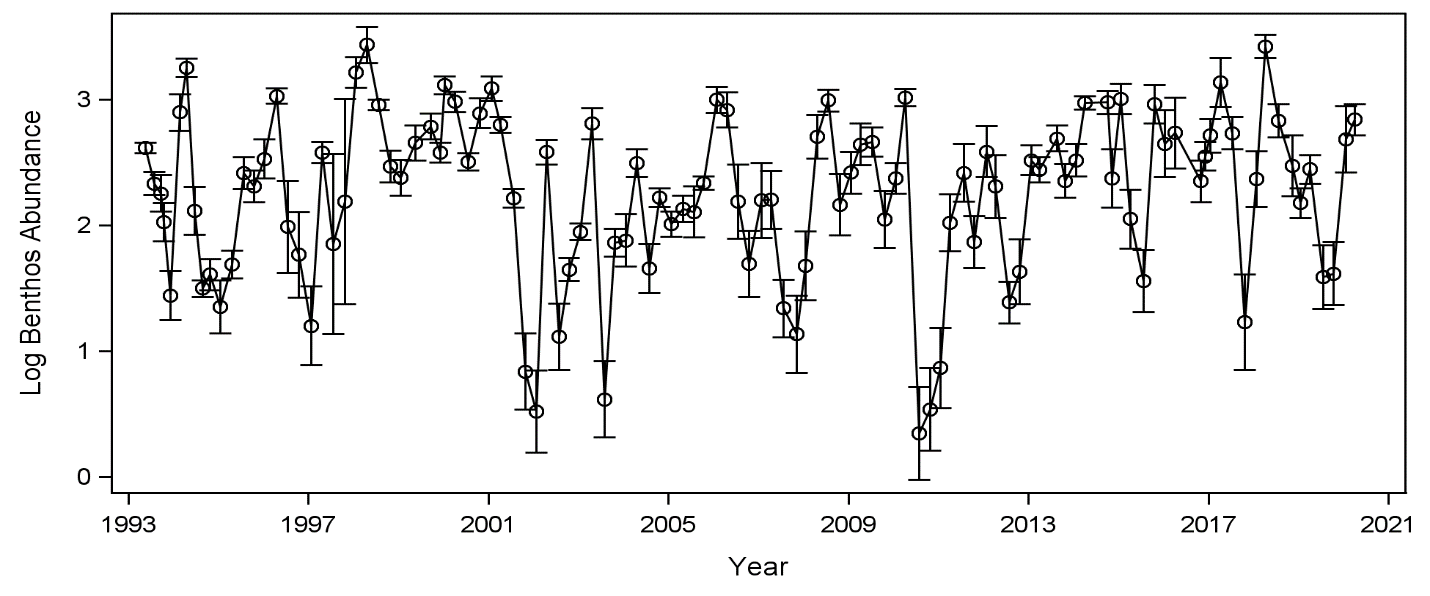


Figure S1q. Benthic abundance (log_10_ n/m^2^) and richness (S/0.023 m^2^ sample) from 1993 to 2020.

Phytoplankton abundance is near zero from 1993 till 2009 (Figure S1rA). After 2009, counts range from 6,000,000 n/m2 to close to zero. Phytoplankton abundance time series was log_10_ transformed (Figure S1rB). Counts spanned seven orders of magnitude. There appears to be three abundance patterns: phytoplankton from lower abundances (<1000/m^3^) in 1993 to 1999, moderate abundances (1000/m^3^ to 10,000/m^3^) from 2000 to 2008, and highest abundances (100,000/m^3^ to 1,000,000/m^3^) from 2009 to 2020. Phytoplankton richness begins low with values under 40 species/ 2 L from 1993 to 1997 and then rises to 85 in 1999 then gradually decreases until 2006 and increases from then to 2012 (Figure S1rC).


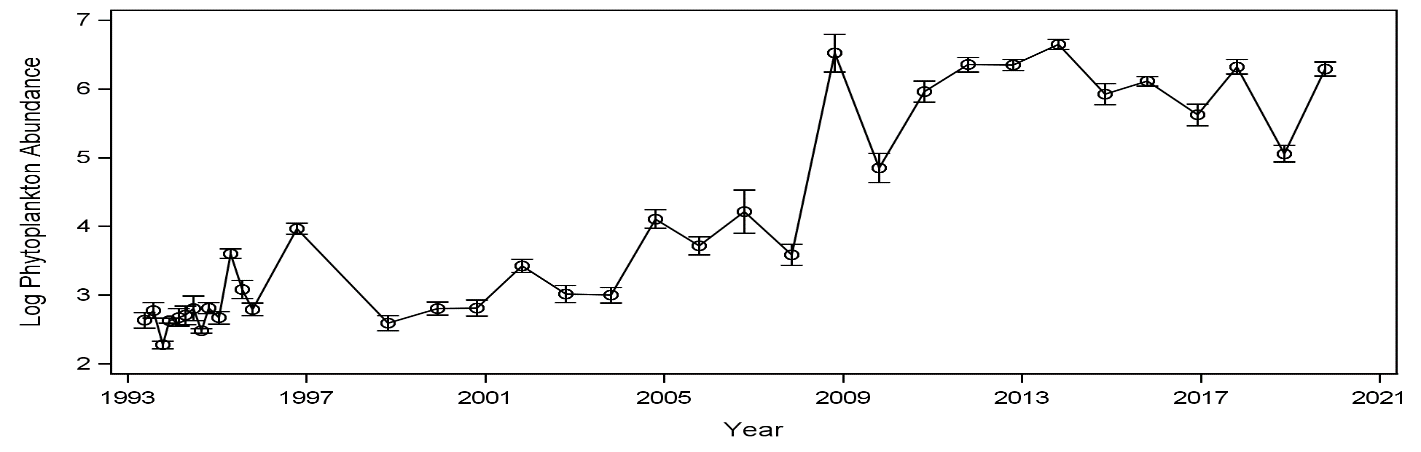

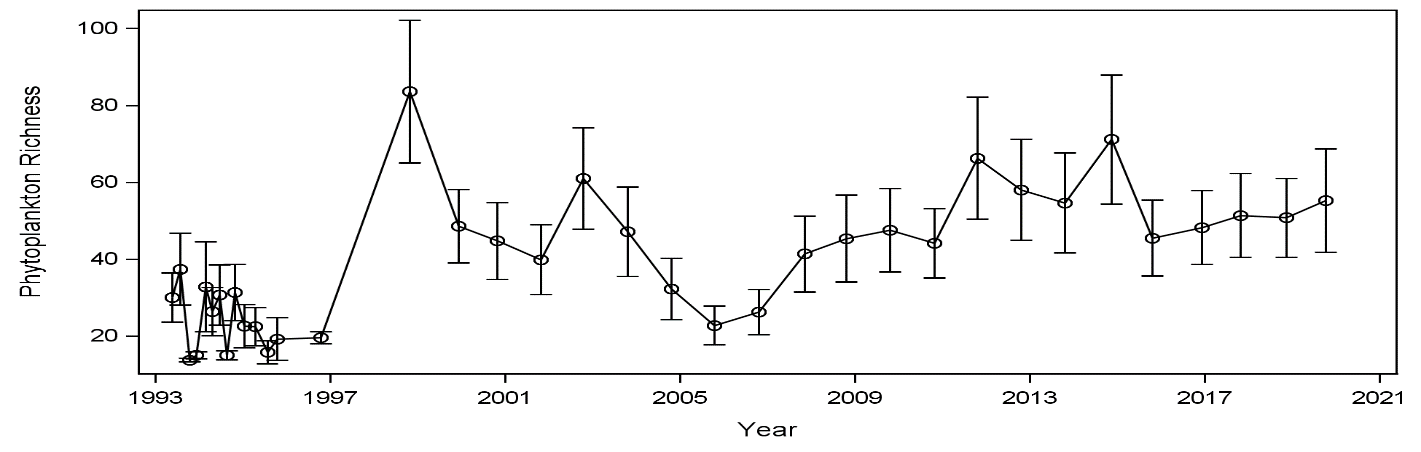


FigureS1r. Phytoplankton abundance (n/m^3^), log abundance (log_10_ n/m^3^) and richness (S/2 L sample) over time.


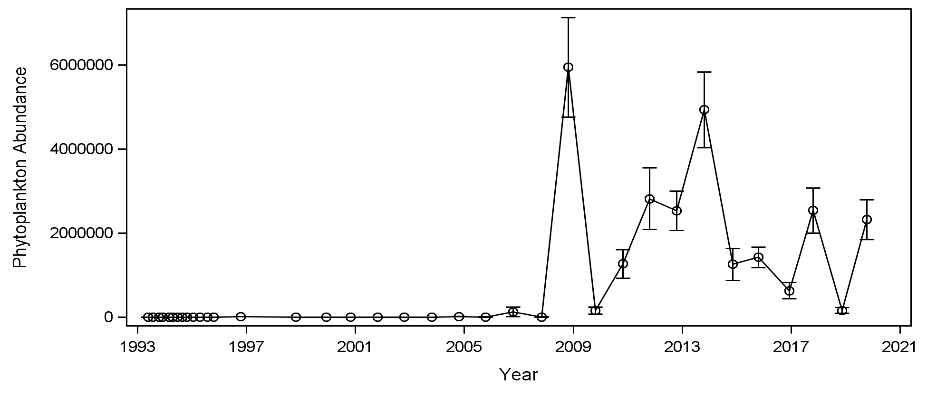


**A**

**A**

**A**

**A**

**B**

**B**

**B**

**B**

**C**

Figure 3. Zooplankton abundance (log_10_ n/m^3^) and richness (S/48 L sample) overtime.**C**

Figure S1s. Zooplankton abundance (log_10_ n/m^3^) and richness (S/48 L sample) overtime.**C**

Figure 4. Zooplankton abundance (log_10_ n/m^3^) and richness (S/48 L sample) overtime.**C**

Zooplankton abundance is near zero from 1993 to 2009 (Figure S1sA). After 2009, counts range from 100,000,000 n/m^3^ to approximately 20,000,000 n/m^3^. Zooplankton abundance spanned eight orders of magnitude (Figure S1sB). There appears to be three abundances between 1993 to 1995, then from 1996 to 2009, and then 2010 to 2020. For richness, the initial period from 1993 to 1995 have lower values under 10 species and then in 1996 and onward values look to be consistent at a higher value of 15 species or more (Figure S1sC).


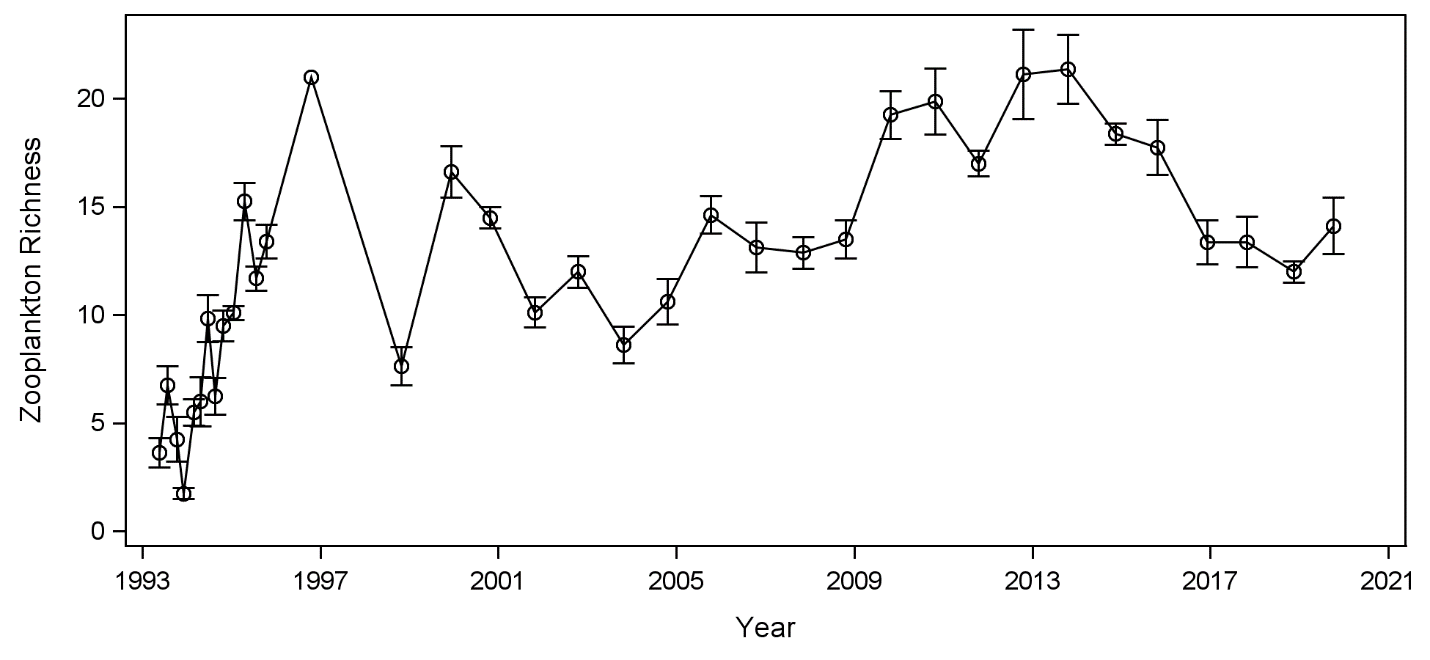

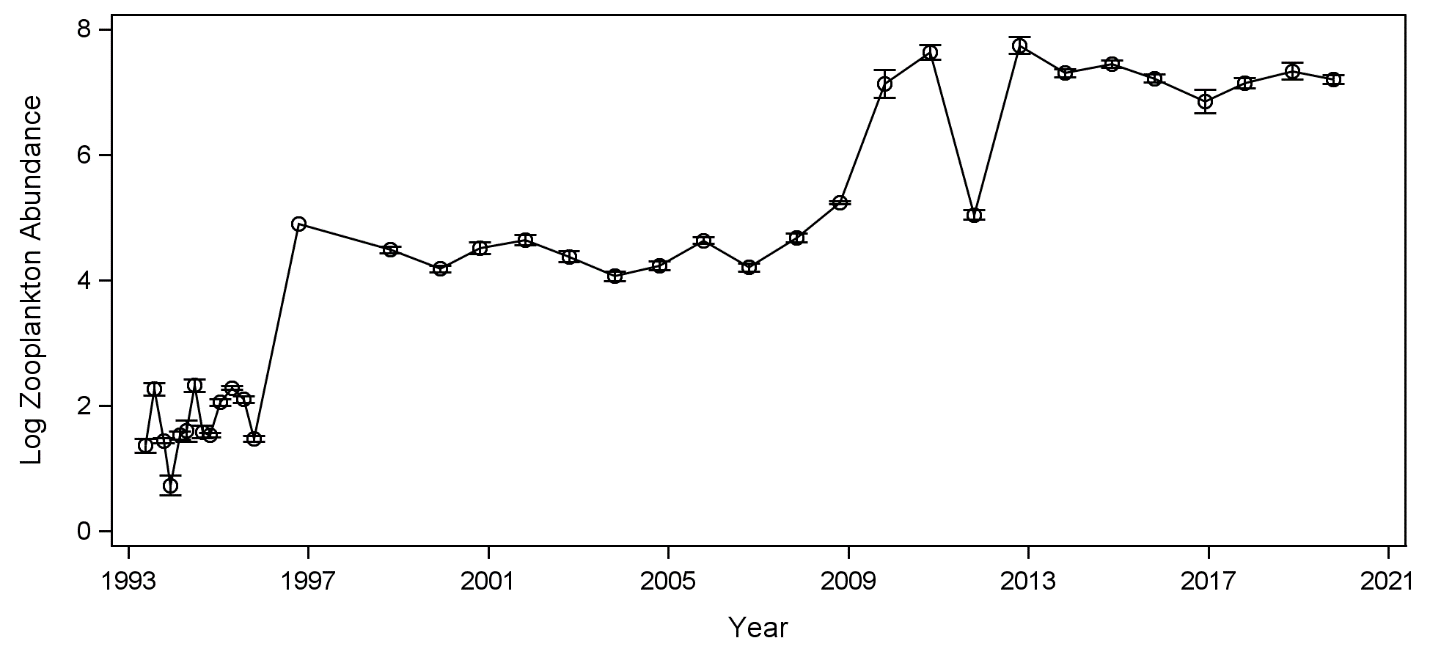


Figure S1s. Zooplankton abundance (log_10_ n/m^3^) and richness (S/48 L sample) overtime.


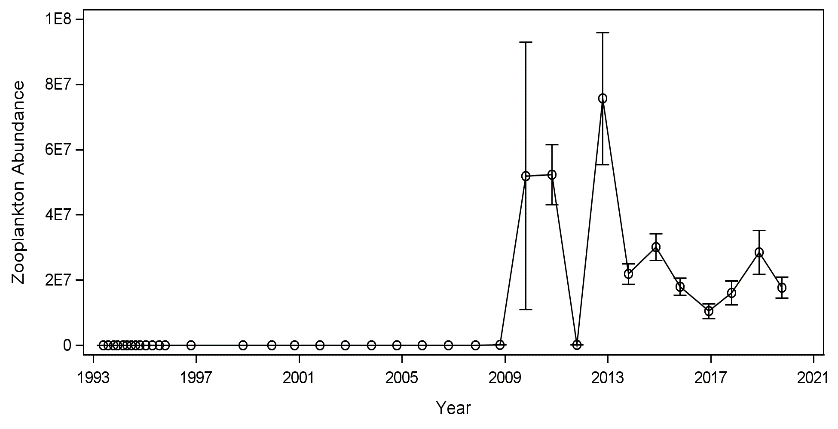


**A**

**A**

**A**

**A**

**B**

**B**

**B**

**B**

**C**

Figure 5. Gillnet abundance (log_10_ n/24 h set) and richness (S/sample) overtime.**C**

Gillnet abundance spans five orders of magnitude (Figure S1t). Abundance values appear to be most often about 1,000 but then decrease for years 1994, 1996 to 1997, 2000 to 2002, 2007 to 2015, 2018, and 2020. Gillnet richness fluctuates with 15 species as the highest species count and one as the lowest.


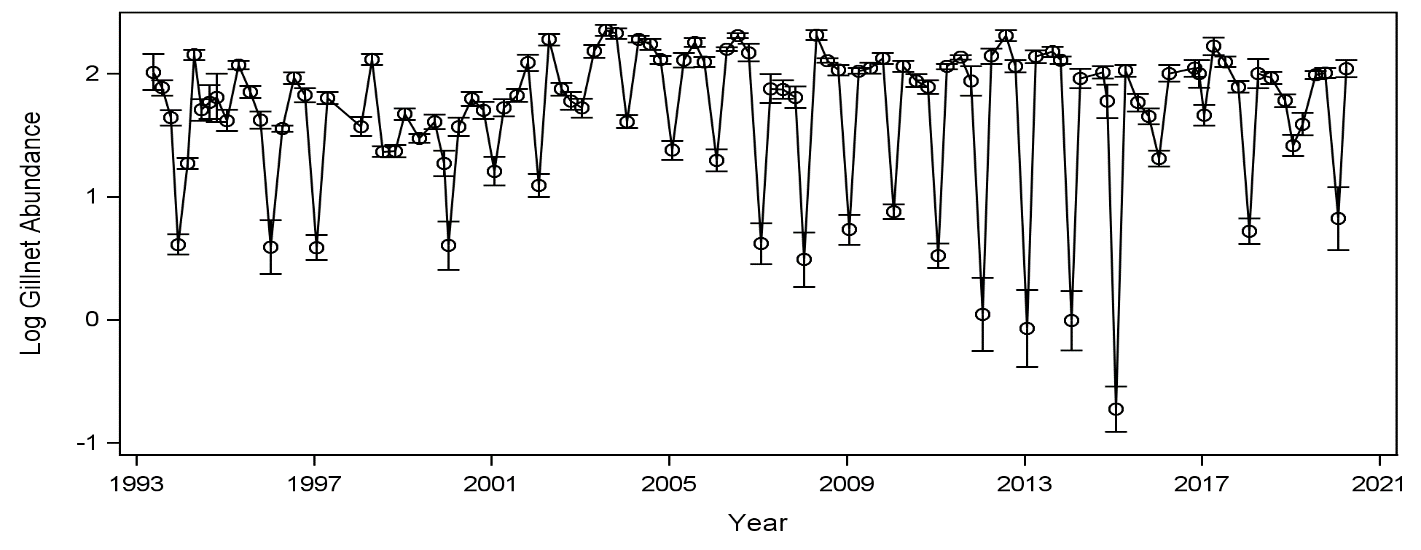

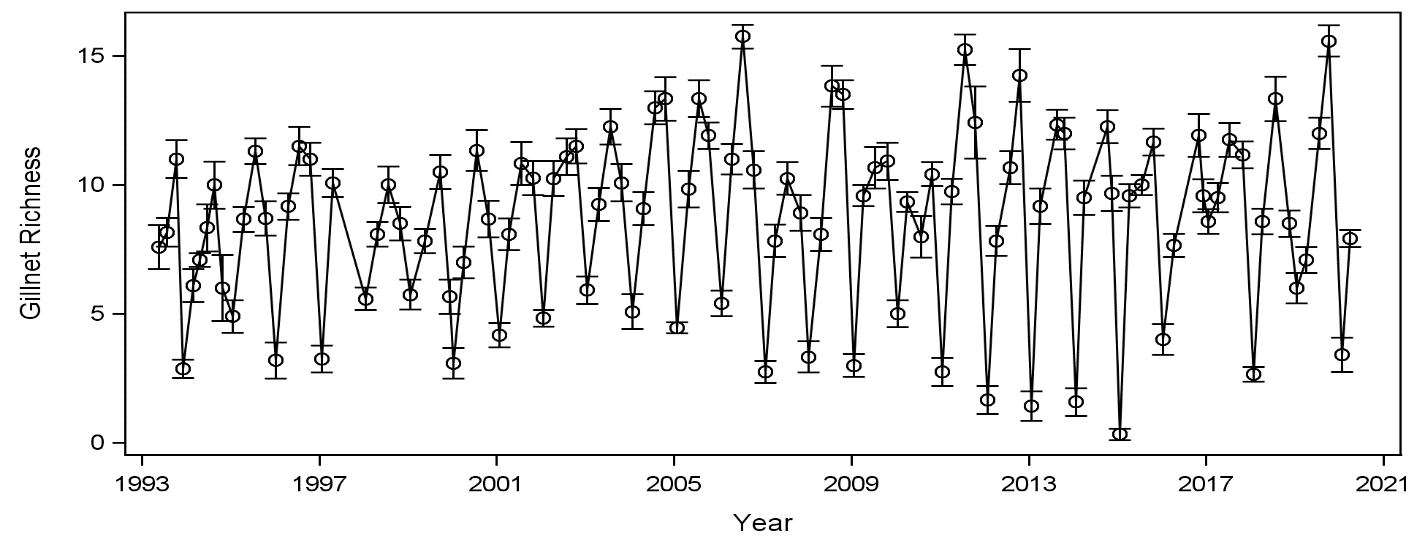


Figure S1t. Gillnet abundance (log_10_ n/24 h set) and richness (S/sample) overtime.

Ichthyoplankton abundance spans 5 orders of magnitude (Figure S1w). Ichthyoplankton richness fluctuates with the highest ichthyoplankton richness in years 2002, 2004, 2006, 2012, and 2020 with 6 to 8 species per sample.


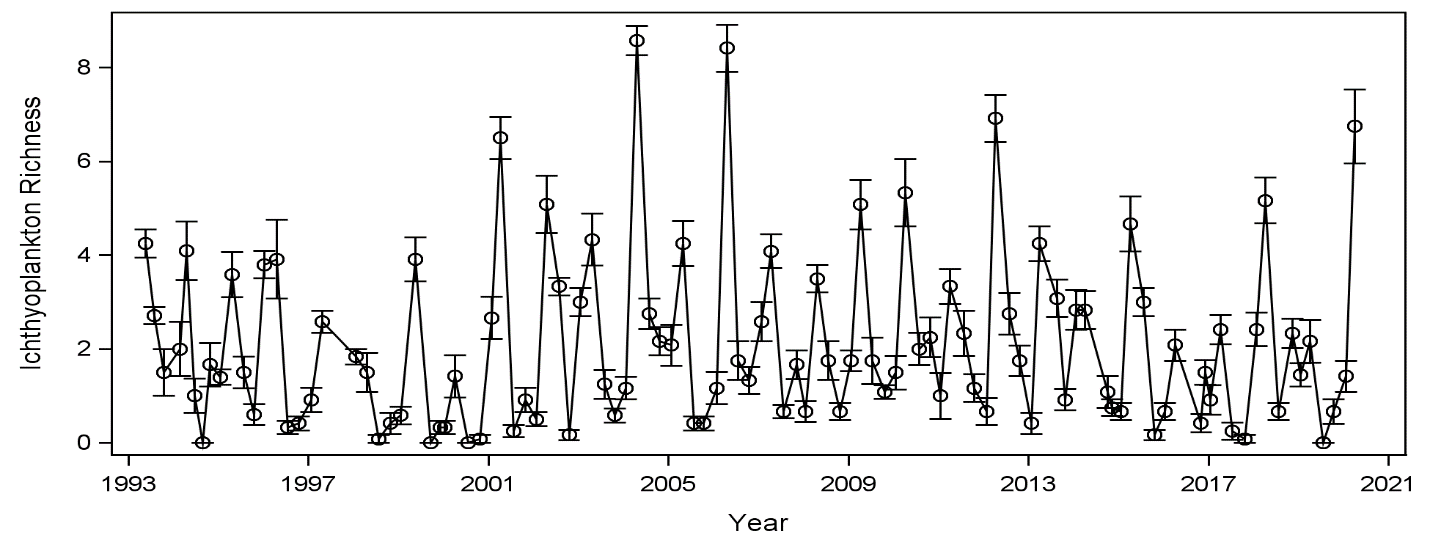

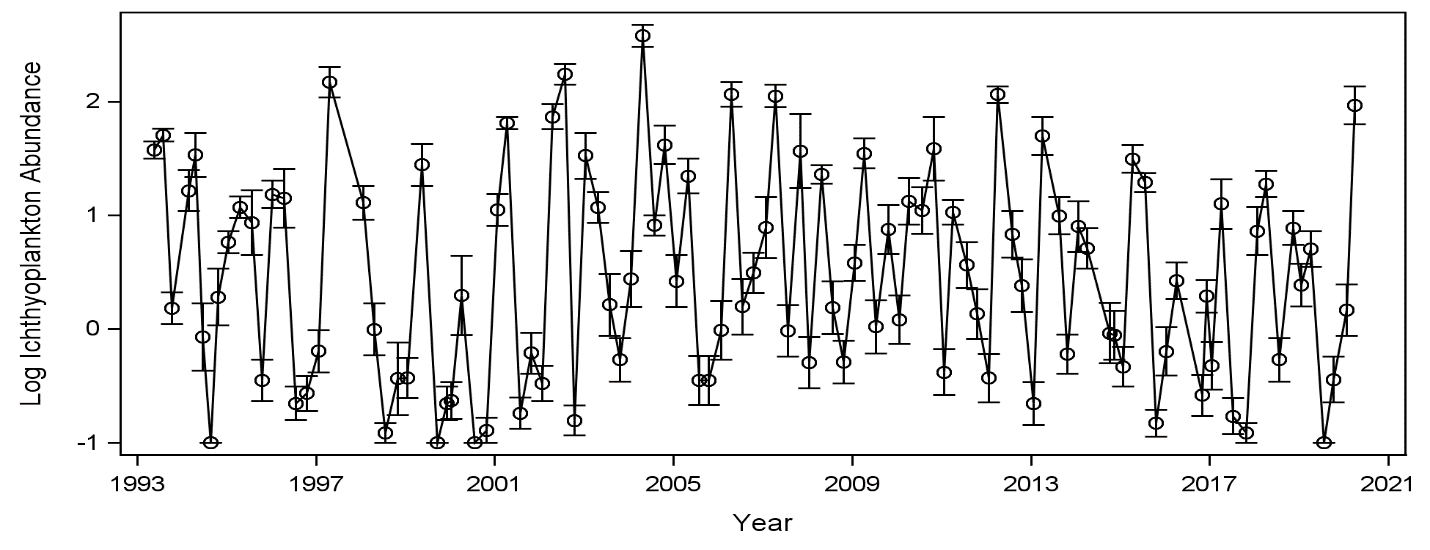


Figure S1w. Ichthyoplankton abundance (log_10_ n/5 min tow) and richness (S/sample) overtime.

Trawl abundance spans five orders of magnitude and gradually decreases overtime (Figure S1x). Abundance was lowest in 2015. Richness in 1993 shows 12 species counted and then gradually decreases to one species accounted for in 2015.


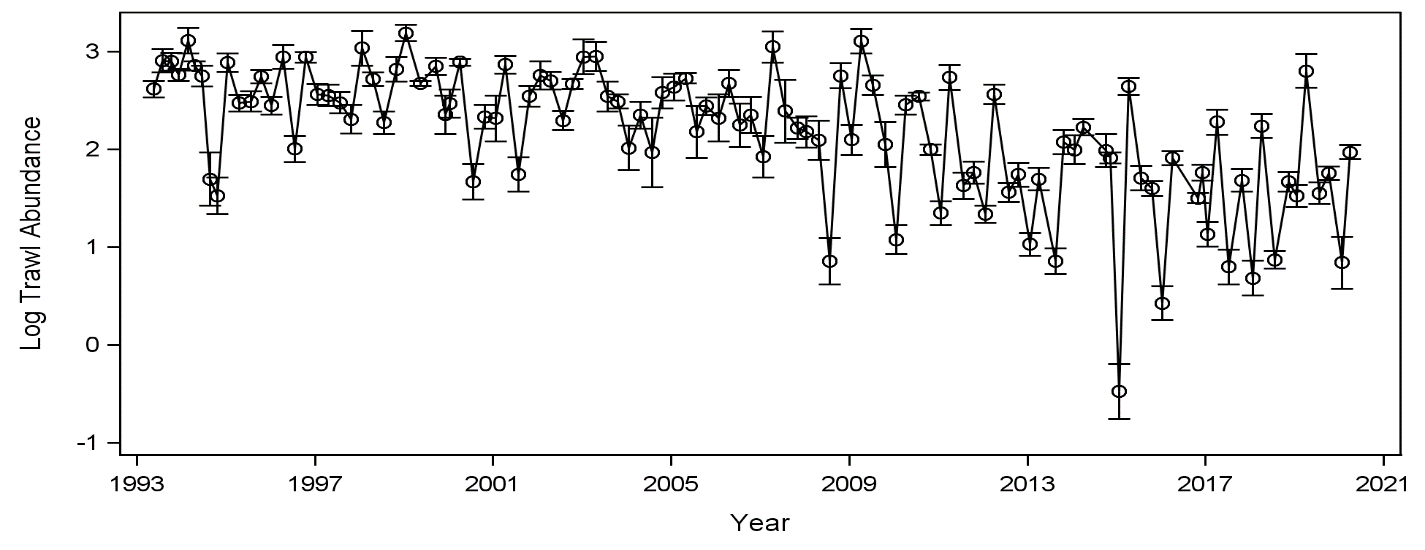

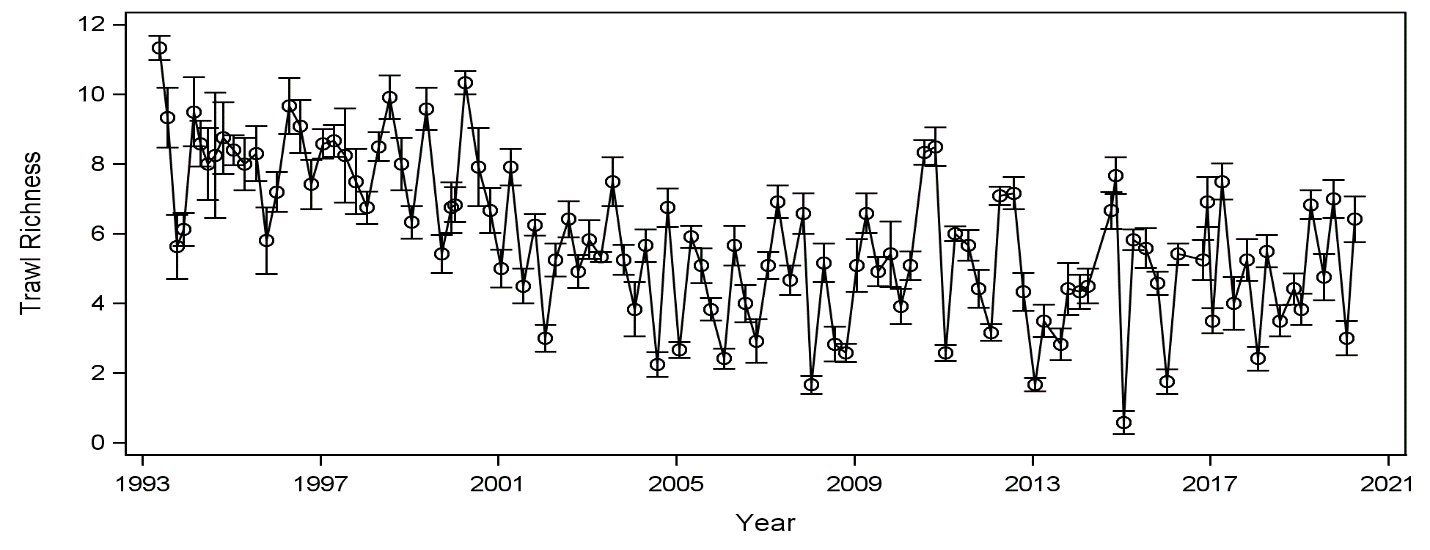


Figure S1x. Trawl abundance (log_10_ n/5 min tow) and richness (S/sample) overtime.

The water column PC-trip scores were plotted over time from 1993 till the end of 2020 (Figure S1yA). Wet periods are negative PC1 scores, and average and dry periods are positive. PC2 plots seasonality overtime (Figure S1yB). Summer 1994 is extremely positive PC2 correlating to a dry climatic period.


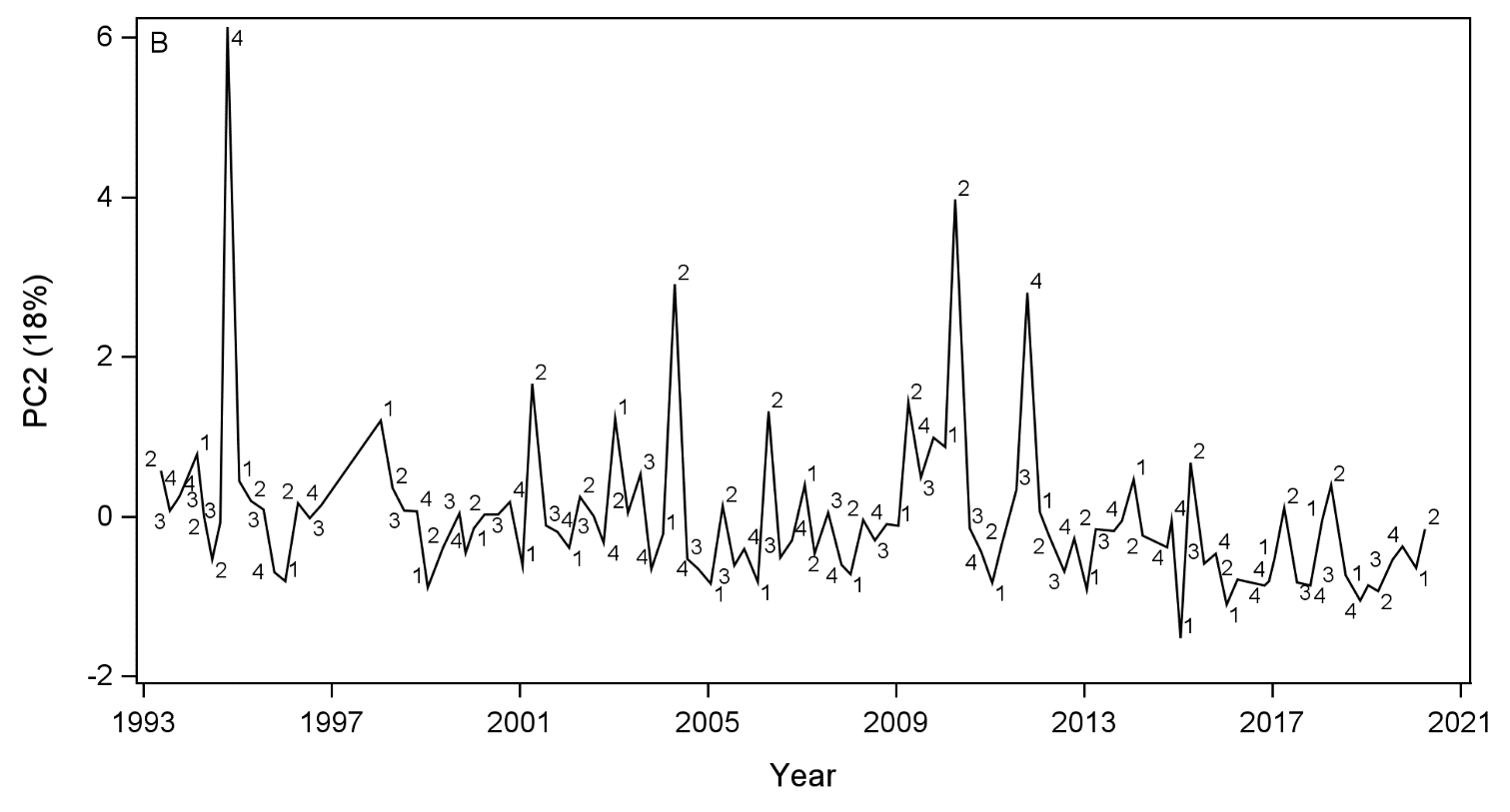

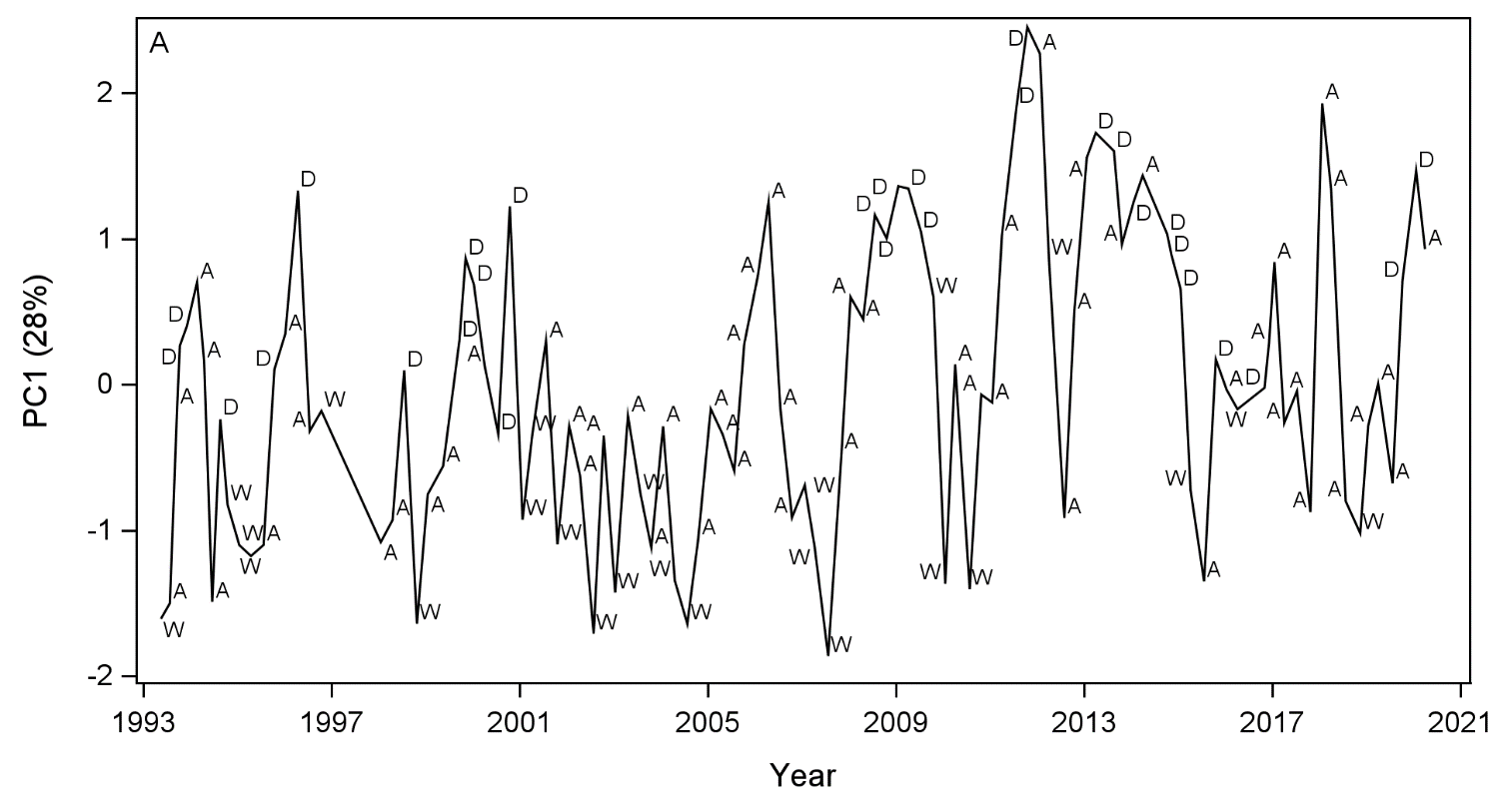


Figure S1y. Water quality PC1 sample scores for sampling dates. Symbols represent climatic periods, where W = wet, A = average, D = dry. PC2 sample scores for sampling dates where symbols represent seasons, 1 = winter, 2 = spring, 3= s summer, 4 = fall.

The PC 1 sediment sample scores were plotted overtime from 1993 through 2020 (Figure S1z). Positive PC1 values indicate higher silt and clay content, which decreased over time. Both plots indicate that sediment variable characteristics do not change based on climatic changes (Figure S1zA) nor seasonal changes (Figure S1zB).


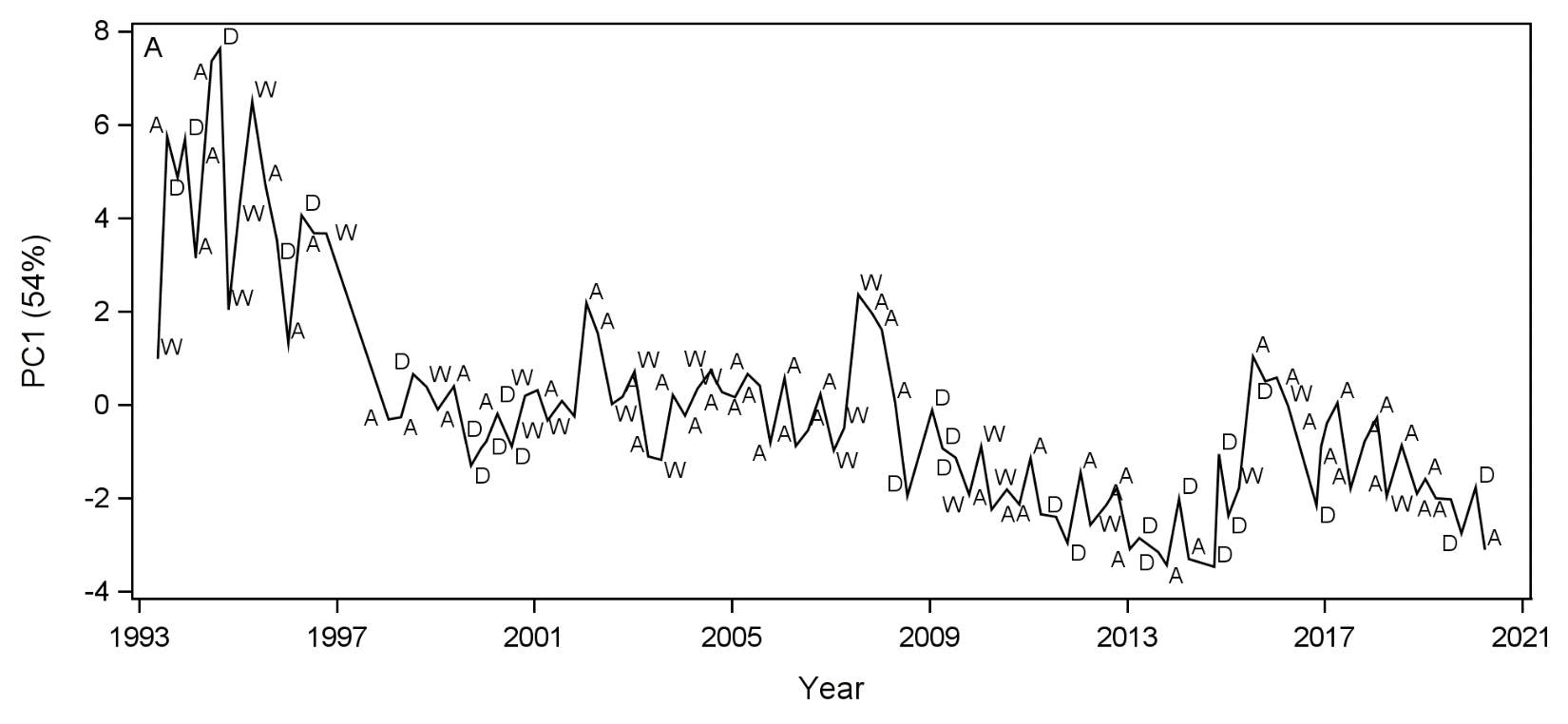

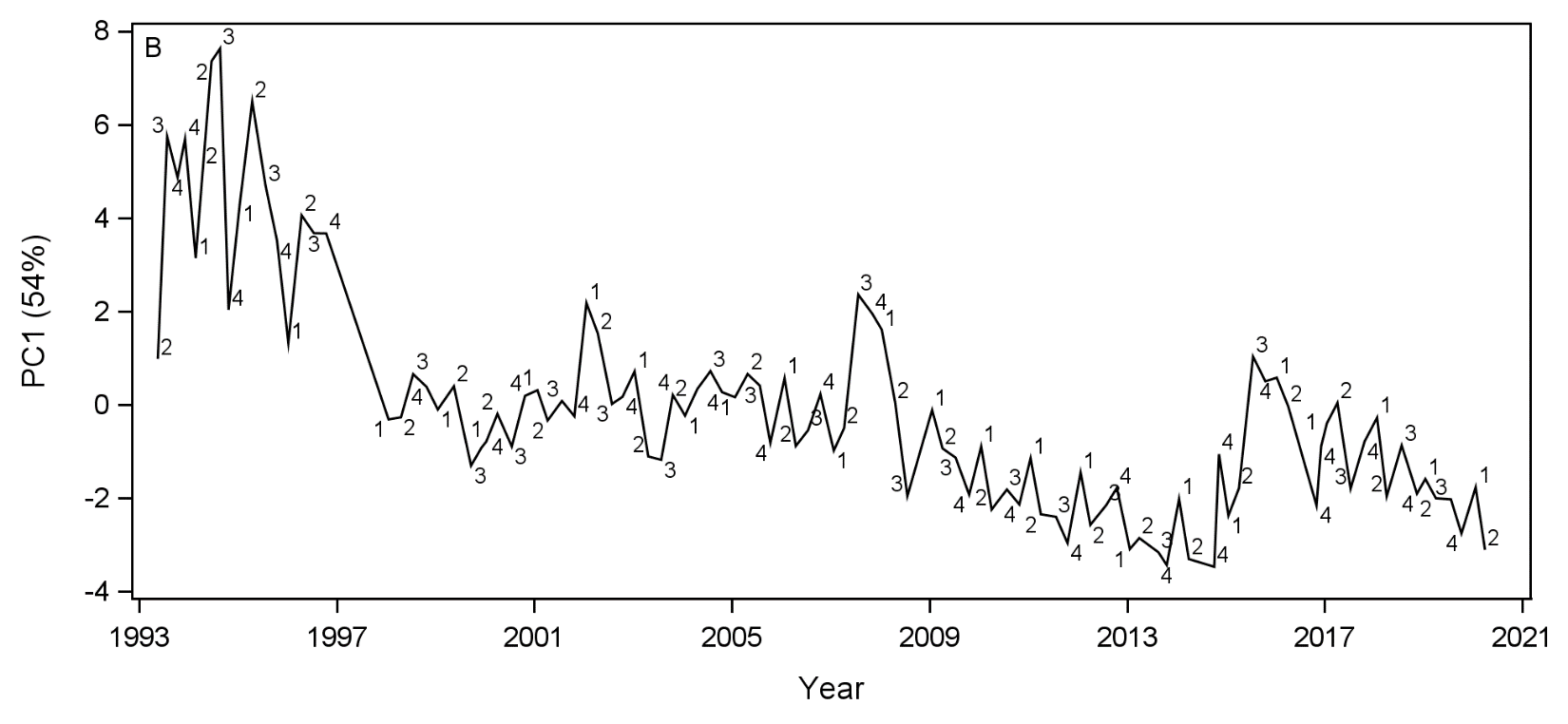


Figure S1z. Sediment PC1 for all trips. A) Sampling scores for PC1 where symbols represent climatic periods. W = wet, A = average, D = dry. B) Seasonality sample scores for PC1 where symbols represent seasonality. 1 = winter, 2 = spring, 3= summer, 4 = fall.

Sediment samples scores for PC 2 were plotted over time from 1993 to 2020 Both plots reaffirm that sediment fluctuations do not change based on climatic changes (Figure S1aaA) nor seasonal changes (Figure S1aaB).


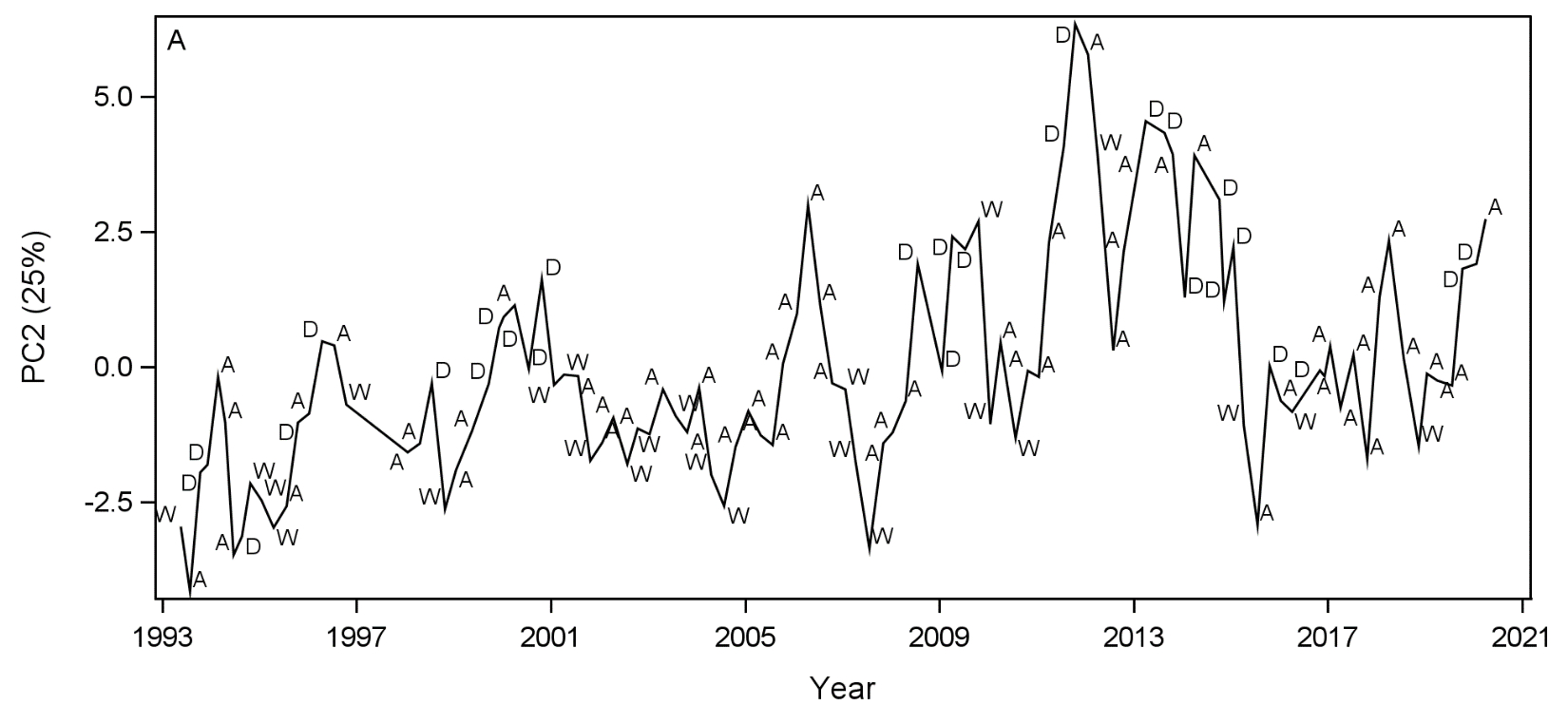

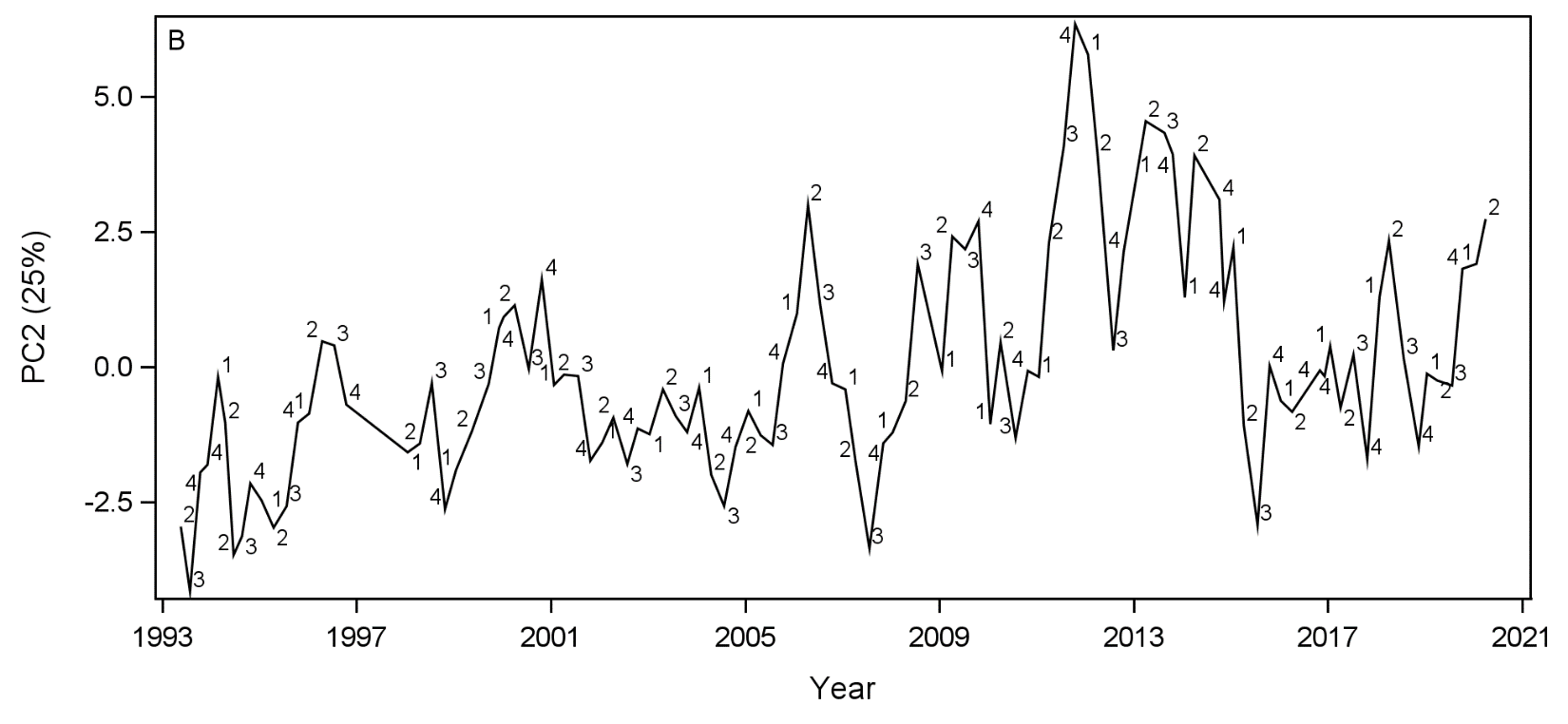


Figure S1aa. Sediment PC2 for all trips. A) Sampling scores for PC2 where symbols represent climatic periods. W = wet, A = average, D = dry. B) Seasonality sample scores for PC2 where symbols represent seasonality. 1 = winter, 2 = spring, 3= summer, 4.
